# Combining Raman Spectroscopy, DFT Calculations, and Atomic Force Microscopy in the Study of Clinker Materials

**DOI:** 10.3390/ma14133648

**Published:** 2021-06-30

**Authors:** Vlasta Mohaček-Grošev, Marija Đuroković, Aleksandar Maksimović

**Affiliations:** 1 Centre of Excellence for Advanced Materials and Sensing Devices, Ruđer Bošković Institute, Bijenička Cesta 54, 10000 Zagreb, Croatia; Aleksandar.Maksimovic@irb.hr; 2 Institut IGH d.d., Janka Rakuše 1, 10000 Zagreb, Croatia; marija.djurokovic@igh.hr

**Keywords:** Portland cement clinker, Raman mapping, alite, belite, phonon density of states, CRYSTAL09

## Abstract

Raman spectroscopy and Raman mapping analysis, combined with density functional theory calculations were applied to the problem of differentiating similar clinker materials such as alite and belite. The Portland cement clinker 217 (further: clinker) was analysed using colocalised Raman mapping and atomic force microscopy mapping, which provided both spatial and chemical information simultaneously. The main constituents found in the clinker were alite, belite, portlandite, amorphous calcium carbonate, and gypsum. Since phonon bands of alite and belite greatly overlap, and their distinction is important for the hydration process during cement setting, we provided the calculated phonon density of states for alite Ca_3_SiO_5_ (<M>*Pc* structure) and belite Ca_2_SiO_4_ (β *P2*_1_/*n* structure) here for the first time. Both calculated phonon densities have similar distribution of phonon modes, with a gap between 560 and 810 cm^−1^. A comparison of the calculated phonon frequencies for Ca_3_SiO_5_ and Ca_2_SiO_4_ shows that the lowest calculated phonon frequency of β-Ca_2_SiO_4_ lies at 102 cm^−1^, while for <M>*Pc* alite the lowest phonon frequency is predicted at 27 cm^−1^. Low frequency Raman spectroscopy could therefore be used for a clearer distinction of these two species in a clinker material.

## 1. Introduction

Portland cement was invented at the beginning of 19th century, partly using previous knowledge from ancient Greece and Rome [1,2]. It is a powder material that, when in contact with water, transforms into a hardened cement paste by chemical reactions and physical processes. Portland cement is produced by milling Portland cement clinker with a few percentages of gypsum. The estimated world production of Portland cement in 2018 was 3.99 billion tones, of which China produced 54.5% [3]. Currently, there exist numerous efforts toward reducing the amount of CO_2_ emitted into the atmosphere during cement clinker production. In 2018, it amounted to 1.5 ± 0.12 GtCO_2_ [4].

The main components of Portland cement clinker are expressed as weight percentages of lime (CaO), silica (SiO_2_), alumina (Al_2_O_3_), and iron oxide (Fe_2_O_3_), but the actual components occur as the following compounds: alite (tricalcium silicate 3CaO·SiO_2_, C_3_S), belite (dicalcium silicate 2CaO·SiO_2_, C_2_S), tricalcium aluminate (3CaO·Al_2_O_3_, C_3_A), and tetracalcium aluminoferrite (4CaO·Al_2_O_3_·Fe_2_O_3_, C_4_AF). Although the relative amount of C3S, C2S, C3A, and C4AF can be estimated using the Bogue calculation [5], X-ray powder diffraction is the only exact physical method suitable for quantitative determination of phase composition [6]. According to Taylor [6], the following seven different polymorphs of alite have been confirmed in different temperature intervals: three triclinic phases, T1, T2, and T3; three monoclinic phases, M1, M2, and M3; and a rhombohedral phase, R, existing above 1070 °C. In clinkers produced in an industrial kiln, alite is mainly present in the form of M_1_ or M_3_ polymorph with 3 to 4% of substituent oxides such as MgO, alkaline sulfates or free lime. The content of MgO and sulfate (SO_3_) determines whether the R polymorph transforms into an M3 or M1 polymorph on cooling from 1450 °C [7,8]. Belite, Ca_2_SiO_4_, has the following five different polymorphs: α, α_H_’, α_L_’, β, and γ [9]. Industrial Portland cement contains belite that is almost entirely present in the form of monoclinic β-C2S, with 4–6% of substituting oxides, mainly Al_2_O_3_ and Fe_2_O_3_ [6].

The CO_2_ footprint of clinker phases decreases in the following order: C3S > C3A > C2S > C4AF [10]. Lowering the CO_2_ footprint is achieved either by including supplementary cementitious materials into the Portland cement clinker, or by using chemical systems based on sulfoaluminate, sulfoferrite, and fluoroaluminate [1]. Tetracalcium trialuminate sulfate (C4A3$\bar{S}$), also known as ye’elimite or Klein’s salt, is the main phase in calcium sulfoaluminate cement, accompanied by belite (belite calcium sulfoaluminate clinker) or alite (alite calcium sulfoaluminate). Only one third of the CO_2_ released by the production of C3S is released during production of ye’elimite [11].

This work is planned as a first step for undertaking Raman/AFM mapping analysis of the calcium-silicate-hydrate (C-S-H) that develops upon cement hydration [12,13]. Several Raman mapping analyses on a hydrated clinker have been performed [14,15,16,17], which have proven that Raman spectroscopy is a technique by which water and hydrates can be successfully monitored. Additionally, atomic force microscopy was used for pore size determination [13]. As stated by Stutzmann et al. [18], in order to obtain high contrast scanning electron microscopy images of the clinker, samples need to be embedded in epoxy resin, cut, and polished, first by using silicon carbide paper of increasing degrees of grit and then, using a diamond paste with finer and finer dimensions of particles. Superposition of the image obtained by backscattered electrons and the images obtained using an energy dispersive X-ray analyser for each selected element (e.g., S, K, Al, Mg, Fe, Na...), using appropriate software, allows one to obtain a segmentation image showing regions of spatial distribution of chosen elements. This contemporary golden standard procedure is a sequel to the light microscopy of the Portland cement clinker of Henry de Chatelier (1887), which is still very useful in distinguishing alite and belite microcrystals [19]. Two major modes of operation for obtaining images using atomic force microscopy, tapping, and contact mode, have both been used in the study of clinker material [20,21]. Here, we opted for the examination of an untreated clinker grain using atomic force microscopy.

Proper assignment of the vibrational bands observed for dry clinker material is a prerequisite for confident analysis of Raman cement’s hydration spectra. In spite of an abundance of spectroscopic studies on single crystal Ca(OH)_2_, for example (Dawson et al. [22], Buchanan et al. [23], Oehler and Günthard [24], and online database spectra [25]), one can find band assignments of Ca(OH)_2_ in the studies of Raman mapping of cement paste that contradict them [17]. A theoretical calculation of the vibrational density of states of crystalline oxide phases helps to discern fundamental phonon bands from overtone and combination bands, as well as from spectral features of other chemical species, e.g., CaO crystallizes in the *Fm*$\bar{3}$*m* space group and disposes with a single triply degenerate phonon of *F_u_* symmetry, which is only observable in a far infrared absorption spectrum. Its second-order Raman spectrum was discussed in the work by Voisin and Mon [26] and Rieder et al. [27]. Quicklime exposed to atmosphere reacts immediately with water vapour and carbon dioxide, producing calcium hydroxide and different polymorphs of calcium carbonate, as documented, e.g., in the work of S. Martínez-Ramírez et al. [28], López-Arce et al. [29], Dubina et al. [30], Schmid and Dariz [31], and Kaszowska et al. [32]. Interpreting Raman spectra in the study of lime and clinker materials in general is not straightforward, because anomalous bands have been recorded to appear when lasers with a 1064-nanometer wavelength in the near infrared region was used [33]. Reproducible vibrational Raman spectra were obtained with green (514.5 nm, 532 nm), blue (488 nm), or red (632 nm) excitation laser lines, as demonstrated in articles by Bensted [34,35], Krishnamurthy and Soots (gypsum) [36], Conjeaud and Boyer [37], Handke [38], Ibááñez et al. [39], and Garg and Wang [40]. Often referred to as fluorescence bands or anomalous bands, strong maxima at 574 cm^−1^ for C3S or at 1023 cm^−1^ for C2S were observed by Dyer et al. [41], and similar bands found by Bonen et al. [42], Aminzadeh et al. [43], and Newman et al. [44], all on top of the very broad intense background. In their study of archaeological burial sites, the Raman spectra of lime substances were analysed with 785 and 1064 nm excitation by Schotsmans et al. and it was concluded that the origin of anomalous bands comes from electronic transitions [45]. In a paper by Potgieter-Vermaak et al. [46], one can find Raman bands of alite and belite tabulated with respect to the laser excitation source, while Skibsted and Hall discuss more Raman results [47]. A summary of Raman bands assignments can be found in a review article by Black [48].

The carbonation of calcium hydroxide nanoparticles exposed to external humidity was studied by López-Arce et al. [29], using both transmission electron microscopy, energy dispersive X-ray spectroscopy, environmental scanning electron microscopy combined with X-ray diffraction, and Raman spectroscopy. We briefly state their results in the following. X-ray diffractograms characteristic of portlandite are present after 7 days of hydration at relative humidity of 33%, while calcite and calcite monohydrate are identified together with a very broad maximum between 2θ = 16° and 28° much later after 28 days. The corresponding Raman spectra display only a portlandite band at 356 cm^−1^ and a calcite band at 1083 cm^−1^. Portlandite reacts with CO_2_ (relative humidity of 54%) and after 7 days forms mainly vaterite, identified by two bands in the interval between 1070 and 1090 cm^−1^. On increasing the humidity further to 75%, X-ray diffraction experiments gave evidence of an amorphous phase, together with aragonite, monohydrocalcite, and vaterite [29]. The corresponding Raman spectra display an intense band at 1086 cm^−1^, with weak bands at 280 and 710 cm^−1^; the band at 710 cm^−1^ was characteristic of aragonite. A detailed deconvolution of the Raman bands of carbonates formed at different depths in lime mortar was performed by Martínez-Ramírez et al. [28]. Although surface Raman spectra only permitted assignments of the bands that are characteristic of calcite, analysis at different depths revealed the presence of aragonite. No vaterite was detected in their samples.

In this work, we shall present the results on dry clinker material coming from three different sources—the first was granular, consisting mainly of alite in the M_3_ phase; the second was a Portland cement powder of the CEM I type; and the third, a polished calcium sulfoaluminate clinker. All of the clinker materials were examined using Raman imaging spectroscopy, while both atomic force microscopy and Raman imaging experiments were performed for clinker 217. To facilitate the assignment of portlandite, alite, and belite vibrational bands in the clinker spectra, the density functional theory was employed in the calculation of the vibrational density of states (VDOS) for Ca(OH)_2_, monoclinic belite (β phase) [49], and the averaged structure of alite polymorph belonging to the <M> *Pc* group [50,51,52], by means of a CRYSTAL09 program [53]. The calculation of alite’s and belite’s VDOS is, as far authors are aware, published for the first time.

## 2. Experimental

A specification of the composition of clinker 217 and sulfoaluminate cement is given in Table 1. In clinker 217, the CaO to SiO_2_ mass ratio was 3.24, and in calcium sulfoaluminate (CSA) cement it was 4.39. The mass percentage of MgO in clinker 217 was 2.17%, while it was 4.66% in CSA cement. Overall contribution of 3CaO·SiO_2_+2CaO·SiO_2_ in mass % was greater than 66.7% in clinker 217, which means that it satisfied the norm HRN EN 197-1:2012 as Portland cement clinker.

**Figure 1:**
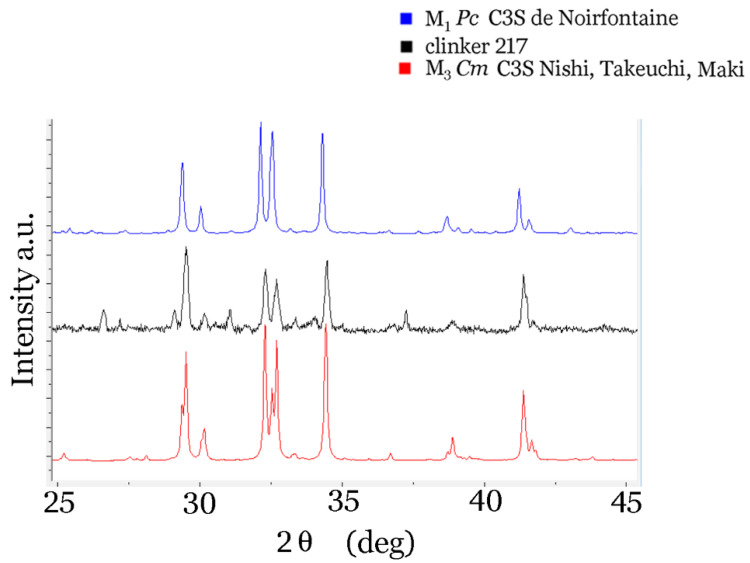
Comparison of the diffractogram of clinker 217 (middle) with the diffractogram of the phase M_1_ *Pc* [50] (above) and with the M_3_ *Cm* [69] (below) (interval 25°–45°).

X-ray powder diffraction data of clinker 217 were collected in the 2nd eta range 10–70° at room temperature on a Bruker D8 Discover diffractometer (Zagreb, Croatia) equipped with an LYNXEYE XE-T detector (Zagreb, Croatia), in theta–theta geometry. The results are shown in Figure 1. Cement powder of the CEM I type was also included in this study. It consisted of the clinker 217 with a few percent of gypsum added.

### 2.1. Computational Details

**Figure 2:**
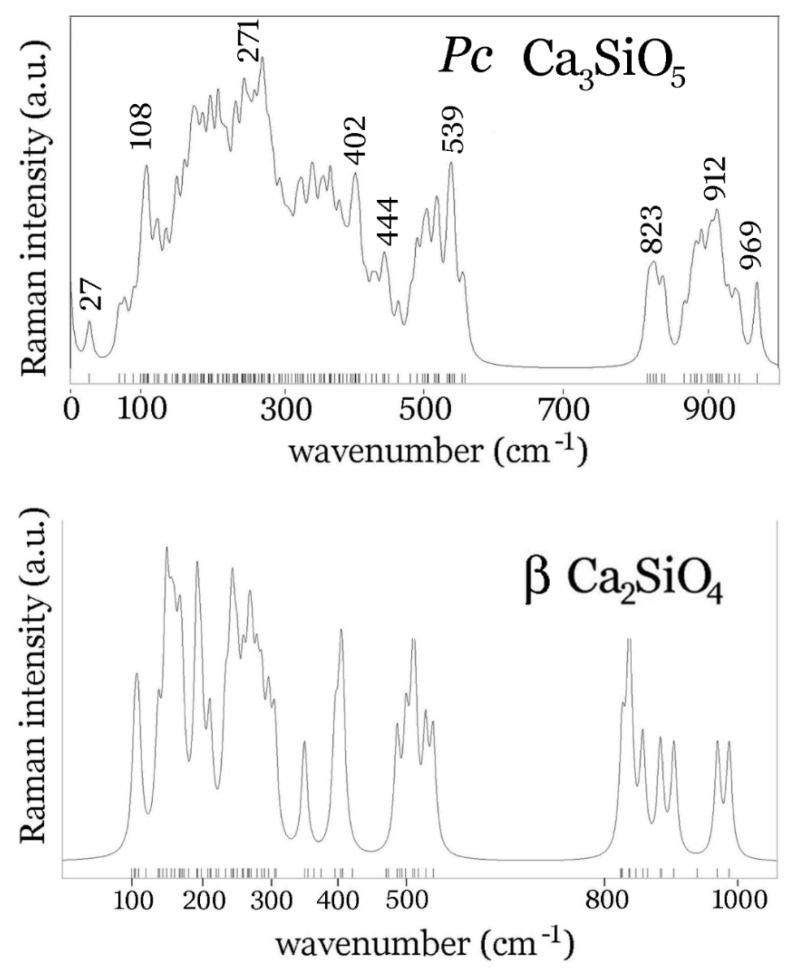
Calculated phonon density of states for Ca_3_SiO_5_ and Ca_2_SiO_4_ crystals.

For ab initio calculation of phonons of the Ca_2_SiO_4_ and Ca_3_SiO_5_, A CRYSTAL09 program was used [53], running on an HP Z640 workstation (Ruđer Bošković Institute, Zagreb, Croatia) using 8 processors. Atomic positions were optimised, starting from crystal geometry (C2S [49], C3S [50]) obtained using X ray powder diffraction, which provided sufficient precision for the calculation to converge and later gave all positive vibrations. The three lowest modes were acoustic modes with zero frequencies. For calcium and oxygen atoms, basis sets were taken from Valenzano’s work on calcite [54], while, for silicon, the basis set refined on Mg_3_Al_2_Si_3_O_12_ [55] and Mg_2_SiO_4_ [56] was used. Density functional theory was implemented using the correlation functional of Vosko, Wilk, and Nusair [57], and a local density approximation for the exchange part of the Hamiltonian [58]. The 35% of mixing of old wavefunction with the new one was applied in each cycle. The convergence criterion for energy was 10^−9^ Ha. The theoretical phonon density of states for C2S and C3S are presented in Figure 2. The outputs are available as Supplementary information.

### 2.2. Raman Spectroscopy of Clinker

Altogether, the Raman spectra were collected using the following three different instruments: HORIBA Jobin-Yvon T64000 triple monochromator located at the Ruđer Bošković Institute, Zagreb, Croatia; Labram HR Evolution located at the HORIBA Jobin-Yvon SAS Application laboratory at Lille, France; and the third instrument was WITec alpha 300 RA located at WITec factory in Ulm, Germany.

**Figure 3:**
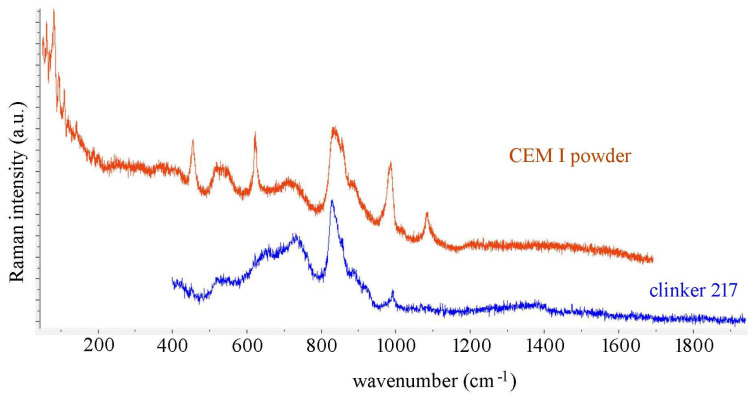
Comparison of Raman spectra of CEM I powder (clinker 217 with few percent of gypsum) and clinker 217.

**Figure 4:**
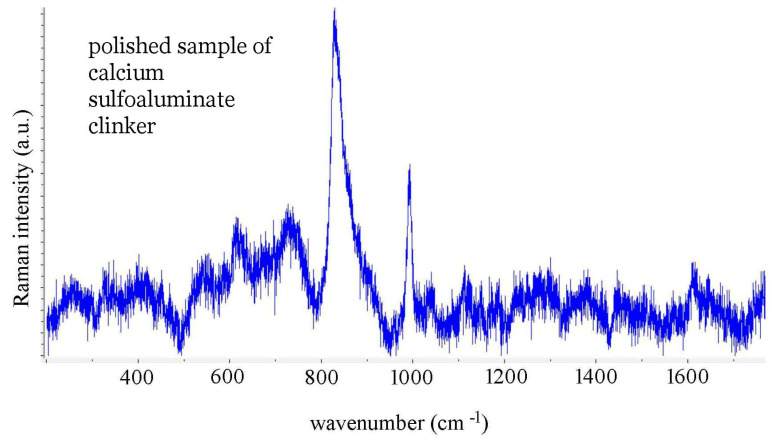
Raman spectrum of polished sample of calcium sulfoaluminate clinker.

The Raman spectra of clinker 217, cement powder of CEM I type, and of polished sulfoaluminate clinker were performed with a T64000 HORIBA Jobin-Yvon Raman spectrometer, Zagreb, Croatia, equipped with three gratings, having 1800 grooves per mm, 532 nm laser excitation, and a 50× long working distance objective. The time constant was between 5 and 20 s, and the number of accumulations varied up to 16. The laser power of 532 nm DPSS laser (ChangChun Industries Ltd., Changchun China) at the sample was 20 mW. Characteristic spectra are presented in Figure 3 and Figure 4, and in Supplementary Figures S1–S3.

**Figure 5:**
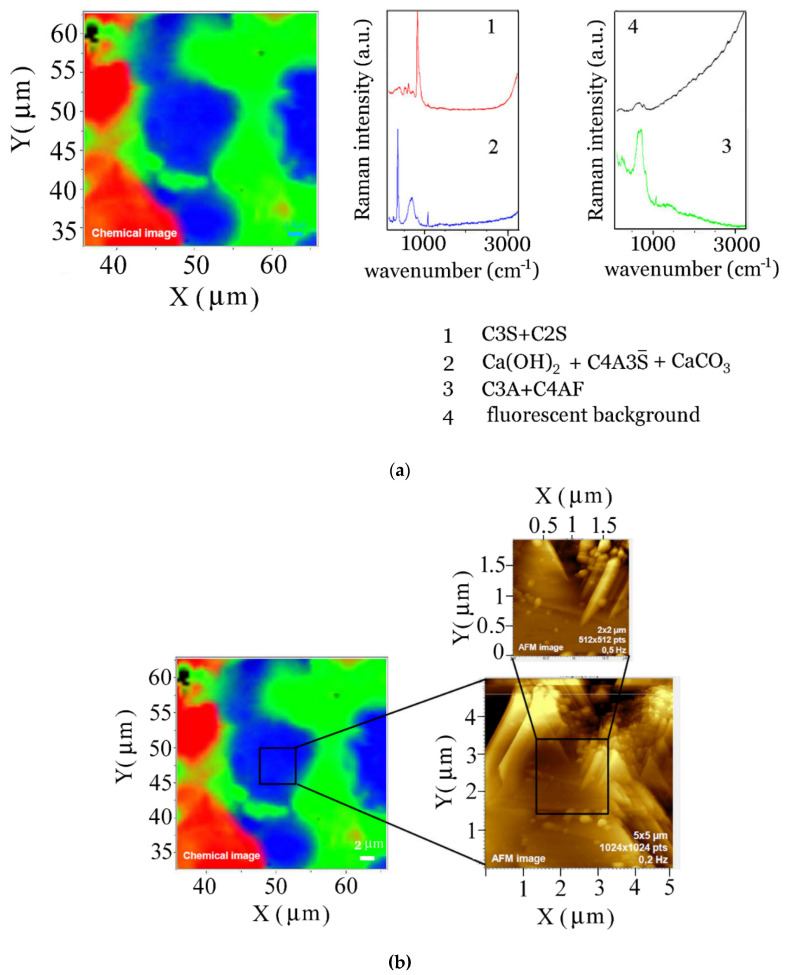
(**a**) Colour coded chemical image of clinker 217 obtained using Raman mapping with a HORIBA Jobin-Yvon Labram instrument. Spectra 1 and 4 were offset vertically for clarity. (**b**). Atomic force micrographs of the selected area of clinker 217 grain from (**a**). There are spherulites corresponding to amorphous CaCO_3_ visible on top of the flat surface of portlandite.

Colocalised Raman and atomic force microscopy mapping of granular clinker 217, which was performed with a Labram HR Evolution, used the 100× Mitutoyo objective on a 30-micrometer by 30-micrometer mapped area, used a scanning step of 0.5 µm, and 0.5 s time per acquisition point. The 532 nm laser with an operating power of 6 mW served as an excitation. The final Raman chemical image map (shown in Figure 5a) was formed using CLS fitting, a proprietary HORIBA software (Lille, France) procedure, which starts from a set of manually selected reference sets, shown on the right of Figure 5a, and finds a linear combination of reference spectra that best fits the observed data. Atomic force microscopy measurements were performed on the central part, 5 µm by 5 µm of the area was selected for Raman measurements (see Figure 5b) using AIST-NT Smart SPM (Novato, CA, USA) in the Normal force mode, using gold etched tip glued on a tuning fork.

**Figure 6:**
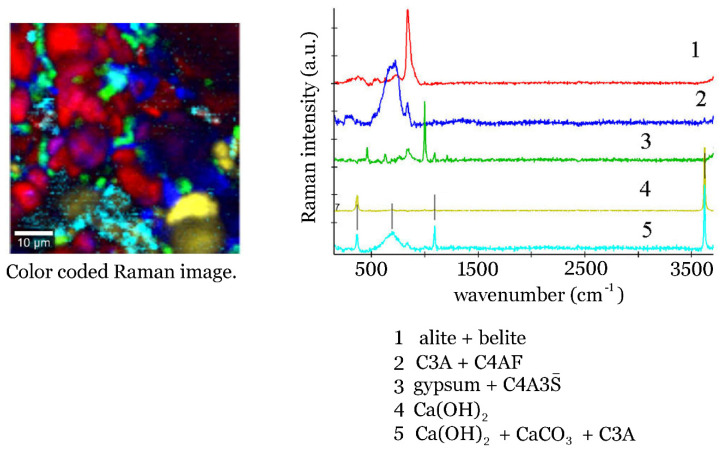
Colour coded Raman image of the 60-micrometer by 60-micrometer area of the clinker 217 grain. Spectral components given on the right-side result from the software analysis.

Raman spectral imaging on clinker 217 was performed at WITec company (Ulm, Germany) using WITec alpha 300 RA spectrometer on a scanned area of 60 µm by 60 µm, using 120 points per line and 120 lines per image with an integration time of 0.2 seconds. The optically pumped semiconductor laser was used for 532 nm excitation. Spectrometer UHTS 300 (Ulm, Germany) had a grating with 600 grooves per mm, and the CCD DU970_UVB detector (Oxford, UK). Spectra were analysed using WITec ProjectFOUR plus software software (Ulm, Germany) and, as a result, five characteristic spectra were selected. The sample area was colour coded and the obtained map is presented in Figure 6.

## 3. Results and Discussion

In order to correctly attribute the observed bands in the clinker, a calculation of phonons for Ca(OH)_2_ using LDA for exchange and VWN correlation pseudopotentials as implemented in the CRYSTAL09 program [53] was performed. The results are given in Table 2. Portlandite crystallizes in space group $P\bar{3}m1$ with a single formula unit per unit cell (*a* = 3.593 Å, b = 3.593 Å, c = 4.909 Å, α = 90°, β = 90°, γ = 120°, [59]).

One of the first applications of density functional theory to portlandite was that of Baranek et al. using the CRYSTAL98 code [60]. The net atomic charges obtained by Mulliken’s partition were (using LDA + VWN and B3LYP functionals [61,62,63,64,65,66]) +1.722*e* (Ca), −1.202*e* (O), and +0.341*e* (H). In comparison, Manzano et al. [67] used the periodic DFT code SIESTA with Perdew, Burke, and Ernzerhof’s exchange correlation functional [68] to obtain the averaged ionic charges of +1.41*e* (Ca), −0.85*e* (O), and +0.16*e* (H) for Ca(OH)_2_. Portlandite is a layered structure, the binding energy between two layers was calculated as −0.015 Ha [60]. The nature of the chemical bonds that Baranek et al. elucidated from Mulliken’s population data is as follows: the Ca-O bond has a very small electron population value (−0.036), hence it is ionic; while the O-H bond has a population value of 0.478 and is therefore mainly covalent. Additionally, the O-H stretching vibration that is observed in the infrared spectrum at 3644 cm^−1^ and at 3620 cm^−1^ in the Raman spectrum [22] proves the covalent character of the O-H bond in Ca(OH)_2_. Manzano et al. studied the hydration of the calcium oxide surface and concluded that the CaO surface will keep its structure for water amounts up to 6.43 molecules/nm^2^ and will distort at water amounts of about 8.58 molecules/nm^2^, thus providing the interval of water coverage important for the catalytic behaviour of CaO [67].

The strongest Raman bands of Ca(OH)_2_ are the band at 3620 and at 357 cm^−1^, both belonging to A_1g_ symmetry. The broad band at 680 cm^−1^, observed in the Raman spectrum of an oriented single crystal study by Dawson to E_g_ phonon [22], was assigned to adsorbed water by Dubina et al. [30]. Dubina et al. reported X-ray diffraction patterns of lime at 10, 20, 40, 60, and 80% of relative humidity, showing the peaks corresponding to portlandite and to carbonation products appearing at a relative humidity of 40% and higher [30]. A band at 1083 cm^−1^ in the Raman spectra of lime at 10% relative humidity was assigned to amorphous CaCO_3_, while it was not possible to detect this amorphous material in X-ray diffractograms. At 40% relative humidity, the diffractograms of lime contained peaks corresponding to aragonite (701, 706 cm^−1^), vaterite (1075 cm^−1^), and calcite CaCO_3_ (1085 cm^−1^) [30].

In Figure 1, a comparison of the calculated X-ray diffraction alite powder patterns for the M_1_ *Pc* structure determined by de Noirfontaine et al. [50,51,52], and the *Cm* structure determined by Nishi, Takeuchi and, Maki [69] for M_3_ phase with experimental powder X-ray diffraction pattern of clinker 217 is given. There is an overall agreement of clinker 217 maxima with those of Nishi et al., and we conclude that the alite present in clinker 217 is dominantly monoclinic M_3_, of space group *Cm.* In order to be able to interpret the Raman spectra of clinker samples, we calculated the phonon density of states for the monoclinic C3S *Pc* structure, as determined by de Noirfontaine et al. [50,51,52]. The parameters of the C3S structure are compared with those of β-C2S in Table 3. Atoms have partial occupancy in the structure determined by Nishi, and that option is not implemented in the CRYSTAL09 program that we had at our disposal. Therefore, we chose the M_1_ *Pc* structure for the calculation of phonons. The *Pc* structure of Noirfontaine et al. has only a glide plane placed at *0*, *b/2,* and at *b*. For this structure, we succeeded in obtaining all the positive frequencies for the 162 modes that are observable both in the Raman and in the infrared spectrum. Both the C3S and C2S structures were successfully optimised using OPTGEOM command. Various functions (PBE, LDA + VWN, PBESOL, etc.) gave slightly different total energies. The final result for the C3S structure with 54 atoms in a unit cell was −16140.66 Ha, and for the *P2_1_/n* C2S structure, −7757.78 Ha, when VWN/LDA correlation/exchange functions were used.

The results are compared with the Raman spectrum of belite calculated for the *P2_1_/n* crystal structure of the β polymorph [49], where in total 21 A_g_ and 21 B_g_ modes are expected, in Figure 2. As one can see, both for C3S and C2S, there is a gap in the calculated phonon densities, from 560 to 810 cm^−1^, present both for M1 (de Noirfontaine *Pc*) and for the β polymorph of C2S. This spectral window offers the possibility for a confident assignment of bands originating from other chemical species such as calcium aluminates. Contrary to Ca_3_SiO_5_, β-Ca_2_SiO_4_ has no bands below 100 cm^−1^, as predicted by this calculation. This fact could be exploited in the assignment of Raman bands of cement powder and the clinker if one were to record spectra without the interference of the rotational spectrum of air. This is, however, highly unlikely unless a vacuum chamber is used, given the long acquisition times necessary to obtain a good signal to noise ratio (see Figure 3, where a low frequency region of the Raman spectrum of cement powder is included, and it contains rotational bands of air below 100 cm^−1^).

One can correlate atomic motions in C3S and C2S with the corresponding phonon frequencies given in the outputs of CRYSTAL09 program available in the Supplementary Material by uploading the outputs to the website of the CRYSPLOT program [70]. In this way, one can assign the bands between 800 and 1000 cm^−1^ to Si-O stretching motions, while translational motions of calcium, oxygen, and silicon tetrahedra have their corresponding bands below 560 cm^−1^. In Table 4, the position of the observed Raman bands of the CEM I powder, clinker 217, and the sulfoaluminate clinker are compared and assigned [48]. The representative spectra are displayed in Figure 3 and Figure 4 and in Supplementary Figures S1–S3.

In the Raman spectrum of CEM I, the sharp bands observed at 985, 623, and 456 cm^−1^ (Figure 3) that correspond to vibrations of SO_4_^2−^ tetrahedra, are also present in the spectrum of gypsum, CaSO_4_∙2H_2_O (see Supplementary Figure S1), but not in the Raman spectrum of clinker 217 (Figure 3). A sharp band at 993 cm^−1^ in the Raman spectrum of the calcium sulfoaluminate clinker is assigned to the symmetric stretching of SO_4_^2−^ coming from ye’elimite (Figure 4). The broad bands in the 600–800 cm^−1^ interval are assigned mainly to tricalcium aluminate C3A or tetracalcium aluminate ferrite, with the band at 658 cm^−1^ in the spectrum of clinker 217 left unassigned. Black reported the frequent observation of a band at 642 cm^−1^ in alite by several groups, without assignment [48].

One of the key advantages of Raman spectroscopy is its ability to provide information on the chemical composition of an untreated sample. As Figure 3 and Figure 4, and Supplementary Figures S1–S3, show, the spectral information obtained may differ from point to point on the sample, since the clinker and cement powder are highly heterogeneous.

One can acquire spectral information for the whole surface by using a Raman imaging procedure. Scanning the surface sequentially and storing spectra gives information on the spatial distribution of the chemical species present. One such Raman mapping was performed on a 30-micrometer by 30-micrometer area of a clinker 217 grain using a HORIBA Jobin-Yvon Labram instrument (Lille, France), and for each point a “CLS fitting procedure” was performed. This procedure found a linear combination of the reference component spectra displayed on the right of Figure 5a, which best fits the raw data for every point on the map. The reference spectra were selected manually within the spectral array of the map. The spectrum denoted with **1** is mainly alite and belite (818, 869 cm^−1^); **2** has a strong band at 346 cm^−1^, corresponding to portlandite Ca(OH)_2_, and an amorphous CaCO_3_ band at 1073 cm^−1^; **3** has a strong contribution from calcium aluminates and calcium aluminoferrites (710 cm^−1^); and **4** is mainly a fluorescent background. By colour coding each area with the same colour that corresponds to the predominating spectrum of that area, one obtains the image on the left of Figure 5a.

Choosing a smaller area assigned to portlandite and performing an atomic force microscopy scan across a 5-micrometer by 5-micrometer square, one observes the layered structure of Ca(OH)_2_ (Figure 5b).

When a smaller part of that area (2 µm by 2 µm square), is scanned further, one observes spherulites characteristic of amorphous calcium carbonate [71]. Another Raman mapping experiment was performed using a WITec alpha 300 RA instrument and the results are displayed in Figure 6, using a red colour for the distribution of alite and belite, while the blue colour corresponds to tricalcium aluminate and calcium aluminoferrite. Sulfates can be detected in the clinker as well, and the strongest symmetric stretching mode ν_1_ (SO_4_^2−^) is clearly visible in the spectral component 3 that is colour coded green (see Supplementary Figure S1 where the spectrum of gypsum, CaSO_4_∙2H_2_O, is presented). The remaining two characteristic spectra are that of Ca(OH)_2_, with two prominent bands at 356 and 3620 cm^−1^ (component 5, colour code yellow), and calcium carbonate mixed with portlandite (component 5, light blue).

A similar mapping procedure was conducted by Higl et al. while following the hydration of synthetic m-C3S having a water to cement ratio of 0.5 [16]. The five significantly different spectra they identified were m-C3S; β-C2S; two hydration products, Ca(OH)_2_ and C-S-H; and the fifth component was an unresolved spectrum due to surface roughness. In another study of cement hydration by Torres-Carrasco, the symmetric stretching band of the SO_4_ group was observed at 996 cm^−1^ in ettringite shifts to lower wavenumbers on cement hydration, while the ν_1_(SO_4_) of monosulfate rises at 993 cm^−1^ [14]. Polymerization of silicate chains causes the appearance of additional Si-O stretching bands in Raman spectra, mainly above 1010 cm^−1^ and below 1050 cm^−1^ [15]. Infrared spectroscopy has been applied for studying C3S polymorphs as well, but the broadness of the bands and their overlap are significantly greater than in Raman spectra [72]. The limitations of Raman mapping procedure are the simultaneous observation of spectral bands originating from different compounds, which can overlap and complicate the assignment.

## 4. Conclusions

The problem of differentiating similar clinker materials, such as alite and belite, was undertaken by means of Raman spectroscopy and Raman mapping analysis, combined with density functional theory calculations. Raman spectroscopy has been used in the study of dry clinker, ordinary Portland cement, and calcium sulfoaluminate cement and the spectra interpreted by comparison with previous results from the literature and our own calculations of the vibrational density of states performed for crystalline β-Ca_2_SiO_4_ and monoclinic *Pc* Ca_3_SiO_5_. The predicted phonon density of states, both for C2S and C3S, displays no vibrational bands in the 560–810 cm^−1^ interval; therefore, tricalcium aluminate or tetracalcium aluminoferrite can be confidently assigned in that interval. Additionally, crystalline β-Ca_2_SiO_4_ has no phonons below 100 cm^−1^, while crystalline M_1_ Ca_3_SiO_5_ has the lowest phonon frequency at 27 cm^−1^. Low frequency Raman spectroscopy could, therefore, be used for a clearer distinction of these two species in a clinker material.

Raman mapping analysis combined with atomic force microscopy provided spatial distribution of different chemical species, requiring no prior chemical preparation of the sample. The limitations of the Raman mapping procedure are the simultaneous observation of spectral bands originating from different compounds, which can overlap and complicate the assignment. It was observed that Ca(OH)_2_ is often observed together with CaCO_3_ due to carbonation. Strong bands originating from sulfate groups in gypsum or ye’elimite are observed at 1018 and 993 cm^−1^, in the spectral interval where symmetric and antisymmetric Si-O stretching bands of silicate tetrahedra are expected in the study of cement hydration.

## Figures and Tables

**Table 1:** Chemical composition of clinker 217. Analysis performed using Institute IGH d.o.o. Zagreb.

*Properties*	*Clinker 217* *% Mass*	*Calcium Sulfoaluminate Cement* *% Mass*
*Loss on ignition*	0.25	0.92
*Sulfate content, SO_3_*	1.72	11.49
*Insoluble residue in HCl i Na_2_CO_3_*	0.50	2.54
*Insoluble residue in HCl i KOH*	0.55	1.96
*Sulfide content, S^2−^*	0.02	0.00
*Manganese oxide content, MnO*	0.14	0.157
*Total silica content SiO_2_*	20.24	9.12
*Iron (III) oxide content, Fe_2_O_3_*	2.89	1.61
*Aluminium oxide content, Al_2_O_3_*	4.56	32.82
*Calcium oxide content, CaO (13.14)*	65.50	40.08
*Magnesium oxide content MgO (13.15)*	2.17	4.66
*Chloride content, Cl^−^*	0.014	0.188
*Sodium oxide content, Na_2_O*	0.26	0.48
*Potassium oxide content, K_2_O*	1.07	0.71
*Sodium oxide equivalent, Na_2_O*	0.96	1.03
*Carbon dioxide content, CO_2_*	0.00	0.22

**Table 2:** Comparison of calculated with observed phonons in Ca(OH)_2_ (cm^−1^).

Phonon Symmetry	Calculated, This Work (CRYSTAL09)	Observed (Dawson et al. [22])
A_2u_	3662	3640
A_1g_	3626	3620
E_g_	721	680
E_u_	377	373
A_1g_	374	357
A_2u_	369	334
E_u_	309	287
E_g_	265	252

**Table 3:** Comparison of two monoclinic crystal structures of Ca_3_SiO_5_ with the structure of β-C2S.

Compound	C3S	C3S	C2S
space symmetry	*Pc*	*Cm*	*P2_1_/n*
structure type	M1	M3	*β*
*a*	9.2912 Å	33.083 Å	5.5075 Å
*b*	7.059 Å	7.027 Å	6.7509 Å
*c*	12.2575 Å	18.499 Å	9.3055 Å
*β*	116.03°	94.12°	94.597°
references	de Noirfontaine [50,51,52]	Nishi et al. [69]	Jost [49]

**Table 4:** Observed Raman bands of cement CEM I powder, clinker 217 grains, and polished calcium sulfoaluminate clinker embedded in resin together with the assignment of bands (cm^−1^). Abbreviations: C3S—Ca_3_SiO_5_, C2S—Ca_2_SiO_4_, C3A—3CaO∙Al_2_O_3_, C4AF—CaO∙Al_2_O_3_∙Fe_2_O_3_.

CEM I Cement Powder (Figure 3)	Clinker 217 Grain (Figure 3)	Calcium Sulfoaluminate Clinker, Place 1 (Figure 4)	Calcium Sulfoaluminate Clinker, Place 2 (Supplementary Figure S3)	Assignment
			1117	ν_3_ (SO_4_^2−^)
1084				calcite CaCO_3_
			1040	
1018				CaSO_4_ $\cdot \frac{1}{2}\cdot$H_2_O
	994	993	993	ν_1_ (SO_4_^2−^)
985				K_2_SO_4_
	921	920		C3S
	893	890		C2S
883				C3S
856	854		858	C3S, C2S
836	830	832	833	C3S
		746		C3A, C4AF
	733		732	C4AF
718		716		C3A
	658			?
623			620	ν_4_ (SO_4_^2−^)
541	546		549	C2S, C3S
522				C2S, C3S
456				ν_2_ (SO_4_^2−^)

## Supplementary Materials

The following are available online at https://www.mdpi.com/article/10.3390/ma14133648/s1, Figure S1: Comparison of Raman spectra of gypsum and Portland cement, Figure S2: Raman spectra of CEM I powder, collected at four different sites. Figure S3: Raman spectrum of polished sample of calcium sulfoaluminate clinker.
