# Supplementary material for: Combining Raman Spectroscopy, DFT Calculations, and Atomic Force Microscopy in the Study of Clinker Materials
Source: Materials (Basel). 2021 Jun 30;14(13):3648. doi: 10.3390/ma14133648 (PMC8269705; doi:10.3390/ma14133648)
Supplement: Supplementary file 1 [file materials-14-03648-s001.zip › materials-1249732-supplementary.pdf]

materials

MDPI

```
OUTPUT OF CRYSTAL09 PROGRAM FOR Ca2SiO4 belite
======================================================================
=:= date: sub, 9.03.2019. 10:48:14 CET
=:= hostname: lmfz640
=:= system: Linux lmfz640 4.15.0-46-generic #49-Ubuntu SMP Wed Feb 6 09:33:07 UTC 2019 x86_64 x86_64 x86_64
GNU/Linux
=:= user: vlasta
=:= scratch directory: /home/vlasta/crystal/tmp/tmp1870
=:= Available predefined $Pcrystal_ENV={isis,neolith,kalkyl,lenngren,interactive,localhost}
=:= $Pcrystal_ENV not defined by user. Trying to guess...
=:= None of the predefined parallel enviroments matched. Configuring "localhost"...
=:= Using predefined environment: localhost
=:= mpirun to use: /usr/bin/mpirun.mpich
=:= Got NSLOTS=8
=:= ======================================================================
Ca2SiO4 cif file
CRYSTAL
1  2  0
P 1 21/N 1
  5.5075  6.7509  9.3055 94.597
7
20    2.259743027399E-01  3.440807844666E-01  4.324013106182E-01
20   -2.789152102887E-01 -4.958528969244E-01  2.015579430480E-01
14    2.679963311619E-01 -2.171331715239E-01  4.178753611653E-01
 8    2.175666700724E-01  1.543947662208E-02  4.443157037911E-01
 8    1.015604961355E-02 -3.312309356329E-01  3.638776329533E-01
 8    4.773877212424E-01 -2.478022947534E-01  3.033977422278E-01
 8    3.504877954991E-01 -3.317228088380E-01 -4.281642024754E-01
FREQCALC
END
END
20 7
0 0 8 2. 1.
 191300. 0.0002204
 26970. 0.001925
 5696. 0.01109
 1489.4 0.04995
 448.3 0.17014
 154.62 0.3685
 60.37 0.4034
 25.09 0.1452
0 1 6 8. 1.
 448.6 -0.00575 0.00847
 105.7 -0.0767 0.06027
 34.69 -0.1122 0.2124
 13.50 0.2537 0.3771
 5.820 0.688 0.401
 1.819 0.349 0.198
0 1 5 8. 1.
 20.75 -0.0020 -0.0365
 8.40 -0.1255 -0.0685
 3.597 -0.6960 0.1570
 1.408 1.029 1.482
 0.726 0.944 1.025
0 1 1 2. 1.
 0.453 1. 1.
0 1 1 0. 1.
 0.295 1. 1.
0 3 2 0. 1.
 3.1910 0.160
 0.8683 0.313
0 3 1 0. 1.
 0.2891 0.406
14 6
0 0 8 2. 1.0
 87645.8 0.000237
 12851.8 0.00192
 2786.28 0.0109
 728.043 0.0496
 219.516 0.1668
 75.9006 0.363
 29.4602 0.4051
 11.9891 0.1504
0 1 6 8. 1.0
 165.958 -0.00884 0.00909
 39.3727 -0.0859 0.0601
 12.7112 -0.0712 0.1952
```

```
 4.777 0.4147 0.3384
 1.8482 0.6168 0.3006
 0.7365 0.1154 0.0648
0 1 3 4. 1.
 4.1752 -0.0199 -0.0087
 1.4472 -0.1864 -0.00438
 0.5023 0.0967 0.2207
0 1 1 0. 1.
 0.322 1. 1.
0 1 1 0. 1.
 0.13 1. 1.
0 3 1 0. 1.
 0.6 1.
8 6
0 0 8 2.0 1.0
 8020. 0.001080
 1338. 0.008040
 255.4 0.053240
 69.22 0.168100
 23.90 0.358100
 9.264 0.385500
 3.851 0.146800
 1.212 0.072800
0 1 4 6. 1.0
 49.43 -0.008830 0.009580
 10.47 -0.091500 0.069600
 3.235 -0.040200 0.206500
 1.217 0.379000 0.347000
0 1 1 0. 1.0
 0.486 1. 1.
0 1 1 0. 1.0
 0.1925 1. 1.
0 3 1 0. 1.
 2.0 1.
0 3 1 0. 1.
 0.500 1.
99 0
ENDBS
SCFDIR
DFT
CORRELAT
VWN
EXCHANGE
LDA
XLGRID
END
SHRINK
 6 6 6
TOLINTEG
6 6 6 6 12
FMIXING
35
MAXCYCLE
 800
LEVSHIFT
6 1
TOLDEE
10
END
=:= Executing: /usr/bin/mpirun.mpich -np 8 -v -machinefile /home/vlasta/crystal/tmp/tmp1870/machines
/home/vlasta/crystal/tmp/tmp1870/Pcrystal
host: lmfz640

==========================================================================================
mpiexec options:
----------------
  Base path: /usr/bin/
  Launcher: (null)
  Debug level: 1
  Enable X: -1

  Global environment:
  -------------------
    EXCITINGVISUAL=/usr/share/exciting/xml/visualizationtemplates
    LS_COL-
ORS=rs=0:di=01;34:ln=01;36:mh=00:pi=40;33:so=01;35:do=01;35:bd=40;33;01:cd=40;33;01:or=40;31;01:mi=00:su=37;4
1:sg=30;43:ca=30;41:tw=30;42:ow=34;42:st=37;44:ex=01;32:*.tar=01;31:*.tgz=01;31:*.arc=01;31:*.arj=01;31:*.taz
=01;31:*.lha=01;31:*.lz4=01;31:*.lzh=01;31:*.lzma=01;31:*.tlz=01;31:*.txz=01;31:*.tzo=01;31:*.t7z=01;31:*.zip
=01;31:*.z=01;31:*.Z=01;31:*.dz=01;31:*.gz=01;31:*.lrz=01;31:*.lz=01;31:*.lzo=01;31:*.xz=01;31:*.zst=01;31:*.
```

```
tzst=01;31:*.bz2=01;31:*.bz=01;31:*.tbz=01;31:*.tbz2=01;31:*.tz=01;31:*.deb=01;31:*.rpm=01;31:*.jar=01;31:*.w
ar=01;31:*.ear=01;31:*.sar=01;31:*.rar=01;31:*.alz=01;31:*.ace=01;31:*.zoo=01;31:*.cpio=01;31:*.7z=01;31:*.rz
=01;31:*.cab=01;31:*.wim=01;31:*.swm=01;31:*.dwm=01;31:*.esd=01;31:*.jpg=01;35:*.jpeg=01;35:*.mjpg=01;35:*.mj
peg=01;35:*.gif=01;35:*.bmp=01;35:*.pbm=01;35:*.pgm=01;35:*.ppm=01;35:*.tga=01;35:*.xbm=01;35:*.xpm=01;35:*.t
if=01;35:*.tiff=01;35:*.png=01;35:*.svg=01;35:*.svgz=01;35:*.mng=01;35:*.pcx=01;35:*.mov=01;35:*.mpg=01;35:*.
mpeg=01;35:*.m2v=01;35:*.mkv=01;35:*.webm=01;35:*.ogm=01;35:*.mp4=01;35:*.m4v=01;35:*.mp4v=01;35:*.vob=01;35:
*.qt=01;35:*.nuv=01;35:*.wmv=01;35:*.asf=01;35:*.rm=01;35:*.rmvb=01;35:*.flc=01;35:*.avi=01;35:*.fli=01;35:*.
flv=01;35:*.gl=01;35:*.dl=01;35:*.xcf=01;35:*.xwd=01;35:*.yuv=01;35:*.cgm=01;35:*.emf=01;35:*.ogv=01;35:*.ogx
=01;35:*.aac=00;36:*.au=00;36:*.flac=00;36:*.m4a=00;36:*.mid=00;36:*.midi=00;36:*.mka=00;36:*.mp3=00;36:*.mpc
=00;36:*.ogg=00;36:*.ra=00;36:*.wav=00;36:*.oga=00;36:*.opus=00;36:*.spx=00;36:*.xspf=00;36:
    LD_LIBRARY_PATH=/usr/local/gaus/g16/bsd:/usr/local/gaus/g16:/usr/local/gaus/gv/lib
    GPAW_SETUP_PATH=/usr/share/gpaw-setups
    GAUSS_BSDDIR=/usr/local/gaus/g16/bsd
    GRA6_EXEDIR=/home/vlasta/crystal/crgra2006/bin/Linux-pgf
    CMR_REPOSITORY=/home/vlasta/cmr
    LC_MEASUREMENT=hr_HR.UTF-8
    SSH_CONNECTION=10.135.24.11 40589 10.135.24.38 22
    LESSCLOSE=/usr/bin/lesspipe %s %s
    LC_PAPER=hr_HR.UTF-8
    LC_MONETARY=hr_HR.UTF-8
    LANG=en_US.UTF-8
    g16root=/usr/local/gaus
    PSEUDO_DIR=/home/vlasta/md/potentials/UPF
    CRY2K6_GRA=/home/vlasta/crystal/crgra2006
    _DSM_BARRIER=SHM
    DACAPOPATH=/usr/share/dacapo-psp
    OPENBLAS_NUM_THREADS=1
    OLDPWD=/home/vlasta/crystal/tmp/c2s
    XCRYSDEN_TOPDIR=/usr/local/share/XCrySDen/
    TMP_DIR=/home/vlasta/crystal/tmp/tmp1870
    EXCITINGATAT=/home/vlasta/md/run/excitingscripts/ATAT@exciting
    GV_DIR=/usr/local/gaus/gv
    DACAPOEXE_SERIAL=/usr/bin/dacapo_serial.run
    CRY2K9_SCRDIR=/home/vlasta/crystal/tmp
    SIESTA_SCRIPT=/home/vlasta/bin/run_siesta.py
    LC_NAME=hr_HR.UTF-8
    GAUSS_LEXEDIR=/usr/local/gaus/g16/linda-exe
    XDG_SESSION_ID=65
    USER=vlasta
    EXCITINGRUNDIR=/home/vlasta/md/run
    CRY2K9_TEST=/home/vlasta/crystal/test_cases/inputs
    VERSION=v2_0_1
    HOTBIT_PARAMETERS=/usr/share/doc/hotbit/param/fixed_parameters
    KSSOLVPATH=/home/vlasta/md/kssolv
    NSLOTS=8
    GAUSS_ARCHDIR=/usr/local/gaus/g16/arch
    ABINIT_PP_PATH=/usr/local/share/abinit-pseudopotentials//GGA_PAW:/usr/local/share/abinit-pseudopoten-
tials//GGA_HGHK:/usr/local/share/abinit-pseudopotentials//GGA_FHI:/usr/local/share/abinit-pseudopoten-
tials//LDA_TM:/usr/local/share/abinit-pseudopotentials//LDA_PAW:/usr/local/share/abinit-pseudopoten-
tials//LDA_HGH:/usr/local/share/abinit-pseudopotentials//GGA_FHI:/usr/local/share/abinit-pseudopoten-
tials//LDA_FHI:/usr/share/doc/abinit-doc/tests/Psps_for_tests
    PWD=/home/vlasta/crystal/tmp/tmp1870
    EXCITINGROOT=/usr
    HOME=/home/vlasta
    CRY2K9_UTILS=/home/vlasta/crystal/utils09
    SSH_CLIENT=10.135.24.11 40589 22
    DFTB_COMMAND=dftb+
    TIMEFORMAT=   Elapsed time = %0lR
    GMXCMD=mpirun -np 4 mdrun_mpi
    PW_ROOT=/usr
    DFTB_PREFIX=/home/vlasta/md/potentials/slako/pbc-0-3/
    GAUSS_EXEDIR=/usr/local/gaus/g16/bsd:/usr/local/gaus/g16
    MALLOC_CHECK_=0
    SIESTA_PP_PATH=.
    CRY2K9_EXEDIR=/home/vlasta/crystal/bin/Linux-ifort
    EXCITINGASE=/home/vlasta/md/run/excitingscripts/ase
    ASE_ABINIT_COMMAND=mpirun -np 2 abinit < PREFIX.files > PREFIX.log
    LC_ADDRESS=hr_HR.UTF-8
    LC_NUMERIC=hr_HR.UTF-8
    DACAPOEXE_PARALLEL=/usr/bin/dacapo_mpi.run
    FLEUR_INPGEN=/home/vlasta/bin/inpgen.x
    SSH_TTY=/dev/pts/0
    MAIL=/var/mail/vlasta
    PAPERSIZE=a4
    ELK_SPECIES_PATH=/usr/local/share/elk-dft/species/
    TERM=xterm
    SHELL=/bin/bash
    CRY2K9_ROOT=/home/vlasta/crystal
    CRY2K9_ARCH=Linux-ifort
```

```
    CRY2K9_BIN=bin
    G16BASIS=/usr/local/gaus/g16/basis
    FLEUR=/home/vlasta/bin/fleur.x
    LAMMPS_COMMAND=/usr/bin/lammps
    SHLVL=2
    LANGUAGE=en_US:en
    PYTHONPATH=/usr/local/gaus/gauopen:/usr/local/gaus/g16/bsd:/usr/local/gaus/g16
    LC_TELEPHONE=hr_HR.UTF-8
    LOGNAME=vlasta
    DBUS_SESSION_BUS_ADDRESS=unix:path=/run/user/1003/bus
    XDG_RUNTIME_DIR=/run/user/1003
    EXCITINGSHARE=/usr/share/exciting
    HOTBIT_DIR=/usr/share/doc/hotbit/examples
    PATH=/home/vlasta/crystal/tmp/tmp1870::/usr/local/share/XCrySDen/:/usr/local/sbin:/usr/lo-
cal/bin:/usr/sbin:/usr/bin:/sbin:/bin:/usr/games:/usr/local/games:./:/home/vlasta/crystal/bin/Linux-
ifort:/home/vlasta/crystal/utils09:/home/vlasta/crystal/crgra2006:/home/vlasta/md/run/exciting-
scripts:/home/vlasta/md/run/excitingscripts/ATAT@exciting:/usr/local/gaus/gauopen:/usr/lo-
cal/gaus/g16/bsd:/usr/local/gaus/g16:/home/tmpacc/QuantumWise/atk/vnl/bin:/home/tmpacc/QuantumWise/atk/atkpy-
thon/bin:/home/tmpacc/QuantumWise/atk/license:/usr/local/share/XCrySDen//scripts:/usr/local/share/XCryS-
Den//util
    LC_IDENTIFICATION=hr_HR.UTF-8
    PERLLIB=/usr/local/gaus/gauopen:/usr/local/gaus/g16/bsd:/usr/local/gaus/g16
    EXCITINGSCRIPTS=/home/vlasta/md/run/excitingscripts
    PGI_TERM=trace,abort
    XCRYSDEN_SCRATCH=/tmp
    XLIB_SKIP_ARGB_VISUALS=1
    ATATROOT=/home/vlasta/md/run/excitingscripts/atat
    EXCITINGBIN=/usr/bin
    LESSOPEN=| /usr/bin/lesspipe %s
    OMP_NUM_THREADS=2
    LC_TIME=hr_HR.UTF-8
    _=/usr/bin/mpirun.mpich

  Hydra internal environment:
  ---------------------------
    GFORTRAN_UNBUFFERED_PRECONNECTED=y


    Proxy information:
    *********************
      [1] proxy: lmfz640 (8 cores)
      Exec list: /home/vlasta/crystal/tmp/tmp1870/Pcrystal (8 processes);


==================================================================================================

[mpiexec@lmfz640] Timeout set to -1 (-1 means infinite)
[mpiexec@lmfz640] Got a control port string of lmfz640:38501

Proxy launch args: /usr/bin/hydra_pmi_proxy --control-port lmfz640:38501 --debug --rmk user --launcher ssh --
demux poll --pgid 0 --retries 10 --usize -2 --proxy-id

Arguments being passed to proxy 0:
--version 3.3a2 --iface-ip-env-name MPIR_CVAR_CH3_INTERFACE_HOSTNAME --hostname lmfz640 --global-core-map
0,8,8 --pmi-id-map 0,0 --global-process-count 8 --auto-cleanup 1 --pmi-kvsname kvs_1911_0 --pmi-process-map-
ping (vector,(0,1,8)) --ckpoint-num -1 --global-inherited-env 93 'EXCITINGVISUAL=/usr/share/exciting/xml/vis-
ualizationtemplates' 'LS_COL-
ORS=rs=0:di=01;34:ln=01;36:mh=00:pi=40;33:so=01;35:do=01;35:bd=40;33;01:cd=40;33;01:or=40;31;01:mi=00:su=37;4
1:sg=30;43:ca=30;41:tw=30;42:ow=34;42:st=37;44:ex=01;32:*.tar=01;31:*.tgz=01;31:*.arc=01;31:*.arj=01;31:*.taz
=01;31:*.lha=01;31:*.lz4=01;31:*.lzh=01;31:*.lzma=01;31:*.tlz=01;31:*.txz=01;31:*.tzo=01;31:*.t7z=01;31:*.zip
=01;31:*.z=01;31:*.Z=01;31:*.dz=01;31:*.gz=01;31:*.lrz=01;31:*.lz=01;31:*.lzo=01;31:*.xz=01;31:*.zst=01;31:*.
tzst=01;31:*.bz2=01;31:*.bz=01;31:*.tbz=01;31:*.tbz2=01;31:*.tz=01;31:*.deb=01;31:*.rpm=01;31:*.jar=01;31:*.w
ar=01;31:*.ear=01;31:*.sar=01;31:*.rar=01;31:*.alz=01;31:*.ace=01;31:*.zoo=01;31:*.cpio=01;31:*.7z=01;31:*.rz
=01;31:*.cab=01;31:*.wim=01;31:*.swm=01;31:*.dwm=01;31:*.esd=01;31:*.jpg=01;35:*.jpeg=01;35:*.mjpg=01;35:*.mj
peg=01;35:*.gif=01;35:*.bmp=01;35:*.pbm=01;35:*.pgm=01;35:*.ppm=01;35:*.tga=01;35:*.xbm=01;35:*.xpm=01;35:*.t
if=01;35:*.tiff=01;35:*.png=01;35:*.svg=01;35:*.svgz=01;35:*.mng=01;35:*.pcx=01;35:*.mov=01;35:*.mpg=01;35:*.
mpeg=01;35:*.m2v=01;35:*.mkv=01;35:*.webm=01;35:*.ogm=01;35:*.mp4=01;35:*.m4v=01;35:*.mp4v=01;35:*.vob=01;35:
*.qt=01;35:*.nuv=01;35:*.wmv=01;35:*.asf=01;35:*.rm=01;35:*.rmvb=01;35:*.flc=01;35:*.avi=01;35:*.fli=01;35:*.
flv=01;35:*.gl=01;35:*.dl=01;35:*.xcf=01;35:*.xwd=01;35:*.yuv=01;35:*.cgm=01;35:*.emf=01;35:*.ogv=01;35:*.ogx
=01;35:*.aac=00;36:*.au=00;36:*.flac=00;36:*.m4a=00;36:*.mid=00;36:*.midi=00;36:*.mka=00;36:*.mp3=00;36:*.mpc
=00;36:*.ogg=00;36:*.ra=00;36:*.wav=00;36:*.oga=00;36:*.opus=00;36:*.spx=00;36:*.xspf=00;36:' 'LD_LI-
BRARY_PATH=/usr/local/gaus/g16/bsd:/usr/local/gaus/g16:/usr/local/gaus/gv/lib'
'GPAW_SETUP_PATH=/usr/share/gpaw-setups' 'GAUSS_BSDDIR=/usr/local/gaus/g16/bsd' 'GRA6_EX-
EDIR=/home/vlasta/crystal/crgra2006/bin/Linux-pgf' 'CMR_REPOSITORY=/home/vlasta/cmr' 'LC_MEASURE-
MENT=hr_HR.UTF-8' 'SSH_CONNECTION=10.135.24.11 40589 10.135.24.38 22' 'LESSCLOSE=/usr/bin/lesspipe %s %s'
'LC_PAPER=hr_HR.UTF-8' 'LC_MONETARY=hr_HR.UTF-8' 'LANG=en_US.UTF-8' 'g16root=/usr/local/gaus'
'PSEUDO_DIR=/home/vlasta/md/potentials/UPF' 'CRY2K6_GRA=/home/vlasta/crystal/crgra2006' '_DSM_BARRIER=SHM'
'DACAPOPATH=/usr/share/dacapo-psp' 'OPENBLAS_NUM_THREADS=1' 'OLDPWD=/home/vlasta/crystal/tmp/c2s' 'XCRYS-
```

```
DEN_TOPDIR=/usr/local/share/XCrySDen/' 'TMP_DIR=/home/vlasta/crystal/tmp/tmp1870' 'EXCIT-
INGATAT=/home/vlasta/md/run/excitingscripts/ATAT@exciting' 'GV_DIR=/usr/local/gaus/gv' 'DACAPOEXE_SE-
RIAL=/usr/bin/dacapo_serial.run' 'CRY2K9_SCRDIR=/home/vlasta/crystal/tmp' 'SI-
ESTA_SCRIPT=/home/vlasta/bin/run_siesta.py' 'LC_NAME=hr_HR.UTF-8' 'GAUSS_LEXEDIR=/usr/local/gaus/g16/linda-
exe' 'XDG_SESSION_ID=65' 'USER=vlasta' 'EXCITINGRUNDIR=/home/vlasta/md/run' 'CRY2K9_TEST=/home/vlasta/crys-
tal/test_cases/inputs' 'VERSION=v2_0_1' 'HOTBIT_PARAMETERS=/usr/share/doc/hotbit/param/fixed_parameters'
'KSSOLVPATH=/home/vlasta/md/kssolv' 'NSLOTS=8' 'GAUSS_ARCHDIR=/usr/local/gaus/g16/arch'
'ABINIT_PP_PATH=/usr/local/share/abinit-pseudopotentials//GGA_PAW:/usr/local/share/abinit-pseudopoten-
tials//GGA_HGHK:/usr/local/share/abinit-pseudopotentials//GGA_FHI:/usr/local/share/abinit-pseudopoten-
tials//LDA_TM:/usr/local/share/abinit-pseudopotentials//LDA_PAW:/usr/local/share/abinit-pseudopoten-
tials//LDA_HGH:/usr/local/share/abinit-pseudopotentials//GGA_FHI:/usr/local/share/abinit-pseudopoten-
tials//LDA_FHI:/usr/share/doc/abinit-doc/tests/Psps_for_tests' 'PWD=/home/vlasta/crystal/tmp/tmp1870' 'EXCIT-
INGROOT=/usr' 'HOME=/home/vlasta' 'CRY2K9_UTILS=/home/vlasta/crystal/utils09' 'SSH_CLIENT=10.135.24.11 40589
22' 'DFTB_COMMAND=dftb+' 'TIMEFORMAT=   Elapsed time = %0lR' 'GMXCMD=mpirun -np 4 mdrun_mpi' 'PW_ROOT=/usr'
'DFTB_PREFIX=/home/vlasta/md/potentials/slako/pbc-0-3/' 'GAUSS_EXEDIR=/usr/local/gaus/g16/bsd:/usr/lo-
cal/gaus/g16' 'MALLOC_CHECK_=0' 'SIESTA_PP_PATH=.' 'CRY2K9_EXEDIR=/home/vlasta/crystal/bin/Linux-ifort' 'EX-
CITINGASE=/home/vlasta/md/run/excitingscripts/ase' 'ASE_ABINIT_COMMAND=mpirun -np 2 abinit < PREFIX.files >
PREFIX.log' 'LC_ADDRESS=hr_HR.UTF-8' 'LC_NUMERIC=hr_HR.UTF-8' 'DACAPOEXE_PARALLEL=/usr/bin/dacapo_mpi.run'
'FLEUR_INPGEN=/home/vlasta/bin/inpgen.x' 'SSH_TTY=/dev/pts/0' 'MAIL=/var/mail/vlasta' 'PAPERSIZE=a4'
'ELK_SPECIES_PATH=/usr/local/share/elk-dft/species/' 'TERM=xterm' 'SHELL=/bin/bash'
'CRY2K9_ROOT=/home/vlasta/crystal' 'CRY2K9_ARCH=Linux-ifort' 'CRY2K9_BIN=bin' 'G16BASIS=/usr/lo-
cal/gaus/g16/basis' 'FLEUR=/home/vlasta/bin/fleur.x' 'LAMMPS_COMMAND=/usr/bin/lammps' 'SHLVL=2' 'LAN-
GUAGE=en_US:en' 'PYTHONPATH=/usr/local/gaus/gauopen:/usr/local/gaus/g16/bsd:/usr/local/gaus/g16' 'LC_TELE-
PHONE=hr_HR.UTF-8' 'LOGNAME=vlasta' 'DBUS_SESSION_BUS_ADDRESS=unix:path=/run/user/1003/bus'
'XDG_RUNTIME_DIR=/run/user/1003' 'EXCITINGSHARE=/usr/share/exciting' 'HOTBIT_DIR=/usr/share/doc/hotbit/exam-
ples' 'PATH=/home/vlasta/crystal/tmp/tmp1870::/usr/local/share/XCrySDen/:/usr/local/sbin:/usr/lo-
cal/bin:/usr/sbin:/usr/bin:/sbin:/bin:/usr/games:/usr/local/games:./:/home/vlasta/crystal/bin/Linux-
ifort:/home/vlasta/crystal/utils09:/home/vlasta/crystal/crgra2006:/home/vlasta/md/run/exciting-
scripts:/home/vlasta/md/run/excitingscripts/ATAT@exciting:/usr/local/gaus/gauopen:/usr/lo-
cal/gaus/g16/bsd:/usr/local/gaus/g16:/home/tmpacc/QuantumWise/atk/vnl/bin:/home/tmpacc/QuantumWise/atk/atkpy-
thon/bin:/home/tmpacc/QuantumWise/atk/license:/usr/local/share/XCrySDen//scripts:/usr/local/share/XCryS-
Den//util' 'LC_IDENTIFICATION=hr_HR.UTF-8' 'PERLLIB=/usr/local/gaus/gauopen:/usr/local/gaus/g16/bsd:/usr/lo-
cal/gaus/g16' 'EXCITINGSCRIPTS=/home/vlasta/md/run/excitingscripts' 'PGI_TERM=trace,abort' 'XCRYS-
DEN_SCRATCH=/tmp' 'XLIB_SKIP_ARGB_VISUALS=1' 'ATATROOT=/home/vlasta/md/run/excitingscripts/atat' 'EXCIT-
INGBIN=/usr/bin' 'LESSOPEN=| /usr/bin/lesspipe %s' 'OMP_NUM_THREADS=2' 'LC_TIME=hr_HR.UTF-8' '_=/usr/bin/mpi-
run.mpich' --global-user-env 0 --global-system-env 1 'GFORTRAN_UNBUFFERED_PRECONNECTED=y' --proxy-core-count
8 --exec --exec-appnum 0 --exec-proc-count 8 --exec-local-env 0 --exec-wdir /home/vlasta/crystal/tmp/tmp1870
--exec-args 1 /home/vlasta/crystal/tmp/tmp1870/Pcrystal

[mpiexec@lmfz640] Launch arguments: /usr/bin/hydra_pmi_proxy --control-port lmfz640:38501 --debug --rmk user
--launcher ssh --demux poll --pgid 0 --retries 10 --usize -2 --proxy-id 0
[proxy:0:0@lmfz640] got pmi command (from 0): init
pmi_version=1 pmi_subversion=1
[proxy:0:0@lmfz640] PMI response: cmd=response_to_init pmi_version=1 pmi_subversion=1 rc=0
[proxy:0:0@lmfz640] got pmi command (from 0): get_maxes

[proxy:0:0@lmfz640] PMI response: cmd=maxes kvsname_max=256 keylen_max=64 vallen_max=1024
[proxy:0:0@lmfz640] got pmi command (from 6): init
pmi_version=1 pmi_subversion=1
[proxy:0:0@lmfz640] PMI response: cmd=response_to_init pmi_version=1 pmi_subversion=1 rc=0
[proxy:0:0@lmfz640] got pmi command (from 8): init
pmi_version=1 pmi_subversion=1
[proxy:0:0@lmfz640] PMI response: cmd=response_to_init pmi_version=1 pmi_subversion=1 rc=0
[proxy:0:0@lmfz640] got pmi command (from 13): init
pmi_version=1 pmi_subversion=1
[proxy:0:0@lmfz640] PMI response: cmd=response_to_init pmi_version=1 pmi_subversion=1 rc=0
[proxy:0:0@lmfz640] got pmi command (from 17): init
pmi_version=1 pmi_subversion=1
[proxy:0:0@lmfz640] PMI response: cmd=response_to_init pmi_version=1 pmi_subversion=1 rc=0
[proxy:0:0@lmfz640] got pmi command (from 21): init
pmi_version=1 pmi_subversion=1
[proxy:0:0@lmfz640] PMI response: cmd=response_to_init pmi_version=1 pmi_subversion=1 rc=0
[proxy:0:0@lmfz640] got pmi command (from 0): get_appnum

[proxy:0:0@lmfz640] PMI response: cmd=appnum appnum=0
[proxy:0:0@lmfz640] got pmi command (from 6): get_maxes

[proxy:0:0@lmfz640] PMI response: cmd=maxes kvsname_max=256 keylen_max=64 vallen_max=1024
[proxy:0:0@lmfz640] got pmi command (from 8): get_maxes

[proxy:0:0@lmfz640] PMI response: cmd=maxes kvsname_max=256 keylen_max=64 vallen_max=1024
[proxy:0:0@lmfz640] got pmi command (from 13): get_maxes

[proxy:0:0@lmfz640] PMI response: cmd=maxes kvsname_max=256 keylen_max=64 vallen_max=1024
[proxy:0:0@lmfz640] got pmi command (from 17): get_maxes

[proxy:0:0@lmfz640] PMI response: cmd=maxes kvsname_max=256 keylen_max=64 vallen_max=1024
[proxy:0:0@lmfz640] got pmi command (from 25): init
pmi_version=1 pmi_subversion=1
```

```
[proxy:0:0@lmfz640] PMI response: cmd=response_to_init pmi_version=1 pmi_subversion=1 rc=0
[proxy:0:0@lmfz640] got pmi command (from 29): init
pmi_version=1 pmi_subversion=1
[proxy:0:0@lmfz640] PMI response: cmd=response_to_init pmi_version=1 pmi_subversion=1 rc=0
[proxy:0:0@lmfz640] got pmi command (from 0): get_my_kvsname

[proxy:0:0@lmfz640] PMI response: cmd=my_kvsname kvsname=kvs_1911_0
[proxy:0:0@lmfz640] got pmi command (from 6): get_appnum

[proxy:0:0@lmfz640] PMI response: cmd=appnum appnum=0
[proxy:0:0@lmfz640] got pmi command (from 8): get_appnum

[proxy:0:0@lmfz640] PMI response: cmd=appnum appnum=0
[proxy:0:0@lmfz640] got pmi command (from 13): get_appnum

[proxy:0:0@lmfz640] PMI response: cmd=appnum appnum=0
[proxy:0:0@lmfz640] got pmi command (from 17): get_appnum

[proxy:0:0@lmfz640] PMI response: cmd=appnum appnum=0
[proxy:0:0@lmfz640] got pmi command (from 21): get_maxes

[proxy:0:0@lmfz640] PMI response: cmd=maxes kvsname_max=256 keylen_max=64 vallen_max=1024
[proxy:0:0@lmfz640] got pmi command (from 25): get_maxes

[proxy:0:0@lmfz640] PMI response: cmd=maxes kvsname_max=256 keylen_max=64 vallen_max=1024
[proxy:0:0@lmfz640] got pmi command (from 0): get_my_kvsname

[proxy:0:0@lmfz640] PMI response: cmd=my_kvsname kvsname=kvs_1911_0
[proxy:0:0@lmfz640] got pmi command (from 6): get_my_kvsname

[proxy:0:0@lmfz640] PMI response: cmd=my_kvsname kvsname=kvs_1911_0
[proxy:0:0@lmfz640] got pmi command (from 8): get_my_kvsname

[proxy:0:0@lmfz640] PMI response: cmd=my_kvsname kvsname=kvs_1911_0
[proxy:0:0@lmfz640] got pmi command (from 13): get_my_kvsname

[proxy:0:0@lmfz640] PMI response: cmd=my_kvsname kvsname=kvs_1911_0
[proxy:0:0@lmfz640] got pmi command (from 17): get_my_kvsname

[proxy:0:0@lmfz640] PMI response: cmd=my_kvsname kvsname=kvs_1911_0
[proxy:0:0@lmfz640] got pmi command (from 21): get_appnum

[proxy:0:0@lmfz640] PMI response: cmd=appnum appnum=0
[proxy:0:0@lmfz640] got pmi command (from 29): get_maxes

[proxy:0:0@lmfz640] PMI response: cmd=maxes kvsname_max=256 keylen_max=64 vallen_max=1024
[proxy:0:0@lmfz640] got pmi command (from 0): get
kvsname=kvs_1911_0 key=PMI_process_mapping
[proxy:0:0@lmfz640] PMI response: cmd=get_result rc=0 msg=success value=(vector,(0,1,8))
[proxy:0:0@lmfz640] got pmi command (from 6): get_my_kvsname

[proxy:0:0@lmfz640] PMI response: cmd=my_kvsname kvsname=kvs_1911_0
[proxy:0:0@lmfz640] got pmi command (from 8): get_my_kvsname

[proxy:0:0@lmfz640] PMI response: cmd=my_kvsname kvsname=kvs_1911_0
[proxy:0:0@lmfz640] got pmi command (from 13): get_my_kvsname

[proxy:0:0@lmfz640] PMI response: cmd=my_kvsname kvsname=kvs_1911_0
[proxy:0:0@lmfz640] got pmi command (from 17): get_my_kvsname

[proxy:0:0@lmfz640] PMI response: cmd=my_kvsname kvsname=kvs_1911_0
[proxy:0:0@lmfz640] got pmi command (from 21): get_my_kvsname

[proxy:0:0@lmfz640] PMI response: cmd=my_kvsname kvsname=kvs_1911_0
[proxy:0:0@lmfz640] got pmi command (from 25): get_appnum

[proxy:0:0@lmfz640] PMI response: cmd=appnum appnum=0
[proxy:0:0@lmfz640] got pmi command (from 0): put
kvsname=kvs_1911_0 key=sharedFilename[0] value=/dev/shm/mpich_shar_tmpnd8GMQ
[proxy:0:0@lmfz640] cached command: sharedFilename[0]=/dev/shm/mpich_shar_tmpnd8GMQ
[proxy:0:0@lmfz640] PMI response: cmd=put_result rc=0 msg=success
[proxy:0:0@lmfz640] got pmi command (from 6): get
kvsname=kvs_1911_0 key=PMI_process_mapping
[proxy:0:0@lmfz640] PMI response: cmd=get_result rc=0 msg=success value=(vector,(0,1,8))
[proxy:0:0@lmfz640] got pmi command (from 8): get
kvsname=kvs_1911_0 key=PMI_process_mapping
[proxy:0:0@lmfz640] PMI response: cmd=get_result rc=0 msg=success value=(vector,(0,1,8))
[proxy:0:0@lmfz640] got pmi command (from 13): get
kvsname=kvs_1911_0 key=PMI_process_mapping
```

```
[proxy:0:0@lmfz640] PMI response: cmd=get_result rc=0 msg=success value=(vector,(0,1,8))
[proxy:0:0@lmfz640] got pmi command (from 17): get
kvsname=kvs_1911_0 key=PMI_process_mapping
[proxy:0:0@lmfz640] PMI response: cmd=get_result rc=0 msg=success value=(vector,(0,1,8))
[proxy:0:0@lmfz640] got pmi command (from 21): get_my_kvsname

[proxy:0:0@lmfz640] PMI response: cmd=my_kvsname kvsname=kvs_1911_0
[proxy:0:0@lmfz640] got pmi command (from 29): get_appnum

[proxy:0:0@lmfz640] PMI response: cmd=appnum appnum=0
[proxy:0:0@lmfz640] got pmi command (from 0): barrier_in

[proxy:0:0@lmfz640] got pmi command (from 6): barrier_in

[proxy:0:0@lmfz640] got pmi command (from 8): barrier_in

[proxy:0:0@lmfz640] got pmi command (from 13): barrier_in

[proxy:0:0@lmfz640] got pmi command (from 17): barrier_in

[proxy:0:0@lmfz640] got pmi command (from 21): get
kvsname=kvs_1911_0 key=PMI_process_mapping
[proxy:0:0@lmfz640] PMI response: cmd=get_result rc=0 msg=success value=(vector,(0,1,8))
[proxy:0:0@lmfz640] got pmi command (from 25): get_my_kvsname

[proxy:0:0@lmfz640] PMI response: cmd=my_kvsname kvsname=kvs_1911_0
[proxy:0:0@lmfz640] got pmi command (from 21): barrier_in

[proxy:0:0@lmfz640] got pmi command (from 29): get_my_kvsname

[proxy:0:0@lmfz640] PMI response: cmd=my_kvsname kvsname=kvs_1911_0
[proxy:0:0@lmfz640] got pmi command (from 25): get_my_kvsname

[proxy:0:0@lmfz640] PMI response: cmd=my_kvsname kvsname=kvs_1911_0
[proxy:0:0@lmfz640] got pmi command (from 29): get_my_kvsname

[proxy:0:0@lmfz640] PMI response: cmd=my_kvsname kvsname=kvs_1911_0
[proxy:0:0@lmfz640] got pmi command (from 25): get
kvsname=kvs_1911_0 key=PMI_process_mapping
[proxy:0:0@lmfz640] PMI response: cmd=get_result rc=0 msg=success value=(vector,(0,1,8))
[proxy:0:0@lmfz640] got pmi command (from 29): get
kvsname=kvs_1911_0 key=PMI_process_mapping
[proxy:0:0@lmfz640] PMI response: cmd=get_result rc=0 msg=success value=(vector,(0,1,8))
[proxy:0:0@lmfz640] got pmi command (from 25): barrier_in

[proxy:0:0@lmfz640] got pmi command (from 29): barrier_in

[proxy:0:0@lmfz640] flushing 1 put command(s) out
[proxy:0:0@lmfz640] forwarding command (cmd=put sharedFilename[0]=/dev/shm/mpich_shar_tmpnd8GMQ) upstream
[mpiexec@lmfz640] [pgid: 0] got PMI command: cmd=put sharedFilename[0]=/dev/shm/mpich_shar_tmpnd8GMQ
[proxy:0:0@lmfz640] forwarding command (cmd=barrier_in) upstream
[mpiexec@lmfz640] [pgid: 0] got PMI command: cmd=barrier_in
[mpiexec@lmfz640] PMI response to fd 6 pid 29: cmd=keyval_cache sharedFile-
name[0]=/dev/shm/mpich_shar_tmpnd8GMQ
[mpiexec@lmfz640] PMI response to fd 6 pid 29: cmd=barrier_out
[proxy:0:0@lmfz640] PMI response: cmd=barrier_out
[proxy:0:0@lmfz640] PMI response: cmd=barrier_out
[proxy:0:0@lmfz640] PMI response: cmd=barrier_out
[proxy:0:0@lmfz640] PMI response: cmd=barrier_out
[proxy:0:0@lmfz640] PMI response: cmd=barrier_out
[proxy:0:0@lmfz640] PMI response: cmd=barrier_out
[proxy:0:0@lmfz640] PMI response: cmd=barrier_out
[proxy:0:0@lmfz640] PMI response: cmd=barrier_out
[proxy:0:0@lmfz640] got pmi command (from 6): get
kvsname=kvs_1911_0 key=sharedFilename[0]
[proxy:0:0@lmfz640] PMI response: cmd=get_result rc=0 msg=success value=/dev/shm/mpich_shar_tmpnd8GMQ
[proxy:0:0@lmfz640] got pmi command (from 8): get
kvsname=kvs_1911_0 key=sharedFilename[0]
[proxy:0:0@lmfz640] PMI response: cmd=get_result rc=0 msg=success value=/dev/shm/mpich_shar_tmpnd8GMQ
[proxy:0:0@lmfz640] got pmi command (from 13): get
kvsname=kvs_1911_0 key=sharedFilename[0]
[proxy:0:0@lmfz640] PMI response: cmd=get_result rc=0 msg=success value=/dev/shm/mpich_shar_tmpnd8GMQ
[proxy:0:0@lmfz640] got pmi command (from 17): get
kvsname=kvs_1911_0 key=sharedFilename[0]
[proxy:0:0@lmfz640] PMI response: cmd=get_result rc=0 msg=success value=/dev/shm/mpich_shar_tmpnd8GMQ
[proxy:0:0@lmfz640] got pmi command (from 21): get
kvsname=kvs_1911_0 key=sharedFilename[0]
[proxy:0:0@lmfz640] PMI response: cmd=get_result rc=0 msg=success value=/dev/shm/mpich_shar_tmpnd8GMQ
[proxy:0:0@lmfz640] got pmi command (from 25): get
```

```
kvsname=kvs_1911_0 key=sharedFilename[0]
[proxy:0:0@lmfz640] PMI response: cmd=get_result rc=0 msg=success value=/dev/shm/mpich_shar_tmpnd8GMQ
[proxy:0:0@lmfz640] got pmi command (from 29): get
kvsname=kvs_1911_0 key=sharedFilename[0]
[proxy:0:0@lmfz640] PMI response: cmd=get_result rc=0 msg=success value=/dev/shm/mpich_shar_tmpnd8GMQ
[proxy:0:0@lmfz640] got pmi command (from 0): put
kvsname=kvs_1911_0 key=P0-businesscard value=description#lmfz640$port#52497$ifname#10.135.24.38$
[proxy:0:0@lmfz640] cached command: P0-businesscard=description#lmfz640$port#52497$ifname#10.135.24.38$
[proxy:0:0@lmfz640] PMI response: cmd=put_result rc=0 msg=success
[proxy:0:0@lmfz640] got pmi command (from 6): put
kvsname=kvs_1911_0 key=P1-businesscard value=description#lmfz640$port#59195$ifname#10.135.24.38$
[proxy:0:0@lmfz640] cached command: P1-businesscard=description#lmfz640$port#59195$ifname#10.135.24.38$
[proxy:0:0@lmfz640] PMI response: cmd=put_result rc=0 msg=success
[proxy:0:0@lmfz640] got pmi command (from 8): put
kvsname=kvs_1911_0 key=P2-businesscard value=description#lmfz640$port#40279$ifname#10.135.24.38$
[proxy:0:0@lmfz640] cached command: P2-businesscard=description#lmfz640$port#40279$ifname#10.135.24.38$
[proxy:0:0@lmfz640] PMI response: cmd=put_result rc=0 msg=success
[proxy:0:0@lmfz640] got pmi command (from 13): put
kvsname=kvs_1911_0 key=P3-businesscard value=description#lmfz640$port#33639$ifname#10.135.24.38$
[proxy:0:0@lmfz640] cached command: P3-businesscard=description#lmfz640$port#33639$ifname#10.135.24.38$
[proxy:0:0@lmfz640] PMI response: cmd=put_result rc=0 msg=success
[proxy:0:0@lmfz640] got pmi command (from 17): put
kvsname=kvs_1911_0 key=P4-businesscard value=description#lmfz640$port#40269$ifname#10.135.24.38$
[proxy:0:0@lmfz640] cached command: P4-businesscard=description#lmfz640$port#40269$ifname#10.135.24.38$
[proxy:0:0@lmfz640] PMI response: cmd=put_result rc=0 msg=success
[proxy:0:0@lmfz640] got pmi command (from 25): put
kvsname=kvs_1911_0 key=P6-businesscard value=description#lmfz640$port#39259$ifname#10.135.24.38$
[proxy:0:0@lmfz640] cached command: P6-businesscard=description#lmfz640$port#39259$ifname#10.135.24.38$
[proxy:0:0@lmfz640] PMI response: cmd=put_result rc=0 msg=success
[proxy:0:0@lmfz640] got pmi command (from 29): put
kvsname=kvs_1911_0 key=P7-businesscard value=description#lmfz640$port#36641$ifname#10.135.24.38$
[proxy:0:0@lmfz640] cached command: P7-businesscard=description#lmfz640$port#36641$ifname#10.135.24.38$
[proxy:0:0@lmfz640] PMI response: cmd=put_result rc=0 msg=success
[proxy:0:0@lmfz640] got pmi command (from 0): barrier_in

[proxy:0:0@lmfz640] got pmi command (from 6): barrier_in

[proxy:0:0@lmfz640] got pmi command (from 8): barrier_in

[proxy:0:0@lmfz640] got pmi command (from 13): barrier_in

[proxy:0:0@lmfz640] got pmi command (from 17): barrier_in

[proxy:0:0@lmfz640] got pmi command (from 21): put
kvsname=kvs_1911_0 key=P5-businesscard value=description#lmfz640$port#53233$ifname#10.135.24.38$
[proxy:0:0@lmfz640] cached command: P5-businesscard=description#lmfz640$port#53233$ifname#10.135.24.38$
[proxy:0:0@lmfz640] PMI response: cmd=put_result rc=0 msg=success
[proxy:0:0@lmfz640] got pmi command (from 25): barrier_in

[proxy:0:0@lmfz640] got pmi command (from 21): barrier_in

[proxy:0:0@lmfz640] got pmi command (from 29): barrier_in

[proxy:0:0@lmfz640] flushing 8 put command(s) out
[proxy:0:0@lmfz640] forwarding command (cmd=put P0-businesscard=description#lmfz640$port#52497$if-
name#10.135.24.38$ P1-businesscard=description#lmfz640$port#59195$ifname#10.135.24.38$ P2-businesscard=de-
scription#lmfz640$port#40279$ifname#10.135.24.38$ P3-businesscard=description#lmfz640$port#33639$if-
name#10.135.24.38$ P4-businesscard=description#lmfz640$port#40269$ifname#10.135.24.38$ P6-businesscard=de-
scription#lmfz640$port#39259$ifname#10.135.24.38$ P7-businesscard=description#lmfz640$port#36641$if-
name#10.135.24.38$ P5-businesscard=description#lmfz640$port#53233$ifname#10.135.24.38$) upstream
[mpiexec@lmfz640] [pgid: 0] got PMI command: cmd=put P0-businesscard=description#lmfz640$port#52497$if-
name#10.135.24.38$ P1-businesscard=description#lmfz640$port#59195$ifname#10.135.24.38$ P2-businesscard=de-
scription#lmfz640$port#40279$ifname#10.135.24.38$ P3-businesscard=description#lmfz640$port#33639$if-
name#10.135.24.38$ P4-businesscard=description#lmfz640$port#40269$ifname#10.135.24.38$ P6-businesscard=de-
scription#lmfz640$port#39259$ifname#10.135.24.38$ P7-businesscard=description#lmfz640$port#36641$if-
name#10.135.24.38$ P5-businesscard=description#lmfz640$port#53233$ifname#10.135.24.38$
[proxy:0:0@lmfz640] forwarding command (cmd=barrier_in) upstream
[mpiexec@lmfz640] [pgid: 0] got PMI command: cmd=barrier_in
[mpiexec@lmfz640] PMI response to fd 6 pid 29: cmd=keyval_cache P0-businesscard=descrip-
tion#lmfz640$port#52497$ifname#10.135.24.38$ P1-businesscard=description#lmfz640$port#59195$if-
name#10.135.24.38$ P2-businesscard=description#lmfz640$port#40279$ifname#10.135.24.38$ P3-businesscard=de-
scription#lmfz640$port#33639$ifname#10.135.24.38$ P4-businesscard=description#lmfz640$port#40269$if-
name#10.135.24.38$ P6-businesscard=description#lmfz640$port#39259$ifname#10.135.24.38$ P7-businesscard=de-
scription#lmfz640$port#36641$ifname#10.135.24.38$ P5-businesscard=description#lmfz640$port#53233$if-
name#10.135.24.38$
[mpiexec@lmfz640] PMI response to fd 6 pid 29: cmd=barrier_out
[proxy:0:0@lmfz640] PMI response: cmd=barrier_out
[proxy:0:0@lmfz640] PMI response: cmd=barrier_out
[proxy:0:0@lmfz640] PMI response: cmd=barrier_out
```

```
[proxy:0:0@lmfz640] PMI response: cmd=barrier_out
[proxy:0:0@lmfz640] PMI response: cmd=barrier_out
[proxy:0:0@lmfz640] PMI response: cmd=barrier_out
[proxy:0:0@lmfz640] PMI response: cmd=barrier_out
[proxy:0:0@lmfz640] PMI response: cmd=barrier_out
 PROCESS            0  OF           8  WORKING
 PROCESS            6  OF           8  WORKING
 PROCESS            3  OF           8  WORKING
 PROCESS            5  OF           8  WORKING
 PROCESS            1  OF           8  WORKING
 PROCESS            2  OF           8  WORKING
 PROCESS            4  OF           8  WORKING
 PROCESS            7  OF           8  WORKING


 ******************************************************************************
 *                                                                            *
 *                                CRYSTAL09                                   *
 *                              public : 2.0                                  *
 *                      December 10th, 2012 - parallel executable             *
 *                                                                            *
 *                                                                            *
 *                                                                            *
 *                              MAIN AUTHORS                                  *
 *                                                                            *
 *    R. DOVESI(1,10), V.R. SAUNDERS(2), C. ROETTI(1,10), R. ORLANDO (1,3),    *
 *  C.M. ZICOVICH-WILSON(1,4), F. PASCALE(5), B. CIVALLERI(1,10), K. DOLL(6),  *
 *       N.M. HARRISON(2,7), I. J. BUSH(2), Ph. D'ARCO(8), M. LLUNELL(9)       *
 *                                                                            *
 * (1) THEORETICAL CHEMISTRY GROUP - UNIVERSITA' DI TORINO - TORINO (ITALY)    *
 *      http://www.crystal.unito.it                                           *
 * (2) COMPUTATIONAL SCIENCE & ENGINEERING DEPARTMENT - STFC DARESBURY LABORATO*Y (UK)
 *     http://www.cse.stfc.ac.uk/cmg/CRYSTAL/                                 *
 * (3) UNIVERSITA' DEL PIEMONTE ORIENTALE - ALESSANDRIA (ITALY)               *
 * (4) UNIVERSIDAD AUTONOMA DEL ESTADO DE MORELOS - CUERNAVACA (MEXICO)       *
 * (5) UNIVERSITE' HENRI POINCARE' - NANCY (FRANCE)                           *
 * (6) MPI FUER FESTKOERPERFORSCHUNG - STUTTGART (GERMANY)                    *
 * (7) IMPERIAL COLLEGE - LONDON (UK)                                         *
 * (8) UNIVERSITE' PIERRE ET MARIE CURIE - PARIS (FRANCE)                     *
 * (9) UNIVERSIDAD DE BARCELONA - BARCELONA (SPAIN)                           *
 *(10) NIS - NANOSTRUCTURED INTERFACES AND SURFACES - TORINO (ITALY)          *
 *     http://www.crystal.unito.it                                            *
 ******************************************************************************
 EEEEEEEEEE STARTING  DATE 09 03 2019 TIME 10:48:14.2
 Ca2SiO4 cif file

 CRYSTAL CALCULATION
 (INPUT ACCORDING TO THE INTERNATIONAL TABLES FOR X-RAY CRYSTALLOGRAPHY)
 CRYSTAL FAMILY                       :  MONOCLINIC
 CRYSTAL CLASS  (GROTH - 1921)        :  MONOCLINIC PRISMATIC

 SPACE GROUP (CENTROSYMMETRIC)        :  P 1 21/N 1

 LATTICE PARAMETERS  (ANGSTROMS AND DEGREES) - CONVENTIONAL CELL
        A           B           C        ALPHA        BETA       GAMMA
    5.50750     6.75090     9.30550    90.00000    94.59700    90.00000


 NUMBER OF IRREDUCIBLE ATOMS IN THE CONVENTIONAL CELL:    7

 INPUT COORDINATES

 ATOM AT. N.              COORDINATES
   1  20     2.259743027399E-01  3.440807844666E-01  4.324013106182E-01
   2  20    -2.789152102887E-01 -4.958528969244E-01  2.015579430480E-01
   3  14     2.679963311619E-01 -2.171331715239E-01  4.178753611653E-01
   4   8     2.175666700724E-01  1.543947662208E-02  4.443157037911E-01
   5   8     1.015604961355E-02 -3.312309356329E-01  3.638776329533E-01
   6   8     4.773877212424E-01 -2.478022947534E-01  3.033977422278E-01
   7   8     3.504877954991E-01 -3.317228088380E-01 -4.281642024754E-01

 ******************************************************************************

 << INFORMATION >>: FROM NOW ON, ALL COORDINATES REFER TO THE PRIMITIVE CELL

 ******************************************************************************

 LATTICE PARAMETERS  (ANGSTROMS AND DEGREES) - PRIMITIVE CELL
       A          B          C         ALPHA     BETA     GAMMA        VOLUME
```

```
   5.50750    6.75090    9.30550    90.00000  94.59700  90.00000    344.870900

COORDINATES OF THE EQUIVALENT ATOMS (FRACTIONAL UNITS)

N. ATOM EQUIV AT. N.          X                  Y                  Z

  1   1   1   20 CA    2.25974302740E-01  3.44080784467E-01  4.32401310618E-01
  2   1   2   20 CA    2.74025697260E-01 -1.55919215533E-01  6.75986893818E-02
  3   1   3   20 CA   -2.25974302740E-01 -3.44080784467E-01 -4.32401310618E-01
  4   1   4   20 CA   -2.74025697260E-01  1.55919215533E-01 -6.75986893818E-02

  5   2   1   20 CA   -2.78915210289E-01 -4.95852896924E-01  2.01557943048E-01
  6   2   2   20 CA   -2.21084789711E-01  4.14710307560E-03  2.98442056952E-01
  7   2   3   20 CA    2.78915210289E-01  4.95852896924E-01 -2.01557943048E-01
  8   2   4   20 CA    2.21084789711E-01 -4.14710307560E-03 -2.98442056952E-01

  9   3   1   14 SI    2.67996331162E-01 -2.17133171524E-01  4.17875361165E-01
 10   3   2   14 SI    2.32003668838E-01  2.82866828476E-01  8.21246388347E-02
 11   3   3   14 SI   -2.67996331162E-01  2.17133171524E-01 -4.17875361165E-01
 12   3   4   14 SI   -2.32003668838E-01 -2.82866828476E-01 -8.21246388347E-02

 13   4   1    8 O     2.17566670072E-01  1.54394766221E-02  4.44315703791E-01
 14   4   2    8 O     2.82433329928E-01 -4.84560523378E-01  5.56842962089E-02
 15   4   3    8 O    -2.17566670072E-01 -1.54394766221E-02 -4.44315703791E-01
 16   4   4    8 O    -2.82433329928E-01  4.84560523378E-01 -5.56842962089E-02

 17   5   1    8 O     1.01560496135E-02 -3.31230935633E-01  3.63877632953E-01
 18   5   2    8 O     4.89843950386E-01  1.68769064367E-01  1.36122367047E-01
 19   5   3    8 O    -1.01560496135E-02  3.31230935633E-01 -3.63877632953E-01
 20   5   4    8 O    -4.89843950386E-01 -1.68769064367E-01 -1.36122367047E-01

 21   6   1    8 O     4.77387721242E-01 -2.47802294753E-01  3.03397742228E-01
 22   6   2    8 O     2.26122787576E-02  2.52197705247E-01  1.96602257772E-01
 23   6   3    8 O    -4.77387721242E-01  2.47802294753E-01 -3.03397742228E-01
 24   6   4    8 O    -2.26122787576E-02 -2.52197705247E-01 -1.96602257772E-01

 25   7   1    8 O     3.50487795499E-01 -3.31722808838E-01 -4.28164202475E-01
 26   7   2    8 O     1.49512204501E-01  1.68277191162E-01 -7.18357975246E-02
 27   7   3    8 O    -3.50487795499E-01  3.31722808838E-01  4.28164202475E-01
 28   7   4    8 O    -1.49512204501E-01 -1.68277191162E-01  7.18357975246E-02

NUMBER OF SYMMETRY OPERATORS         :    4
*******************************************************************************
* GEOMETRY EDITING - INPUT COORDINATES ARE GIVEN IN ANGSTROM
*******************************************************************************

GEOMETRY NOW FULLY CONSISTENT WITH THE GROUP

FRQFRQFRQFRQFRQFRQFRQFRQFRQFRQFRQFRQFRQFRQFRQFRQFRQFRQFRQFRQFRQFRQFRQFRQFRQFRQ

                      FREQUENCY CALCULATION


GCALCO - MAX INDICES DIRECT LATTICE VECTOR    19    15    11
NO.OF VECTORS CREATED 6999 STARS 1862 RMAX   157.41387 BOHR

GEOMETRY FOR WAVE FUNCTION - DIMENSIONALITY OF THE SYSTEM    3
(NON PERIODIC DIRECTION: LATTICE PARAMETER FORMALLY SET TO 500)
*******************************************************************************
LATTICE PARAMETERS (ANGSTROMS AND DEGREES) - BOHR = 0.5291772083 ANGSTROM
PRIMITIVE CELL - CENTRING CODE 1/0 VOLUME=   344.870900 - DENSITY  3.310 g/cm^3
         A              B              C           ALPHA      BETA       GAMMA
    5.50750000     6.75090000     9.30550000    90.000000  94.597000  90.000000
*******************************************************************************
ATOMS IN THE ASYMMETRIC UNIT    7 - ATOMS IN THE UNIT CELL:   28
    ATOM              X/A                 Y/B                 Z/C
*******************************************************************************
  1 T  20 CA    2.259743027399E-01  3.440807844666E-01  4.324013106182E-01
  2 F  20 CA    2.740256972601E-01 -1.559192155334E-01  6.759868938180E-02
  3 F  20 CA   -2.259743027399E-01 -3.440807844666E-01 -4.324013106182E-01
  4 F  20 CA   -2.740256972601E-01  1.559192155334E-01 -6.759868938180E-02
  5 T  20 CA   -2.789152102887E-01 -4.958528969244E-01  2.015579430480E-01
  6 F  20 CA   -2.210847897113E-01  4.147103075600E-03  2.984420569520E-01
  7 F  20 CA    2.789152102887E-01  4.958528969244E-01 -2.015579430480E-01
  8 F  20 CA    2.210847897113E-01 -4.147103075600E-03 -2.984420569520E-01
  9 T  14 SI    2.679963311619E-01 -2.171331715239E-01  4.178753611653E-01
 10 F  14 SI    2.320036688381E-01  2.828668284761E-01  8.212463883470E-02
 11 F  14 SI   -2.679963311619E-01  2.171331715239E-01 -4.178753611653E-01
 12 F  14 SI   -2.320036688381E-01 -2.828668284761E-01 -8.212463883470E-02
```

```
 13 T   8 O     2.175666700724E-01  1.543947662208E-02  4.443157037911E-01
 14 F   8 O     2.824333299276E-01 -4.845605233779E-01  5.568429620890E-02
 15 F   8 O    -2.175666700724E-01 -1.543947662208E-02 -4.443157037911E-01
 16 F   8 O    -2.824333299276E-01  4.845605233779E-01 -5.568429620890E-02
 17 T   8 O     1.015604961355E-02 -3.312309356329E-01  3.638776329533E-01
 18 F   8 O     4.898439503864E-01  1.687690643671E-01  1.361223670467E-01
 19 F   8 O    -1.015604961355E-02  3.312309356329E-01 -3.638776329533E-01
 20 F   8 O    -4.898439503865E-01 -1.687690643671E-01 -1.361223670467E-01
 21 T   8 O     4.773877212424E-01 -2.478022947534E-01  3.033977422278E-01
 22 F   8 O     2.261227875760E-02  2.521977052466E-01  1.966022577722E-01
 23 F   8 O    -4.773877212424E-01  2.478022947534E-01 -3.033977422278E-01
 24 F   8 O    -2.261227875760E-02 -2.521977052466E-01 -1.966022577722E-01
 25 T   8 O     3.504877954991E-01 -3.317228088380E-01 -4.281642024754E-01
 26 F   8 O     1.495122045009E-01  1.682771911620E-01 -7.183579752460E-02
 27 F   8 O    -3.504877954991E-01  3.317228088380E-01  4.281642024754E-01
 28 F   8 O    -1.495122045009E-01 -1.682771911620E-01  7.183579752460E-02

T = ATOM BELONGING TO THE ASYMMETRIC UNIT

****   4 SYMMOPS - TRANSLATORS IN FRACTIONAL UNITS
 V INV                    ROTATION MATRICES                          TRANSLATOR
 1  1  1.00  0.00  0.00  0.00  1.00  0.00  0.00  0.00  1.00    0.00  0.00  0.00
 2  2 -1.00  0.00  0.00 -0.00  1.00  0.00  0.00  0.00 -1.00    0.50  0.50  0.50
 3  3 -1.00  0.00  0.00  0.00 -1.00  0.00  0.00  0.00 -1.00    0.00  0.00  0.00
 4  4  1.00  0.00 -0.00  0.00 -1.00 -0.00  0.00  0.00  1.00    0.50  0.50  0.50

DIRECT LATTICE VECTORS CARTESIAN COMPONENTS (ANGSTROM)
         X                    Y                    Z
  0.550750000000E+01   0.337237112315E-15   0.000000000000E+00
  0.000000000000E+00   0.675090000000E+01   0.000000000000E+00
 -0.745805423921E+00   0.569797539473E-15   0.927556491647E+01


CARTESIAN COORDINATES - PRIMITIVE CELL
*******************************************************************************
*      ATOM          X(ANGSTROM)         Y(ANGSTROM)         Z(ANGSTROM)
*******************************************************************************
  1    20 CA    9.220662295702E-01  2.322854967856E+00  4.010766426606E+00
  2    20 CA    1.458781058469E+00 -1.052595032144E+00  6.270160316292E-01
  3    20 CA   -9.220662295702E-01 -2.322854967856E+00 -4.010766426606E+00
  4    20 CA   -1.458781058469E+00  1.052595032144E+00 -6.270160316292E-01
  5    20 CA   -1.686448527825E+00 -3.347453321847E+00  1.869563785172E+00
  6    20 CA   -1.440204184136E+00  2.799667815307E-02  2.768218673063E+00
  7    20 CA    1.686448527825E+00  3.347453321847E+00 -1.869563785172E+00
  8    20 CA    1.440204184136E+00 -2.799667815307E-02 -2.768218673063E+00
  9    14 SI    1.164336082994E+00 -1.465844327641E+00  3.876030039482E+00
 10    14 SI    1.216511205045E+00  1.909605672359E+00  7.617524187529E-01
 11    14 SI   -1.164336082994E+00  1.465844327641E+00 -3.876030039482E+00
 12    14 SI   -1.216511205045E+00 -1.909605672359E+00 -7.617524187529E-01
 13     8 O     8.668753736029E-01  1.042303627280E-01  4.121279153921E+00
 14     8 O     1.513971914436E+00 -3.271219637272E+00  5.165033043136E-01
 15     8 O    -8.668753736029E-01 -1.042303627280E-01 -4.121279153921E+00
 16     8 O    -1.513971914436E+00  3.271219637272E+00 -5.165033043136E-01
 17     8 O    -2.154474690536E-01 -2.236106923364E+00  3.375170606110E+00
 18     8 O     2.596294757093E+00  1.139343076636E+00  1.262611852125E+00
 19     8 O     2.154474690536E-01  2.236106923364E+00 -3.375170606110E+00
 20     8 O    -2.596294757093E+00 -1.139343076636E+00 -1.262611852125E+00
 21     8 O     2.402937192984E+00 -1.672888511651E+00  2.814185453544E+00
 22     8 O    -2.208990494422E-02  1.702561488349E+00  1.823597004691E+00
 23     8 O    -2.402937192984E+00  1.672888511651E+00 -2.814185453544E+00
 24     8 O     2.208990494422E-02 -1.702561488349E+00 -1.823597004691E+00
 25     8 O     2.249638718246E+00 -2.239427510184E+00 -3.971464854969E+00
 26     8 O     8.770139937143E-01  1.136022489816E+00 -6.663176032658E-01
 27     8 O    -2.249638718246E+00  2.239427510184E+00  3.971464854969E+00
 28     8 O    -8.770139937143E-01 -1.136022489816E+00  6.663176032658E-01

*******************************************************************************
LOCAL ATOMIC FUNCTIONS BASIS SET
*******************************************************************************
   ATOM  X(AU)  Y(AU)  Z(AU)    NO. TYPE  EXPONENT  S COEF   P COEF   D/F/G COEF
*******************************************************************************
   1 CA  1.742  4.390  7.579
                                  1 S
                                         1.913E+05 2.204E-04 0.000E+00 0.000E+00
                                         2.697E+04 1.925E-03 0.000E+00 0.000E+00
                                         5.696E+03 1.109E-02 0.000E+00 0.000E+00
                                         1.489E+03 4.995E-02 0.000E+00 0.000E+00
                                         4.483E+02 1.701E-01 0.000E+00 0.000E+00
                                         1.546E+02 3.685E-01 0.000E+00 0.000E+00
```

```
                                              6.037E+01 4.034E-01 0.000E+00 0.000E+00
                                              2.509E+01 1.452E-01 0.000E+00 0.000E+00
                               2-   5 SP
                                              4.486E+02-5.750E-03 8.470E-03 0.000E+00
                                              1.057E+02-7.670E-02 6.027E-02 0.000E+00
                                              3.469E+01-1.122E-01 2.124E-01 0.000E+00
                                              1.350E+01 2.537E-01 3.771E-01 0.000E+00
                                              5.820E+00 6.880E-01 4.010E-01 0.000E+00
                                              1.819E+00 3.490E-01 1.980E-01 0.000E+00
                               6-   9 SP
                                              2.075E+01-2.000E-03-3.650E-02 0.000E+00
                                              8.400E+00-1.255E-01-6.850E-02 0.000E+00
                                              3.597E+00-6.960E-01 1.570E-01 0.000E+00
                                              1.408E+00 1.029E+00 1.482E+00 0.000E+00
                                              7.260E-01 9.440E-01 1.025E+00 0.000E+00
                              10-  13 SP
                                              4.530E-01 1.000E+00 1.000E+00 0.000E+00
                              14-  17 SP
                                              2.950E-01 1.000E+00 1.000E+00 0.000E+00
                              18-  22 D
                                              3.191E+00 0.000E+00 0.000E+00 1.600E-01
                                              8.683E-01 0.000E+00 0.000E+00 3.130E-01
                              23-  27 D
                                              2.891E-01 0.000E+00 0.000E+00 4.060E-01
  2 CA  2.757 -1.989  1.185
  3 CA -1.742 -4.390 -7.579
  4 CA -2.757  1.989 -1.185
  5 CA -3.187 -6.326  3.533
  6 CA -2.722  0.053  5.231
  7 CA  3.187  6.326 -3.533
  8 CA  2.722 -0.053 -5.231
  9 SI  2.200 -2.770  7.325
                                  217 S
                                              8.765E+04 2.370E-04 0.000E+00 0.000E+00
                                              1.285E+04 1.920E-03 0.000E+00 0.000E+00
                                              2.786E+03 1.090E-02 0.000E+00 0.000E+00
                                              7.280E+02 4.960E-02 0.000E+00 0.000E+00
                                              2.195E+02 1.668E-01 0.000E+00 0.000E+00
                                              7.590E+01 3.630E-01 0.000E+00 0.000E+00
                                              2.946E+01 4.051E-01 0.000E+00 0.000E+00
                                              1.199E+01 1.504E-01 0.000E+00 0.000E+00
                             218- 221 SP
                                              1.660E+02-8.840E-03 9.090E-03 0.000E+00
                                              3.937E+01-8.590E-02 6.010E-02 0.000E+00
                                              1.271E+01-7.120E-02 1.952E-01 0.000E+00
                                              4.777E+00 4.147E-01 3.384E-01 0.000E+00
                                              1.848E+00 6.168E-01 3.006E-01 0.000E+00
                                              7.365E-01 1.154E-01 6.480E-02 0.000E+00
                             222- 225 SP
                                              4.175E+00-1.990E-02-8.700E-03 0.000E+00
                                              1.447E+00-1.864E-01-4.380E-03 0.000E+00
                                              5.023E-01 9.670E-02 2.207E-01 0.000E+00
                             226- 229 SP
                                              3.220E-01 1.000E+00 1.000E+00 0.000E+00
                             230- 233 SP
                                              1.300E-01 1.000E+00 1.000E+00 0.000E+00
                             234- 238 D
                                              6.000E-01 0.000E+00 0.000E+00 1.000E+00
 10 SI  2.299  3.609  1.440
 11 SI -2.200  2.770 -7.325
 12 SI -2.299 -3.609 -1.440
 13 O   1.638  0.197  7.788
                                  305 S
                                              8.020E+03 1.080E-03 0.000E+00 0.000E+00
                                              1.338E+03 8.040E-03 0.000E+00 0.000E+00
                                              2.554E+02 5.324E-02 0.000E+00 0.000E+00
                                              6.922E+01 1.681E-01 0.000E+00 0.000E+00
                                              2.390E+01 3.581E-01 0.000E+00 0.000E+00
                                              9.264E+00 3.855E-01 0.000E+00 0.000E+00
                                              3.851E+00 1.468E-01 0.000E+00 0.000E+00
                                              1.212E+00 7.280E-02 0.000E+00 0.000E+00
                             306- 309 SP
                                              4.943E+01-8.830E-03 9.580E-03 0.000E+00
                                              1.047E+01-9.150E-02 6.960E-02 0.000E+00
                                              3.235E+00-4.020E-02 2.065E-01 0.000E+00
                                              1.217E+00 3.790E-01 3.470E-01 0.000E+00
                             310- 313 SP
                                              4.860E-01 1.000E+00 1.000E+00 0.000E+00
                             314- 317 SP
```

```
                                  1.925E-01 1.000E+00 1.000E+00 0.000E+00
                      318- 322 D
                                  2.000E+00 0.000E+00 0.000E+00 1.000E+00
                      323- 327 D
                                  5.000E-01 0.000E+00 0.000E+00 1.000E+00
 14 O   2.861 -6.182  0.976
 15 O  -1.638 -0.197 -7.788
 16 O  -2.861  6.182 -0.976
 17 O  -0.407 -4.226  6.378
 18 O   4.906  2.153  2.386
 19 O   0.407  4.226 -6.378
 20 O  -4.906 -2.153 -2.386
 21 O   4.541 -3.161  5.318
 22 O  -0.042  3.217  3.446
 23 O  -4.541  3.161 -5.318
 24 O   0.042 -3.217 -3.446
 25 O   4.251 -4.232 -7.505
 26 O   1.657  2.147 -1.259
 27 O  -4.251  4.232  7.505
 28 O  -1.657 -2.147  1.259
INFORMATION **** READM2 **** FULL DIRECT SCF (MONO AND BIEL INT) SELECTED
INFORMATION **** TOLINTEG **** COULOMB AND EXCHANGE SERIES TOLERANCES MODIFIED
INFORMATION **** MAXCYCLE **** MAX NUMBER OF SCF CYCLES SET TO         800
INFORMATION **** LEVSHIFT **** LEVEL SHIFTER ACTIVE
INFORMATION **** TOLDEE **** SCF TOL ON TOTAL ENERGY SET TO          10
*******************************************************************************
N. OF ATOMS PER CELL       28  COULOMB OVERLAP TOL         (T1) 10** -6
NUMBER OF SHELLS          176  COULOMB PENETRATION TOL     (T2) 10** -6
NUMBER OF AO              672  EXCHANGE OVERLAP TOL        (T3) 10** 20
N. OF ELECTRONS PER CELL  344  EXCHANGE PSEUDO OVP (F(G))  (T4) 10** 20
CORE ELECTRONS PER CELL   216  EXCHANGE PSEUDO OVP (P(G))  (T5) 10** 20
N. OF SYMMETRY OPERATORS    4  POLE ORDER IN MONO ZONE              4
*******************************************************************************
TYPE OF CALCULATION :  RESTRICTED CLOSED SHELL
KOHN-SHAM HAMILTONIAN

(EXCHANGE)[CORRELATION] FUNCTIONAL:(DIRAC-SLATER LDA)[VOSKO-WILK-NUSAIR]

EIGENVALUE LEVEL SHIFTING OF  0.600 HARTREE
LOCKING - FERMI ENERGY ALTERED BY LEVEL SHIFTER

CAPPA:IS1  6;IS2  6;IS3  6; K PTS MONK NET   80; SYMMOPS: K SPACE  4;G SPACE  4

*******************************************************************************
MAX NUMBER OF SCF CYCLES     800  CONVERGENCE ON DELTAP        10**-20
WEIGHT OF F(I) IN F(I+1)      35% CONVERGENCE ON ENERGY        10**-10
EIGENVALUE LEVEL SHIFTING OF  0.600 HARTREE
LOCKING - FERMI ENERGY ALTERED BY LEVEL SHIFTER
SHRINK. FACT.(MONKH.)    6  6  6  NUMBER OF K POINTS IN THE IBZ     80
SHRINKING FACTOR(GILAT NET)    6  NUMBER OF K POINTS(GILAT NET)     80
*******************************************************************************
*** K POINTS COORDINATES (OBLIQUE COORDINATES IN UNITS OF IS =  6)
  1-R(  0  0  0)   2-C(  1  0  0)   3-C(  2  0  0)   4-R(  3  0  0)
  5-C(  0  1  0)   6-C(  1  1  0)   7-C(  2  1  0)   8-C(  3  1  0)
  9-C(  0  2  0)  10-C(  1  2  0)  11-C(  2  2  0)  12-C(  3  2  0)
 13-R(  0  3  0)  14-C(  1  3  0)  15-C(  2  3  0)  16-R(  3  3  0)
 17-C(  0  0  1)  18-C(  1  0  1)  19-C(  2  0  1)  20-C(  3  0  1)
 21-C(  4  0  1)  22-C(  5  0  1)  23-C(  0  1  1)  24-C(  1  1  1)
 25-C(  2  1  1)  26-C(  3  1  1)  27-C(  4  1  1)  28-C(  5  1  1)
 29-C(  0  2  1)  30-C(  1  2  1)  31-C(  2  2  1)  32-C(  3  2  1)
 33-C(  4  2  1)  34-C(  5  2  1)  35-C(  0  3  1)  36-C(  1  3  1)
 37-C(  2  3  1)  38-C(  3  3  1)  39-C(  4  3  1)  40-C(  5  3  1)
 41-C(  0  0  2)  42-C(  1  0  2)  43-C(  2  0  2)  44-C(  3  0  2)
 45-C(  4  0  2)  46-C(  5  0  2)  47-C(  0  1  2)  48-C(  1  1  2)
 49-C(  2  1  2)  50-C(  3  1  2)  51-C(  4  1  2)  52-C(  5  1  2)
 53-C(  0  2  2)  54-C(  1  2  2)  55-C(  2  2  2)  56-C(  3  2  2)
 57-C(  4  2  2)  58-C(  5  2  2)  59-C(  0  3  2)  60-C(  1  3  2)
 61-C(  2  3  2)  62-C(  3  3  2)  63-C(  4  3  2)  64-C(  5  3  2)
 65-R(  0  0  3)  66-C(  1  0  3)  67-C(  2  0  3)  68-R(  3  0  3)
 69-C(  0  1  3)  70-C(  1  1  3)  71-C(  2  1  3)  72-C(  3  1  3)
 73-C(  0  2  3)  74-C(  1  2  3)  75-C(  2  2  3)  76-C(  3  2  3)
 77-R(  0  3  3)  78-C(  1  3  3)  79-C(  2  3  3)  80-R(  3  3  3)

DIRECT LATTICE VECTORS COMPON. (A.U.)      RECIP. LATTICE VECTORS COMPON. (A.U.)
       X            Y            Z              X            Y            Z
  10.4076667    0.0000000    0.0000000      0.6037074    0.0000000    0.0485413
   0.0000000   12.7573522    0.0000000     -0.0000000    0.4925148   -0.0000000
  -1.4093680    0.0000000   17.5282774      0.0000000    0.0000000    0.3584599
```

```
DISK SPACE FOR EIGENVECTORS (FTN 10)   68640768 REALS

SYMMETRY ADAPTION OF THE BLOCH FUNCTIONS ENABLED
TTTTTTTTTTTTTTTTTTTTTTTTTTTTTT gordsh1     TELAPSE        0.02 TCPU        0.01

DIMENSIONS  P(G)=  389912 F(G)=  195388 P(G),F(G) (IRR)   49967
MAX G-VECTOR INDEX FOR 1- AND 2-ELECTRON INTEGRALS  33

TTTTTTTTTTTTTTTTTTTTTTTTTTTTTT INPUT       TELAPSE        0.03 TCPU        0.02

NEIGHBORS OF THE NON-EQUIVALENT ATOMS

N = NUMBER OF NEIGHBORS AT DISTANCE R
   ATOM  N     R/ANG      R/AU   NEIGHBORS (ATOM LABELS AND CELL INDICES)
  1 CA   1     2.2221     4.1991  13 O    0 0 0
  1 CA   1     2.3376     4.4175  27 O    1 0 0
  1 CA   1     2.3849     4.5068  19 O    0 0 1
  1 CA   1     2.4617     4.6519  22 O    0 0 0
  1 CA   1     2.5500     4.8188  17 O    0 1 0
  1 CA   1     2.6079     4.9282  25 O    0 1 1

  5 CA   1     2.3802     4.4979  22 O    0-1 0
  5 CA   1     2.3803     4.4981  17 O    0 0 0
  5 CA   1     2.3890     4.5146  21 O   -1 0 0
  5 CA   1     2.3959     4.5277  16 O    0-1 0
  5 CA   1     2.4678     4.6635  27 O    0-1 0
  5 CA   1     2.6445     4.9974  28 O    0 0 0

  9 SI   1     1.6167     3.0551  13 O    0 0 0
  9 SI   1     1.6445     3.1077  21 O    0 0 0
  9 SI   1     1.6577     3.1326  17 O    0 0 0
  9 SI   1     1.6592     3.1355  25 O    0 0 1
  9 SI   1     2.9751     5.6222   1 CA   0-1 0
  9 SI   1     3.0351     5.7356   8 CA   0 0 1

 13 O    1     1.6167     3.0551   9 SI   0 0 0
 13 O    1     2.2221     4.1991   1 CA   0 0 0
 13 O    1     2.3959     4.5277   8 CA   0 0 1
 13 O    1     2.6757     5.0563   6 CA   0 0 0
 13 O    1     2.6843     5.0725  17 O    0 0 0
 13 O    1     2.6881     5.0799  21 O    0 0 0

 17 O    1     1.6577     3.1326   9 SI   0 0 0
 17 O    1     2.3803     4.4981   5 CA   0 0 0
 17 O    1     2.3849     4.5068   3 CA   0 0 1
 17 O    1     2.5500     4.8188   1 CA   0-1 0
 17 O    1     2.5839     4.8829  25 O    0 0 1
 17 O    1     2.6447     4.9978   6 CA   0 0 0

 21 O    1     1.6445     3.1077   9 SI   0 0 0
 21 O    1     2.3802     4.4979   6 CA   1 0 0
 21 O    1     2.3890     4.5146   5 CA   1 0 0
 21 O    1     2.4617     4.6519   2 CA   0 0 0
 21 O    1     2.6881     5.0799  13 O    0 0 0
 21 O    1     2.7072     5.1159  25 O    0 0 1

 25 O    1     1.6592     3.1355   9 SI   0 0-1
 25 O    1     2.3376     4.4175   3 CA   1 0 0
 25 O    1     2.4678     4.6635   7 CA   0-1 0
 25 O    1     2.5839     4.8829  17 O    0 0-1
 25 O    1     2.6079     4.9282   1 CA   0-1-1
 25 O    1     2.6445     4.9974   8 CA   0 0 0


SYMMETRY ALLOWED INTERNAL DEGREE(S) OF FREEDOM:  21
TTTTTTTTTTTTTTTTTTTTTTTTTTTTTT SYMM        TELAPSE        0.38 TCPU        0.37
TTTTTTTTTTTTTTTTTTTTTTTTTTTTTT INT_SCREEN  TELAPSE        0.39 TCPU        0.37

*******************************************************************************
*                                                                             *
*                                                                             *
*         FFFFF  RRRR   EEEE   EEE   U   U  EEEE  N   N   CCC  Y   Y          *
*         F      R   R  E     E   E  U   U  E     NN  N  C      Y Y           *
*         FFF    RRRR   EEEE  E   E  U   U  EEEE  N N N  C       Y            *
*         F      R   R  E     E  EE  U   U  E     N  NN  C       Y            *
*         F      R   R  EEEE   EE E   UUU   EEEE  N   N   CCC    Y            *
*                                                                             *
*                                                                             *
* CALCULATION OF PHONON FREQUENCIES AT THE GAMMA POINT.                       *
```

```
*                                                                              *
* SYMMETRY IS EXPLOITED TO BUILD THE TOTAL HESSIAN MATRIX.                     *
* (F. PASCALE PHD THESIS TURIN-PARIS 2002)                                     *
*                                                                              *
********************************************************************************


*                                                                              *
* REFERENCES TO BE QUOTED WHEN USING THIS MODULE:                              *
*                                                                              *
* F. Pascale, C.M. Zicovich-Wilson, F. Lopez, B. Civalleri                     *
* R. Orlando, R. Dovesi                                                        *
* The calculation of the vibration frequencies of crystalline                  *
* compounds and its implementation in the CRYSTAL code                         *
* J. Comput. Chem. 25 (2004) 888-897                                           *
*                                                                              *
* C.M. Zicovich-Wilson, F. Pascale, C. Roetti, V.R. Saunders,                  *
* R. Orlando, R. Dovesi                                                        *
* The calculation of the vibration frequencies of alpha-quartz:                *
* the effect of hamiltonian and basis set                                      *
* J. Comput. Chem. 25 (2004) 1873-1881                                         *
********************************************************************************



ATOMS ISOTOPIC MASS (AMU) FOR FREQUENCY CALCULATION

   1 CA     39.9626    2 CA     39.9626    3 CA     39.9626    4 CA     39.9626
   5 CA     39.9626    6 CA     39.9626    7 CA     39.9626    8 CA     39.9626
   9 SI     27.9769   10 SI     27.9769   11 SI     27.9769   12 SI     27.9769
  13 O      15.9949   14 O      15.9949   15 O      15.9949   16 O      15.9949
  17 O      15.9949   18 O      15.9949   19 O      15.9949   20 O      15.9949
  21 O      15.9949   22 O      15.9949   23 O      15.9949   24 O      15.9949
  25 O      15.9949   26 O      15.9949   27 O      15.9949   28 O      15.9949

STEP SIZE                  0.0030 ANGSTROM

INFORMATION CONCERNING THE SCF+GRADIENT CALCULATIONS REQUIRED FOR
GENERATING FREQUENCIES. IN PRINCIPLE 3N+1 SCF + GRADIENT
CALCULATIONS  ARE REQUIRED;
FOR EACH OF THEM THE REMAINING POINT SYMMETRY IS INDICATED.
POINT SYMMETRY PERMITS TO GENERATE GRADIENTS FOR DISPLACEMENT B
STARTING FROM THE GRADIENT GENERATED BY DISPLACEMENT A.

  N   LABEL SYMBOL DISPLACEMENT     SYM.

  1     EQUILIBRIUM GEOMETRY            4

  2      1   CA           DX           1
  3      1   CA           DY           1
  4      1   CA           DZ           1
  5      5   CA           DX           1
  6      5   CA           DY           1
  7      5   CA           DZ           1
  8      9   SI           DX           1
  9      9   SI           DY           1
 10      9   SI           DZ           1
 11     13   O            DX           1
 12     13   O            DY           1
 13     13   O            DZ           1
 14     17   O            DX           1
 15     17   O            DY           1
 16     17   O            DZ           1
 17     21   O            DX           1
 18     21   O            DY           1
 19     21   O            DZ           1
 20     25   O            DX           1
 21     25   O            DY           1
 22     25   O            DZ           1
USE OF RESIDUAL SYMMETRY AFTER DISPLACEMENT

NUMERICAL GRADIENT COMPUTED WITH A SINGLE DISPLACEMENT (+dx) FOR EACH
CARTESIAN COORDINATE WITH RESPECT TO THE EQUILIBRIUM CONFIGURATION
dx=  0.003
NUMBER OF IRREDUCIBLE ATOMS                    7
NUMBER OF SCF+GRADIENT CALCULATIONS           22

 ATOM  SYMOP  ORDER
   1      1      1
```

```
   5      1      1
   9      1      1
  13      1      1
  17      1      1
  21      1      1
  25      1      1

ATOM  : IRREDUCIBLE ATOM
SYMOP : NUMBER OF SYMMETRY OPERATORS THAT DOESN'T MOVE THE IRREDUCIBLE ATOM
ORDER : MAXIMUM ORDER AMONG THE OPERATORS OF THE IRREDUCIBLE ATOM

*******************************************************************************


GCALCO - MAX INDICES DIRECT LATTICE VECTOR    19    15    11
NO.OF VECTORS CREATED 6999 STARS 1862 RMAX   157.41387 BOHR

CAPPA:IS1  6;IS2  6;IS3  6; K PTS MONK NET   80; SYMMOPS: K SPACE  4;G SPACE  4

TTTTTTTTTTTTTTTTTTTTTTTTTTTTTT gordsh1     TELAPSE        0.42 TCPU        0.41

DIMENSIONS  P(G)=  389912 F(G)=  195388 P(G),F(G) (IRR)   49967
MAX G-VECTOR INDEX FOR 1- AND 2-ELECTRON INTEGRALS  33

TTTTTTTTTTTTTTTTTTTTTTTTTTTTTT INPUT       TELAPSE        0.42 TCPU        0.41

SYMMETRY ALLOWED INTERNAL DEGREE(S) OF FREEDOM:  21
TTTTTTTTTTTTTTTTTTTTTTTTTTTTTT SYMM        TELAPSE        0.68 TCPU        0.68
TTTTTTTTTTTTTTTTTTTTTTTTTTTTTT INT_SCREEN  TELAPSE        0.69 TCPU        0.68
INFORMATION **** EXCBUF **** EXCH. BIPO BUFFER: WORDS USED =           0

DFT PARAMETERS

    ATOM       ELECTRONS   NET CHARGE   R(ANGSTROM)
  1  20  CA     20.0000      0.0000     1.97000000
  9  14  SI     14.0000      0.0000     1.17000000
 13   8  O       8.0000      0.0000     0.74000000

SIZE OF GRID=     145905
TTTTTTTTTTTTTTTTTTTTTTTTTTTTTT MAKE_GRID2  TELAPSE        7.25 TCPU        7.24
BECKE WEIGHT FUNCTION
RADSAFE =     2.00
TOLERANCES - DENSITY:10**- 6; POTENTIAL:10**- 9; GRID WGT:10**-14

RADIAL INTEGRATION  - INTERVALS (POINTS,UPPER LIMIT):            1( 75,  4.0*R)

ANGULAR INTEGRATION - INTERVALS (ACCURACY LEVEL [N. POINTS] UPPER LIMIT):
 1(  4[  86]   0.2)  2(  8[ 194]   0.5)  3( 12[ 350]   0.9)  4( 16[ 974]   3.5)
 5( 12[ 350]9999.0)
TTTTTTTTTTTTTTTTTTTTTTTTTTTTTT INT_CALC    TELAPSE        7.28 TCPU        7.27

*******************************************************************************
Ca2SiO4 cif file
CRYSTAL - SCF - TYPE OF CALCULATION :  RESTRICTED CLOSED SHELL
*******************************************************************************

CAPPA:IS1  6;IS2  6;IS3  6; K PTS MONK NET   80; SYMMOPS: K SPACE  4;G SPACE  4

TTTTTTTTTTTTTTTTTTTTTTTTTTTTTT SDIK        TELAPSE        8.49 TCPU        8.48

AAAAAAAAAAAAAAAAAAAAAAAAAAAAAAAAAAAAAAAAAAAAAAAAAAAAAAAAAAAAAAAAAAAAAAAAAAAAAAA
        ATOMIC WAVEFUNCTION(S)


NUCLEAR CHARGE 20.0  SYMMETRY SPECIES            S    P
N. ELECTRONS   20.0  NUMBER OF PRIMITIVE GTOS   21   13
                     NUMBER OF CONTRACTED GTOS   5    4
                     NUMBER OF CLOSED SHELLS     4    2
                     OPEN SHELL OCCUPATION       0    0

 ZNUC SCFIT  TOTAL HF ENERGY   KINETIC ENERGY   VIRIAL THEOREM ACCURACY
 20.0   7   -6.759945013E+02  6.789840598E+02 -1.995597012E+00  3.2E-06

NUCLEAR CHARGE 14.0  SYMMETRY SPECIES            S    P
N. ELECTRONS   14.0  NUMBER OF PRIMITIVE GTOS   19   11
                     NUMBER OF CONTRACTED GTOS   5    4
                     NUMBER OF CLOSED SHELLS     3    1
                     OPEN SHELL OCCUPATION       0    2
```

```
 ZNUC SCFIT  TOTAL HF ENERGY   KINETIC ENERGY   VIRIAL THEOREM ACCURACY
 14.0  10   -2.888271346E+02  2.895266850E+02 -1.997583814E+00  2.8E-06

NUCLEAR CHARGE  8.0  SYMMETRY SPECIES            S    P
N. ELECTRONS    8.0  NUMBER OF PRIMITIVE GTOS   14    6
                     NUMBER OF CONTRACTED GTOS   4    3
                     NUMBER OF CLOSED SHELLS     2    0
                     OPEN SHELL OCCUPATION       0    4

 ZNUC SCFIT  TOTAL HF ENERGY   KINETIC ENERGY   VIRIAL THEOREM ACCURACY
  8.0  13   -7.480044816E+01  7.461868841E+01 -2.002435848E+00  2.7E-06

AAAAAAAAAAAAAAAAAAAAAAAAAAAAAAAAAAAAAAAAAAAAAAAAAAAAAAAAAAAAAAAAAAAAAAAAAAAAAAAA

TTTTTTTTTTTTTTTTTTTTTTTTTTTTTT MOQGAD      TELAPSE        9.06 TCPU        9.05
TTTTTTTTTTTTTTTTTTTTTTTTTTTTTT SHELLX      TELAPSE       11.56 TCPU       11.55
TTTTTTTTTTTTTTTTTTTTTTTTTTTTTT MONMO3      TELAPSE       11.90 TCPU       11.89
NUMERICALLY INTEGRATED DENSITY    344.0006883459
TTTTTTTTTTTTTTTTTTTTTTTTTTTTTT NUMDFT      TELAPSE       12.39 TCPU       12.38
CYC   0 ETOT(AU) -7.752307743317E+03 DETOT -7.75E+03 tst  0.00E+00 PX  1.00E+00
TTTTTTTTTTTTTTTTTTTTTTTTTTTTTT FDIK        TELAPSE       57.81 TCPU       57.80
INSULATING STATE - LEVEL SHIFTER    0.60 au
TOP OF VALENCE BANDS -    BAND    172; K   68; EIG -3.3657655E-01 AU
TOP OF VALENCE BANDS -    BAND    172; K    1; EIG -3.4268257E-01 AU
BOTTOM OF VIRTUAL BANDS - BAND    173; K    1; EIG -5.2645346E-02 AU
TTTTTTTTTTTTTTTTTTTTTTTTTTTTTT PDIG        TELAPSE       58.87 TCPU       58.86
TTTTTTTTTTTTTTTTTTTTTTTTTTTTTT MOQGAD      TELAPSE       58.90 TCPU       58.88
TTTTTTTTTTTTTTTTTTTTTTTTTTTTTT SHELLX      TELAPSE       61.94 TCPU       61.93
TTTTTTTTTTTTTTTTTTTTTTTTTTTTTT MONMO3      TELAPSE       62.29 TCPU       62.27
NUMERICALLY INTEGRATED DENSITY    344.0007440116
TTTTTTTTTTTTTTTTTTTTTTTTTTTTTT NUMDFT      TELAPSE       63.18 TCPU       63.17
CYC   1 ETOT(AU) -7.755363207893E+03 DETOT -3.06E+00 tst  0.00E+00 PX  1.00E+00
TTTTTTTTTTTTTTTTTTTTTTTTTTTTTT FDIK        TELAPSE       78.75 TCPU       78.73
INSULATING STATE - LEVEL SHIFTER    0.60 au
TOP OF VALENCE BANDS -    BAND    172; K    1; EIG -6.3583528E-01 AU
BOTTOM OF VIRTUAL BANDS - BAND    173; K    1; EIG  6.1897450E-02 AU
TTTTTTTTTTTTTTTTTTTTTTTTTTTTTT PDIG        TELAPSE       79.81 TCPU       79.78
TTTTTTTTTTTTTTTTTTTTTTTTTTTTTT MOQGAD      TELAPSE       79.83 TCPU       79.81
TTTTTTTTTTTTTTTTTTTTTTTTTTTTTT SHELLX      TELAPSE       82.85 TCPU       82.83
TTTTTTTTTTTTTTTTTTTTTTTTTTTTTT MONMO3      TELAPSE       83.21 TCPU       83.19
NUMERICALLY INTEGRATED DENSITY    344.0009159677
TTTTTTTTTTTTTTTTTTTTTTTTTTTTTT NUMDFT      TELAPSE       84.08 TCPU       84.06
CYC   2 ETOT(AU) -7.757656663868E+03 DETOT -2.29E+00 tst  2.12E-02 PX  1.67E-01
TTTTTTTTTTTTTTTTTTTTTTTTTTTTTT FDIK        TELAPSE       98.58 TCPU       98.23
INSULATING STATE - LEVEL SHIFTER    0.60 au
TOP OF VALENCE BANDS -    BAND    172; K    1; EIG -7.0172452E-01 AU
BOTTOM OF VIRTUAL BANDS - BAND    173; K    1; EIG  5.1551244E-02 AU
TTTTTTTTTTTTTTTTTTTTTTTTTTTTTT PDIG        TELAPSE       99.64 TCPU       99.29
TTTTTTTTTTTTTTTTTTTTTTTTTTTTTT MOQGAD      TELAPSE       99.67 TCPU       99.32
TTTTTTTTTTTTTTTTTTTTTTTTTTTTTT SHELLX      TELAPSE      102.69 TCPU      102.34
TTTTTTTTTTTTTTTTTTTTTTTTTTTTTT MONMO3      TELAPSE      103.04 TCPU      102.69
NUMERICALLY INTEGRATED DENSITY    344.0008959092
TTTTTTTTTTTTTTTTTTTTTTTTTTTTTT NUMDFT      TELAPSE      103.92 TCPU      103.57
CYC   3 ETOT(AU) -7.757730758006E+03 DETOT -7.41E-02 tst  1.59E-03 PX  5.10E-02
TTTTTTTTTTTTTTTTTTTTTTTTTTTTTT FDIK        TELAPSE      117.40 TCPU      117.05
INSULATING STATE - LEVEL SHIFTER    0.60 au
TOP OF VALENCE BANDS -    BAND    172; K    1; EIG -7.4594343E-01 AU
BOTTOM OF VIRTUAL BANDS - BAND    173; K    1; EIG  3.7226642E-02 AU
TTTTTTTTTTTTTTTTTTTTTTTTTTTTTT PDIG        TELAPSE      118.46 TCPU      118.11
TTTTTTTTTTTTTTTTTTTTTTTTTTTTTT MOQGAD      TELAPSE      118.48 TCPU      118.13
TTTTTTTTTTTTTTTTTTTTTTTTTTTTTT SHELLX      TELAPSE      121.51 TCPU      121.16
TTTTTTTTTTTTTTTTTTTTTTTTTTTTTT MONMO3      TELAPSE      121.86 TCPU      121.51
NUMERICALLY INTEGRATED DENSITY    344.0008614976
TTTTTTTTTTTTTTTTTTTTTTTTTTTTTT NUMDFT      TELAPSE      122.73 TCPU      122.38
CYC   4 ETOT(AU) -7.757780145819E+03 DETOT -4.94E-02 tst  2.25E-04 PX  3.40E-02
TTTTTTTTTTTTTTTTTTTTTTTTTTTTTT FDIK        TELAPSE      135.47 TCPU      135.11
INSULATING STATE - LEVEL SHIFTER    0.60 au
TOP OF VALENCE BANDS -    BAND    172; K    1; EIG -7.4515386E-01 AU
BOTTOM OF VIRTUAL BANDS - BAND    173; K    1; EIG  3.6329158E-02 AU
TTTTTTTTTTTTTTTTTTTTTTTTTTTTTT PDIG        TELAPSE      136.52 TCPU      136.17
TTTTTTTTTTTTTTTTTTTTTTTTTTTTTT MOQGAD      TELAPSE      136.55 TCPU      136.20
TTTTTTTTTTTTTTTTTTTTTTTTTTTTTT SHELLX      TELAPSE      139.57 TCPU      139.22
TTTTTTTTTTTTTTTTTTTTTTTTTTTTTT MONMO3      TELAPSE      139.92 TCPU      139.57
NUMERICALLY INTEGRATED DENSITY    344.0008562403
TTTTTTTTTTTTTTTTTTTTTTTTTTTTTT NUMDFT      TELAPSE      140.80 TCPU      140.45
CYC   5 ETOT(AU) -7.757782171810E+03 DETOT -2.03E-03 tst  2.87E-05 PX  7.03E-03
TTTTTTTTTTTTTTTTTTTTTTTTTTTTTT FDIK        TELAPSE      155.89 TCPU      154.47
INSULATING STATE - LEVEL SHIFTER    0.60 au
TOP OF VALENCE BANDS -    BAND    172; K    1; EIG -7.4306067E-01 AU
```

```
BOTTOM OF VIRTUAL BANDS - BAND    173; K    1; EIG  3.6277538E-02 AU
TTTTTTTTTTTTTTTTTTTTTTTTTTTTTT PDIG        TELAPSE      156.94 TCPU      155.53
TTTTTTTTTTTTTTTTTTTTTTTTTTTTTT MOQGAD      TELAPSE      156.97 TCPU      155.55
TTTTTTTTTTTTTTTTTTTTTTTTTTTTTT SHELLX      TELAPSE      159.99 TCPU      158.57
TTTTTTTTTTTTTTTTTTTTTTTTTTTTTT MONMO3      TELAPSE      160.35 TCPU      158.93
NUMERICALLY INTEGRATED DENSITY    344.0008574063
TTTTTTTTTTTTTTTTTTTTTTTTTTTTTT NUMDFT      TELAPSE      161.23 TCPU      159.81
CYC   6 ETOT(AU) -7.757783150526E+03 DETOT -9.79E-04 tst  2.27E-05 PX  5.90E-03
TTTTTTTTTTTTTTTTTTTTTTTTTTTTTT FDIK        TELAPSE      173.02 TCPU      171.59
INSULATING STATE - LEVEL SHIFTER    0.60 au
TOP OF VALENCE BANDS -    BAND    172; K    1; EIG -7.4479991E-01 AU
BOTTOM OF VIRTUAL BANDS - BAND    173; K    1; EIG  3.5366494E-02 AU
TTTTTTTTTTTTTTTTTTTTTTTTTTTTTT PDIG        TELAPSE      174.09 TCPU      172.66
TTTTTTTTTTTTTTTTTTTTTTTTTTTTTT MOQGAD      TELAPSE      174.11 TCPU      172.69
TTTTTTTTTTTTTTTTTTTTTTTTTTTTTT SHELLX      TELAPSE      177.14 TCPU      175.71
TTTTTTTTTTTTTTTTTTTTTTTTTTTTTT MONMO3      TELAPSE      177.50 TCPU      176.07
NUMERICALLY INTEGRATED DENSITY    344.0008568813
TTTTTTTTTTTTTTTTTTTTTTTTTTTTTT NUMDFT      TELAPSE      178.37 TCPU      176.94
CYC   7 ETOT(AU) -7.757783293363E+03 DETOT -1.43E-04 tst  9.05E-06 PX  3.41E-03
TTTTTTTTTTTTTTTTTTTTTTTTTTTTTT FDIK        TELAPSE      190.97 TCPU      189.54
INSULATING STATE - LEVEL SHIFTER    0.60 au
TOP OF VALENCE BANDS -    BAND    172; K    1; EIG -7.4587947E-01 AU
BOTTOM OF VIRTUAL BANDS - BAND    173; K    1; EIG  3.4848394E-02 AU
TTTTTTTTTTTTTTTTTTTTTTTTTTTTTT PDIG        TELAPSE      192.02 TCPU      190.60
TTTTTTTTTTTTTTTTTTTTTTTTTTTTTT MOQGAD      TELAPSE      192.05 TCPU      190.62
TTTTTTTTTTTTTTTTTTTTTTTTTTTTTT SHELLX      TELAPSE      195.07 TCPU      193.64
TTTTTTTTTTTTTTTTTTTTTTTTTTTTTT MONMO3      TELAPSE      195.44 TCPU      194.01
NUMERICALLY INTEGRATED DENSITY    344.0008562216
TTTTTTTTTTTTTTTTTTTTTTTTTTTTTT NUMDFT      TELAPSE      196.31 TCPU      194.88
CYC   8 ETOT(AU) -7.757783356682E+03 DETOT -6.33E-05 tst  3.72E-06 PX  1.80E-03
TTTTTTTTTTTTTTTTTTTTTTTTTTTTTT FDIK        TELAPSE      206.58 TCPU      202.66
INSULATING STATE - LEVEL SHIFTER    0.60 au
TOP OF VALENCE BANDS -    BAND    172; K    1; EIG -7.4608235E-01 AU
BOTTOM OF VIRTUAL BANDS - BAND    173; K    1; EIG  3.4686577E-02 AU
TTTTTTTTTTTTTTTTTTTTTTTTTTTTTT PDIG        TELAPSE      207.64 TCPU      203.71
TTTTTTTTTTTTTTTTTTTTTTTTTTTTTT MOQGAD      TELAPSE      207.67 TCPU      203.74
TTTTTTTTTTTTTTTTTTTTTTTTTTTTTT SHELLX      TELAPSE      210.69 TCPU      206.76
TTTTTTTTTTTTTTTTTTTTTTTTTTTTTT MONMO3      TELAPSE      211.05 TCPU      207.12
NUMERICALLY INTEGRATED DENSITY    344.0008560359
TTTTTTTTTTTTTTTTTTTTTTTTTTTTTT NUMDFT      TELAPSE      211.92 TCPU      207.99
CYC   9 ETOT(AU) -7.757783374664E+03 DETOT -1.80E-05 tst  1.87E-06 PX  1.20E-03
TTTTTTTTTTTTTTTTTTTTTTTTTTTTTT FDIK        TELAPSE      222.19 TCPU      218.27
INSULATING STATE - LEVEL SHIFTER    0.60 au
TOP OF VALENCE BANDS -    BAND    172; K    1; EIG -7.4616976E-01 AU
BOTTOM OF VIRTUAL BANDS - BAND    173; K    1; EIG  3.4604658E-02 AU
TTTTTTTTTTTTTTTTTTTTTTTTTTTTTT PDIG        TELAPSE      223.25 TCPU      219.32
TTTTTTTTTTTTTTTTTTTTTTTTTTTTTT MOQGAD      TELAPSE      223.28 TCPU      219.35
TTTTTTTTTTTTTTTTTTTTTTTTTTTTTT SHELLX      TELAPSE      226.30 TCPU      222.37
TTTTTTTTTTTTTTTTTTTTTTTTTTTTTT MONMO3      TELAPSE      226.66 TCPU      222.73
NUMERICALLY INTEGRATED DENSITY    344.0008559791
TTTTTTTTTTTTTTTTTTTTTTTTTTTTTT NUMDFT      TELAPSE      227.54 TCPU      223.61
CYC  10 ETOT(AU) -7.757783381372E+03 DETOT -6.71E-06 tst  9.40E-07 PX  8.17E-04
TTTTTTTTTTTTTTTTTTTTTTTTTTTTTT FDIK        TELAPSE      236.75 TCPU      232.81
INSULATING STATE - LEVEL SHIFTER    0.60 au
TOP OF VALENCE BANDS -    BAND    172; K    1; EIG -7.4626916E-01 AU
BOTTOM OF VIRTUAL BANDS - BAND    173; K    1; EIG  3.4544574E-02 AU
TTTTTTTTTTTTTTTTTTTTTTTTTTTTTT PDIG        TELAPSE      237.80 TCPU      233.86
TTTTTTTTTTTTTTTTTTTTTTTTTTTTTT MOQGAD      TELAPSE      237.83 TCPU      233.89
TTTTTTTTTTTTTTTTTTTTTTTTTTTTTT SHELLX      TELAPSE      240.84 TCPU      236.91
TTTTTTTTTTTTTTTTTTTTTTTTTTTTTT MONMO3      TELAPSE      241.21 TCPU      237.27
NUMERICALLY INTEGRATED DENSITY    344.0008559215
TTTTTTTTTTTTTTTTTTTTTTTTTTTTTT NUMDFT      TELAPSE      242.09 TCPU      238.15
CYC  11 ETOT(AU) -7.757783383892E+03 DETOT -2.52E-06 tst  4.66E-07 PX  5.06E-04
TTTTTTTTTTTTTTTTTTTTTTTTTTTTTT FDIK        TELAPSE      252.13 TCPU      248.19
INSULATING STATE - LEVEL SHIFTER    0.60 au
TOP OF VALENCE BANDS -    BAND    172; K    1; EIG -7.4632826E-01 AU
BOTTOM OF VIRTUAL BANDS - BAND    173; K    1; EIG  3.4511730E-02 AU
TTTTTTTTTTTTTTTTTTTTTTTTTTTTTT PDIG        TELAPSE      253.18 TCPU      249.24
TTTTTTTTTTTTTTTTTTTTTTTTTTTTTT MOQGAD      TELAPSE      253.21 TCPU      249.27
TTTTTTTTTTTTTTTTTTTTTTTTTTTTTT SHELLX      TELAPSE      256.23 TCPU      252.29
TTTTTTTTTTTTTTTTTTTTTTTTTTTTTT MONMO3      TELAPSE      256.59 TCPU      252.65
NUMERICALLY INTEGRATED DENSITY    344.0008558798
TTTTTTTTTTTTTTTTTTTTTTTTTTTTTT NUMDFT      TELAPSE      257.46 TCPU      253.52
CYC  12 ETOT(AU) -7.757783384849E+03 DETOT -9.57E-07 tst  2.33E-07 PX  3.26E-04
TTTTTTTTTTTTTTTTTTTTTTTTTTTTTT FDIK        TELAPSE      266.40 TCPU      262.46
INSULATING STATE - LEVEL SHIFTER    0.60 au
TOP OF VALENCE BANDS -    BAND    172; K    1; EIG -7.4635537E-01 AU
BOTTOM OF VIRTUAL BANDS - BAND    173; K    1; EIG  3.4495414E-02 AU
TTTTTTTTTTTTTTTTTTTTTTTTTTTTTT PDIG        TELAPSE      267.45 TCPU      263.51
```

```
TTTTTTTTTTTTTTTTTTTTTTTTTTTTTT MOQGAD      TELAPSE      267.48 TCPU      263.54
TTTTTTTTTTTTTTTTTTTTTTTTTTTTTT SHELLX      TELAPSE      270.51 TCPU      266.57
TTTTTTTTTTTTTTTTTTTTTTTTTTTTTT MONMO3      TELAPSE      270.85 TCPU      266.91
NUMERICALLY INTEGRATED DENSITY    344.0008558572
TTTTTTTTTTTTTTTTTTTTTTTTTTTTTT NUMDFT      TELAPSE      271.73 TCPU      267.79
CYC  13 ETOT(AU) -7.757783385152E+03 DETOT -3.04E-07 tst  1.21E-07 PX  2.12E-04
TTTTTTTTTTTTTTTTTTTTTTTTTTTTTT FDIK        TELAPSE      280.81 TCPU      276.87
INSULATING STATE - LEVEL SHIFTER    0.60 au
TOP OF VALENCE BANDS -    BAND    172; K    1; EIG -7.4637080E-01 AU
BOTTOM OF VIRTUAL BANDS - BAND    173; K    1; EIG  3.4485961E-02 AU
TTTTTTTTTTTTTTTTTTTTTTTTTTTTTT PDIG        TELAPSE      281.87 TCPU      277.93
TTTTTTTTTTTTTTTTTTTTTTTTTTTTTT MOQGAD      TELAPSE      281.89 TCPU      277.95
TTTTTTTTTTTTTTTTTTTTTTTTTTTTTT SHELLX      TELAPSE      284.91 TCPU      280.97
TTTTTTTTTTTTTTTTTTTTTTTTTTTTTT MONMO3      TELAPSE      285.27 TCPU      281.33
NUMERICALLY INTEGRATED DENSITY    344.0008558434
TTTTTTTTTTTTTTTTTTTTTTTTTTTTTT NUMDFT      TELAPSE      286.14 TCPU      282.20
CYC  14 ETOT(AU) -7.757783385233E+03 DETOT -8.07E-08 tst  6.81E-08 PX  1.38E-04
TTTTTTTTTTTTTTTTTTTTTTTTTTTTTT FDIK        TELAPSE      295.13 TCPU      291.17
INSULATING STATE - LEVEL SHIFTER    0.60 au
TOP OF VALENCE BANDS -    BAND    172; K    1; EIG -7.4638129E-01 AU
BOTTOM OF VIRTUAL BANDS - BAND    173; K    1; EIG  3.4479894E-02 AU
TTTTTTTTTTTTTTTTTTTTTTTTTTTTTT PDIG        TELAPSE      296.18 TCPU      292.23
TTTTTTTTTTTTTTTTTTTTTTTTTTTTTT MOQGAD      TELAPSE      296.21 TCPU      292.25
TTTTTTTTTTTTTTTTTTTTTTTTTTTTTT SHELLX      TELAPSE      299.23 TCPU      295.27
TTTTTTTTTTTTTTTTTTTTTTTTTTTTTT MONMO3      TELAPSE      299.58 TCPU      295.63
NUMERICALLY INTEGRATED DENSITY    344.0008558340
TTTTTTTTTTTTTTTTTTTTTTTTTTTTTT NUMDFT      TELAPSE      300.45 TCPU      296.49
CYC  15 ETOT(AU) -7.757783385238E+03 DETOT -4.89E-09 tst  3.83E-08 PX  9.21E-05
TTTTTTTTTTTTTTTTTTTTTTTTTTTTTT FDIK        TELAPSE      309.59 TCPU      305.64
INSULATING STATE - LEVEL SHIFTER    0.60 au
TOP OF VALENCE BANDS -    BAND    172; K    1; EIG -7.4638796E-01 AU
BOTTOM OF VIRTUAL BANDS - BAND    173; K    1; EIG  3.4476073E-02 AU
TTTTTTTTTTTTTTTTTTTTTTTTTTTTTT PDIG        TELAPSE      310.65 TCPU      306.69
TTTTTTTTTTTTTTTTTTTTTTTTTTTTTT MOQGAD      TELAPSE      310.68 TCPU      306.72
TTTTTTTTTTTTTTTTTTTTTTTTTTTTTT SHELLX      TELAPSE      313.69 TCPU      309.74
TTTTTTTTTTTTTTTTTTTTTTTTTTTTTT MONMO3      TELAPSE      314.05 TCPU      310.09
NUMERICALLY INTEGRATED DENSITY    344.0008558277
TTTTTTTTTTTTTTTTTTTTTTTTTTTTTT NUMDFT      TELAPSE      314.94 TCPU      310.98
CYC  16 ETOT(AU) -7.757783385218E+03 DETOT  1.99E-08 tst  2.16E-08 PX  6.07E-05
TTTTTTTTTTTTTTTTTTTTTTTTTTTTTT FDIK        TELAPSE      323.32 TCPU      319.37
INSULATING STATE - LEVEL SHIFTER    0.60 au
TOP OF VALENCE BANDS -    BAND    172; K    1; EIG -7.4639214E-01 AU
BOTTOM OF VIRTUAL BANDS - BAND    173; K    1; EIG  3.4473665E-02 AU
TTTTTTTTTTTTTTTTTTTTTTTTTTTTTT PDIG        TELAPSE      324.38 TCPU      320.42
TTTTTTTTTTTTTTTTTTTTTTTTTTTTTT MOQGAD      TELAPSE      324.41 TCPU      320.45
TTTTTTTTTTTTTTTTTTTTTTTTTTTTTT SHELLX      TELAPSE      327.43 TCPU      323.47
TTTTTTTTTTTTTTTTTTTTTTTTTTTTTT MONMO3      TELAPSE      327.79 TCPU      323.83
NUMERICALLY INTEGRATED DENSITY    344.0008558238
TTTTTTTTTTTTTTTTTTTTTTTTTTTTTT NUMDFT      TELAPSE      328.67 TCPU      324.71
CYC  17 ETOT(AU) -7.757783385198E+03 DETOT  1.97E-08 tst  1.22E-08 PX  4.07E-05
TTTTTTTTTTTTTTTTTTTTTTTTTTTTTT FDIK        TELAPSE      337.36 TCPU      333.40
INSULATING STATE - LEVEL SHIFTER    0.60 au
TOP OF VALENCE BANDS -    BAND    172; K    1; EIG -7.4639484E-01 AU
BOTTOM OF VIRTUAL BANDS - BAND    173; K    1; EIG  3.4472140E-02 AU
TTTTTTTTTTTTTTTTTTTTTTTTTTTTTT PDIG        TELAPSE      338.42 TCPU      334.46
TTTTTTTTTTTTTTTTTTTTTTTTTTTTTT MOQGAD      TELAPSE      338.44 TCPU      334.49
TTTTTTTTTTTTTTTTTTTTTTTTTTTTTT SHELLX      TELAPSE      341.47 TCPU      337.51
TTTTTTTTTTTTTTTTTTTTTTTTTTTTTT MONMO3      TELAPSE      341.82 TCPU      337.86
NUMERICALLY INTEGRATED DENSITY    344.0008558210
TTTTTTTTTTTTTTTTTTTTTTTTTTTTTT NUMDFT      TELAPSE      342.69 TCPU      338.74
CYC  18 ETOT(AU) -7.757783385180E+03 DETOT  1.86E-08 tst  6.86E-09 PX  2.70E-05
TTTTTTTTTTTTTTTTTTTTTTTTTTTTTT FDIK        TELAPSE      351.29 TCPU      347.33
INSULATING STATE - LEVEL SHIFTER    0.60 au
TOP OF VALENCE BANDS -    BAND    172; K    1; EIG -7.4639654E-01 AU
BOTTOM OF VIRTUAL BANDS - BAND    173; K    1; EIG  3.4471160E-02 AU
TTTTTTTTTTTTTTTTTTTTTTTTTTTTTT PDIG        TELAPSE      352.34 TCPU      348.38
TTTTTTTTTTTTTTTTTTTTTTTTTTTTTT MOQGAD      TELAPSE      352.37 TCPU      348.41
TTTTTTTTTTTTTTTTTTTTTTTTTTTTTT SHELLX      TELAPSE      355.39 TCPU      351.43
TTTTTTTTTTTTTTTTTTTTTTTTTTTTTT MONMO3      TELAPSE      355.75 TCPU      351.79
NUMERICALLY INTEGRATED DENSITY    344.0008558193
TTTTTTTTTTTTTTTTTTTTTTTTTTTTTT NUMDFT      TELAPSE      356.61 TCPU      352.65
CYC  19 ETOT(AU) -7.757783385166E+03 DETOT  1.33E-08 tst  3.87E-09 PX  1.82E-05
TTTTTTTTTTTTTTTTTTTTTTTTTTTTTT FDIK        TELAPSE      365.06 TCPU      361.10
INSULATING STATE - LEVEL SHIFTER    0.60 au
TOP OF VALENCE BANDS -    BAND    172; K    1; EIG -7.4639767E-01 AU
BOTTOM OF VIRTUAL BANDS - BAND    173; K    1; EIG  3.4470514E-02 AU
TTTTTTTTTTTTTTTTTTTTTTTTTTTTTT PDIG        TELAPSE      366.11 TCPU      362.16
TTTTTTTTTTTTTTTTTTTTTTTTTTTTTT MOQGAD      TELAPSE      366.14 TCPU      362.18
TTTTTTTTTTTTTTTTTTTTTTTTTTTTTT SHELLX      TELAPSE      369.16 TCPU      365.20
```

```
TTTTTTTTTTTTTTTTTTTTTTTTTTTTTT MONMO3      TELAPSE      369.51 TCPU      365.56
NUMERICALLY INTEGRATED DENSITY    344.0008558181
TTTTTTTTTTTTTTTTTTTTTTTTTTTTTT NUMDFT      TELAPSE      370.39 TCPU      366.43
CYC  20 ETOT(AU) -7.757783385157E+03 DETOT  9.08E-09 tst  2.19E-09 PX  1.21E-05
TTTTTTTTTTTTTTTTTTTTTTTTTTTTTT FDIK        TELAPSE      378.70 TCPU      374.74
INSULATING STATE - LEVEL SHIFTER    0.60 au
TOP OF VALENCE BANDS -    BAND    172; K    1; EIG -7.4639842E-01 AU
BOTTOM OF VIRTUAL BANDS - BAND    173; K    1; EIG  3.4470091E-02 AU
TTTTTTTTTTTTTTTTTTTTTTTTTTTTTT PDIG        TELAPSE      379.75 TCPU      375.79
TTTTTTTTTTTTTTTTTTTTTTTTTTTTTT MOQGAD      TELAPSE      379.78 TCPU      375.82
TTTTTTTTTTTTTTTTTTTTTTTTTTTTTT SHELLX      TELAPSE      382.80 TCPU      378.84
TTTTTTTTTTTTTTTTTTTTTTTTTTTTTT MONMO3      TELAPSE      383.16 TCPU      379.20
NUMERICALLY INTEGRATED DENSITY    344.0008558173
TTTTTTTTTTTTTTTTTTTTTTTTTTTTTT NUMDFT      TELAPSE      384.03 TCPU      380.07
CYC  21 ETOT(AU) -7.757783385150E+03 DETOT  6.87E-09 tst  1.24E-09 PX  8.24E-06
TTTTTTTTTTTTTTTTTTTTTTTTTTTTTT FDIK        TELAPSE      392.43 TCPU      388.47
INSULATING STATE - LEVEL SHIFTER    0.60 au
TOP OF VALENCE BANDS -    BAND    172; K    1; EIG -7.4639892E-01 AU
BOTTOM OF VIRTUAL BANDS - BAND    173; K    1; EIG  3.4469811E-02 AU
TTTTTTTTTTTTTTTTTTTTTTTTTTTTTT PDIG        TELAPSE      393.49 TCPU      389.53
TTTTTTTTTTTTTTTTTTTTTTTTTTTTTT MOQGAD      TELAPSE      393.51 TCPU      389.55
TTTTTTTTTTTTTTTTTTTTTTTTTTTTTT SHELLX      TELAPSE      396.54 TCPU      392.58
TTTTTTTTTTTTTTTTTTTTTTTTTTTTTT MONMO3      TELAPSE      396.89 TCPU      392.93
NUMERICALLY INTEGRATED DENSITY    344.0008558168
TTTTTTTTTTTTTTTTTTTTTTTTTTTTTT NUMDFT      TELAPSE      397.76 TCPU      393.80
CYC  22 ETOT(AU) -7.757783385146E+03 DETOT  4.00E-09 tst  6.99E-10 PX  5.56E-06
TTTTTTTTTTTTTTTTTTTTTTTTTTTTTT FDIK        TELAPSE      405.75 TCPU      401.79
INSULATING STATE - LEVEL SHIFTER    0.60 au
TOP OF VALENCE BANDS -    BAND    172; K    1; EIG -7.4639924E-01 AU
BOTTOM OF VIRTUAL BANDS - BAND    173; K    1; EIG  3.4469628E-02 AU
TTTTTTTTTTTTTTTTTTTTTTTTTTTTTT PDIG        TELAPSE      406.81 TCPU      402.85
TTTTTTTTTTTTTTTTTTTTTTTTTTTTTT MOQGAD      TELAPSE      406.83 TCPU      402.88
TTTTTTTTTTTTTTTTTTTTTTTTTTTTTT SHELLX      TELAPSE      409.86 TCPU      405.90
TTTTTTTTTTTTTTTTTTTTTTTTTTTTTT MONMO3      TELAPSE      410.21 TCPU      406.25
NUMERICALLY INTEGRATED DENSITY    344.0008558164
TTTTTTTTTTTTTTTTTTTTTTTTTTTTTT NUMDFT      TELAPSE      411.09 TCPU      407.13
CYC  23 ETOT(AU) -7.757783385144E+03 DETOT  2.82E-09 tst  3.95E-10 PX  3.79E-06
TTTTTTTTTTTTTTTTTTTTTTTTTTTTTT FDIK        TELAPSE      419.17 TCPU      415.22
INSULATING STATE - LEVEL SHIFTER    0.60 au
TOP OF VALENCE BANDS -    BAND    172; K    1; EIG -7.4639946E-01 AU
BOTTOM OF VIRTUAL BANDS - BAND    173; K    1; EIG  3.4469504E-02 AU
TTTTTTTTTTTTTTTTTTTTTTTTTTTTTT PDIG        TELAPSE      420.23 TCPU      416.27
TTTTTTTTTTTTTTTTTTTTTTTTTTTTTT MOQGAD      TELAPSE      420.26 TCPU      416.30
TTTTTTTTTTTTTTTTTTTTTTTTTTTTTT SHELLX      TELAPSE      423.28 TCPU      419.32
TTTTTTTTTTTTTTTTTTTTTTTTTTTTTT MONMO3      TELAPSE      423.63 TCPU      419.67
NUMERICALLY INTEGRATED DENSITY    344.0008558162
TTTTTTTTTTTTTTTTTTTTTTTTTTTTTT NUMDFT      TELAPSE      424.50 TCPU      420.54
CYC  24 ETOT(AU) -7.757783385142E+03 DETOT  1.76E-09 tst  2.24E-10 PX  2.59E-06
TTTTTTTTTTTTTTTTTTTTTTTTTTTTTT FDIK        TELAPSE      430.97 TCPU      427.01
INSULATING STATE - LEVEL SHIFTER    0.60 au
TOP OF VALENCE BANDS -    BAND    172; K    1; EIG -7.4639961E-01 AU
BOTTOM OF VIRTUAL BANDS - BAND    173; K    1; EIG  3.4469421E-02 AU
TTTTTTTTTTTTTTTTTTTTTTTTTTTTTT PDIG        TELAPSE      432.06 TCPU      428.10
TTTTTTTTTTTTTTTTTTTTTTTTTTTTTT MOQGAD      TELAPSE      432.09 TCPU      428.13
TTTTTTTTTTTTTTTTTTTTTTTTTTTTTT SHELLX      TELAPSE      435.14 TCPU      431.18
TTTTTTTTTTTTTTTTTTTTTTTTTTTTTT MONMO3      TELAPSE      435.49 TCPU      431.53
NUMERICALLY INTEGRATED DENSITY    344.0008558161
TTTTTTTTTTTTTTTTTTTTTTTTTTTTTT NUMDFT      TELAPSE      436.36 TCPU      432.40
CYC  25 ETOT(AU) -7.757783385141E+03 DETOT  1.21E-09 tst  1.27E-10 PX  1.76E-06
TTTTTTTTTTTTTTTTTTTTTTTTTTTTTT FDIK        TELAPSE      442.40 TCPU      438.44
INSULATING STATE - LEVEL SHIFTER    0.60 au
TOP OF VALENCE BANDS -    BAND    172; K    1; EIG -7.4639972E-01 AU
BOTTOM OF VIRTUAL BANDS - BAND    173; K    1; EIG  3.4469363E-02 AU
TTTTTTTTTTTTTTTTTTTTTTTTTTTTTT PDIG        TELAPSE      443.45 TCPU      439.49
TTTTTTTTTTTTTTTTTTTTTTTTTTTTTT MOQGAD      TELAPSE      443.48 TCPU      439.52
TTTTTTTTTTTTTTTTTTTTTTTTTTTTTT SHELLX      TELAPSE      446.50 TCPU      442.54
TTTTTTTTTTTTTTTTTTTTTTTTTTTTTT MONMO3      TELAPSE      446.86 TCPU      442.90
NUMERICALLY INTEGRATED DENSITY    344.0008558160
TTTTTTTTTTTTTTTTTTTTTTTTTTTTTT NUMDFT      TELAPSE      447.73 TCPU      443.77
CYC  26 ETOT(AU) -7.757783385140E+03 DETOT  8.89E-10 tst  7.23E-11 PX  1.20E-06
TTTTTTTTTTTTTTTTTTTTTTTTTTTTTT FDIK        TELAPSE      454.16 TCPU      450.20
INSULATING STATE - LEVEL SHIFTER    0.60 au
TOP OF VALENCE BANDS -    BAND    172; K    1; EIG -7.4639980E-01 AU
BOTTOM OF VIRTUAL BANDS - BAND    173; K    1; EIG  3.4469325E-02 AU
TTTTTTTTTTTTTTTTTTTTTTTTTTTTTT PDIG        TELAPSE      455.22 TCPU      451.25
TTTTTTTTTTTTTTTTTTTTTTTTTTTTTT MOQGAD      TELAPSE      455.24 TCPU      451.28
TTTTTTTTTTTTTTTTTTTTTTTTTTTTTT SHELLX      TELAPSE      458.26 TCPU      454.30
TTTTTTTTTTTTTTTTTTTTTTTTTTTTTT MONMO3      TELAPSE      458.62 TCPU      454.66
NUMERICALLY INTEGRATED DENSITY    344.0008558159
```

```
TTTTTTTTTTTTTTTTTTTTTTTTTTTTTT NUMDFT      TELAPSE      459.50 TCPU      455.54
CYC  27 ETOT(AU) -7.757783385139E+03 DETOT  4.82E-10 tst  4.11E-11 PX  8.15E-07
TTTTTTTTTTTTTTTTTTTTTTTTTTTTTT FDIK        TELAPSE      465.84 TCPU      461.88
INSULATING STATE - LEVEL SHIFTER    0.60 au
TOP OF VALENCE BANDS -    BAND    172; K    1; EIG -7.4639984E-01 AU
BOTTOM OF VIRTUAL BANDS - BAND    173; K    1; EIG  3.4469305E-02 AU
TTTTTTTTTTTTTTTTTTTTTTTTTTTTTT PDIG        TELAPSE      466.89 TCPU      462.93
TTTTTTTTTTTTTTTTTTTTTTTTTTTTTT MOQGAD      TELAPSE      466.92 TCPU      462.96
TTTTTTTTTTTTTTTTTTTTTTTTTTTTTT SHELLX      TELAPSE      469.94 TCPU      465.98
TTTTTTTTTTTTTTTTTTTTTTTTTTTTTT MONMO3      TELAPSE      470.30 TCPU      466.34
NUMERICALLY INTEGRATED DENSITY    344.0008558158
TTTTTTTTTTTTTTTTTTTTTTTTTTTTTT NUMDFT      TELAPSE      471.17 TCPU      467.21
CYC  28 ETOT(AU) -7.757783385139E+03 DETOT  2.37E-10 tst  2.34E-11 PX  5.70E-07
TTTTTTTTTTTTTTTTTTTTTTTTTTTTTT FDIK        TELAPSE      477.18 TCPU      473.22
INSULATING STATE - LEVEL SHIFTER    0.60 au
TOP OF VALENCE BANDS -    BAND    172; K    1; EIG -7.4639987E-01 AU
BOTTOM OF VIRTUAL BANDS - BAND    173; K    1; EIG  3.4469289E-02 AU
TTTTTTTTTTTTTTTTTTTTTTTTTTTTTT PDIG        TELAPSE      478.25 TCPU      474.29
TTTTTTTTTTTTTTTTTTTTTTTTTTTTTT MOQGAD      TELAPSE      478.28 TCPU      474.32
TTTTTTTTTTTTTTTTTTTTTTTTTTTTTT SHELLX      TELAPSE      481.32 TCPU      477.36
TTTTTTTTTTTTTTTTTTTTTTTTTTTTTT MONMO3      TELAPSE      481.67 TCPU      477.71
NUMERICALLY INTEGRATED DENSITY    344.0008558158
TTTTTTTTTTTTTTTTTTTTTTTTTTTTTT NUMDFT      TELAPSE      482.56 TCPU      478.60
CYC  29 ETOT(AU) -7.757783385139E+03 DETOT  2.29E-10 tst  1.34E-11 PX  3.92E-07
TTTTTTTTTTTTTTTTTTTTTTTTTTTTTT FDIK        TELAPSE      488.50 TCPU      484.54
INSULATING STATE - LEVEL SHIFTER    0.60 au
TOP OF VALENCE BANDS -    BAND    172; K    1; EIG -7.4639990E-01 AU
BOTTOM OF VIRTUAL BANDS - BAND    173; K    1; EIG  3.4469275E-02 AU
TTTTTTTTTTTTTTTTTTTTTTTTTTTTTT PDIG        TELAPSE      489.61 TCPU      485.65
TTTTTTTTTTTTTTTTTTTTTTTTTTTTTT MOQGAD      TELAPSE      489.64 TCPU      485.68
TTTTTTTTTTTTTTTTTTTTTTTTTTTTTT SHELLX      TELAPSE      492.69 TCPU      488.73
TTTTTTTTTTTTTTTTTTTTTTTTTTTTTT MONMO3      TELAPSE      493.03 TCPU      489.07
NUMERICALLY INTEGRATED DENSITY    344.0008558158
TTTTTTTTTTTTTTTTTTTTTTTTTTTTTT NUMDFT      TELAPSE      493.92 TCPU      489.96
CYC  30 ETOT(AU) -7.757783385139E+03 DETOT  1.45E-10 tst  7.60E-12 PX  2.64E-07
TTTTTTTTTTTTTTTTTTTTTTTTTTTTTT FDIK        TELAPSE      500.25 TCPU      496.29
INSULATING STATE - LEVEL SHIFTER    0.60 au
TOP OF VALENCE BANDS -    BAND    172; K    1; EIG -7.4639992E-01 AU
BOTTOM OF VIRTUAL BANDS - BAND    173; K    1; EIG  3.4469266E-02 AU
TTTTTTTTTTTTTTTTTTTTTTTTTTTTTT PDIG        TELAPSE      501.31 TCPU      497.35
TTTTTTTTTTTTTTTTTTTTTTTTTTTTTT MOQGAD      TELAPSE      501.33 TCPU      497.38
TTTTTTTTTTTTTTTTTTTTTTTTTTTTTT SHELLX      TELAPSE      504.37 TCPU      500.41
TTTTTTTTTTTTTTTTTTTTTTTTTTTTTT MONMO3      TELAPSE      504.72 TCPU      500.76
NUMERICALLY INTEGRATED DENSITY    344.0008558158
TTTTTTTTTTTTTTTTTTTTTTTTTTTTTT NUMDFT      TELAPSE      505.59 TCPU      501.63
CYC  31 ETOT(AU) -7.757783385139E+03 DETOT  1.23E-10 tst  4.33E-12 PX  1.80E-07
TTTTTTTTTTTTTTTTTTTTTTTTTTTTTT FDIK        TELAPSE      511.40 TCPU      507.44
INSULATING STATE - LEVEL SHIFTER    0.60 au
TOP OF VALENCE BANDS -    BAND    172; K    1; EIG -7.4639993E-01 AU
BOTTOM OF VIRTUAL BANDS - BAND    173; K    1; EIG  3.4469262E-02 AU
TTTTTTTTTTTTTTTTTTTTTTTTTTTTTT PDIG        TELAPSE      512.46 TCPU      508.50
TTTTTTTTTTTTTTTTTTTTTTTTTTTTTT MOQGAD      TELAPSE      512.48 TCPU      508.52
TTTTTTTTTTTTTTTTTTTTTTTTTTTTTT SHELLX      TELAPSE      515.51 TCPU      511.55
TTTTTTTTTTTTTTTTTTTTTTTTTTTTTT MONMO3      TELAPSE      515.86 TCPU      511.90
NUMERICALLY INTEGRATED DENSITY    344.0008558157
TTTTTTTTTTTTTTTTTTTTTTTTTTTTTT NUMDFT      TELAPSE      516.73 TCPU      512.77
CYC  32 ETOT(AU) -7.757783385138E+03 DETOT  7.28E-11 tst  2.47E-12 PX  1.29E-07
TTTTTTTTTTTTTTTTTTTTTTTTTTTTTT FDIK        TELAPSE      522.35 TCPU      518.39
INSULATING STATE - LEVEL SHIFTER    0.60 au
TOP OF VALENCE BANDS -    BAND    172; K    1; EIG -7.4639993E-01 AU
BOTTOM OF VIRTUAL BANDS - BAND    173; K    1; EIG  3.4469260E-02 AU
TTTTTTTTTTTTTTTTTTTTTTTTTTTTTT PDIG        TELAPSE      523.41 TCPU      519.45
TTTTTTTTTTTTTTTTTTTTTTTTTTTTTT MOQGAD      TELAPSE      523.43 TCPU      519.47
TTTTTTTTTTTTTTTTTTTTTTTTTTTTTT SHELLX      TELAPSE      526.47 TCPU      522.51
::: PSEUDO TOTAL   ENERGY                        -7.2729486318120E+03
::: VIRIAL COEFFICIENT                            1.0321311011979E+00
TTTTTTTTTTTTTTTTTTTTTTTTTTTTTT MONMO3      TELAPSE      526.82 TCPU      522.86
NUMERICALLY INTEGRATED DENSITY    344.0008558157
TTTTTTTTTTTTTTTTTTTTTTTTTTTTTT NUMDFT      TELAPSE      527.68 TCPU      523.72
CYC  33 ETOT(AU) -7.757783385138E+03 DETOT  4.37E-11 tst  1.41E-12 PX  1.29E-07

== SCF ENDED - CONVERGENCE ON ENERGY      E(AU) -7.7577833851384E+03 CYCLES  33


ENERGY EXPRESSION=HARTREE+FOCK EXCH*0.00000+(LDA    EXCH)*1.00000+VWN    CORR


TOTAL ENERGY(DFT)(AU)( 33) -7.7577833851384E+03 DE 4.4E-11 tester 1.4E-12
TTTTTTTTTTTTTTTTTTTTTTTTTTTTTT EDFT        TELAPSE      527.69 TCPU      523.73
```

```
*******************************************************************************
*                              FORCE CALCULATION                              *
*******************************************************************************

TTTTTTTTTTTTTTTTTTTTTTTTTTTTTT NUMDFG      TELAPSE      563.42 TCPU      559.46
INFORMATION **** EXCPOG **** EXCH. BIPO BUFFER LENGTH (WORDS) =          0
WARNING **** GENPOG **** COULOMB BIPO BUFFER TOO SMALL - TO AVOID I/O SET BIPOSIZE =     1208000
INFORMATION **** GENPOG **** BIPO BUFFER LENGTH (WORDS) =     1000000
TTTTTTTTTTTTTTTTTTTTTTTTTTTTTT SHELXG      TELAPSE      579.90 TCPU      575.94

CARTESIAN FORCES IN HARTREE/BOHR (ANALYTICAL)
  ATOM                     X                   Y                   Z
   1  20           2.005954584314E-04 -1.903282468074E-06  2.594509983762E-04
   2  20          -2.005954583939E-04 -1.903282412563E-06 -2.594509984171E-04
   3  20          -2.005954584212E-04  1.903282437099E-06 -2.594509984455E-04
   4  20           2.005954583895E-04  1.903282414895E-06  2.594509984699E-04
   5  20           1.171798142803E-04 -1.108867630987E-04 -1.046996983036E-04
   6  20          -1.171798142949E-04 -1.108867631183E-04  1.046996982534E-04
   7  20          -1.171798142772E-04  1.108867631160E-04  1.046996983134E-04
   8  20           1.171798142874E-04  1.108867631282E-04 -1.046996982421E-04
   9  14           4.255192638869E-05 -1.552706514781E-04 -7.894493773764E-05
  10  14          -4.255192639668E-05 -1.552706515269E-04  7.894493767235E-05
  11  14          -4.255192640401E-05  1.552706515020E-04  7.894493766836E-05
  12  14           4.255192637492E-05  1.552706515039E-04 -7.894493763949E-05
  13   8          -1.116428598608E-04  6.199391342765E-05  1.346451790902E-04
  14   8           1.116428598759E-04  6.199391342054E-05 -1.346451791135E-04
  15   8           1.116428598620E-04 -6.199391342321E-05 -1.346451791369E-04
  16   8          -1.116428598675E-04 -6.199391339390E-05  1.346451791342E-04
  17   8          -1.543717094643E-05 -5.515118809329E-05  4.719253771945E-05
  18   8           1.543717095043E-05 -5.515118811683E-05 -4.719253774432E-05
  19   8           1.543717094510E-05  5.515118810662E-05 -4.719253773722E-05
  20   8          -1.543717094599E-05  5.515118810706E-05  4.719253776564E-05
  21   8           1.389047716041E-04  4.441944020783E-05 -7.249625152372E-05
  22   8          -1.389047715916E-04  4.441944020273E-05  7.249625150374E-05
  23   8          -1.389047715983E-04 -4.441944021139E-05  7.249625150152E-05
  24   8           1.389047715952E-04 -4.441944020761E-05 -7.249625148109E-05
  25   8          -3.131411372828E-04 -5.522204511443E-06 -1.489384940712E-05
  26   8           3.131411373072E-04 -5.522204492348E-06  1.489384945419E-05
  27   8           3.131411372888E-04  5.522204482133E-06  1.489384941422E-05
  28   8          -3.131411373003E-04  5.522204498121E-06 -1.489384941245E-05

RESULTANT FORCE    -8.604228440845E-16  2.137179322403E-15 -4.884981308351E-15

SYMMETRY ALLOWED FORCES (ANALYTICAL) (DIRECTION, FORCE)

    1  4.0119092E-04    2 -3.8065649E-06    3  5.1890200E-04    4  2.3435963E-04
    5 -2.2177353E-04    6 -2.0939940E-04    7  8.5103853E-05    8 -3.1054130E-04
    9 -1.5788988E-04   10 -2.2328572E-04   11  1.2398783E-04   12  2.6929036E-04
   13 -3.0874342E-05   14 -1.1030238E-04   15  9.4385075E-05   16  2.7780954E-04
   17  8.8838880E-05   18 -1.4499250E-04   19 -6.2628227E-04   20 -1.1044409E-05
   21 -2.9787699E-05
TTTTTTTTTTTTTTTTTTTTTTTTTTTTTT TOTGRA      TELAPSE      581.50 TCPU      577.54

HHHHHHHHHHHHHHHHHHHHHHHHHHHHHHHHHHHHHHHHHHHHHHHHHHHHHHHHHHHHHHHHHHHHHHHHHHHHHHH
                  FORCE CONSTANT MATRIX - NUMERICAL ESTIMATE
HHHHHHHHHHHHHHHHHHHHHHHHHHHHHHHHHHHHHHHHHHHHHHHHHHHHHHHHHHHHHHHHHHHHHHHHHHHHHHH
MAX ABS(DGRAD): MAXIMUM ABSOLUTE GRADIENT DIFFERENCE WITH RESPECT TO
                THE CENTRAL POINT
DE:             ENERGY DIFFERENCE WITH RESPECT TO THE CENTRAL POINT
                (DE IS EXPECTED TO BE POSITIVE FOR ALL DISPLACEMENTS)

  ATOM      MAX ABS(DGRAD)       TOTAL ENERGY (AU)  N.CYC      DE       SYM
   CENTRAL POINT            -7.757783385138E+03    33     0.0000E+00    4
    1 CA DX   4.2462E-04      -7.757783385034E+03    13     1.0471E-07    1
    1 CA DY   5.7689E-04      -7.757783383544E+03     8     1.5948E-06    1
    1 CA DZ   3.8668E-04      -7.757783385603E+03     8    -4.6483E-07    1
    5 CA DX   3.4910E-04      -7.757783384908E+03     8     2.3015E-07    1
    5 CA DY   4.5805E-04      -7.757783382768E+03     9     2.3706E-06    1
    5 CA DZ   5.4965E-04      -7.757783382723E+03    12     2.4151E-06    1
    9 SI DX   2.4478E-03      -7.757783379046E+03    16     6.0923E-06    1
    9 SI DY   2.9249E-03      -7.757783374505E+03    14     1.0633E-05    1
    9 SI DZ   2.4828E-03      -7.757783376575E+03    13     8.5639E-06    1
   13 O  DX   3.7220E-04      -7.757783383004E+03    15     2.1343E-06    1
   13 O  DY   2.1762E-03      -7.757783379306E+03    13     5.8320E-06    1
   13 O  DZ   4.2724E-04      -7.757783385078E+03    11     6.0474E-08    1
   17 O  DX   1.3355E-03      -7.757783380390E+03    15     4.7485E-06    1
   17 O  DY   6.3890E-04      -7.757783382741E+03    15     2.3976E-06    1
   17 O  DZ   6.4228E-04      -7.757783383829E+03    11     1.3090E-06    1
```

```
  21 O  DX   1.2080E-03      -7.757783382248E+03    13     2.8906E-06    1
  21 O  DY   4.6578E-04      -7.757783384992E+03    14     1.4609E-07    1
  21 O  DZ   1.0063E-03      -7.757783381243E+03    14     3.8950E-06    1
  25 O  DX   5.4692E-04      -7.757783381648E+03    13     3.4899E-06    1
  25 O  DY   6.1902E-04      -7.757783383464E+03    14     1.6739E-06    1
  25 O  DZ   1.4045E-03      -7.757783381608E+03    14     3.5302E-06    1

GCALCO - MAX INDICES DIRECT LATTICE VECTOR    19    15    11
NO.OF VECTORS CREATED 6999 STARS 1862 RMAX   157.41387 BOHR

CAPPA:IS1  6;IS2  6;IS3  6; K PTS MONK NET   80; SYMMOPS: K SPACE  4;G SPACE  4

TTTTTTTTTTTTTTTTTTTTTTTTTTTTTT gordsh1     TELAPSE    17231.85 TCPU    17095.91

DIMENSIONS  P(G)=  389912 F(G)=  195388 P(G),F(G) (IRR)   49967
MAX G-VECTOR INDEX FOR 1- AND 2-ELECTRON INTEGRALS  33

TTTTTTTTTTTTTTTTTTTTTTTTTTTTTT INPUT       TELAPSE    17231.85 TCPU    17095.91

SYMMETRY ALLOWED INTERNAL DEGREE(S) OF FREEDOM:  21
TTTTTTTTTTTTTTTTTTTTTTTTTTTTTT SYMM        TELAPSE    17232.08 TCPU    17096.14
TTTTTTTTTTTTTTTTTTTTTTTTTTTTTT INT_SCREEN  TELAPSE    17232.08 TCPU    17096.14

+++ SYMMETRY ADAPTION OF VIBRATIONAL MODES +++

SYMMETRY INFORMATION:
K-LITTLE GROUP: CLASS TABLE, CHARACTER TABLE.
IRREP-(DIMENSION, NO. IRREDUCIBLE SETS)
[WARNINGS: (1) ONLY ACTIVE IRREPS ARE GENERATED AND LISTED.
           (2) ONLY RELEVANT CLASSES ARE CONSIDERED IN THE CHARACTER TABLE
           (3) SYMBOLS MAY NOT FULLY COINCIDE WITH THOSE FROM TEXT BOOKS.
IN CLASSES WRITTEN AS CX.Y OR SX.Y, X.Y IS A DECIMAL NUMBER THAT
APPROACHES THE OPERATION FRACTIONAL ORDER.]

(P, D, RP, RD, STAND FOR PAIRING, DOUBLING, REAL PAIRING AND REAL DOUBLING
OF THE IRREPS (SEE MANUAL))

K[   1] (  0  0  0)

CLASS | GROUP OPERATORS (SEE SYMMOPS KEYWORD)
-----------------------------------------------------------------------
C2    |  2;
I     |  3;
SGH   |  4;

IRREP/CLA   E    C2    I     SGH
--------------------------------
   AG  |  1.00  1.00  1.00  1.00
   BG  |  1.00 -1.00  1.00 -1.00
   AU  |  1.00  1.00 -1.00 -1.00
   BU  |  1.00 -1.00 -1.00  1.00

AG -(1,  21); BG -(1,  21);
AU -(1,  21); BU -(1,  21);

HHHHHHHHHHHHHHHHHHHHHHHHHHHHHHHHHHHHHHHHHHHHHHHHHHHHHHHHHHHHHHHHHHHHHHHHHHHHHHHH
EIGENVALUES (EIGV) OF THE MASS WEIGHTED HESSIAN MATRIX AND HARMONIC
FREQUENCIES. IRREP LABELS REFER TO SYMMETRY REPRESENTATION ANALYSIS;  A AND I
INDICATE WHETHER THE MODE IS ACTIVE OR INACTIVE, RESPECTIVELY, FOR IR AND
RAMAN; IR INTENSITIES IN BRACKETS.

CONVERSION FACTORS FOR FREQUENCIES:
     1 CM**(-1) =   0.2194746E+06 HARTREE
     1 THZ      =   0.2997925E-01 CM**(-1)

HHHHHHHHHHHHHHHHHHHHHHHHHHHHHHHHHHHHHHHHHHHHHHHHHHHHHHHHHHHHHHHHHHHHHHHHHHHHHHHH

   MODES         EIGV          FREQUENCIES     IRREP  IR   INTENS    RAMAN
             (HARTREE**2)   (CM**-1)     (THZ)             (KM/MOL)
   1-   1   -0.8150E-10     -1.9813   -0.0594  (AU )   A (     0.00)   I
   2-   2   -0.6489E-10     -1.7680   -0.0530  (BU )   A (     0.00)   I
   3-   3   -0.5362E-10     -1.6071   -0.0482  (BU )   A (     0.00)   I
   4-   4    0.2140E-06    101.5304    3.0438  (AU )   A (     0.00)   I
   5-   5    0.2327E-06    105.8656    3.1738  (BU )   A (     0.00)   I
   6-   6    0.2376E-06    106.9805    3.2072  (BG )   I (     0.00)   A
   7-   7    0.2623E-06    112.4017    3.3697  (AG )   I (     0.00)   A
   8-   8    0.3153E-06    123.2323    3.6944  (AU )   A (     0.00)   I
   9-   9    0.4100E-06    140.5407    4.2133  (AG )   I (     0.00)   A
  10-  10    0.4271E-06    143.4382    4.3002  (AU )   A (     0.00)   I
```

```
  11-  11    0.4546E-06    147.9712    4.4361  (BU )   A (     0.00)   I
  12-  12    0.4852E-06    152.8855    4.5834  (AG )   I (     0.00)   A
  13-  13    0.4887E-06    153.4235    4.5995  (BG )   I (     0.00)   A
  14-  14    0.5321E-06    160.1032    4.7998  (BG )   I (     0.00)   A
  15-  15    0.5643E-06    164.8685    4.9426  (AG )   I (     0.00)   A
  16-  16    0.6166E-06    172.3387    5.1666  (BG )   I (     0.00)   A
  17-  17    0.6188E-06    172.6450    5.1758  (BU )   A (     0.00)   I
  18-  18    0.6422E-06    175.8747    5.2726  (AG )   I (     0.00)   A
  19-  19    0.6679E-06    179.3651    5.3772  (AU )   A (     0.00)   I
  20-  20    0.7169E-06    185.8291    5.5710  (AU )   A (     0.00)   I
  21-  21    0.8103E-06    197.5586    5.9227  (AG )   I (     0.00)   A
  22-  22    0.8169E-06    198.3720    5.9470  (BG )   I (     0.00)   A
  23-  23    0.8251E-06    199.3636    5.9768  (BU )   A (     0.00)   I
  24-  24    0.8605E-06    203.5862    6.1034  (AG )   I (     0.00)   A
  25-  25    0.9472E-06    213.6074    6.4038  (BU )   A (     0.00)   I
  26-  26    0.9812E-06    217.4072    6.5177  (BG )   I (     0.00)   A
  27-  27    0.9937E-06    218.7831    6.5590  (AU )   A (     0.00)   I
  28-  28    0.1059E-05    225.8044    6.7694  (AU )   A (     0.00)   I
  29-  29    0.1100E-05    230.1363    6.8993  (BU )   A (     0.00)   I
  30-  30    0.1198E-05    240.2116    7.2014  (AG )   I (     0.00)   A
  31-  31    0.1277E-05    247.9782    7.4342  (BG )   I (     0.00)   A
  32-  32    0.1313E-05    251.4795    7.5392  (AG )   I (     0.00)   A
  33-  33    0.1322E-05    252.3780    7.5661  (BU )   A (     0.00)   I
  34-  34    0.1366E-05    256.5462    7.6911  (BG )   I (     0.00)   A
  35-  35    0.1375E-05    257.3969    7.7166  (AU )   A (     0.00)   I
  36-  36    0.1454E-05    264.6186    7.9331  (BU )   A (     0.00)   I
  37-  37    0.1468E-05    265.8781    7.9708  (AG )   I (     0.00)   A
  38-  38    0.1531E-05    271.6048    8.1425  (AU )   A (     0.00)   I
  39-  39    0.1554E-05    273.6134    8.2027  (BG )   I (     0.00)   A
  40-  40    0.1568E-05    274.8033    8.2384  (AU )   A (     0.00)   I
  41-  41    0.1608E-05    278.3316    8.3442  (AG )   I (     0.00)   A
  42-  42    0.1699E-05    286.0507    8.5756  (BG )   I (     0.00)   A
  43-  43    0.1699E-05    286.0778    8.5764  (BU )   A (     0.00)   I
  44-  44    0.1786E-05    293.3028    8.7930  (BG )   I (     0.00)   A
  45-  45    0.1837E-05    297.4590    8.9176  (AU )   A (     0.00)   I
  46-  46    0.1907E-05    303.0856    9.0863  (AG )   I (     0.00)   A
  47-  47    0.2020E-05    311.9444    9.3519  (BG )   I (     0.00)   A
  48-  48    0.2046E-05    313.9130    9.4109  (BU )   A (     0.00)   I
  49-  49    0.2625E-05    355.6024   10.6607  (AG )   I (     0.00)   A
  50-  50    0.2707E-05    361.1312   10.8264  (BU )   A (     0.00)   I
  51-  51    0.2848E-05    370.4070   11.1045  (AU )   A (     0.00)   I
  52-  52    0.3001E-05    380.2340   11.3991  (AU )   A (     0.00)   I
  53-  53    0.3340E-05    401.1185   12.0252  (BG )   I (     0.00)   A
  54-  54    0.3475E-05    409.1040   12.2646  (AG )   I (     0.00)   A
  55-  55    0.3528E-05    412.2252   12.3582  (BG )   I (     0.00)   A
  56-  56    0.3763E-05    425.7683   12.7642  (BU )   A (     0.00)   I
  57-  57    0.4699E-05    475.7407   14.2623  (BU )   A (     0.00)   I
  58-  58    0.4761E-05    478.8918   14.3568  (AU )   A (     0.00)   I
  59-  59    0.5023E-05    491.8932   14.7466  (AG )   I (     0.00)   A
  60-  60    0.5112E-05    496.2041   14.8758  (BU )   A (     0.00)   I
  61-  61    0.5166E-05    498.8237   14.9544  (AU )   A (     0.00)   I
  62-  62    0.5301E-05    505.3093   15.1488  (BG )   I (     0.00)   A
  63-  63    0.5504E-05    514.8988   15.4363  (BG )   I (     0.00)   A
  64-  64    0.5565E-05    517.7312   15.5212  (AG )   I (     0.00)   A
  65-  65    0.5669E-05    522.5593   15.6659  (AU )   A (     0.00)   I
  66-  66    0.5687E-05    523.3729   15.6903  (BU )   A (     0.00)   I
  67-  67    0.5911E-05    533.5816   15.9964  (AG )   I (     0.00)   A
  68-  68    0.6163E-05    544.8323   16.3337  (BG )   I (     0.00)   A
  69-  69    0.1395E-04    819.7798   24.5764  (BU )   A (     0.00)   I
  70-  70    0.1403E-04    822.1795   24.6483  (AU )   A (     0.00)   I
  71-  71    0.1407E-04    823.2486   24.6804  (AG )   I (     0.00)   A
  72-  72    0.1439E-04    832.5324   24.9587  (BG )   I (     0.00)   A
  73-  73    0.1445E-04    834.3249   25.0124  (AG )   I (     0.00)   A
  74-  74    0.1474E-04    842.5880   25.2602  (BU )   A (     0.00)   I
  75-  75    0.1509E-04    852.7031   25.5634  (BG )   I (     0.00)   A
  76-  76    0.1535E-04    859.8794   25.7785  (AU )   A (     0.00)   I
  77-  77    0.1605E-04    879.2470   26.3592  (AG )   I (     0.00)   A
  78-  78    0.1610E-04    880.6863   26.4023  (BU )   A (     0.00)   I
  79-  79    0.1676E-04    898.5178   26.9369  (BG )   I (     0.00)   A
  80-  80    0.1808E-04    933.1644   27.9756  (AU )   A (     0.00)   I
  81-  81    0.1926E-04    963.1394   28.8742  (AU )   A (     0.00)   I
  82-  82    0.1926E-04    963.2878   28.8786  (AG )   I (     0.00)   A
  83-  83    0.1996E-04    980.4268   29.3925  (BU )   A (     0.00)   I
  84-  84    0.1996E-04    980.4542   29.3933  (BG )   I (     0.00)   A

NORMAL MODES NORMALIZED TO CLASSICAL AMPLITUDES

FREQ(CM**-1)      -1.98      -1.77      -1.61    101.53    105.87    106.98
```

AT. 1 CA	X	-0.0005	0.2114	0.2443	0.0415	0.0070	0.0112
	Y	0.2979	0.0007	0.0006	-0.0057	-0.0468	-0.0010
	Z	0.0015	0.2333	-0.2219	-0.0135	0.0019	0.0461
AT. 2 CA	X	0.0005	0.2114	0.2443	-0.0415	0.0070	0.0112
	Y	0.2979	-0.0007	-0.0006	-0.0057	0.0468	0.0010
	Z	-0.0015	0.2333	-0.2219	0.0135	0.0019	0.0461
AT. 3 CA	X	-0.0005	0.2114	0.2443	0.0415	0.0070	-0.0112
	Y	0.2979	0.0007	0.0006	-0.0057	-0.0468	0.0010
	Z	0.0015	0.2333	-0.2219	-0.0135	0.0019	-0.0461
AT. 4 CA	X	0.0005	0.2114	0.2443	-0.0415	0.0070	-0.0112
	Y	0.2979	-0.0007	-0.0006	-0.0057	0.0468	-0.0010
	Z	-0.0015	0.2333	-0.2219	0.0135	0.0019	-0.0461
AT. 5 CA	X	-0.0007	0.2107	0.2443	0.0152	0.0042	0.0430
	Y	0.2972	-0.0008	0.0000	0.0100	0.0006	-0.0039
	Z	-0.0005	0.2331	-0.2222	-0.0009	0.0017	-0.0107
AT. 6 CA	X	0.0007	0.2107	0.2443	-0.0152	0.0042	0.0430
	Y	0.2972	0.0008	-0.0000	0.0100	-0.0006	0.0039
	Z	0.0005	0.2331	-0.2222	0.0009	0.0017	-0.0107
AT. 7 CA	X	-0.0007	0.2107	0.2443	0.0152	0.0042	-0.0430
	Y	0.2972	-0.0008	0.0000	0.0100	0.0006	0.0039
	Z	-0.0005	0.2331	-0.2222	-0.0009	0.0017	0.0107
AT. 8 CA	X	0.0007	0.2107	0.2443	-0.0152	0.0042	-0.0430
	Y	0.2972	0.0008	-0.0000	0.0100	-0.0006	-0.0039
	Z	0.0005	0.2331	-0.2222	0.0009	0.0017	0.0107
AT. 9 SI	X	-0.0008	0.2114	0.2450	0.0380	-0.0018	0.0221
	Y	0.2963	0.0009	0.0010	-0.0010	-0.0402	-0.0013
	Z	-0.0003	0.2332	-0.2208	0.0057	-0.0005	0.0144
AT. 10 SI	X	0.0008	0.2114	0.2450	-0.0380	-0.0018	0.0221
	Y	0.2963	-0.0009	-0.0010	-0.0010	0.0402	0.0013
	Z	0.0003	0.2332	-0.2208	-0.0057	-0.0005	0.0144
AT. 11 SI	X	-0.0008	0.2114	0.2450	0.0380	-0.0018	-0.0221
	Y	0.2963	0.0009	0.0010	-0.0010	-0.0402	0.0013
	Z	-0.0003	0.2332	-0.2208	0.0057	-0.0005	-0.0144
AT. 12 SI	X	0.0008	0.2114	0.2450	-0.0380	-0.0018	-0.0221
	Y	0.2963	-0.0009	-0.0010	-0.0010	0.0402	-0.0013
	Z	0.0003	0.2332	-0.2208	-0.0057	-0.0005	-0.0144
AT. 13 O	X	-0.0040	0.2128	0.2463	0.0783	-0.0427	0.0047
	Y	0.2979	0.0007	0.0010	0.0048	-0.0440	-0.0058
	Z	0.0012	0.2317	-0.2196	0.0033	-0.0128	0.0141
AT. 14 O	X	0.0040	0.2128	0.2463	-0.0783	-0.0427	0.0047
	Y	0.2979	-0.0007	-0.0010	0.0048	0.0440	0.0058
	Z	-0.0012	0.2317	-0.2196	-0.0033	-0.0128	0.0141
AT. 15 O	X	-0.0040	0.2128	0.2463	0.0783	-0.0427	-0.0047
	Y	0.2979	0.0007	0.0010	0.0048	-0.0440	0.0058
	Z	0.0012	0.2317	-0.2196	0.0033	-0.0128	-0.0141
AT. 16 O	X	0.0040	0.2128	0.2463	-0.0783	-0.0427	-0.0047
	Y	0.2979	-0.0007	-0.0010	0.0048	0.0440	-0.0058
	Z	-0.0012	0.2317	-0.2196	-0.0033	-0.0128	-0.0141
AT. 17 O	X	-0.0017	0.2109	0.2436	0.0365	0.0089	0.0340
	Y	0.2969	-0.0007	0.0014	0.0129	-0.0634	0.0013
	Z	0.0006	0.2337	-0.2226	-0.0094	0.0087	-0.0193
AT. 18 O	X	0.0017	0.2109	0.2436	-0.0365	0.0089	0.0340
	Y	0.2969	0.0007	-0.0014	0.0129	0.0634	-0.0013
	Z	-0.0006	0.2337	-0.2226	0.0094	0.0087	-0.0193
AT. 19 O	X	-0.0017	0.2109	0.2436	0.0365	0.0089	-0.0340
	Y	0.2969	-0.0007	0.0014	0.0129	-0.0634	-0.0013
	Z	0.0006	0.2337	-0.2226	-0.0094	0.0087	0.0193
AT. 20 O	X	0.0017	0.2109	0.2436	-0.0365	0.0089	-0.0340
	Y	0.2969	0.0007	-0.0014	0.0129	0.0634	0.0013
	Z	-0.0006	0.2337	-0.2226	0.0094	0.0087	0.0193
AT. 21 O	X	-0.0014	0.2123	0.2440	0.0332	0.0009	0.0460
	Y	0.2978	-0.0005	0.0012	-0.0179	-0.0050	0.0028
	Z	-0.0002	0.2321	-0.2226	0.0027	-0.0055	0.0389
AT. 22 O	X	0.0014	0.2123	0.2440	-0.0332	0.0009	0.0460
	Y	0.2978	0.0005	-0.0012	-0.0179	0.0050	-0.0028
	Z	0.0002	0.2321	-0.2226	-0.0027	-0.0055	0.0389
AT. 23 O	X	-0.0014	0.2123	0.2440	0.0332	0.0009	-0.0460
	Y	0.2978	-0.0005	0.0012	-0.0179	-0.0050	-0.0028
	Z	-0.0002	0.2321	-0.2226	0.0027	-0.0055	-0.0389
AT. 24 O	X	0.0014	0.2123	0.2440	-0.0332	0.0009	-0.0460
	Y	0.2978	0.0005	-0.0012	-0.0179	0.0050	0.0028
	Z	0.0002	0.2321	-0.2226	-0.0027	-0.0055	-0.0389
AT. 25 O	X	-0.0003	0.2119	0.2439	0.0320	0.0092	-0.0072
	Y	0.2970	-0.0002	0.0007	-0.0067	-0.0326	-0.0112
	Z	0.0001	0.2340	-0.2228	0.0030	0.0010	0.0146
AT. 26 O	X	0.0003	0.2119	0.2439	-0.0320	0.0092	-0.0072
	Y	0.2970	0.0002	-0.0007	-0.0067	0.0326	0.0112
	Z	-0.0001	0.2340	-0.2228	-0.0030	0.0010	0.0146
AT. 27 O	X	-0.0003	0.2119	0.2439	0.0320	0.0092	0.0072

	Y	0.2970	-0.0002	0.0007	-0.0067	-0.0326	0.0112
	Z	0.0001	0.2340	-0.2228	0.0030	0.0010	-0.0146
AT. 28 O	X	0.0003	0.2119	0.2439	-0.0320	0.0092	0.0072
	Y	0.2970	0.0002	-0.0007	-0.0067	0.0326	-0.0112
	Z	-0.0001	0.2340	-0.2228	-0.0030	0.0010	-0.0146
FREQ(CM**-1)		112.40	123.23	140.54	143.44	147.97	152.89
AT. 1 CA	X	0.0100	0.0053	0.0041	0.0035	-0.0039	-0.0208
	Y	0.0112	0.0011	-0.0036	-0.0162	0.0147	0.0038
	Z	0.0271	0.0448	0.0062	-0.0146	-0.0031	0.0233
AT. 2 CA	X	-0.0100	-0.0053	-0.0041	-0.0035	-0.0039	0.0208
	Y	0.0112	0.0011	-0.0036	-0.0162	-0.0147	0.0038
	Z	-0.0271	-0.0448	-0.0062	0.0146	-0.0031	-0.0233
AT. 3 CA	X	-0.0100	0.0053	-0.0041	0.0035	-0.0039	0.0208
	Y	-0.0112	0.0011	0.0036	-0.0162	0.0147	-0.0038
	Z	-0.0271	0.0448	-0.0062	-0.0146	-0.0031	-0.0233
AT. 4 CA	X	0.0100	-0.0053	0.0041	-0.0035	-0.0039	-0.0208
	Y	-0.0112	0.0011	0.0036	-0.0162	-0.0147	-0.0038
	Z	0.0271	-0.0448	0.0062	0.0146	-0.0031	0.0233
AT. 5 CA	X	0.0071	0.0223	0.0380	0.0318	0.0217	-0.0389
	Y	0.0163	0.0028	0.0563	0.0422	0.0044	0.0141
	Z	-0.0007	0.0004	-0.0120	-0.0223	-0.0040	-0.0130
AT. 6 CA	X	-0.0071	-0.0223	-0.0380	-0.0318	0.0217	0.0389
	Y	0.0163	0.0028	0.0563	0.0422	-0.0044	0.0141
	Z	0.0007	-0.0004	0.0120	0.0223	-0.0040	0.0130
AT. 7 CA	X	-0.0071	0.0223	-0.0380	0.0318	0.0217	0.0389
	Y	-0.0163	0.0028	-0.0563	0.0422	0.0044	-0.0141
	Z	0.0007	0.0004	0.0120	-0.0223	-0.0040	0.0130
AT. 8 CA	X	0.0071	-0.0223	0.0380	-0.0318	0.0217	-0.0389
	Y	-0.0163	0.0028	-0.0563	0.0422	-0.0044	-0.0141
	Z	-0.0007	-0.0004	-0.0120	0.0223	-0.0040	-0.0130
AT. 9 SI	X	-0.0066	-0.0066	0.0009	-0.0066	-0.0017	0.0178
	Y	0.0132	-0.0016	-0.0017	-0.0086	0.0127	-0.0025
	Z	-0.0222	-0.0043	-0.0029	0.0100	0.0016	-0.0161
AT. 10 SI	X	0.0066	0.0066	-0.0009	0.0066	-0.0017	-0.0178
	Y	0.0132	-0.0016	-0.0017	-0.0086	-0.0127	-0.0025
	Z	0.0222	0.0043	0.0029	-0.0100	0.0016	0.0161
AT. 11 SI	X	0.0066	-0.0066	-0.0009	-0.0066	-0.0017	-0.0178
	Y	-0.0132	-0.0016	0.0017	-0.0086	0.0127	0.0025
	Z	0.0222	-0.0043	0.0029	0.0100	0.0016	0.0161
AT. 12 SI	X	-0.0066	0.0066	0.0009	0.0066	-0.0017	0.0178
	Y	-0.0132	-0.0016	0.0017	-0.0086	-0.0127	0.0025
	Z	-0.0222	0.0043	-0.0029	-0.0100	0.0016	-0.0161
AT. 13 O	X	0.0241	0.0296	-0.0042	-0.0381	-0.0528	-0.0191
	Y	0.0104	-0.0010	-0.0018	-0.0141	0.0091	-0.0068
	Z	0.0308	0.0369	-0.0090	0.0134	-0.0369	-0.0218
AT. 14 O	X	-0.0241	-0.0296	0.0042	0.0381	-0.0528	0.0191
	Y	0.0104	-0.0010	-0.0018	-0.0141	-0.0091	-0.0068
	Z	-0.0308	-0.0369	0.0090	-0.0134	-0.0369	0.0218
AT. 15 O	X	-0.0241	0.0296	0.0042	-0.0381	-0.0528	0.0191
	Y	-0.0104	-0.0010	0.0018	-0.0141	0.0091	0.0068
	Z	-0.0308	0.0369	0.0090	0.0134	-0.0369	0.0218
AT. 16 O	X	0.0241	-0.0296	-0.0042	0.0381	-0.0528	-0.0191
	Y	-0.0104	-0.0010	0.0018	-0.0141	-0.0091	0.0068
	Z	0.0308	-0.0369	-0.0090	-0.0134	-0.0369	-0.0218
AT. 17 O	X	-0.0232	-0.0315	0.0124	0.0051	0.0157	0.0242
	Y	0.0512	0.0207	-0.0199	-0.0229	-0.0302	-0.0159
	Z	-0.0329	0.0297	0.0055	0.0016	0.0179	-0.0179
AT. 18 O	X	0.0232	0.0315	-0.0124	-0.0051	0.0157	-0.0242
	Y	0.0512	0.0207	-0.0199	-0.0229	0.0302	-0.0159
	Z	0.0329	-0.0297	-0.0055	-0.0016	0.0179	0.0179
AT. 19 O	X	0.0232	-0.0315	-0.0124	0.0051	0.0157	-0.0242
	Y	-0.0512	0.0207	0.0199	-0.0229	-0.0302	0.0159
	Z	0.0329	0.0297	-0.0055	0.0016	0.0179	0.0179
AT. 20 O	X	-0.0232	0.0315	0.0124	-0.0051	0.0157	0.0242
	Y	-0.0512	0.0207	0.0199	-0.0229	0.0302	0.0159
	Z	-0.0329	-0.0297	0.0055	-0.0016	0.0179	-0.0179
AT. 21 O	X	-0.0196	-0.0349	0.0105	-0.0019	-0.0019	0.0318
	Y	0.0215	0.0045	0.0066	0.0122	0.0030	0.0233
	Z	-0.0388	-0.0372	0.0078	0.0089	0.0036	-0.0027
AT. 22 O	X	0.0196	0.0349	-0.0105	0.0019	-0.0019	-0.0318
	Y	0.0215	0.0045	0.0066	0.0122	-0.0030	0.0233
	Z	0.0388	0.0372	-0.0078	-0.0089	0.0036	0.0027
AT. 23 O	X	0.0196	-0.0349	-0.0105	-0.0019	-0.0019	-0.0318
	Y	-0.0215	0.0045	-0.0066	0.0122	0.0030	-0.0233
	Z	0.0388	-0.0372	-0.0078	0.0089	0.0036	0.0027
AT. 24 O	X	-0.0196	0.0349	0.0105	0.0019	-0.0019	0.0318
	Y	-0.0215	0.0045	-0.0066	0.0122	-0.0030	-0.0233

	Z	-0.0388	0.0372	0.0078	-0.0089	0.0036	-0.0027
AT. 25 O	X	-0.0146	0.0142	0.0011	0.0051	-0.0022	0.0165
	Y	-0.0405	-0.0318	0.0175	-0.0253	0.0627	-0.0026
	Z	-0.0471	-0.0210	0.0106	-0.0049	0.0299	-0.0153
AT. 26 O	X	0.0146	-0.0142	-0.0011	-0.0051	-0.0022	-0.0165
	Y	-0.0405	-0.0318	0.0175	-0.0253	-0.0627	-0.0026
	Z	0.0471	0.0210	-0.0106	0.0049	0.0299	0.0153
AT. 27 O	X	0.0146	0.0142	-0.0011	0.0051	-0.0022	-0.0165
	Y	0.0405	-0.0318	-0.0175	-0.0253	0.0627	0.0026
	Z	0.0471	-0.0210	-0.0106	-0.0049	0.0299	0.0153
AT. 28 O	X	-0.0146	-0.0142	0.0011	-0.0051	-0.0022	0.0165
	Y	0.0405	-0.0318	-0.0175	-0.0253	-0.0627	0.0026
	Z	-0.0471	0.0210	0.0106	0.0049	0.0299	-0.0153

FREQ(CM**-1)		153.42	160.10	164.87	172.34	172.64	175.87
AT. 1 CA	X	-0.0097	-0.0279	0.0211	0.0310	-0.0130	0.0257
	Y	-0.0286	-0.0071	0.0282	-0.0018	-0.0103	-0.0270
	Z	-0.0032	0.0283	-0.0043	0.0284	-0.0259	0.0336
AT. 2 CA	X	-0.0097	-0.0279	-0.0211	0.0310	-0.0130	-0.0257
	Y	0.0286	0.0071	0.0282	0.0018	0.0103	-0.0270
	Z	-0.0032	0.0283	0.0043	0.0284	-0.0259	-0.0336
AT. 3 CA	X	0.0097	0.0279	-0.0211	-0.0310	-0.0130	-0.0257
	Y	0.0286	0.0071	-0.0282	0.0018	-0.0103	0.0270
	Z	0.0032	-0.0283	0.0043	-0.0284	-0.0259	-0.0336
AT. 4 CA	X	0.0097	0.0279	0.0211	-0.0310	-0.0130	0.0257
	Y	-0.0286	-0.0071	-0.0282	-0.0018	0.0103	0.0270
	Z	0.0032	-0.0283	-0.0043	-0.0284	-0.0259	0.0336
AT. 5 CA	X	-0.0004	-0.0409	-0.0093	-0.0163	-0.0134	0.0043
	Y	-0.0244	-0.0094	-0.0021	-0.0242	0.0054	-0.0128
	Z	0.0093	-0.0167	-0.0274	-0.0003	-0.0102	-0.0011
AT. 6 CA	X	-0.0004	-0.0409	0.0093	-0.0163	-0.0134	-0.0043
	Y	0.0244	0.0094	-0.0021	0.0242	-0.0054	-0.0128
	Z	0.0093	-0.0167	0.0274	-0.0003	-0.0102	0.0011
AT. 7 CA	X	0.0004	0.0409	0.0093	0.0163	-0.0134	-0.0043
	Y	0.0244	0.0094	0.0021	0.0242	0.0054	0.0128
	Z	-0.0093	0.0167	0.0274	0.0003	-0.0102	0.0011
AT. 8 CA	X	0.0004	0.0409	-0.0093	0.0163	-0.0134	0.0043
	Y	-0.0244	-0.0094	0.0021	-0.0242	-0.0054	0.0128
	Z	-0.0093	0.0167	-0.0274	0.0003	-0.0102	-0.0011
AT. 9 SI	X	0.0060	0.0047	-0.0208	-0.0312	0.0055	-0.0121
	Y	-0.0220	-0.0057	0.0156	-0.0004	-0.0115	-0.0213
	Z	-0.0134	0.0077	-0.0090	-0.0068	0.0138	-0.0099
AT. 10 SI	X	0.0060	0.0047	0.0208	-0.0312	0.0055	0.0121
	Y	0.0220	0.0057	0.0156	0.0004	0.0115	-0.0213
	Z	-0.0134	0.0077	0.0090	-0.0068	0.0138	0.0099
AT. 11 SI	X	-0.0060	-0.0047	0.0208	0.0312	0.0055	0.0121
	Y	0.0220	0.0057	-0.0156	0.0004	-0.0115	0.0213
	Z	0.0134	-0.0077	0.0090	0.0068	0.0138	0.0099
AT. 12 SI	X	-0.0060	-0.0047	-0.0208	0.0312	0.0055	-0.0121
	Y	-0.0220	-0.0057	-0.0156	-0.0004	0.0115	0.0213
	Z	0.0134	-0.0077	-0.0090	0.0068	0.0138	-0.0099
AT. 13 O	X	-0.0291	0.0175	-0.0227	-0.0104	0.0454	-0.0108
	Y	-0.0246	-0.0078	0.0211	0.0033	-0.0015	-0.0199
	Z	-0.0331	0.0332	-0.0402	-0.0149	-0.0147	-0.0156
AT. 14 O	X	-0.0291	0.0175	0.0227	-0.0104	0.0454	0.0108
	Y	0.0246	0.0078	0.0211	-0.0033	0.0015	-0.0199
	Z	-0.0331	0.0332	0.0402	-0.0149	-0.0147	0.0156
AT. 15 O	X	0.0291	-0.0175	0.0227	0.0104	0.0454	0.0108
	Y	0.0246	0.0078	-0.0211	-0.0033	-0.0015	0.0199
	Z	0.0331	-0.0332	0.0402	0.0149	-0.0147	0.0156
AT. 16 O	X	0.0291	-0.0175	-0.0227	0.0104	0.0454	-0.0108
	Y	-0.0246	-0.0078	-0.0211	0.0033	0.0015	0.0199
	Z	0.0331	-0.0332	-0.0402	0.0149	-0.0147	-0.0156
AT. 17 O	X	0.0212	0.0084	-0.0190	-0.0269	0.0076	-0.0062
	Y	-0.0535	-0.0036	0.0103	0.0028	-0.0097	-0.0183
	Z	0.0010	-0.0065	-0.0044	-0.0033	0.0135	-0.0172
AT. 18 O	X	0.0212	0.0084	0.0190	-0.0269	0.0076	0.0062
	Y	0.0535	0.0036	0.0103	-0.0028	0.0097	-0.0183
	Z	0.0010	-0.0065	0.0044	-0.0033	0.0135	0.0172
AT. 19 O	X	-0.0212	-0.0084	0.0190	0.0269	0.0076	0.0062
	Y	0.0535	0.0036	-0.0103	-0.0028	-0.0097	0.0183
	Z	-0.0010	0.0065	0.0044	0.0033	0.0135	0.0172
AT. 20 O	X	-0.0212	-0.0084	-0.0190	0.0269	0.0076	-0.0062
	Y	-0.0535	-0.0036	-0.0103	0.0028	0.0097	0.0183
	Z	-0.0010	0.0065	-0.0044	0.0033	0.0135	-0.0172
AT. 21 O	X	0.0189	-0.0013	-0.0068	-0.0141	0.0156	0.0069
	Y	0.0041	0.0119	-0.0009	-0.0182	-0.0403	-0.0257
	Z	-0.0023	-0.0035	0.0138	0.0140	0.0318	0.0147

AT. 22 O	X	0.0189	-0.0013	0.0068	-0.0141	0.0156	-0.0069
	Y	-0.0041	-0.0119	-0.0009	0.0182	0.0403	-0.0257
	Z	-0.0023	-0.0035	-0.0138	0.0140	0.0318	-0.0147
AT. 23 O	X	-0.0189	0.0013	0.0068	0.0141	0.0156	-0.0069
	Y	-0.0041	-0.0119	0.0009	0.0182	-0.0403	0.0257
	Z	0.0023	0.0035	-0.0138	-0.0140	0.0318	-0.0147
AT. 24 O	X	-0.0189	0.0013	-0.0068	0.0141	0.0156	0.0069
	Y	0.0041	0.0119	0.0009	-0.0182	0.0403	0.0257
	Z	0.0023	0.0035	0.0138	-0.0140	0.0318	0.0147
AT. 25 O	X	0.0173	0.0127	-0.0256	-0.0241	-0.0122	-0.0177
	Y	0.0213	-0.0215	0.0299	0.0142	0.0163	-0.0079
	Z	0.0056	-0.0015	0.0012	0.0007	0.0350	-0.0023
AT. 26 O	X	0.0173	0.0127	0.0256	-0.0241	-0.0122	0.0177
	Y	-0.0213	0.0215	0.0299	-0.0142	-0.0163	-0.0079
	Z	0.0056	-0.0015	-0.0012	0.0007	0.0350	0.0023
AT. 27 O	X	-0.0173	-0.0127	0.0256	0.0241	-0.0122	0.0177
	Y	-0.0213	0.0215	-0.0299	-0.0142	0.0163	0.0079
	Z	-0.0056	0.0015	-0.0012	-0.0007	0.0350	0.0023
AT. 28 O	X	-0.0173	-0.0127	-0.0256	0.0241	-0.0122	-0.0177
	Y	0.0213	-0.0215	-0.0299	0.0142	-0.0163	0.0079
	Z	-0.0056	0.0015	0.0012	-0.0007	0.0350	-0.0023

FREQ(CM**-1)		179.37	185.83	197.56	198.37	199.36	203.59
AT. 1 CA	X	-0.0364	-0.0112	-0.0181	-0.0168	-0.0108	-0.0023
	Y	0.0029	0.0052	0.0052	0.0401	-0.0033	0.0272
	Z	-0.0197	0.0130	-0.0090	0.0025	-0.0340	0.0339
AT. 2 CA	X	0.0364	0.0112	0.0181	-0.0168	-0.0108	0.0023
	Y	0.0029	0.0052	0.0052	-0.0401	0.0033	0.0272
	Z	0.0197	-0.0130	0.0090	0.0025	-0.0340	-0.0339
AT. 3 CA	X	-0.0364	-0.0112	0.0181	0.0168	-0.0108	0.0023
	Y	0.0029	0.0052	-0.0052	-0.0401	-0.0033	-0.0272
	Z	-0.0197	0.0130	0.0090	-0.0025	-0.0340	-0.0339
AT. 4 CA	X	0.0364	0.0112	-0.0181	0.0168	-0.0108	-0.0023
	Y	0.0029	0.0052	-0.0052	0.0401	0.0033	-0.0272
	Z	0.0197	-0.0130	-0.0090	-0.0025	-0.0340	0.0339
AT. 5 CA	X	0.0226	0.0341	0.0260	-0.0064	0.0176	0.0155
	Y	-0.0056	0.0054	-0.0207	-0.0196	0.0053	-0.0157
	Z	-0.0141	0.0346	-0.0328	0.0013	0.0066	0.0055
AT. 6 CA	X	-0.0226	-0.0341	-0.0260	-0.0064	0.0176	-0.0155
	Y	-0.0056	0.0054	-0.0207	0.0196	-0.0053	-0.0157
	Z	0.0141	-0.0346	0.0328	0.0013	0.0066	-0.0055
AT. 7 CA	X	0.0226	0.0341	-0.0260	0.0064	0.0176	-0.0155
	Y	-0.0056	0.0054	0.0207	0.0196	0.0053	0.0157
	Z	-0.0141	0.0346	0.0328	-0.0013	0.0066	-0.0055
AT. 8 CA	X	-0.0226	-0.0341	0.0260	0.0064	0.0176	0.0155
	Y	-0.0056	0.0054	0.0207	-0.0196	-0.0053	0.0157
	Z	0.0141	-0.0346	-0.0328	-0.0013	0.0066	0.0055
AT. 9 SI	X	-0.0091	0.0035	0.0110	0.0087	0.0007	0.0144
	Y	0.0020	-0.0036	-0.0134	0.0222	0.0054	0.0150
	Z	0.0053	-0.0057	-0.0165	-0.0037	0.0106	0.0118
AT. 10 SI	X	0.0091	-0.0035	-0.0110	0.0087	0.0007	-0.0144
	Y	0.0020	-0.0036	-0.0134	-0.0222	-0.0054	0.0150
	Z	-0.0053	0.0057	0.0165	-0.0037	0.0106	-0.0118
AT. 11 SI	X	-0.0091	0.0035	-0.0110	-0.0087	0.0007	-0.0144
	Y	0.0020	-0.0036	0.0134	-0.0222	0.0054	-0.0150
	Z	0.0053	-0.0057	0.0165	0.0037	0.0106	-0.0118
AT. 12 SI	X	0.0091	-0.0035	0.0110	-0.0087	0.0007	0.0144
	Y	0.0020	-0.0036	0.0134	0.0222	-0.0054	-0.0150
	Z	-0.0053	0.0057	-0.0165	0.0037	0.0106	0.0118
AT. 13 O	X	0.0421	0.0004	0.0140	-0.0024	-0.0307	0.0157
	Y	0.0091	-0.0005	-0.0102	0.0219	-0.0042	0.0150
	Z	0.0036	-0.0335	-0.0179	-0.0216	0.0403	0.0100
AT. 14 O	X	-0.0421	-0.0004	-0.0140	-0.0024	-0.0307	-0.0157
	Y	0.0091	-0.0005	-0.0102	-0.0219	0.0042	0.0150
	Z	-0.0036	0.0335	0.0179	-0.0216	0.0403	-0.0100
AT. 15 O	X	0.0421	0.0004	-0.0140	0.0024	-0.0307	-0.0157
	Y	0.0091	-0.0005	0.0102	-0.0219	-0.0042	-0.0150
	Z	0.0036	-0.0335	0.0179	0.0216	0.0403	-0.0100
AT. 16 O	X	-0.0421	-0.0004	0.0140	0.0024	-0.0307	0.0157
	Y	0.0091	-0.0005	0.0102	0.0219	0.0042	-0.0150
	Z	-0.0036	0.0335	-0.0179	0.0216	0.0403	0.0100
AT. 17 O	X	-0.0170	0.0123	0.0027	0.0196	0.0053	0.0204
	Y	0.0252	-0.0306	-0.0057	-0.0058	0.0137	0.0007
	Z	-0.0057	0.0113	-0.0105	-0.0032	-0.0183	0.0092
AT. 18 O	X	0.0170	-0.0123	-0.0027	0.0196	0.0053	-0.0204
	Y	0.0252	-0.0306	-0.0057	0.0058	-0.0137	0.0007
	Z	0.0057	-0.0113	0.0105	-0.0032	-0.0183	-0.0092
AT. 19 O	X	-0.0170	0.0123	-0.0027	-0.0196	0.0053	-0.0204

	Y	0.0252	-0.0306	0.0057	0.0058	0.0137	-0.0007
	Z	-0.0057	0.0113	0.0105	0.0032	-0.0183	-0.0092
AT. 20 O	X	0.0170	-0.0123	0.0027	-0.0196	0.0053	0.0204
	Y	0.0252	-0.0306	0.0057	-0.0058	-0.0137	-0.0007
	Z	0.0057	-0.0113	-0.0105	0.0032	-0.0183	0.0092
AT. 21 O	X	0.0005	0.0076	0.0063	0.0108	0.0175	0.0182
	Y	-0.0236	-0.0121	-0.0163	-0.0053	0.0209	-0.0081
	Z	0.0162	0.0015	-0.0215	0.0038	0.0290	0.0205
AT. 22 O	X	-0.0005	-0.0076	-0.0063	0.0108	0.0175	-0.0182
	Y	-0.0236	-0.0121	-0.0163	0.0053	-0.0209	-0.0081
	Z	-0.0162	-0.0015	0.0215	0.0038	0.0290	-0.0205
AT. 23 O	X	0.0005	0.0076	-0.0063	-0.0108	0.0175	-0.0182
	Y	-0.0236	-0.0121	0.0163	0.0053	0.0209	0.0081
	Z	0.0162	0.0015	0.0215	-0.0038	0.0290	-0.0205
AT. 24 O	X	-0.0005	-0.0076	0.0063	-0.0108	0.0175	0.0182
	Y	-0.0236	-0.0121	0.0163	-0.0053	-0.0209	0.0081
	Z	-0.0162	-0.0015	-0.0215	-0.0038	0.0290	0.0205
AT. 25 O	X	-0.0212	-0.0027	0.0100	0.0022	-0.0099	0.0033
	Y	-0.0068	0.0236	-0.0092	0.0329	-0.0148	0.0099
	Z	0.0016	0.0117	-0.0165	0.0066	-0.0004	0.0144
AT. 26 O	X	0.0212	0.0027	-0.0100	0.0022	-0.0099	-0.0033
	Y	-0.0068	0.0236	-0.0092	-0.0329	0.0148	0.0099
	Z	-0.0016	-0.0117	0.0165	0.0066	-0.0004	-0.0144
AT. 27 O	X	-0.0212	-0.0027	-0.0100	-0.0022	-0.0099	-0.0033
	Y	-0.0068	0.0236	0.0092	-0.0329	-0.0148	-0.0099
	Z	0.0016	0.0117	0.0165	-0.0066	-0.0004	-0.0144
AT. 28 O	X	0.0212	0.0027	0.0100	-0.0022	-0.0099	0.0033
	Y	-0.0068	0.0236	0.0092	0.0329	0.0148	-0.0099
	Z	-0.0016	-0.0117	-0.0165	-0.0066	-0.0004	0.0144
FREQ(CM**-1)		213.61	217.41	218.78	225.80	230.14	240.21
AT. 1 CA	X	-0.0164	0.0044	-0.0245	0.0022	-0.0372	0.0163
	Y	-0.0177	-0.0047	-0.0017	0.0103	0.0176	0.0040
	Z	0.0172	-0.0115	0.0165	0.0222	0.0155	-0.0170
AT. 2 CA	X	-0.0164	0.0044	0.0245	-0.0022	-0.0372	-0.0163
	Y	0.0177	0.0047	-0.0017	0.0103	-0.0176	0.0040
	Z	0.0172	-0.0115	-0.0165	-0.0222	0.0155	0.0170
AT. 3 CA	X	-0.0164	-0.0044	-0.0245	0.0022	-0.0372	-0.0163
	Y	-0.0177	0.0047	-0.0017	0.0103	0.0176	-0.0040
	Z	0.0172	0.0115	0.0165	0.0222	0.0155	0.0170
AT. 4 CA	X	-0.0164	-0.0044	0.0245	-0.0022	-0.0372	0.0163
	Y	0.0177	-0.0047	-0.0017	0.0103	-0.0176	-0.0040
	Z	0.0172	0.0115	-0.0165	-0.0222	0.0155	-0.0170
AT. 5 CA	X	0.0282	-0.0161	-0.0283	0.0099	0.0099	-0.0098
	Y	0.0337	-0.0015	0.0273	-0.0175	-0.0164	0.0084
	Z	-0.0045	0.0259	0.0017	-0.0242	-0.0041	-0.0099
AT. 6 CA	X	0.0282	-0.0161	0.0283	-0.0099	0.0099	0.0098
	Y	-0.0337	0.0015	0.0273	-0.0175	0.0164	0.0084
	Z	-0.0045	0.0259	-0.0017	0.0242	-0.0041	0.0099
AT. 7 CA	X	0.0282	0.0161	-0.0283	0.0099	0.0099	0.0098
	Y	0.0337	0.0015	0.0273	-0.0175	-0.0164	-0.0084
	Z	-0.0045	-0.0259	0.0017	-0.0242	-0.0041	0.0099
AT. 8 CA	X	0.0282	0.0161	0.0283	-0.0099	0.0099	-0.0098
	Y	-0.0337	-0.0015	0.0273	-0.0175	0.0164	-0.0084
	Z	-0.0045	-0.0259	-0.0017	0.0242	-0.0041	-0.0099
AT. 9 SI	X	-0.0020	0.0065	0.0079	0.0075	0.0096	0.0149
	Y	-0.0029	-0.0055	-0.0121	0.0056	-0.0079	-0.0069
	Z	-0.0069	0.0242	0.0082	0.0224	-0.0056	0.0080
AT. 10 SI	X	-0.0020	0.0065	-0.0079	-0.0075	0.0096	-0.0149
	Y	0.0029	0.0055	-0.0121	0.0056	0.0079	-0.0069
	Z	-0.0069	0.0242	-0.0082	-0.0224	-0.0056	-0.0080
AT. 11 SI	X	-0.0020	-0.0065	0.0079	0.0075	0.0096	-0.0149
	Y	-0.0029	0.0055	-0.0121	0.0056	-0.0079	0.0069
	Z	-0.0069	-0.0242	0.0082	0.0224	-0.0056	-0.0080
AT. 12 SI	X	-0.0020	-0.0065	-0.0079	-0.0075	0.0096	0.0149
	Y	0.0029	-0.0055	-0.0121	0.0056	0.0079	0.0069
	Z	-0.0069	-0.0242	-0.0082	-0.0224	-0.0056	0.0080
AT. 13 O	X	0.0116	0.0200	0.0256	-0.0130	0.0078	0.0312
	Y	-0.0023	-0.0022	-0.0068	0.0038	-0.0080	-0.0017
	Z	-0.0071	0.0149	0.0040	0.0179	-0.0057	0.0094
AT. 14 O	X	0.0116	0.0200	-0.0256	0.0130	0.0078	-0.0312
	Y	0.0023	0.0022	-0.0068	0.0038	0.0080	-0.0017
	Z	-0.0071	0.0149	-0.0040	-0.0179	-0.0057	-0.0094
AT. 15 O	X	0.0116	-0.0200	0.0256	-0.0130	0.0078	-0.0312
	Y	-0.0023	0.0022	-0.0068	0.0038	-0.0080	0.0017
	Z	-0.0071	-0.0149	0.0040	0.0179	-0.0057	-0.0094
AT. 16 O	X	0.0116	-0.0200	-0.0256	0.0130	0.0078	0.0312
	Y	0.0023	-0.0022	-0.0068	0.0038	0.0080	0.0017

	Z	-0.0071	-0.0149	-0.0040	-0.0179	-0.0057	0.0094
AT. 17 O	X	-0.0159	0.0046	0.0093	0.0157	0.0186	0.0170
	Y	0.0262	0.0060	-0.0170	-0.0087	-0.0199	0.0140
	Z	-0.0080	0.0206	0.0154	0.0155	-0.0118	-0.0214
AT. 18 O	X	-0.0159	0.0046	-0.0093	-0.0157	0.0186	-0.0170
	Y	-0.0262	-0.0060	-0.0170	-0.0087	0.0199	0.0140
	Z	-0.0080	0.0206	-0.0154	-0.0155	-0.0118	0.0214
AT. 19 O	X	-0.0159	-0.0046	0.0093	0.0157	0.0186	-0.0170
	Y	0.0262	-0.0060	-0.0170	-0.0087	-0.0199	-0.0140
	Z	-0.0080	-0.0206	0.0154	0.0155	-0.0118	0.0214
AT. 20 O	X	-0.0159	-0.0046	-0.0093	-0.0157	0.0186	0.0170
	Y	-0.0262	0.0060	-0.0170	-0.0087	0.0199	-0.0140
	Z	-0.0080	-0.0206	-0.0154	-0.0155	-0.0118	-0.0214
AT. 21 O	X	-0.0056	0.0161	0.0151	0.0146	0.0232	0.0304
	Y	-0.0108	-0.0228	-0.0055	-0.0015	0.0000	-0.0182
	Z	-0.0043	0.0404	0.0134	0.0303	0.0065	0.0289
AT. 22 O	X	-0.0056	0.0161	-0.0151	-0.0146	0.0232	-0.0304
	Y	0.0108	0.0228	-0.0055	-0.0015	-0.0000	-0.0182
	Z	-0.0043	0.0404	-0.0134	-0.0303	0.0065	-0.0289
AT. 23 O	X	-0.0056	-0.0161	0.0151	0.0146	0.0232	-0.0304
	Y	-0.0108	0.0228	-0.0055	-0.0015	0.0000	0.0182
	Z	-0.0043	-0.0404	0.0134	0.0303	0.0065	-0.0289
AT. 24 O	X	-0.0056	-0.0161	-0.0151	-0.0146	0.0232	0.0304
	Y	0.0108	-0.0228	-0.0055	-0.0015	-0.0000	0.0182
	Z	-0.0043	-0.0404	-0.0134	-0.0303	0.0065	0.0289
AT. 25 O	X	-0.0157	-0.0023	0.0041	-0.0000	0.0018	-0.0182
	Y	0.0050	0.0105	-0.0136	0.0144	-0.0193	-0.0268
	Z	-0.0001	0.0333	0.0088	0.0303	-0.0079	0.0042
AT. 26 O	X	-0.0157	-0.0023	-0.0041	0.0000	0.0018	0.0182
	Y	-0.0050	-0.0105	-0.0136	0.0144	0.0193	-0.0268
	Z	-0.0001	0.0333	-0.0088	-0.0303	-0.0079	-0.0042
AT. 27 O	X	-0.0157	0.0023	0.0041	-0.0000	0.0018	0.0182
	Y	0.0050	-0.0105	-0.0136	0.0144	-0.0193	0.0268
	Z	-0.0001	-0.0333	0.0088	0.0303	-0.0079	-0.0042
AT. 28 O	X	-0.0157	0.0023	-0.0041	0.0000	0.0018	-0.0182
	Y	-0.0050	0.0105	-0.0136	0.0144	0.0193	0.0268
	Z	-0.0001	-0.0333	-0.0088	-0.0303	-0.0079	0.0042

FREQ(CM**-1)		247.98	251.48	252.38	256.55	257.40	264.62
AT. 1 CA	X	0.0113	-0.0075	-0.0183	0.0226	-0.0079	-0.0129
	Y	0.0103	-0.0042	-0.0177	0.0170	0.0350	-0.0038
	Z	-0.0021	-0.0115	-0.0004	-0.0014	-0.0045	-0.0172
AT. 2 CA	X	0.0113	0.0075	-0.0183	0.0226	0.0079	-0.0129
	Y	-0.0103	-0.0042	0.0177	-0.0170	0.0350	0.0038
	Z	-0.0021	0.0115	-0.0004	-0.0014	0.0045	-0.0172
AT. 3 CA	X	-0.0113	0.0075	-0.0183	-0.0226	-0.0079	-0.0129
	Y	-0.0103	0.0042	-0.0177	-0.0170	0.0350	-0.0038
	Z	0.0021	0.0115	-0.0004	0.0014	-0.0045	-0.0172
AT. 4 CA	X	-0.0113	-0.0075	-0.0183	-0.0226	0.0079	-0.0129
	Y	0.0103	0.0042	0.0177	0.0170	0.0350	0.0038
	Z	0.0021	-0.0115	-0.0004	0.0014	0.0045	-0.0172
AT. 5 CA	X	0.0005	0.0153	-0.0127	-0.0016	0.0028	0.0080
	Y	-0.0214	-0.0065	0.0085	0.0002	-0.0084	-0.0068
	Z	0.0265	0.0263	-0.0066	-0.0063	-0.0062	0.0375
AT. 6 CA	X	0.0005	-0.0153	-0.0127	-0.0016	-0.0028	0.0080
	Y	0.0214	-0.0065	-0.0085	-0.0002	-0.0084	0.0068
	Z	0.0265	-0.0263	-0.0066	-0.0063	0.0062	0.0375
AT. 7 CA	X	-0.0005	-0.0153	-0.0127	0.0016	0.0028	0.0080
	Y	0.0214	0.0065	0.0085	-0.0002	-0.0084	-0.0068
	Z	-0.0265	-0.0263	-0.0066	0.0063	-0.0062	0.0375
AT. 8 CA	X	-0.0005	0.0153	-0.0127	0.0016	-0.0028	0.0080
	Y	-0.0214	0.0065	-0.0085	0.0002	-0.0084	0.0068
	Z	-0.0265	0.0263	-0.0066	0.0063	0.0062	0.0375
AT. 9 SI	X	0.0099	-0.0001	0.0116	-0.0043	0.0079	0.0032
	Y	-0.0053	0.0126	0.0100	0.0001	-0.0027	0.0012
	Z	-0.0185	-0.0132	0.0014	0.0162	-0.0138	-0.0053
AT. 10 SI	X	0.0099	0.0001	0.0116	-0.0043	-0.0079	0.0032
	Y	0.0053	0.0126	-0.0100	-0.0001	-0.0027	-0.0012
	Z	-0.0185	0.0132	0.0014	0.0162	0.0138	-0.0053
AT. 11 SI	X	-0.0099	0.0001	0.0116	0.0043	0.0079	0.0032
	Y	0.0053	-0.0126	0.0100	-0.0001	-0.0027	0.0012
	Z	0.0185	0.0132	0.0014	-0.0162	-0.0138	-0.0053
AT. 12 SI	X	-0.0099	-0.0001	0.0116	0.0043	-0.0079	0.0032
	Y	-0.0053	-0.0126	-0.0100	0.0001	-0.0027	-0.0012
	Z	0.0185	-0.0132	0.0014	-0.0162	0.0138	-0.0053
AT. 13 O	X	0.0235	-0.0145	0.0119	-0.0157	-0.0067	0.0085
	Y	-0.0056	0.0106	0.0065	-0.0040	-0.0078	0.0047
	Z	0.0125	-0.0260	0.0203	0.0250	0.0002	-0.0221

AT. 14 O	X	0.0235	0.0145	0.0119	-0.0157	0.0067	0.0085
	Y	0.0056	0.0106	-0.0065	0.0040	-0.0078	-0.0047
	Z	0.0125	0.0260	0.0203	0.0250	-0.0002	-0.0221
AT. 15 O	X	-0.0235	0.0145	0.0119	0.0157	-0.0067	0.0085
	Y	0.0056	-0.0106	0.0065	0.0040	-0.0078	0.0047
	Z	-0.0125	0.0260	0.0203	-0.0250	0.0002	-0.0221
AT. 16 O	X	-0.0235	-0.0145	0.0119	0.0157	0.0067	0.0085
	Y	-0.0056	-0.0106	-0.0065	-0.0040	-0.0078	-0.0047
	Z	-0.0125	-0.0260	0.0203	-0.0250	-0.0002	-0.0221
AT. 17 O	X	0.0023	0.0130	0.0163	0.0117	0.0154	-0.0089
	Y	0.0039	0.0060	-0.0071	-0.0400	-0.0215	0.0052
	Z	-0.0030	-0.0364	0.0150	0.0267	-0.0059	0.0127
AT. 18 O	X	0.0023	-0.0130	0.0163	0.0117	-0.0154	-0.0089
	Y	-0.0039	0.0060	0.0071	0.0400	-0.0215	-0.0052
	Z	-0.0030	0.0364	0.0150	0.0267	0.0059	0.0127
AT. 19 O	X	-0.0023	-0.0130	0.0163	-0.0117	0.0154	-0.0089
	Y	-0.0039	-0.0060	-0.0071	0.0400	-0.0215	0.0052
	Z	0.0030	0.0364	0.0150	-0.0267	-0.0059	0.0127
AT. 20 O	X	-0.0023	0.0130	0.0163	-0.0117	-0.0154	-0.0089
	Y	0.0039	-0.0060	0.0071	-0.0400	-0.0215	-0.0052
	Z	0.0030	-0.0364	0.0150	-0.0267	0.0059	0.0127
AT. 21 O	X	0.0116	0.0229	-0.0013	-0.0142	0.0131	-0.0116
	Y	0.0077	0.0139	0.0418	0.0219	0.0010	-0.0129
	Z	-0.0125	0.0130	-0.0214	-0.0046	-0.0063	-0.0206
AT. 22 O	X	0.0116	-0.0229	-0.0013	-0.0142	-0.0131	-0.0116
	Y	-0.0077	0.0139	-0.0418	-0.0219	0.0010	0.0129
	Z	-0.0125	-0.0130	-0.0214	-0.0046	0.0063	-0.0206
AT. 23 O	X	-0.0116	-0.0229	-0.0013	0.0142	0.0131	-0.0116
	Y	-0.0077	-0.0139	0.0418	-0.0219	0.0010	-0.0129
	Z	0.0125	-0.0130	-0.0214	0.0046	-0.0063	-0.0206
AT. 24 O	X	-0.0116	0.0229	-0.0013	0.0142	-0.0131	-0.0116
	Y	0.0077	-0.0139	-0.0418	0.0219	0.0010	0.0129
	Z	0.0125	0.0130	-0.0214	0.0046	0.0063	-0.0206
AT. 25 O	X	0.0050	-0.0182	0.0301	0.0252	0.0169	0.0187
	Y	-0.0356	0.0036	0.0219	-0.0051	-0.0336	0.0004
	Z	-0.0293	-0.0146	0.0012	0.0064	-0.0286	-0.0117
AT. 26 O	X	0.0050	0.0182	0.0301	0.0252	-0.0169	0.0187
	Y	0.0356	0.0036	-0.0219	0.0051	-0.0336	-0.0004
	Z	-0.0293	0.0146	0.0012	0.0064	0.0286	-0.0117
AT. 27 O	X	-0.0050	0.0182	0.0301	-0.0252	0.0169	0.0187
	Y	0.0356	-0.0036	0.0219	0.0051	-0.0336	0.0004
	Z	0.0293	0.0146	0.0012	-0.0064	-0.0286	-0.0117
AT. 28 O	X	-0.0050	-0.0182	0.0301	-0.0252	-0.0169	0.0187
	Y	-0.0356	-0.0036	-0.0219	-0.0051	-0.0336	-0.0004
	Z	0.0293	-0.0146	0.0012	-0.0064	0.0286	-0.0117

FREQ(CM**-1)		265.88	271.60	273.61	274.80	278.33	286.05
AT. 1 CA	X	0.0331	0.0132	-0.0114	-0.0178	0.0121	0.0210
	Y	0.0133	0.0132	0.0193	-0.0167	-0.0268	0.0037
	Z	-0.0086	-0.0006	0.0116	0.0025	0.0012	-0.0115
AT. 2 CA	X	-0.0331	-0.0132	-0.0114	0.0178	-0.0121	0.0210
	Y	0.0133	0.0132	-0.0193	-0.0167	-0.0268	-0.0037
	Z	0.0086	0.0006	0.0116	-0.0025	-0.0012	-0.0115
AT. 3 CA	X	-0.0331	0.0132	0.0114	-0.0178	-0.0121	-0.0210
	Y	-0.0133	0.0132	-0.0193	-0.0167	0.0268	-0.0037
	Z	0.0086	-0.0006	-0.0116	0.0025	-0.0012	0.0115
AT. 4 CA	X	0.0331	-0.0132	0.0114	0.0178	0.0121	-0.0210
	Y	-0.0133	0.0132	0.0193	-0.0167	0.0268	0.0037
	Z	-0.0086	0.0006	-0.0116	-0.0025	0.0012	0.0115
AT. 5 CA	X	0.0024	-0.0007	0.0001	0.0059	0.0001	-0.0179
	Y	-0.0053	0.0029	0.0174	-0.0089	-0.0071	0.0128
	Z	0.0079	0.0253	0.0078	0.0148	-0.0119	-0.0179
AT. 6 CA	X	-0.0024	0.0007	0.0001	-0.0059	-0.0001	-0.0179
	Y	-0.0053	0.0029	-0.0174	-0.0089	-0.0071	-0.0128
	Z	-0.0079	-0.0253	0.0078	-0.0148	0.0119	-0.0179
AT. 7 CA	X	-0.0024	-0.0007	-0.0001	0.0059	-0.0001	0.0179
	Y	0.0053	0.0029	-0.0174	-0.0089	0.0071	-0.0128
	Z	-0.0079	0.0253	-0.0078	0.0148	0.0119	0.0179
AT. 8 CA	X	0.0024	0.0007	-0.0001	-0.0059	0.0001	0.0179
	Y	0.0053	0.0029	0.0174	-0.0089	0.0071	0.0128
	Z	0.0079	-0.0253	-0.0078	-0.0148	-0.0119	0.0179
AT. 9 SI	X	0.0079	-0.0087	-0.0054	0.0120	0.0142	0.0068
	Y	-0.0004	-0.0041	-0.0208	0.0081	0.0141	-0.0059
	Z	-0.0077	0.0174	0.0034	0.0007	-0.0024	-0.0094
AT. 10 SI	X	-0.0079	0.0087	-0.0054	-0.0120	-0.0142	0.0068
	Y	-0.0004	-0.0041	0.0208	0.0081	0.0141	0.0059
	Z	0.0077	-0.0174	0.0034	-0.0007	0.0024	-0.0094
AT. 11 SI	X	-0.0079	-0.0087	0.0054	0.0120	-0.0142	-0.0068

	Y	0.0004	-0.0041	0.0208	0.0081	-0.0141	0.0059
	Z	0.0077	0.0174	-0.0034	0.0007	0.0024	0.0094
AT. 12 SI	X	0.0079	0.0087	0.0054	-0.0120	0.0142	-0.0068
	Y	0.0004	-0.0041	-0.0208	0.0081	-0.0141	-0.0059
	Z	-0.0077	-0.0174	-0.0034	-0.0007	-0.0024	0.0094
AT. 13 O	X	-0.0061	-0.0085	-0.0095	-0.0078	0.0136	0.0191
	Y	-0.0050	-0.0036	-0.0161	0.0005	0.0132	-0.0026
	Z	0.0008	0.0208	-0.0259	0.0302	0.0002	-0.0154
AT. 14 O	X	0.0061	0.0085	-0.0095	0.0078	-0.0136	0.0191
	Y	-0.0050	-0.0036	0.0161	0.0005	0.0132	0.0026
	Z	-0.0008	-0.0208	-0.0259	-0.0302	-0.0002	-0.0154
AT. 15 O	X	0.0061	-0.0085	0.0095	-0.0078	-0.0136	-0.0191
	Y	0.0050	-0.0036	0.0161	0.0005	-0.0132	0.0026
	Z	-0.0008	0.0208	0.0259	0.0302	-0.0002	0.0154
AT. 16 O	X	-0.0061	0.0085	0.0095	0.0078	0.0136	-0.0191
	Y	0.0050	-0.0036	-0.0161	0.0005	-0.0132	-0.0026
	Z	0.0008	-0.0208	0.0259	-0.0302	0.0002	0.0154
AT. 17 O	X	0.0199	-0.0078	-0.0091	0.0188	0.0055	0.0216
	Y	-0.0292	0.0133	-0.0180	0.0150	0.0310	-0.0120
	Z	0.0025	-0.0120	0.0102	-0.0203	0.0024	-0.0293
AT. 18 O	X	-0.0199	0.0078	-0.0091	-0.0188	-0.0055	0.0216
	Y	-0.0292	0.0133	0.0180	0.0150	0.0310	0.0120
	Z	-0.0025	0.0120	0.0102	0.0203	-0.0024	-0.0293
AT. 19 O	X	-0.0199	-0.0078	0.0091	0.0188	-0.0055	-0.0216
	Y	0.0292	0.0133	0.0180	0.0150	-0.0310	0.0120
	Z	-0.0025	-0.0120	-0.0102	-0.0203	-0.0024	0.0293
AT. 20 O	X	0.0199	0.0078	0.0091	-0.0188	0.0055	-0.0216
	Y	0.0292	0.0133	-0.0180	0.0150	-0.0310	-0.0120
	Z	0.0025	0.0120	-0.0102	0.0203	0.0024	0.0293
AT. 21 O	X	0.0027	0.0030	-0.0035	0.0200	0.0125	0.0226
	Y	0.0160	-0.0112	-0.0315	0.0392	0.0187	-0.0134
	Z	-0.0207	0.0322	0.0090	0.0024	-0.0062	0.0032
AT. 22 O	X	-0.0027	-0.0030	-0.0035	-0.0200	-0.0125	0.0226
	Y	0.0160	-0.0112	0.0315	0.0392	0.0187	0.0134
	Z	0.0207	-0.0322	0.0090	-0.0024	0.0062	0.0032
AT. 23 O	X	-0.0027	0.0030	0.0035	0.0200	-0.0125	-0.0226
	Y	-0.0160	-0.0112	0.0315	0.0392	-0.0187	0.0134
	Z	0.0207	0.0322	-0.0090	0.0024	0.0062	-0.0032
AT. 24 O	X	0.0027	-0.0030	0.0035	-0.0200	0.0125	-0.0226
	Y	-0.0160	-0.0112	-0.0315	0.0392	-0.0187	-0.0134
	Z	-0.0207	-0.0322	-0.0090	-0.0024	-0.0062	-0.0032
AT. 25 O	X	0.0316	-0.0252	-0.0007	0.0121	0.0188	-0.0023
	Y	-0.0062	-0.0317	-0.0296	-0.0049	0.0337	-0.0024
	Z	-0.0147	0.0049	-0.0082	-0.0081	0.0050	-0.0039
AT. 26 O	X	-0.0316	0.0252	-0.0007	-0.0121	-0.0188	-0.0023
	Y	-0.0062	-0.0317	0.0296	-0.0049	0.0337	0.0024
	Z	0.0147	-0.0049	-0.0082	0.0081	-0.0050	-0.0039
AT. 27 O	X	-0.0316	-0.0252	0.0007	0.0121	-0.0188	0.0023
	Y	0.0062	-0.0317	0.0296	-0.0049	-0.0337	0.0024
	Z	0.0147	0.0049	0.0082	-0.0081	-0.0050	0.0039
AT. 28 O	X	0.0316	0.0252	0.0007	-0.0121	0.0188	0.0023
	Y	0.0062	-0.0317	-0.0296	-0.0049	-0.0337	-0.0024
	Z	-0.0147	-0.0049	0.0082	0.0081	0.0050	0.0039

FREQ(CM**-1)		286.08	293.30	297.46	303.09	311.94	313.91
AT. 1 CA	X	-0.0036	0.0059	-0.0047	-0.0013	-0.0075	0.0005
	Y	0.0066	-0.0039	0.0031	-0.0033	0.0064	-0.0223
	Z	-0.0068	0.0068	-0.0206	-0.0013	-0.0179	0.0038
AT. 2 CA	X	-0.0036	0.0059	0.0047	0.0013	-0.0075	0.0005
	Y	-0.0066	0.0039	0.0031	-0.0033	-0.0064	0.0223
	Z	-0.0068	0.0068	0.0206	0.0013	-0.0179	0.0038
AT. 3 CA	X	-0.0036	-0.0059	-0.0047	0.0013	0.0075	0.0005
	Y	0.0066	0.0039	0.0031	0.0033	-0.0064	-0.0223
	Z	-0.0068	-0.0068	-0.0206	0.0013	0.0179	0.0038
AT. 4 CA	X	-0.0036	-0.0059	0.0047	-0.0013	0.0075	0.0005
	Y	-0.0066	-0.0039	0.0031	0.0033	0.0064	0.0223
	Z	-0.0068	-0.0068	0.0206	-0.0013	0.0179	0.0038
AT. 5 CA	X	0.0182	0.0064	-0.0022	0.0069	0.0108	0.0105
	Y	-0.0109	-0.0067	-0.0061	-0.0096	-0.0221	-0.0331
	Z	-0.0211	-0.0117	0.0116	-0.0175	-0.0187	-0.0021
AT. 6 CA	X	0.0182	0.0064	0.0022	-0.0069	0.0108	0.0105
	Y	0.0109	0.0067	-0.0061	-0.0096	0.0221	0.0331
	Z	-0.0211	-0.0117	-0.0116	0.0175	-0.0187	-0.0021
AT. 7 CA	X	0.0182	-0.0064	-0.0022	-0.0069	-0.0108	0.0105
	Y	-0.0109	0.0067	-0.0061	0.0096	0.0221	-0.0331
	Z	-0.0211	0.0117	0.0116	0.0175	0.0187	-0.0021
AT. 8 CA	X	0.0182	-0.0064	0.0022	0.0069	-0.0108	0.0105
	Y	0.0109	-0.0067	-0.0061	0.0096	-0.0221	0.0331

	Z	-0.0211	0.0117	-0.0116	-0.0175	0.0187	-0.0021
AT. 9 SI	X	-0.0045	0.0164	0.0032	-0.0091	-0.0056	-0.0050
	Y	-0.0043	-0.0056	0.0031	0.0023	-0.0132	0.0022
	Z	0.0092	0.0004	0.0248	0.0128	0.0052	-0.0018
AT. 10 SI	X	-0.0045	0.0164	-0.0032	0.0091	-0.0056	-0.0050
	Y	0.0043	0.0056	0.0031	0.0023	0.0132	-0.0022
	Z	0.0092	0.0004	-0.0248	-0.0128	0.0052	-0.0018
AT. 11 SI	X	-0.0045	-0.0164	0.0032	0.0091	0.0056	-0.0050
	Y	-0.0043	0.0056	0.0031	-0.0023	0.0132	0.0022
	Z	0.0092	-0.0004	0.0248	-0.0128	-0.0052	-0.0018
AT. 12 SI	X	-0.0045	-0.0164	-0.0032	-0.0091	0.0056	-0.0050
	Y	0.0043	-0.0056	0.0031	-0.0023	-0.0132	-0.0022
	Z	0.0092	-0.0004	-0.0248	0.0128	-0.0052	-0.0018
AT. 13 O	X	0.0030	0.0311	-0.0037	-0.0288	0.0074	0.0103
	Y	-0.0019	-0.0001	0.0037	-0.0031	-0.0095	0.0094
	Z	0.0193	-0.0127	0.0060	0.0353	0.0166	-0.0023
AT. 14 O	X	0.0030	0.0311	0.0037	0.0288	0.0074	0.0103
	Y	0.0019	0.0001	0.0037	-0.0031	0.0095	-0.0094
	Z	0.0193	-0.0127	-0.0060	-0.0353	0.0166	-0.0023
AT. 15 O	X	0.0030	-0.0311	-0.0037	0.0288	-0.0074	0.0103
	Y	-0.0019	0.0001	0.0037	0.0031	0.0095	0.0094
	Z	0.0193	0.0127	0.0060	-0.0353	-0.0166	-0.0023
AT. 16 O	X	0.0030	-0.0311	0.0037	-0.0288	-0.0074	0.0103
	Y	0.0019	-0.0001	0.0037	0.0031	-0.0095	-0.0094
	Z	0.0193	0.0127	-0.0060	0.0353	-0.0166	-0.0023
AT. 17 O	X	-0.0213	-0.0059	-0.0039	0.0100	-0.0049	-0.0066
	Y	0.0013	0.0102	-0.0022	-0.0096	-0.0095	0.0108
	Z	0.0419	0.0302	0.0431	-0.0146	-0.0055	-0.0107
AT. 18 O	X	-0.0213	-0.0059	0.0039	-0.0100	-0.0049	-0.0066
	Y	-0.0013	-0.0102	-0.0022	-0.0096	0.0095	-0.0108
	Z	0.0419	0.0302	-0.0431	0.0146	-0.0055	-0.0107
AT. 19 O	X	-0.0213	0.0059	-0.0039	-0.0100	0.0049	-0.0066
	Y	0.0013	-0.0102	-0.0022	0.0096	0.0095	0.0108
	Z	0.0419	-0.0302	0.0431	0.0146	0.0055	-0.0107
AT. 20 O	X	-0.0213	0.0059	0.0039	0.0100	0.0049	-0.0066
	Y	-0.0013	0.0102	-0.0022	0.0096	-0.0095	-0.0108
	Z	0.0419	-0.0302	-0.0431	-0.0146	0.0055	-0.0107
AT. 21 O	X	-0.0257	-0.0107	-0.0191	-0.0002	-0.0079	-0.0002
	Y	-0.0090	-0.0325	0.0035	0.0399	-0.0176	0.0149
	Z	-0.0102	-0.0258	-0.0034	0.0159	0.0012	0.0020
AT. 22 O	X	-0.0257	-0.0107	0.0191	0.0002	-0.0079	-0.0002
	Y	0.0090	0.0325	0.0035	0.0399	0.0176	-0.0149
	Z	-0.0102	-0.0258	0.0034	-0.0159	0.0012	0.0020
AT. 23 O	X	-0.0257	0.0107	-0.0191	0.0002	0.0079	-0.0002
	Y	-0.0090	0.0325	0.0035	-0.0399	0.0176	0.0149
	Z	-0.0102	0.0258	-0.0034	-0.0159	-0.0012	0.0020
AT. 24 O	X	-0.0257	0.0107	0.0191	-0.0002	0.0079	-0.0002
	Y	0.0090	-0.0325	0.0035	-0.0399	-0.0176	-0.0149
	Z	-0.0102	0.0258	0.0034	0.0159	-0.0012	0.0020
AT. 25 O	X	0.0155	0.0301	0.0355	-0.0097	-0.0292	-0.0224
	Y	-0.0138	0.0121	-0.0028	-0.0127	-0.0148	0.0184
	Z	0.0027	0.0043	0.0056	0.0049	0.0139	0.0100
AT. 26 O	X	0.0155	0.0301	-0.0355	0.0097	-0.0292	-0.0224
	Y	0.0138	-0.0121	-0.0028	-0.0127	0.0148	-0.0184
	Z	0.0027	0.0043	-0.0056	-0.0049	0.0139	0.0100
AT. 27 O	X	0.0155	-0.0301	0.0355	0.0097	0.0292	-0.0224
	Y	-0.0138	-0.0121	-0.0028	0.0127	0.0148	0.0184
	Z	0.0027	-0.0043	0.0056	-0.0049	-0.0139	0.0100
AT. 28 O	X	0.0155	-0.0301	-0.0355	-0.0097	0.0292	-0.0224
	Y	0.0138	0.0121	-0.0028	0.0127	-0.0148	-0.0184
	Z	0.0027	-0.0043	-0.0056	0.0049	-0.0139	0.0100
FREQ(CM**-1)		355.60	361.13	370.41	380.23	401.12	409.10
AT. 1 CA	X	0.0017	-0.0040	-0.0025	-0.0057	0.0020	-0.0000
	Y	-0.0006	-0.0096	0.0074	-0.0008	0.0010	0.0027
	Z	0.0013	0.0055	-0.0050	0.0053	-0.0088	-0.0018
AT. 2 CA	X	-0.0017	-0.0040	0.0025	0.0057	0.0020	0.0000
	Y	-0.0006	0.0096	0.0074	-0.0008	-0.0010	0.0027
	Z	-0.0013	0.0055	0.0050	-0.0053	-0.0088	0.0018
AT. 3 CA	X	-0.0017	-0.0040	-0.0025	-0.0057	-0.0020	0.0000
	Y	0.0006	-0.0096	0.0074	-0.0008	-0.0010	-0.0027
	Z	-0.0013	0.0055	-0.0050	0.0053	0.0088	0.0018
AT. 4 CA	X	0.0017	-0.0040	0.0025	0.0057	-0.0020	-0.0000
	Y	0.0006	0.0096	0.0074	-0.0008	0.0010	-0.0027
	Z	0.0013	0.0055	0.0050	-0.0053	0.0088	-0.0018
AT. 5 CA	X	0.0056	-0.0080	-0.0011	-0.0003	-0.0036	0.0019
	Y	-0.0009	-0.0065	0.0027	0.0054	-0.0098	0.0034
	Z	0.0004	-0.0074	0.0025	0.0007	-0.0110	-0.0002

```
AT.    6 CA X    -0.0056   -0.0080    0.0011    0.0003   -0.0036   -0.0019
            Y    -0.0009    0.0065    0.0027    0.0054    0.0098    0.0034
            Z    -0.0004   -0.0074   -0.0025   -0.0007   -0.0110    0.0002
AT.    7 CA X    -0.0056   -0.0080   -0.0011   -0.0003    0.0036   -0.0019
            Y     0.0009   -0.0065    0.0027    0.0054    0.0098   -0.0034
            Z    -0.0004   -0.0074    0.0025    0.0007    0.0110    0.0002
AT.    8 CA X     0.0056   -0.0080    0.0011    0.0003    0.0036    0.0019
            Y     0.0009    0.0065    0.0027    0.0054   -0.0098   -0.0034
            Z     0.0004   -0.0074   -0.0025   -0.0007    0.0110   -0.0002
AT.    9 SI X     0.0012    0.0091   -0.0035    0.0088    0.0028    0.0007
            Y    -0.0044    0.0045   -0.0035    0.0003    0.0018    0.0033
            Z     0.0083   -0.0002    0.0037   -0.0032    0.0049   -0.0002
AT.   10 SI X    -0.0012    0.0091    0.0035   -0.0088    0.0028   -0.0007
            Y    -0.0044   -0.0045   -0.0035    0.0003   -0.0018    0.0033
            Z    -0.0083   -0.0002   -0.0037    0.0032    0.0049    0.0002
AT.   11 SI X    -0.0012    0.0091   -0.0035    0.0088   -0.0028   -0.0007
            Y     0.0044    0.0045   -0.0035    0.0003   -0.0018   -0.0033
            Z    -0.0083   -0.0002    0.0037   -0.0032   -0.0049    0.0002
AT.   12 SI X     0.0012    0.0091    0.0035   -0.0088   -0.0028    0.0007
            Y     0.0044   -0.0045   -0.0035    0.0003    0.0018   -0.0033
            Z     0.0083   -0.0002   -0.0037    0.0032   -0.0049   -0.0002
AT.   13 O  X    -0.0089   -0.0285    0.0075   -0.0265   -0.0312   -0.0345
            Y    -0.0016    0.0025   -0.0089   -0.0050   -0.0043   -0.0066
            Z    -0.0262   -0.0129    0.0389    0.0018   -0.0074    0.0179
AT.   14 O  X     0.0089   -0.0285   -0.0075    0.0265   -0.0312    0.0345
            Y    -0.0016   -0.0025   -0.0089   -0.0050    0.0043   -0.0066
            Z     0.0262   -0.0129   -0.0389   -0.0018   -0.0074   -0.0179
AT.   15 O  X     0.0089   -0.0285    0.0075   -0.0265    0.0312    0.0345
            Y     0.0016    0.0025   -0.0089   -0.0050    0.0043    0.0066
            Z     0.0262   -0.0129    0.0389    0.0018    0.0074   -0.0179
AT.   16 O  X    -0.0089   -0.0285   -0.0075    0.0265    0.0312   -0.0345
            Y     0.0016   -0.0025   -0.0089   -0.0050   -0.0043    0.0066
            Z    -0.0262   -0.0129   -0.0389   -0.0018    0.0074    0.0179
AT.   17 O  X    -0.0208   -0.0079    0.0145   -0.0011   -0.0114   -0.0043
            Y     0.0195    0.0340   -0.0207    0.0294    0.0281    0.0206
            Z     0.0317    0.0083   -0.0246   -0.0117    0.0028   -0.0139
AT.   18 O  X     0.0208   -0.0079   -0.0145    0.0011   -0.0114    0.0043
            Y     0.0195   -0.0340   -0.0207    0.0294   -0.0281    0.0206
            Z    -0.0317    0.0083    0.0246    0.0117    0.0028    0.0139
AT.   19 O  X     0.0208   -0.0079    0.0145   -0.0011    0.0114    0.0043
            Y    -0.0195    0.0340   -0.0207    0.0294   -0.0281   -0.0206
            Z    -0.0317    0.0083   -0.0246   -0.0117   -0.0028    0.0139
AT.   20 O  X    -0.0208   -0.0079   -0.0145    0.0011    0.0114   -0.0043
            Y    -0.0195   -0.0340   -0.0207    0.0294    0.0281   -0.0206
            Z     0.0317    0.0083    0.0246    0.0117   -0.0028   -0.0139
AT.   21 O  X     0.0158    0.0207   -0.0232    0.0019    0.0169    0.0041
            Y     0.0191   -0.0186   -0.0120   -0.0436   -0.0035   -0.0326
            Z     0.0201    0.0172   -0.0221    0.0008    0.0190    0.0111
AT.   22 O  X    -0.0158    0.0207    0.0232   -0.0019    0.0169   -0.0041
            Y     0.0191    0.0186   -0.0120   -0.0436    0.0035   -0.0326
            Z    -0.0201    0.0172    0.0221   -0.0008    0.0190   -0.0111
AT.   23 O  X    -0.0158    0.0207   -0.0232    0.0019   -0.0169   -0.0041
            Y    -0.0191   -0.0186   -0.0120   -0.0436    0.0035    0.0326
            Z    -0.0201    0.0172   -0.0221    0.0008   -0.0190   -0.0111
AT.   24 O  X     0.0158    0.0207    0.0232   -0.0019   -0.0169    0.0041
            Y    -0.0191    0.0186   -0.0120   -0.0436   -0.0035    0.0326
            Z     0.0201    0.0172    0.0221   -0.0008   -0.0190    0.0111
AT.   25 O  X     0.0168    0.0298   -0.0027    0.0297    0.0292    0.0318
            Y    -0.0325    0.0005    0.0225    0.0067   -0.0173    0.0054
            Z    -0.0100   -0.0074    0.0135   -0.0021   -0.0083   -0.0091
AT.   26 O  X    -0.0168    0.0298    0.0027   -0.0297    0.0292   -0.0318
            Y    -0.0325   -0.0005    0.0225    0.0067    0.0173    0.0054
            Z     0.0100   -0.0074   -0.0135    0.0021   -0.0083    0.0091
AT.   27 O  X    -0.0168    0.0298   -0.0027    0.0297   -0.0292   -0.0318
            Y     0.0325    0.0005    0.0225    0.0067    0.0173   -0.0054
            Z     0.0100   -0.0074    0.0135   -0.0021    0.0083    0.0091
AT.   28 O  X     0.0168    0.0298    0.0027   -0.0297   -0.0292    0.0318
            Y     0.0325   -0.0005    0.0225    0.0067   -0.0173   -0.0054
            Z    -0.0100   -0.0074   -0.0135    0.0021    0.0083   -0.0091

FREQ(CM**-1)     412.23    425.77    475.74    478.89    491.89    496.20

AT.    1 CA X     0.0002   -0.0022    0.0015    0.0029   -0.0031    0.0006
            Y    -0.0033    0.0017    0.0019   -0.0029   -0.0003    0.0018
            Z    -0.0013    0.0013    0.0006    0.0023   -0.0025   -0.0006
AT.    2 CA X     0.0002   -0.0022    0.0015   -0.0029    0.0031    0.0006
            Y     0.0033   -0.0017   -0.0019   -0.0029   -0.0003   -0.0018
            Z    -0.0013    0.0013    0.0006   -0.0023    0.0025   -0.0006
AT.    3 CA X    -0.0002   -0.0022    0.0015    0.0029    0.0031    0.0006
```

	Y	0.0033	0.0017	0.0019	-0.0029	0.0003	0.0018
	Z	0.0013	0.0013	0.0006	0.0023	0.0025	-0.0006
AT. 4 CA	X	-0.0002	-0.0022	0.0015	-0.0029	-0.0031	0.0006
	Y	-0.0033	-0.0017	-0.0019	-0.0029	0.0003	-0.0018
	Z	0.0013	0.0013	0.0006	-0.0023	-0.0025	-0.0006
AT. 5 CA	X	-0.0022	-0.0010	0.0021	-0.0009	-0.0018	-0.0038
	Y	-0.0033	-0.0045	0.0003	-0.0009	-0.0009	-0.0009
	Z	-0.0063	0.0007	0.0029	0.0012	-0.0001	0.0027
AT. 6 CA	X	-0.0022	-0.0010	0.0021	0.0009	0.0018	-0.0038
	Y	0.0033	0.0045	-0.0003	-0.0009	-0.0009	0.0009
	Z	-0.0063	0.0007	0.0029	-0.0012	0.0001	0.0027
AT. 7 CA	X	0.0022	-0.0010	0.0021	-0.0009	0.0018	-0.0038
	Y	0.0033	-0.0045	0.0003	-0.0009	0.0009	-0.0009
	Z	0.0063	0.0007	0.0029	0.0012	0.0001	0.0027
AT. 8 CA	X	0.0022	-0.0010	0.0021	0.0009	-0.0018	-0.0038
	Y	-0.0033	0.0045	-0.0003	-0.0009	0.0009	0.0009
	Z	0.0063	0.0007	0.0029	-0.0012	-0.0001	0.0027
AT. 9 SI	X	0.0012	0.0048	-0.0179	-0.0236	-0.0157	0.0177
	Y	0.0029	-0.0090	-0.0069	0.0036	0.0021	-0.0024
	Z	0.0089	0.0016	-0.0193	-0.0057	-0.0156	-0.0166
AT. 10 SI	X	0.0012	0.0048	-0.0179	0.0236	0.0157	0.0177
	Y	-0.0029	0.0090	0.0069	0.0036	0.0021	0.0024
	Z	0.0089	0.0016	-0.0193	0.0057	0.0156	-0.0166
AT. 11 SI	X	-0.0012	0.0048	-0.0179	-0.0236	0.0157	0.0177
	Y	-0.0029	-0.0090	-0.0069	0.0036	-0.0021	-0.0024
	Z	-0.0089	0.0016	-0.0193	-0.0057	0.0156	-0.0166
AT. 12 SI	X	-0.0012	0.0048	-0.0179	0.0236	-0.0157	0.0177
	Y	0.0029	0.0090	0.0069	0.0036	-0.0021	0.0024
	Z	-0.0089	0.0016	-0.0193	0.0057	-0.0156	-0.0166
AT. 13 O	X	0.0177	-0.0079	0.0093	0.0109	0.0202	-0.0077
	Y	0.0113	-0.0117	-0.0063	0.0095	0.0034	-0.0109
	Z	-0.0354	0.0263	0.0180	0.0074	0.0192	0.0165
AT. 14 O	X	0.0177	-0.0079	0.0093	-0.0109	-0.0202	-0.0077
	Y	-0.0113	0.0117	0.0063	0.0095	0.0034	0.0109
	Z	-0.0354	0.0263	0.0180	-0.0074	-0.0192	0.0165
AT. 15 O	X	-0.0177	-0.0079	0.0093	0.0109	-0.0202	-0.0077
	Y	-0.0113	-0.0117	-0.0063	0.0095	-0.0034	-0.0109
	Z	0.0354	0.0263	0.0180	0.0074	-0.0192	0.0165
AT. 16 O	X	-0.0177	-0.0079	0.0093	-0.0109	0.0202	-0.0077
	Y	0.0113	0.0117	0.0063	0.0095	-0.0034	0.0109
	Z	0.0354	0.0263	0.0180	-0.0074	0.0192	0.0165
AT. 17 O	X	-0.0034	0.0120	-0.0194	-0.0085	-0.0165	-0.0051
	Y	-0.0026	0.0076	-0.0132	-0.0168	-0.0174	0.0046
	Z	0.0269	-0.0295	0.0003	-0.0090	0.0084	0.0245
AT. 18 O	X	-0.0034	0.0120	-0.0194	0.0085	0.0165	-0.0051
	Y	0.0026	-0.0076	0.0132	-0.0168	-0.0174	-0.0046
	Z	0.0269	-0.0295	0.0003	0.0090	-0.0084	0.0245
AT. 19 O	X	0.0034	0.0120	-0.0194	-0.0085	0.0165	-0.0051
	Y	0.0026	0.0076	-0.0132	-0.0168	0.0174	0.0046
	Z	-0.0269	-0.0295	0.0003	-0.0090	-0.0084	0.0245
AT. 20 O	X	0.0034	0.0120	-0.0194	0.0085	-0.0165	-0.0051
	Y	-0.0026	-0.0076	0.0132	-0.0168	0.0174	-0.0046
	Z	-0.0269	-0.0295	0.0003	0.0090	0.0084	0.0245
AT. 21 O	X	0.0076	-0.0169	0.0141	0.0017	0.0176	0.0190
	Y	0.0315	-0.0286	-0.0015	-0.0012	0.0027	-0.0066
	Z	0.0056	-0.0189	0.0188	0.0295	0.0225	-0.0168
AT. 22 O	X	0.0076	-0.0169	0.0141	-0.0017	-0.0176	0.0190
	Y	-0.0315	0.0286	0.0015	-0.0012	0.0027	0.0066
	Z	0.0056	-0.0189	0.0188	-0.0295	-0.0225	-0.0168
AT. 23 O	X	-0.0076	-0.0169	0.0141	0.0017	-0.0176	0.0190
	Y	-0.0315	-0.0286	-0.0015	-0.0012	-0.0027	-0.0066
	Z	-0.0056	-0.0189	0.0188	0.0295	-0.0225	-0.0168
AT. 24 O	X	-0.0076	-0.0169	0.0141	-0.0017	0.0176	0.0190
	Y	0.0315	0.0286	0.0015	-0.0012	-0.0027	0.0066
	Z	-0.0056	-0.0189	0.0188	-0.0295	0.0225	-0.0168
AT. 25 O	X	-0.0183	0.0126	0.0183	0.0288	0.0095	-0.0291
	Y	-0.0197	0.0191	0.0239	0.0117	0.0123	0.0076
	Z	0.0023	0.0144	-0.0121	-0.0166	-0.0203	-0.0004
AT. 26 O	X	-0.0183	0.0126	0.0183	-0.0288	-0.0095	-0.0291
	Y	0.0197	-0.0191	-0.0239	0.0117	0.0123	-0.0076
	Z	0.0023	0.0144	-0.0121	0.0166	0.0203	-0.0004
AT. 27 O	X	0.0183	0.0126	0.0183	0.0288	-0.0095	-0.0291
	Y	0.0197	0.0191	0.0239	0.0117	-0.0123	0.0076
	Z	-0.0023	0.0144	-0.0121	-0.0166	0.0203	-0.0004
AT. 28 O	X	0.0183	0.0126	0.0183	-0.0288	0.0095	-0.0291
	Y	-0.0197	-0.0191	-0.0239	0.0117	-0.0123	-0.0076
	Z	-0.0023	0.0144	-0.0121	0.0166	-0.0203	-0.0004
FREQ(CM**-1)		498.82	505.31	514.90	517.73	522.56	523.37

AT. 1 CA	X	0.0004	-0.0034	-0.0046	-0.0017	0.0024	0.0006
	Y	-0.0081	0.0023	-0.0003	0.0009	0.0029	0.0089
	Z	-0.0010	-0.0002	-0.0025	0.0022	-0.0063	-0.0024
AT. 2 CA	X	-0.0004	-0.0034	-0.0046	0.0017	-0.0024	0.0006
	Y	-0.0081	-0.0023	0.0003	0.0009	0.0029	-0.0089
	Z	0.0010	-0.0002	-0.0025	-0.0022	0.0063	-0.0024
AT. 3 CA	X	0.0004	0.0034	0.0046	0.0017	0.0024	0.0006
	Y	-0.0081	-0.0023	0.0003	-0.0009	0.0029	0.0089
	Z	-0.0010	0.0002	0.0025	-0.0022	-0.0063	-0.0024
AT. 4 CA	X	-0.0004	0.0034	0.0046	-0.0017	-0.0024	0.0006
	Y	-0.0081	0.0023	-0.0003	-0.0009	0.0029	-0.0089
	Z	0.0010	0.0002	0.0025	0.0022	0.0063	-0.0024
AT. 5 CA	X	-0.0023	0.0006	0.0072	-0.0003	0.0006	-0.0016
	Y	0.0003	0.0048	-0.0019	0.0009	0.0010	0.0005
	Z	0.0015	-0.0068	-0.0003	0.0000	0.0016	0.0009
AT. 6 CA	X	0.0023	0.0006	0.0072	0.0003	-0.0006	-0.0016
	Y	0.0003	-0.0048	0.0019	0.0009	0.0010	-0.0005
	Z	-0.0015	-0.0068	-0.0003	-0.0000	-0.0016	0.0009
AT. 7 CA	X	-0.0023	-0.0006	-0.0072	0.0003	0.0006	-0.0016
	Y	0.0003	-0.0048	0.0019	-0.0009	0.0010	0.0005
	Z	0.0015	0.0068	0.0003	-0.0000	0.0016	0.0009
AT. 8 CA	X	0.0023	-0.0006	-0.0072	-0.0003	-0.0006	-0.0016
	Y	0.0003	0.0048	-0.0019	-0.0009	0.0010	-0.0005
	Z	-0.0015	0.0068	0.0003	0.0000	-0.0016	0.0009
AT. 9 SI	X	0.0043	-0.0039	-0.0190	-0.0142	0.0044	0.0032
	Y	0.0244	0.0009	-0.0022	-0.0052	-0.0104	-0.0228
	Z	-0.0051	-0.0176	0.0036	0.0136	-0.0138	0.0049
AT. 10 SI	X	-0.0043	-0.0039	-0.0190	0.0142	-0.0044	0.0032
	Y	0.0244	-0.0009	0.0022	-0.0052	-0.0104	0.0228
	Z	0.0051	-0.0176	0.0036	-0.0136	0.0138	0.0049
AT. 11 SI	X	0.0043	0.0039	0.0190	0.0142	0.0044	0.0032
	Y	0.0244	-0.0009	0.0022	0.0052	-0.0104	-0.0228
	Z	-0.0051	0.0176	-0.0036	-0.0136	-0.0138	0.0049
AT. 12 SI	X	-0.0043	0.0039	0.0190	-0.0142	-0.0044	0.0032
	Y	0.0244	0.0009	-0.0022	0.0052	-0.0104	0.0228
	Z	0.0051	0.0176	-0.0036	0.0136	0.0138	0.0049
AT. 13 O	X	-0.0051	0.0056	0.0269	0.0198	-0.0025	0.0020
	Y	0.0187	-0.0057	0.0079	0.0059	-0.0148	-0.0199
	Z	0.0137	0.0179	-0.0051	-0.0158	0.0237	-0.0141
AT. 14 O	X	0.0051	0.0056	0.0269	-0.0198	0.0025	0.0020
	Y	0.0187	0.0057	-0.0079	0.0059	-0.0148	0.0199
	Z	-0.0137	0.0179	-0.0051	0.0158	-0.0237	-0.0141
AT. 15 O	X	-0.0051	-0.0056	-0.0269	-0.0198	-0.0025	0.0020
	Y	0.0187	0.0057	-0.0079	-0.0059	-0.0148	-0.0199
	Z	0.0137	-0.0179	0.0051	0.0158	0.0237	-0.0141
AT. 16 O	X	0.0051	-0.0056	-0.0269	0.0198	0.0025	0.0020
	Y	0.0187	-0.0057	0.0079	-0.0059	-0.0148	0.0199
	Z	-0.0137	-0.0179	0.0051	-0.0158	-0.0237	-0.0141
AT. 17 O	X	0.0129	-0.0205	-0.0091	0.0009	-0.0199	-0.0170
	Y	-0.0138	-0.0100	-0.0104	-0.0017	0.0021	0.0178
	Z	0.0169	0.0250	-0.0175	-0.0293	0.0227	-0.0042
AT. 18 O	X	-0.0129	-0.0205	-0.0091	-0.0009	0.0199	-0.0170
	Y	-0.0138	0.0100	0.0104	-0.0017	0.0021	-0.0178
	Z	-0.0169	0.0250	-0.0175	0.0293	-0.0227	-0.0042
AT. 19 O	X	0.0129	0.0205	0.0091	-0.0009	-0.0199	-0.0170
	Y	-0.0138	0.0100	0.0104	0.0017	0.0021	0.0178
	Z	0.0169	-0.0250	0.0175	0.0293	0.0227	-0.0042
AT. 20 O	X	-0.0129	0.0205	0.0091	0.0009	0.0199	-0.0170
	Y	-0.0138	-0.0100	-0.0104	0.0017	0.0021	-0.0178
	Z	-0.0169	-0.0250	0.0175	-0.0293	-0.0227	-0.0042
AT. 21 O	X	0.0012	0.0287	-0.0060	-0.0167	0.0258	0.0055
	Y	-0.0227	0.0007	0.0070	0.0076	0.0001	0.0269
	Z	-0.0010	0.0107	0.0220	0.0104	0.0017	-0.0049
AT. 22 O	X	-0.0012	0.0287	-0.0060	0.0167	-0.0258	0.0055
	Y	-0.0227	-0.0007	-0.0070	0.0076	0.0001	-0.0269
	Z	0.0010	0.0107	0.0220	-0.0104	-0.0017	-0.0049
AT. 23 O	X	0.0012	-0.0287	0.0060	0.0167	0.0258	0.0055
	Y	-0.0227	-0.0007	-0.0070	-0.0076	0.0001	0.0269
	Z	-0.0010	-0.0107	-0.0220	-0.0104	0.0017	-0.0049
AT. 24 O	X	-0.0012	-0.0287	0.0060	-0.0167	-0.0258	0.0055
	Y	-0.0227	0.0007	0.0070	-0.0076	0.0001	-0.0269
	Z	0.0010	-0.0107	-0.0220	0.0104	-0.0017	-0.0049
AT. 25 O	X	-0.0187	-0.0055	0.0287	0.0295	-0.0077	0.0065
	Y	-0.0053	0.0157	0.0040	-0.0007	0.0208	0.0051
	Z	-0.0190	-0.0140	-0.0079	0.0057	-0.0058	0.0181
AT. 26 O	X	0.0187	-0.0055	0.0287	-0.0295	0.0077	0.0065
	Y	-0.0053	-0.0157	-0.0040	-0.0007	0.0208	-0.0051
	Z	0.0190	-0.0140	-0.0079	-0.0057	0.0058	0.0181

```
AT.  27 O   X    -0.0187    0.0055   -0.0287   -0.0295   -0.0077    0.0065
            Y    -0.0053   -0.0157   -0.0040    0.0007    0.0208    0.0051
            Z    -0.0190    0.0140    0.0079   -0.0057   -0.0058    0.0181
AT.  28 O   X     0.0187    0.0055   -0.0287    0.0295    0.0077    0.0065
            Y    -0.0053    0.0157    0.0040    0.0007    0.0208   -0.0051
            Z     0.0190    0.0140    0.0079    0.0057    0.0058    0.0181

FREQ(CM**-1)     533.58    544.83    819.78    822.18    823.25    832.53

AT.   1 CA X    -0.0011    0.0017   -0.0001    0.0000   -0.0004   -0.0004
            Y    -0.0103    0.0107   -0.0007   -0.0006   -0.0012   -0.0015
            Z     0.0002    0.0015   -0.0000   -0.0010   -0.0004    0.0011
AT.   2 CA X     0.0011    0.0017   -0.0001   -0.0000    0.0004   -0.0004
            Y    -0.0103   -0.0107    0.0007   -0.0006   -0.0012    0.0015
            Z    -0.0002    0.0015   -0.0000    0.0010    0.0004    0.0011
AT.   3 CA X     0.0011   -0.0017   -0.0001    0.0000    0.0004    0.0004
            Y     0.0103   -0.0107   -0.0007   -0.0006    0.0012    0.0015
            Z    -0.0002   -0.0015   -0.0000   -0.0010    0.0004   -0.0011
AT.   4 CA X    -0.0011   -0.0017   -0.0001   -0.0000   -0.0004    0.0004
            Y     0.0103    0.0107    0.0007   -0.0006    0.0012   -0.0015
            Z     0.0002   -0.0015   -0.0000    0.0010   -0.0004   -0.0011
AT.   5 CA X    -0.0013   -0.0008    0.0002   -0.0001   -0.0002    0.0004
            Y     0.0010    0.0014    0.0011    0.0002   -0.0003    0.0011
            Z    -0.0054    0.0010    0.0002    0.0008   -0.0003    0.0019
AT.   6 CA X     0.0013   -0.0008    0.0002    0.0001    0.0002    0.0004
            Y     0.0010   -0.0014   -0.0011    0.0002   -0.0003   -0.0011
            Z     0.0054    0.0010    0.0002   -0.0008    0.0003    0.0019
AT.   7 CA X     0.0013    0.0008    0.0002   -0.0001    0.0002   -0.0004
            Y    -0.0010   -0.0014    0.0011    0.0002    0.0003   -0.0011
            Z     0.0054   -0.0010    0.0002    0.0008    0.0003   -0.0019
AT.   8 CA X    -0.0013    0.0008    0.0002    0.0001   -0.0002   -0.0004
            Y    -0.0010    0.0014   -0.0011    0.0002    0.0003    0.0011
            Z    -0.0054   -0.0010    0.0002   -0.0008   -0.0003   -0.0019
AT.   9 SI X    -0.0025    0.0029   -0.0034   -0.0004   -0.0087   -0.0049
            Y     0.0205   -0.0185    0.0075    0.0081    0.0044    0.0040
            Z     0.0054   -0.0015   -0.0043   -0.0016   -0.0122   -0.0063
AT.  10 SI X     0.0025    0.0029   -0.0034    0.0004    0.0087   -0.0049
            Y     0.0205    0.0185   -0.0075    0.0081    0.0044   -0.0040
            Z    -0.0054   -0.0015   -0.0043    0.0016    0.0122   -0.0063
AT.  11 SI X     0.0025   -0.0029   -0.0034   -0.0004    0.0087    0.0049
            Y    -0.0205    0.0185    0.0075    0.0081   -0.0044   -0.0040
            Z    -0.0054    0.0015   -0.0043   -0.0016    0.0122    0.0063
AT.  12 SI X    -0.0025   -0.0029   -0.0034    0.0004   -0.0087    0.0049
            Y    -0.0205   -0.0185   -0.0075    0.0081   -0.0044    0.0040
            Z     0.0054    0.0015   -0.0043    0.0016   -0.0122    0.0063
AT.  13 O   X    -0.0017    0.0039   -0.0021   -0.0022   -0.0026   -0.0029
            Y     0.0248   -0.0217    0.0146    0.0136    0.0153    0.0199
            Z    -0.0052   -0.0046    0.0027    0.0028    0.0026    0.0024
AT.  14 O   X     0.0017    0.0039   -0.0021    0.0022    0.0026   -0.0029
            Y     0.0248    0.0217   -0.0146    0.0136    0.0153   -0.0199
            Z     0.0052   -0.0046    0.0027   -0.0028   -0.0026    0.0024
AT.  15 O   X     0.0017   -0.0039   -0.0021   -0.0022    0.0026    0.0029
            Y    -0.0248    0.0217    0.0146    0.0136   -0.0153   -0.0199
            Z     0.0052    0.0046    0.0027    0.0028   -0.0026   -0.0024
AT.  16 O   X    -0.0017   -0.0039   -0.0021    0.0022   -0.0026    0.0029
            Y    -0.0248   -0.0217   -0.0146    0.0136   -0.0153    0.0199
            Z    -0.0052    0.0046    0.0027   -0.0028    0.0026   -0.0024
AT.  17 O   X     0.0172   -0.0175   -0.0169   -0.0224   -0.0046   -0.0125
            Y    -0.0143    0.0145   -0.0090   -0.0121   -0.0023   -0.0086
            Z     0.0089   -0.0102   -0.0079   -0.0083   -0.0020   -0.0048
AT.  18 O   X    -0.0172   -0.0175   -0.0169    0.0224    0.0046   -0.0125
            Y    -0.0143   -0.0145    0.0090   -0.0121   -0.0023    0.0086
            Z    -0.0089   -0.0102   -0.0079    0.0083    0.0020   -0.0048
AT.  19 O   X    -0.0172    0.0175   -0.0169   -0.0224    0.0046    0.0125
            Y     0.0143   -0.0145   -0.0090   -0.0121    0.0023    0.0086
            Z    -0.0089    0.0102   -0.0079   -0.0083    0.0020    0.0048
AT.  20 O   X     0.0172    0.0175   -0.0169    0.0224   -0.0046    0.0125
            Y     0.0143    0.0145    0.0090   -0.0121    0.0023   -0.0086
            Z     0.0089    0.0102   -0.0079    0.0083   -0.0020    0.0048
AT.  21 O   X    -0.0123    0.0046    0.0173    0.0179    0.0138    0.0157
            Y    -0.0172    0.0248   -0.0033   -0.0028   -0.0037   -0.0052
            Z     0.0038   -0.0082   -0.0146   -0.0140   -0.0104   -0.0131
AT.  22 O   X     0.0123    0.0046    0.0173   -0.0179   -0.0138    0.0157
            Y    -0.0172   -0.0248    0.0033   -0.0028   -0.0037    0.0052
            Z    -0.0038   -0.0082   -0.0146    0.0140    0.0104   -0.0131
AT.  23 O   X     0.0123   -0.0046    0.0173    0.0179   -0.0138   -0.0157
            Y     0.0172   -0.0248   -0.0033   -0.0028    0.0037    0.0052
            Z    -0.0038    0.0082   -0.0146   -0.0140    0.0104    0.0131
AT.  24 O   X    -0.0123   -0.0046    0.0173   -0.0179    0.0138   -0.0157
```

	Y	0.0172	0.0248	0.0033	-0.0028	0.0037	-0.0052
	Z	0.0038	0.0082	-0.0146	0.0140	-0.0104	0.0131
AT. 25 O	X	0.0005	0.0037	0.0073	0.0076	0.0082	0.0079
	Y	-0.0181	0.0134	-0.0138	-0.0119	-0.0166	-0.0150
	Z	-0.0192	0.0226	0.0268	0.0229	0.0303	0.0265
AT. 26 O	X	-0.0005	0.0037	0.0073	-0.0076	-0.0082	0.0079
	Y	-0.0181	-0.0134	0.0138	-0.0119	-0.0166	0.0150
	Z	0.0192	0.0226	0.0268	-0.0229	-0.0303	0.0265
AT. 27 O	X	-0.0005	-0.0037	0.0073	0.0076	-0.0082	-0.0079
	Y	0.0181	-0.0134	-0.0138	-0.0119	0.0166	0.0150
	Z	0.0192	-0.0226	0.0268	0.0229	-0.0303	-0.0265
AT. 28 O	X	0.0005	-0.0037	0.0073	-0.0076	0.0082	-0.0079
	Y	0.0181	0.0134	0.0138	-0.0119	0.0166	-0.0150
	Z	-0.0192	-0.0226	0.0268	-0.0229	0.0303	-0.0265

FREQ(CM**-1)		834.32	842.59	852.70	859.88	879.25	880.69
AT. 1 CA	X	0.0009	-0.0001	0.0015	-0.0006	-0.0004	0.0000
	Y	-0.0010	-0.0001	-0.0008	-0.0001	0.0006	0.0002
	Z	0.0003	0.0006	0.0001	0.0008	0.0004	-0.0001
AT. 2 CA	X	-0.0009	-0.0001	0.0015	0.0006	0.0004	0.0000
	Y	-0.0010	0.0001	0.0008	-0.0001	0.0006	-0.0002
	Z	-0.0003	0.0006	0.0001	-0.0008	-0.0004	-0.0001
AT. 3 CA	X	-0.0009	-0.0001	-0.0015	-0.0006	0.0004	0.0000
	Y	0.0010	-0.0001	0.0008	-0.0001	-0.0006	0.0002
	Z	-0.0003	0.0006	-0.0001	0.0008	-0.0004	-0.0001
AT. 4 CA	X	0.0009	-0.0001	-0.0015	0.0006	-0.0004	0.0000
	Y	0.0010	0.0001	-0.0008	-0.0001	-0.0006	-0.0002
	Z	0.0003	0.0006	-0.0001	-0.0008	0.0004	-0.0001
AT. 5 CA	X	0.0003	-0.0000	-0.0011	0.0002	-0.0002	0.0008
	Y	0.0007	0.0002	-0.0010	0.0003	0.0002	0.0003
	Z	-0.0003	-0.0010	0.0011	-0.0003	-0.0001	-0.0004
AT. 6 CA	X	-0.0003	-0.0000	-0.0011	-0.0002	0.0002	0.0008
	Y	0.0007	-0.0002	0.0010	0.0003	0.0002	-0.0003
	Z	0.0003	-0.0010	0.0011	0.0003	0.0001	-0.0004
AT. 7 CA	X	-0.0003	-0.0000	0.0011	0.0002	0.0002	0.0008
	Y	-0.0007	0.0002	0.0010	0.0003	-0.0002	0.0003
	Z	0.0003	-0.0010	-0.0011	-0.0003	0.0001	-0.0004
AT. 8 CA	X	0.0003	-0.0000	0.0011	-0.0002	-0.0002	0.0008
	Y	-0.0007	-0.0002	-0.0010	0.0003	-0.0002	-0.0003
	Z	-0.0003	-0.0010	-0.0011	0.0003	-0.0001	-0.0004
AT. 9 SI	X	0.0123	0.0139	0.0168	0.0228	-0.0176	-0.0174
	Y	0.0031	0.0016	0.0013	0.0024	-0.0049	-0.0068
	Z	0.0140	0.0174	0.0147	0.0035	0.0147	0.0143
AT. 10 SI	X	-0.0123	0.0139	0.0168	-0.0228	0.0176	-0.0174
	Y	0.0031	-0.0016	-0.0013	0.0024	-0.0049	0.0068
	Z	-0.0140	0.0174	0.0147	-0.0035	-0.0147	0.0143
AT. 11 SI	X	-0.0123	0.0139	-0.0168	0.0228	0.0176	-0.0174
	Y	-0.0031	0.0016	-0.0013	0.0024	0.0049	-0.0068
	Z	-0.0140	0.0174	-0.0147	0.0035	-0.0147	0.0143
AT. 12 SI	X	0.0123	0.0139	-0.0168	-0.0228	-0.0176	-0.0174
	Y	-0.0031	-0.0016	0.0013	0.0024	0.0049	0.0068
	Z	0.0140	0.0174	-0.0147	-0.0035	0.0147	0.0143
AT. 13 O	X	-0.0025	-0.0002	-0.0024	0.0004	-0.0002	-0.0004
	Y	0.0108	0.0026	0.0067	-0.0005	0.0016	0.0051
	Z	0.0007	0.0007	0.0001	-0.0013	-0.0004	0.0009
AT. 14 O	X	0.0025	-0.0002	-0.0024	-0.0004	0.0002	-0.0004
	Y	0.0108	-0.0026	-0.0067	-0.0005	0.0016	-0.0051
	Z	-0.0007	0.0007	0.0001	0.0013	0.0004	0.0009
AT. 15 O	X	0.0025	-0.0002	0.0024	0.0004	0.0002	-0.0004
	Y	-0.0108	0.0026	-0.0067	-0.0005	-0.0016	0.0051
	Z	-0.0007	0.0007	-0.0001	-0.0013	0.0004	0.0009
AT. 16 O	X	-0.0025	-0.0002	0.0024	-0.0004	-0.0002	-0.0004
	Y	-0.0108	-0.0026	0.0067	-0.0005	-0.0016	-0.0051
	Z	0.0007	0.0007	-0.0001	0.0013	-0.0004	0.0009
AT. 17 O	X	-0.0294	-0.0249	-0.0261	-0.0225	0.0103	0.0103
	Y	-0.0162	-0.0144	-0.0142	-0.0116	0.0058	0.0057
	Z	-0.0123	-0.0105	-0.0128	-0.0106	0.0037	0.0049
AT. 18 O	X	0.0294	-0.0249	-0.0261	0.0225	-0.0103	0.0103
	Y	-0.0162	0.0144	0.0142	-0.0116	0.0058	-0.0057
	Z	0.0123	-0.0105	-0.0128	0.0106	-0.0037	0.0049
AT. 19 O	X	0.0294	-0.0249	0.0261	-0.0225	-0.0103	0.0103
	Y	0.0162	-0.0144	0.0142	-0.0116	-0.0058	0.0057
	Z	0.0123	-0.0105	0.0128	-0.0106	-0.0037	0.0049
AT. 20 O	X	-0.0294	-0.0249	0.0261	0.0225	0.0103	0.0103
	Y	0.0162	0.0144	-0.0142	-0.0116	-0.0058	-0.0057
	Z	-0.0123	-0.0105	0.0128	0.0106	0.0037	0.0049
AT. 21 O	X	0.0105	0.0039	0.0023	-0.0145	0.0224	0.0214
	Y	-0.0016	-0.0027	-0.0011	0.0029	-0.0039	-0.0041

	Z	-0.0092	-0.0028	-0.0031	0.0124	-0.0195	-0.0188
AT. 22 O	X	-0.0105	0.0039	0.0023	0.0145	-0.0224	0.0214
	Y	-0.0016	0.0027	0.0011	0.0029	-0.0039	0.0041
	Z	0.0092	-0.0028	-0.0031	-0.0124	0.0195	-0.0188
AT. 23 O	X	-0.0105	0.0039	-0.0023	-0.0145	-0.0224	0.0214
	Y	0.0016	-0.0027	0.0011	0.0029	0.0039	-0.0041
	Z	0.0092	-0.0028	0.0031	0.0124	0.0195	-0.0188
AT. 24 O	X	0.0105	0.0039	-0.0023	0.0145	0.0224	0.0214
	Y	0.0016	0.0027	-0.0011	0.0029	0.0039	0.0041
	Z	-0.0092	-0.0028	0.0031	-0.0124	-0.0195	-0.0188
AT. 25 O	X	-0.0018	-0.0029	-0.0043	-0.0020	-0.0025	-0.0030
	Y	0.0020	0.0097	0.0052	0.0046	0.0053	0.0051
	Z	-0.0036	-0.0166	-0.0124	-0.0096	-0.0096	-0.0107
AT. 26 O	X	0.0018	-0.0029	-0.0043	0.0020	0.0025	-0.0030
	Y	0.0020	-0.0097	-0.0052	0.0046	0.0053	-0.0051
	Z	0.0036	-0.0166	-0.0124	0.0096	0.0096	-0.0107
AT. 27 O	X	0.0018	-0.0029	0.0043	-0.0020	0.0025	-0.0030
	Y	-0.0020	0.0097	-0.0052	0.0046	-0.0053	0.0051
	Z	0.0036	-0.0166	0.0124	-0.0096	0.0096	-0.0107
AT. 28 O	X	-0.0018	-0.0029	0.0043	0.0020	-0.0025	-0.0030
	Y	-0.0020	-0.0097	0.0052	0.0046	-0.0053	-0.0051
	Z	-0.0036	-0.0166	0.0124	0.0096	-0.0096	-0.0107
FREQ(CM**-1)		898.52	933.16	963.14	963.29	980.43	980.45
AT. 1 CA	X	-0.0007	0.0000	-0.0002	0.0001	0.0000	-0.0003
	Y	0.0006	0.0003	0.0000	0.0005	-0.0005	-0.0008
	Z	-0.0001	0.0022	-0.0009	0.0002	0.0001	-0.0002
AT. 2 CA	X	-0.0007	-0.0000	0.0002	-0.0001	0.0000	-0.0003
	Y	-0.0006	0.0003	0.0000	0.0005	0.0005	0.0008
	Z	-0.0001	-0.0022	0.0009	-0.0002	0.0001	-0.0002
AT. 3 CA	X	0.0007	0.0000	-0.0002	-0.0001	0.0000	0.0003
	Y	-0.0006	0.0003	0.0000	-0.0005	-0.0005	0.0008
	Z	0.0001	0.0022	-0.0009	-0.0002	0.0001	0.0002
AT. 4 CA	X	0.0007	-0.0000	0.0002	0.0001	0.0000	0.0003
	Y	0.0006	0.0003	0.0000	-0.0005	0.0005	-0.0008
	Z	0.0001	-0.0022	0.0009	0.0002	0.0001	0.0002
AT. 5 CA	X	0.0004	0.0000	0.0003	0.0002	0.0004	0.0005
	Y	-0.0009	-0.0002	-0.0002	0.0000	-0.0014	-0.0004
	Z	0.0013	-0.0005	0.0000	0.0011	-0.0003	0.0012
AT. 6 CA	X	0.0004	-0.0000	-0.0003	-0.0002	0.0004	0.0005
	Y	0.0009	-0.0002	-0.0002	0.0000	0.0014	0.0004
	Z	0.0013	0.0005	-0.0000	-0.0011	-0.0003	0.0012
AT. 7 CA	X	-0.0004	0.0000	0.0003	-0.0002	0.0004	-0.0005
	Y	0.0009	-0.0002	-0.0002	-0.0000	-0.0014	0.0004
	Z	-0.0013	-0.0005	0.0000	-0.0011	-0.0003	-0.0012
AT. 8 CA	X	-0.0004	-0.0000	-0.0003	0.0002	0.0004	-0.0005
	Y	-0.0009	-0.0002	-0.0002	-0.0000	0.0014	-0.0004
	Z	-0.0013	0.0005	-0.0000	0.0011	-0.0003	-0.0012
AT. 9 SI	X	-0.0143	-0.0031	0.0044	0.0041	-0.0057	-0.0060
	Y	-0.0091	-0.0070	-0.0191	-0.0216	0.0196	0.0205
	Z	0.0159	0.0209	-0.0068	-0.0031	0.0040	0.0063
AT. 10 SI	X	-0.0143	0.0031	-0.0044	-0.0041	-0.0057	-0.0060
	Y	0.0091	-0.0070	-0.0191	-0.0216	-0.0196	-0.0205
	Z	0.0159	-0.0209	0.0068	0.0031	0.0040	0.0063
AT. 11 SI	X	0.0143	-0.0031	0.0044	-0.0041	-0.0057	0.0060
	Y	0.0091	-0.0070	-0.0191	0.0216	0.0196	-0.0205
	Z	-0.0159	0.0209	-0.0068	0.0031	0.0040	-0.0063
AT. 12 SI	X	0.0143	0.0031	-0.0044	0.0041	-0.0057	0.0060
	Y	-0.0091	-0.0070	-0.0191	0.0216	-0.0196	0.0205
	Z	-0.0159	-0.0209	0.0068	-0.0031	0.0040	-0.0063
AT. 13 O	X	-0.0008	-0.0016	-0.0049	-0.0060	0.0050	0.0059
	Y	0.0065	0.0087	0.0327	0.0306	-0.0327	-0.0284
	Z	0.0011	-0.0035	0.0055	0.0049	-0.0037	-0.0045
AT. 14 O	X	-0.0008	0.0016	0.0049	0.0060	0.0050	0.0059
	Y	-0.0065	0.0087	0.0327	0.0306	0.0327	0.0284
	Z	0.0011	0.0035	-0.0055	-0.0049	-0.0037	-0.0045
AT. 15 O	X	0.0008	-0.0016	-0.0049	0.0060	0.0050	-0.0059
	Y	-0.0065	0.0087	0.0327	-0.0306	-0.0327	0.0284
	Z	-0.0011	-0.0035	0.0055	-0.0049	-0.0037	0.0045
AT. 16 O	X	0.0008	0.0016	0.0049	-0.0060	0.0050	-0.0059
	Y	0.0065	0.0087	0.0327	-0.0306	0.0327	-0.0284
	Z	-0.0011	0.0035	-0.0055	0.0049	-0.0037	0.0045
AT. 17 O	X	0.0081	-0.0043	0.0042	0.0039	-0.0018	-0.0035
	Y	0.0065	-0.0023	0.0013	0.0031	0.0001	-0.0036
	Z	0.0010	-0.0058	0.0017	0.0022	-0.0007	-0.0033
AT. 18 O	X	0.0081	0.0043	-0.0042	-0.0039	-0.0018	-0.0035
	Y	-0.0065	-0.0023	0.0013	0.0031	-0.0001	0.0036
	Z	0.0010	0.0058	-0.0017	-0.0022	-0.0007	-0.0033

```
AT.  19 O  X    -0.0081   -0.0043    0.0042   -0.0039   -0.0018    0.0035
           Y    -0.0065   -0.0023    0.0013   -0.0031    0.0001    0.0036
           Z    -0.0010   -0.0058    0.0017   -0.0022   -0.0007    0.0033
AT.  20 O  X    -0.0081    0.0043   -0.0042    0.0039   -0.0018    0.0035
           Y     0.0065   -0.0023    0.0013   -0.0031   -0.0001   -0.0036
           Z    -0.0010    0.0058   -0.0017    0.0022   -0.0007    0.0033
AT.  21 O  X     0.0200    0.0152   -0.0060   -0.0042    0.0045    0.0068
           Y    -0.0021   -0.0030    0.0007    0.0013   -0.0016   -0.0037
           Z    -0.0195   -0.0148    0.0032    0.0032   -0.0041   -0.0082
AT.  22 O  X     0.0200   -0.0152    0.0060    0.0042    0.0045    0.0068
           Y     0.0021   -0.0030    0.0007    0.0013    0.0016    0.0037
           Z    -0.0195    0.0148   -0.0032   -0.0032   -0.0041   -0.0082
AT.  23 O  X    -0.0200    0.0152   -0.0060    0.0042    0.0045   -0.0068
           Y     0.0021   -0.0030    0.0007   -0.0013   -0.0016    0.0037
           Z     0.0195   -0.0148    0.0032   -0.0032   -0.0041    0.0082
AT.  24 O  X    -0.0200   -0.0152    0.0060   -0.0042    0.0045   -0.0068
           Y    -0.0021   -0.0030    0.0007   -0.0013    0.0016   -0.0037
           Z     0.0195    0.0148   -0.0032    0.0032   -0.0041    0.0082
AT.  25 O  X    -0.0019   -0.0034   -0.0007   -0.0009    0.0011    0.0010
           Y     0.0072    0.0086   -0.0010    0.0036   -0.0002   -0.0034
           Z    -0.0120   -0.0211    0.0023   -0.0047    0.0020    0.0028
AT.  26 O  X    -0.0019    0.0034    0.0007    0.0009    0.0011    0.0010
           Y    -0.0072    0.0086   -0.0010    0.0036    0.0002    0.0034
           Z    -0.0120    0.0211   -0.0023    0.0047    0.0020    0.0028
AT.  27 O  X     0.0019   -0.0034   -0.0007    0.0009    0.0011   -0.0010
           Y    -0.0072    0.0086   -0.0010   -0.0036   -0.0002    0.0034
           Z     0.0120   -0.0211    0.0023    0.0047    0.0020   -0.0028
AT.  28 O  X     0.0019    0.0034    0.0007   -0.0009    0.0011   -0.0010
           Y     0.0072    0.0086   -0.0010   -0.0036    0.0002   -0.0034
           Z     0.0120    0.0211   -0.0023   -0.0047    0.0020   -0.0028

*******************************************************************************

VIBRATIONAL TEMPERATURES (K) [MODE NUMBER;IRREP]

TO MODES
  146.1 [   4;AU ]   152.3 [   5;BU ]   153.9 [   6;BG ]   161.7 [   7;AG ]
  177.3 [   8;AU ]   202.2 [   9;AG ]   206.4 [  10;AU ]   212.9 [  11;BU ]
  220.0 [  12;AG ]   220.7 [  13;BG ]   230.4 [  14;BG ]   237.2 [  15;AG ]
  248.0 [  16;BG ]   248.4 [  17;BU ]   253.0 [  18;AG ]   258.1 [  19;AU ]
  267.4 [  20;AU ]   284.2 [  21;AG ]   285.4 [  22;BG ]   286.8 [  23;BU ]
  292.9 [  24;AG ]   307.3 [  25;BU ]   312.8 [  26;BG ]   314.8 [  27;AU ]
  324.9 [  28;AU ]   331.1 [  29;BU ]   345.6 [  30;AG ]   356.8 [  31;BG ]
  361.8 [  32;AG ]   363.1 [  33;BU ]   369.1 [  34;BG ]   370.3 [  35;AU ]
  380.7 [  36;BU ]   382.5 [  37;AG ]   390.8 [  38;AU ]   393.7 [  39;BG ]
  395.4 [  40;AU ]   400.5 [  41;AG ]   411.6 [  42;BG ]   411.6 [  43;BU ]
  422.0 [  44;BG ]   428.0 [  45;AU ]   436.1 [  46;AG ]   448.8 [  47;BG ]
  451.7 [  48;BU ]   511.6 [  49;AG ]   519.6 [  50;BU ]   532.9 [  51;AU ]
  547.1 [  52;AU ]   577.1 [  53;BG ]   588.6 [  54;AG ]   593.1 [  55;BG ]
  612.6 [  56;BU ]   684.5 [  57;BU ]   689.0 [  58;AU ]   707.7 [  59;AG ]
  713.9 [  60;BU ]   717.7 [  61;AU ]   727.0 [  62;BG ]   740.8 [  63;BG ]
  744.9 [  64;AG ]   751.8 [  65;AU ]   753.0 [  66;BU ]   767.7 [  67;AG ]
  783.9 [  68;BG ]  1179.5 [  69;BU ]  1182.9 [  70;AU ]  1184.5 [  71;AG ]
 1197.8 [  72;BG ]  1200.4 [  73;AG ]  1212.3 [  74;BU ]  1226.8 [  75;BG ]
 1237.2 [  76;AU ]  1265.0 [  77;AG ]  1267.1 [  78;BU ]  1292.8 [  79;BG ]
 1342.6 [  80;AU ]  1385.7 [  81;AU ]  1386.0 [  82;AG ]  1410.6 [  83;BU ]
 1410.7 [  84;BG ]

*******************************************************************************

HARMONIC VIBRATIONAL CONTRIBUTIONS TO THERMODYNAMIC FUNCTIONS AT GIVEN
TEMPERATURE AND PRESSURE:

(EL = ELECTRONIC ENERGY
 E0 = ZERO-POINT ENERGY
 ET = THERMAL CONTRIBUTION TO THE VIBRATIONAL ENERGY
 PV = PRESSURE * VOLUME
 TS = TEMPERATURE * ENTROPY)

                          AU/CELL             EV/CELL                 KJ/MOL
EL            :   -7757.783385138424 -211100.018027151498   -20368057.40730108
E0            :       0.075216382264       2.046741815747         197.48058380


*******************************************************************************

THERMODYNAMIC FUNCTIONS WITH VIBRATIONAL CONTRIBUTIONS

AT (T =  298.15 K, P =   0.10132500E+00 MPA):
```

```
                        AU/CELL             EV/CELL                 KJ/MOL
 ET            :       0.030654598144       0.834154023071           80.48363609
 PV            :       0.000008015160       0.000218103590            0.02104380
 TS            :       0.053690717498       1.460998698871          140.96495893
 ET+PV-TS      :      -0.023028104194      -0.626626572210          -60.46027904
 EL+E0+ET+PV-TS:   -7757.731196860354 -211098.597911907942   -20367920.38699631

 OTHER THERMODINAMIC FUNCTIONS:

                     mHARTREE/(CELL*K)     mEV/(CELL*K)              J/(MOL*K)
 ENTROPY       :       0.180079548880       4.900213647059          472.79878895
 HEAT CAPACITY :       0.185691404291       5.052919996244          487.53271326

 *******************************************************************************
 TTTTTTTTTTTTTTTTTTTTTTTTTTTTTT END         TELAPSE    17232.26 TCPU    17096.32
 EEEEEEEEEE TERMINATION  DATE 09 03 2019 TIME 15:35:26.5
NODE    0 CPU TIME =     17096.321
NODE    1 CPU TIME =     17171.029
NODE    2 CPU TIME =     17169.278
NODE    3 CPU TIME =     17172.342
NODE    4 CPU TIME =     17180.631
NODE    5 CPU TIME =     17169.118
NODE    6 CPU TIME =     17157.979
NODE    7 CPU TIME =     17184.305
    TOTAL CPU TIME =    137301.003
[proxy:0:0@lmfz640] got pmi command (from 29): finalize

[proxy:0:0@lmfz640] PMI response: cmd=finalize_ack
[proxy:0:0@lmfz640] got pmi command (from 21): finalize

[proxy:0:0@lmfz640] PMI response: cmd=finalize_ack
[proxy:0:0@lmfz640] got pmi command (from 0): finalize

[proxy:0:0@lmfz640] PMI response: cmd=finalize_ack
[proxy:0:0@lmfz640] got pmi command (from 6): finalize

[proxy:0:0@lmfz640] PMI response: cmd=finalize_ack
[proxy:0:0@lmfz640] got pmi command (from 13): finalize

[proxy:0:0@lmfz640] PMI response: cmd=finalize_ack
[proxy:0:0@lmfz640] got pmi command (from 17): finalize

[proxy:0:0@lmfz640] PMI response: cmd=finalize_ack
[proxy:0:0@lmfz640] got pmi command (from 25): finalize

[proxy:0:0@lmfz640] PMI response: cmd=finalize_ack
[proxy:0:0@lmfz640] got pmi command (from 8): finalize

[proxy:0:0@lmfz640] PMI response: cmd=finalize_ack
-----------------------------------------------
total 1502700
-rw-rw-r-- 1 vlasta vlasta         0 ožu   9 10:48 dffit3.dat
-rw-rw-r-- 1 vlasta vlasta      2156 ožu   9 15:34 ERROR
-rw-rw-r-- 1 vlasta vlasta      2156 ožu   9 15:34 ERROR.pe1
-rw-rw-r-- 1 vlasta vlasta      2156 ožu   9 15:34 ERROR.pe2
-rw-rw-r-- 1 vlasta vlasta      2156 ožu   9 15:34 ERROR.pe3
-rw-rw-r-- 1 vlasta vlasta      2156 ožu   9 15:34 ERROR.pe4
-rw-rw-r-- 1 vlasta vlasta      2156 ožu   9 15:34 ERROR.pe5
-rw-rw-r-- 1 vlasta vlasta      2156 ožu   9 15:34 ERROR.pe6
-rw-rw-r-- 1 vlasta vlasta      2156 ožu   9 15:34 ERROR.pe7
-rw-rw-r-- 1 vlasta vlasta     14144 ožu   9 15:35 fort.10.pe0
-rw-rw-r-- 1 vlasta vlasta 101154928 ožu   9 15:31 fort.10.pe1
-rw-rw-r-- 1 vlasta vlasta 101154928 ožu   9 15:31 fort.10.pe2
-rw-rw-r-- 1 vlasta vlasta  93929584 ožu   9 15:31 fort.10.pe3
-rw-rw-r-- 1 vlasta vlasta  93929584 ožu   9 15:31 fort.10.pe4
-rw-rw-r-- 1 vlasta vlasta 101154928 ožu   9 15:31 fort.10.pe5
-rw-rw-r-- 1 vlasta vlasta 101154928 ožu   9 15:31 fort.10.pe6
-rw-rw-r-- 1 vlasta vlasta  93929584 ožu   9 15:31 fort.10.pe7
-rw-rw-r-- 1 vlasta vlasta   1574184 ožu   9 15:31 fort.11.pe0
-rw-rw-r-- 1 vlasta vlasta   1574184 ožu   9 15:31 fort.11.pe1
-rw-rw-r-- 1 vlasta vlasta   1574184 ožu   9 15:31 fort.11.pe2
-rw-rw-r-- 1 vlasta vlasta   1574184 ožu   9 15:31 fort.11.pe3
-rw-rw-r-- 1 vlasta vlasta   1574184 ožu   9 15:31 fort.11.pe4
-rw-rw-r-- 1 vlasta vlasta   1574184 ožu   9 15:31 fort.11.pe5
-rw-rw-r-- 1 vlasta vlasta   1574184 ožu   9 15:31 fort.11.pe6
-rw-rw-r-- 1 vlasta vlasta   1574184 ožu   9 15:31 fort.11.pe7
-rw-rw-r-- 1 vlasta vlasta     51524 ožu   9 15:21 fort.12
-rw-rw-r-- 1 vlasta vlasta   3119304 ožu   9 10:57 fort.13
```

```
-rw-rw-r-- 1 vlasta vlasta         0 ožu   9 10:48 fort.17.pe1
-rw-rw-r-- 1 vlasta vlasta         0 ožu   9 10:48 fort.17.pe2
-rw-rw-r-- 1 vlasta vlasta         0 ožu   9 10:48 fort.17.pe3
-rw-rw-r-- 1 vlasta vlasta         0 ožu   9 10:48 fort.17.pe4
-rw-rw-r-- 1 vlasta vlasta         0 ožu   9 10:48 fort.17.pe5
-rw-rw-r-- 1 vlasta vlasta         0 ožu   9 10:48 fort.17.pe6
-rw-rw-r-- 1 vlasta vlasta         0 ožu   9 10:48 fort.17.pe7
-rw-rw-r-- 1 vlasta vlasta         0 ožu   9 10:48 fort.18.pe0
-rw-rw-r-- 1 vlasta vlasta         0 ožu   9 10:48 fort.18.pe1
-rw-rw-r-- 1 vlasta vlasta         0 ožu   9 10:48 fort.18.pe2
-rw-rw-r-- 1 vlasta vlasta         0 ožu   9 10:48 fort.18.pe3
-rw-rw-r-- 1 vlasta vlasta         0 ožu   9 10:48 fort.18.pe4
-rw-rw-r-- 1 vlasta vlasta         0 ožu   9 10:48 fort.18.pe5
-rw-rw-r-- 1 vlasta vlasta         0 ožu   9 10:48 fort.18.pe6
-rw-rw-r-- 1 vlasta vlasta         0 ožu   9 10:48 fort.18.pe7
-rw-rw-r-- 1 vlasta vlasta      4464 ožu   9 15:35 fort.19.pe0
-rw-rw-r-- 1 vlasta vlasta   1319712 ožu   9 15:35 fort.19.pe1
-rw-rw-r-- 1 vlasta vlasta   1319712 ožu   9 15:35 fort.19.pe2
-rw-rw-r-- 1 vlasta vlasta   1319712 ožu   9 15:35 fort.19.pe3
-rw-rw-r-- 1 vlasta vlasta   1319712 ožu   9 15:35 fort.19.pe4
-rw-rw-r-- 1 vlasta vlasta   1319712 ožu   9 15:35 fort.19.pe5
-rw-rw-r-- 1 vlasta vlasta   1319712 ožu   9 15:35 fort.19.pe6
-rw-rw-r-- 1 vlasta vlasta   1319712 ožu   9 15:35 fort.19.pe7
-rw-rw-r-- 1 vlasta vlasta         0 ožu   9 10:48 fort.1.pe0
-rw-rw-r-- 1 vlasta vlasta         0 ožu   9 10:48 fort.1.pe1
-rw-rw-r-- 1 vlasta vlasta         0 ožu   9 10:48 fort.1.pe2
-rw-rw-r-- 1 vlasta vlasta         0 ožu   9 10:48 fort.1.pe3
-rw-rw-r-- 1 vlasta vlasta         0 ožu   9 10:48 fort.1.pe4
-rw-rw-r-- 1 vlasta vlasta         0 ožu   9 10:48 fort.1.pe5
-rw-rw-r-- 1 vlasta vlasta         0 ožu   9 10:48 fort.1.pe6
-rw-rw-r-- 1 vlasta vlasta         0 ožu   9 10:48 fort.1.pe7
-rw-rw-r-- 1 vlasta vlasta   1257308 ožu   9 10:57 fort.20
-rw-rw-r-- 1 vlasta vlasta         0 ožu   9 10:48 fort.28
-rw-rw-r-- 1 vlasta vlasta         0 ožu   9 10:48 fort.29
-rw-rw-r-- 1 vlasta vlasta         0 ožu   9 10:48 fort.38.pe0
-rw-rw-r-- 1 vlasta vlasta         0 ožu   9 10:48 fort.38.pe1
-rw-rw-r-- 1 vlasta vlasta         0 ožu   9 10:48 fort.38.pe2
-rw-rw-r-- 1 vlasta vlasta         0 ožu   9 10:48 fort.38.pe3
-rw-rw-r-- 1 vlasta vlasta         0 ožu   9 10:48 fort.38.pe4
-rw-rw-r-- 1 vlasta vlasta         0 ožu   9 10:48 fort.38.pe5
-rw-rw-r-- 1 vlasta vlasta         0 ožu   9 10:48 fort.38.pe6
-rw-rw-r-- 1 vlasta vlasta         0 ožu   9 10:48 fort.38.pe7
-rw-rw-r-- 1 vlasta vlasta         0 ožu   9 10:48 fort.3.pe0
-rw-rw-r-- 1 vlasta vlasta         0 ožu   9 10:48 fort.3.pe1
-rw-rw-r-- 1 vlasta vlasta         0 ožu   9 10:48 fort.3.pe2
-rw-rw-r-- 1 vlasta vlasta         0 ožu   9 10:48 fort.3.pe3
-rw-rw-r-- 1 vlasta vlasta         0 ožu   9 10:48 fort.3.pe4
-rw-rw-r-- 1 vlasta vlasta         0 ožu   9 10:48 fort.3.pe5
-rw-rw-r-- 1 vlasta vlasta         0 ožu   9 10:48 fort.3.pe6
-rw-rw-r-- 1 vlasta vlasta         0 ožu   9 10:48 fort.3.pe7
-rw-rw-r-- 1 vlasta vlasta   1680640 ožu   9 15:34 fort.40.pe0
-rw-rw-r-- 1 vlasta vlasta   1680640 ožu   9 15:34 fort.40.pe1
-rw-rw-r-- 1 vlasta vlasta   1680640 ožu   9 15:34 fort.40.pe2
-rw-rw-r-- 1 vlasta vlasta   1680640 ožu   9 15:34 fort.40.pe3
-rw-rw-r-- 1 vlasta vlasta   1680640 ožu   9 15:34 fort.40.pe4
-rw-rw-r-- 1 vlasta vlasta   1680640 ožu   9 15:34 fort.40.pe5
-rw-rw-r-- 1 vlasta vlasta   1680640 ožu   9 15:34 fort.40.pe6
-rw-rw-r-- 1 vlasta vlasta   1680640 ožu   9 15:34 fort.40.pe7
-rw-rw-r-- 1 vlasta vlasta  93929584 ožu   9 15:31 fort.8.pe0
-rw-rw-r-- 1 vlasta vlasta 101154928 ožu   9 15:31 fort.8.pe1
-rw-rw-r-- 1 vlasta vlasta 101154928 ožu   9 15:31 fort.8.pe2
-rw-rw-r-- 1 vlasta vlasta  93929584 ožu   9 15:31 fort.8.pe3
-rw-rw-r-- 1 vlasta vlasta  93929584 ožu   9 15:31 fort.8.pe4
-rw-rw-r-- 1 vlasta vlasta 101154928 ožu   9 15:31 fort.8.pe5
-rw-rw-r-- 1 vlasta vlasta 101154928 ožu   9 15:31 fort.8.pe6
-rw-rw-r-- 1 vlasta vlasta  93929584 ožu   9 15:31 fort.8.pe7
-rw-rw-r-- 1 vlasta vlasta   1257308 ožu   9 10:57 fort.9
-rw-rw-r-- 1 vlasta vlasta   1615000 ožu   9 15:31 fort.95.pe0
-rw-rw-r-- 1 vlasta vlasta   1615000 ožu   9 15:31 fort.95.pe1
-rw-rw-r-- 1 vlasta vlasta   1615000 ožu   9 15:31 fort.95.pe2
-rw-rw-r-- 1 vlasta vlasta   1615000 ožu   9 15:31 fort.95.pe3
-rw-rw-r-- 1 vlasta vlasta   1615000 ožu   9 15:31 fort.95.pe4
-rw-rw-r-- 1 vlasta vlasta   1615000 ožu   9 15:31 fort.95.pe5
-rw-rw-r-- 1 vlasta vlasta   1615000 ožu   9 15:31 fort.95.pe6
-rw-rw-r-- 1 vlasta vlasta   1615000 ožu   9 15:31 fort.95.pe7
-rw-rw-r-- 1 vlasta vlasta   5975515 ožu   9 15:35 FREQINFO.DAT
-rw-rw-r-- 1 vlasta vlasta    149940 ožu   9 15:35 HESSFREQ.DAT
-rw-rw-r-- 1 vlasta vlasta      2143 ožu   9 10:48 INPUT
-rw-rw-r-- 1 vlasta vlasta        64 ožu   9 10:48 machines
```

```
-rwxr-xr-x 1 vlasta vlasta  11332453 ožu   9 10:48 Pcrystal
-rw-rw-r-- 1 vlasta vlasta    398603 ožu   9 15:35 SCFOUT.LOG
-----------------------------------------------
=:= Removing scratch directories on:  ...
=:= finished sub, 9.03.2019. 15:35:26 CET
=:= Elapsed time: 4h:47m:12s
```

```
OUTPUT OF CRYSTAL09 PROGRAM FOR Ca3SiO5 alite
=================================================================
=:= date: pet, 11.10.2019. 14:14:25 CEST
=:= hostname: lmfz640
=:= system: Linux lmfz640 5.0.0-31-generic #33~18.04.1-Ubuntu SMP Tue Oct 1 10:20:39 UTC 2019 x86_64 x86_64
x86_64 GNU/Linux
=:= user: vlasta
=:= scratch directory: /home/vlasta/crystal/tmp/tmp2648
=:= Available predefined $Pcrystal_ENV={isis,neolith,kalkyl,lenngren,interactive,localhost}
=:= $Pcrystal_ENV not defined by user. Trying to guess...
=:= None of the predefined parallel enviroments matched. Configuring "localhost"...
=:= Using predefined environment: localhost
=:= mpirun to use: /usr/bin/mpirun.mpich
=:= Got NSLOTS=8
=:= ==================================================================
Ca3SiO5 Cm  81100 ICSD W G Mumme LDA i VWN PP
CRYSTAL
0 0 0
 8
 12.235  7.073   9.298 116.31
  27
   20    2.785434612087E-01  1.246419190595E-17  3.006880006403E-01
   20    1.531537006405E-02  2.585068888852E-01  3.338407517012E-01
   20    1.531537006405E-02 -2.585068888852E-01  3.338407517012E-01
   20   -2.413293694602E-01 -2.359161832691E-01 -2.038417046735E-02
   20   -2.413293694602E-01  2.359161832691E-01 -2.038417046735E-02
   20   -3.032353025404E-01 -1.112570498310E-17 -3.537485816856E-01
   20    2.760504695417E-02  5.000000000000E-01  2.002234093501E-02
   20   -1.864937213094E-02 -2.762003324293E-01 -3.065722468464E-01
   20   -1.864937213094E-02  2.762003324293E-01 -3.065722468464E-01
   14   -2.804944480822E-01 -5.000000000000E-01 -3.483602425146E-01
   14    2.866911555732E-01  5.000000000000E-01  3.584634624474E-01
   14    1.563117691256E-03  7.236497377012E-19  1.317167878642E-03
    8    1.799435653619E-01 -4.321182612850E-18  4.596840795229E-01
    8    3.146382415874E-01  3.135397453095E-01  2.746504196733E-01
    8    3.146382415874E-01 -3.135397453095E-01  2.746504196733E-01
    8    1.416369842747E-01  5.000000000000E-01  3.242115389560E-01
    8   -1.202960000615E-01  5.000000000000E-01  1.158423931343E-01
    8   -1.323967604115E-01  3.626286874279E-18 -4.442461060711E-01
    8   -1.316127431580E-01 -5.000000000000E-01 -2.417309932451E-01
    8   -3.379350013612E-01 -3.099994556439E-01 -3.067690551272E-01
    8   -3.379350013612E-01  3.099994556439E-01 -3.067690551272E-01
    8    4.801992063025E-01 -3.310750185887E-17  4.956578249650E-01
    8    1.247353671791E-01  5.000000000000E-01 -1.435912966766E-01
    8    2.507864841237E-03  1.841554408899E-19  1.768212209225E-01
    8   -1.429906011707E-01  2.018186860997E-18 -1.219030917026E-01
```

```
    8    7.001017431963E-02  1.873620964908E-01 -2.362955768687E-02
    8    7.001017431963E-02 -1.873620964908E-01 -2.362955768687E-02
FREQCALC
END
END
20 7
0 0 8 2. 1.
 191300. 0.0002204
 26970. 0.001925
 5696. 0.01109
 1489.4 0.04995
 448.3 0.17014
 154.62 0.3685
 60.37 0.4034
 25.09 0.1452
0 1 6 8. 1.
 448.6 -0.00575 0.00847
 105.7 -0.0767 0.06027
 34.69 -0.1122 0.2124
 13.50 0.2537 0.3771
 5.820 0.688 0.401
 1.819 0.349 0.198
0 1 5 8. 1.
 20.75 -0.0020 -0.0365
 8.40 -0.1255 -0.0685
 3.597 -0.6960 0.1570
 1.408 1.029 1.482
 0.726 0.944 1.025
0 1 1 2. 1.
 0.453 1. 1.
0 1 1 0. 1.
 0.295 1. 1.
0 3 2 0. 1.
 3.1910 0.160
 0.8683 0.313
0 3 1 0. 1.
 0.2891 0.406
14 6
0 0 8 2. 1.0
 87645.8 0.000237
 12851.8 0.00192
 2786.28 0.0109
 728.043 0.0496
 219.516 0.1668
 75.9006 0.363
 29.4602 0.4051
 11.9891 0.1504
0 1 6 8. 1.0
 165.958 -0.00884 0.00909
 39.3727 -0.0859 0.0601
 12.7112 -0.0712 0.1952
 4.777 0.4147 0.3384
 1.8482 0.6168 0.3006
 0.7365 0.1154 0.0648
0 1 3 4. 1.
 4.1752 -0.0199 -0.0087
 1.4472 -0.1864 -0.00438
 0.5023 0.0967 0.2207
0 1 1 0. 1.
 0.322 1. 1.
0 1 1 0. 1.
 0.13 1. 1.
0 3 1 0. 1.
 0.6 1.
8 6
0 0 8 2.0 1.0
 8020. 0.001080
 1338. 0.008040
 255.4 0.053240
 69.22 0.168100
 23.90 0.358100
 9.264 0.385500
 3.851 0.146800
 1.212 0.072800
0 1 4 6. 1.0
 49.43 -0.008830 0.009580
 10.47 -0.091500 0.069600
 3.235 -0.040200 0.206500
 1.217 0.379000 0.347000
```

```
0 1 1 0. 1.0
 0.486 1. 1.
0 1 1 0. 1.0
 0.1925 1. 1.
0 3 1 0. 1.
 2.0 1.
0 3 1 0. 1.
 0.500 1.
99 0
ENDBS
SCFDIR
DFT
CORRELAT
VWN
EXCHANGE
LDA
XLGRID
END
SHRINK
 4 4 4
TOLINTEG
6 6 6 6 12
FMIXING
75
MAXCYCLE
 800
LEVSHIFT
6 1
TOLDEE
10
END
=:= Executing: /usr/bin/mpirun.mpich -np 8 -v -machinefile /home/vlasta/crystal/tmp/tmp2648/machines
/home/vlasta/crystal/tmp/tmp2648/Pcrystal
host: lmfz640

==================================================================================================
mpiexec options:
----------------
  Base path: /usr/bin/
  Launcher: (null)
  Debug level: 1
  Enable X: -1

  Global environment:
  -------------------
    EXCITINGVISUAL=/usr/share/exciting/xml/visualizationtemplates
    LS_COL-
ORS=rs=0:di=01;34:ln=01;36:mh=00:pi=40;33:so=01;35:do=01;35:bd=40;33;01:cd=40;33;01:or=40;31;01:mi=00:su=37;4
1:sg=30;43:ca=30;41:tw=30;42:ow=34;42:st=37;44:ex=01;32:*.tar=01;31:*.tgz=01;31:*.arc=01;31:*.arj=01;31:*.taz
=01;31:*.lha=01;31:*.lz4=01;31:*.lzh=01;31:*.lzma=01;31:*.tlz=01;31:*.txz=01;31:*.tzo=01;31:*.t7z=01;31:*.zip
=01;31:*.z=01;31:*.Z=01;31:*.dz=01;31:*.gz=01;31:*.lrz=01;31:*.lz=01;31:*.lzo=01;31:*.xz=01;31:*.zst=01;31:*.
tzst=01;31:*.bz2=01;31:*.bz=01;31:*.tbz=01;31:*.tbz2=01;31:*.tz=01;31:*.deb=01;31:*.rpm=01;31:*.jar=01;31:*.w
ar=01;31:*.ear=01;31:*.sar=01;31:*.rar=01;31:*.alz=01;31:*.ace=01;31:*.zoo=01;31:*.cpio=01;31:*.7z=01;31:*.rz
=01;31:*.cab=01;31:*.wim=01;31:*.swm=01;31:*.dwm=01;31:*.esd=01;31:*.jpg=01;35:*.jpeg=01;35:*.mjpg=01;35:*.mj
peg=01;35:*.gif=01;35:*.bmp=01;35:*.pbm=01;35:*.pgm=01;35:*.ppm=01;35:*.tga=01;35:*.xbm=01;35:*.xpm=01;35:*.t
if=01;35:*.tiff=01;35:*.png=01;35:*.svg=01;35:*.svgz=01;35:*.mng=01;35:*.pcx=01;35:*.mov=01;35:*.mpg=01;35:*.
mpeg=01;35:*.m2v=01;35:*.mkv=01;35:*.webm=01;35:*.ogm=01;35:*.mp4=01;35:*.m4v=01;35:*.mp4v=01;35:*.vob=01;35:
*.qt=01;35:*.nuv=01;35:*.wmv=01;35:*.asf=01;35:*.rm=01;35:*.rmvb=01;35:*.flc=01;35:*.avi=01;35:*.fli=01;35:*.
flv=01;35:*.gl=01;35:*.dl=01;35:*.xcf=01;35:*.xwd=01;35:*.yuv=01;35:*.cgm=01;35:*.emf=01;35:*.ogv=01;35:*.ogx
=01;35:*.aac=00;36:*.au=00;36:*.flac=00;36:*.m4a=00;36:*.mid=00;36:*.midi=00;36:*.mka=00;36:*.mp3=00;36:*.mpc
=00;36:*.ogg=00;36:*.ra=00;36:*.wav=00;36:*.oga=00;36:*.opus=00;36:*.spx=00;36:*.xspf=00;36:
    LD_LIBRARY_PATH=/usr/local/gaus/g16/bsd:/usr/local/gaus/g16:/usr/local/gaus/gv/lib
    GPAW_SETUP_PATH=/usr/share/gpaw-setups
    GAUSS_BSDDIR=/usr/local/gaus/g16/bsd
    GRA6_EXEDIR=/home/vlasta/crystal/crgra2006/bin/Linux-pgf
    CMR_REPOSITORY=/home/vlasta/cmr
    LC_MEASUREMENT=hr_HR.UTF-8
    SSH_CONNECTION=10.135.24.32 50621 10.135.24.38 22
    LESSCLOSE=/usr/bin/lesspipe %s %s
    LC_PAPER=hr_HR.UTF-8
    LC_MONETARY=hr_HR.UTF-8
    LANG=en_US.UTF-8
    g16root=/usr/local/gaus
    PSEUDO_DIR=/home/vlasta/md/potentials/UPF
    CRY2K6_GRA=/home/vlasta/crystal/crgra2006
    _DSM_BARRIER=SHM
    DACAPOPATH=/usr/share/dacapo-psp
    OPENBLAS_NUM_THREADS=1
    OLDPWD=/home/vlasta/crystal/tmp/c3s
```

```
    XCRYSDEN_TOPDIR=/usr/local/share/XCrySDen/
    TMP_DIR=/home/vlasta/crystal/tmp/tmp2648
    EXCITINGATAT=/home/vlasta/md/run/excitingscripts/ATAT@exciting
    GV_DIR=/usr/local/gaus/gv
    DACAPOEXE_SERIAL=/usr/bin/dacapo_serial.run
    CRY2K9_SCRDIR=/home/vlasta/crystal/tmp
    SIESTA_SCRIPT=/home/vlasta/bin/run_siesta.py
    LC_NAME=hr_HR.UTF-8
    GAUSS_LEXEDIR=/usr/local/gaus/g16/linda-exe
    XDG_SESSION_ID=10
    USER=vlasta
    EXCITINGRUNDIR=/home/vlasta/md/run
    CRY2K9_TEST=/home/vlasta/crystal/test_cases/inputs
    VERSION=v2_0_1
    HOTBIT_PARAMETERS=/usr/share/doc/hotbit/param/fixed_parameters
    KSSOLVPATH=/home/vlasta/md/kssolv
    NSLOTS=8
    GAUSS_ARCHDIR=/usr/local/gaus/g16/arch
    ABINIT_PP_PATH=/usr/local/share/abinit-pseudopotentials//GGA_PAW:/usr/local/share/abinit-pseudopoten-
tials//GGA_HGHK:/usr/local/share/abinit-pseudopotentials//GGA_FHI:/usr/local/share/abinit-pseudopoten-
tials//LDA_TM:/usr/local/share/abinit-pseudopotentials//LDA_PAW:/usr/local/share/abinit-pseudopoten-
tials//LDA_HGH:/usr/local/share/abinit-pseudopotentials//GGA_FHI:/usr/local/share/abinit-pseudopoten-
tials//LDA_FHI:/usr/share/doc/abinit-doc/tests/Psps_for_tests
    PWD=/home/vlasta/crystal/tmp/tmp2648
    EXCITINGROOT=/usr
    HOME=/home/vlasta
    CRY2K9_UTILS=/home/vlasta/crystal/utils09
    SSH_CLIENT=10.135.24.32 50621 22
    DFTB_COMMAND=dftb+
    TIMEFORMAT=   Elapsed time = %0lR
    GMXCMD=mpirun -np 4 mdrun_mpi
    PW_ROOT=/usr
    DFTB_PREFIX=/home/vlasta/md/potentials/slako/pbc-0-3/
    GAUSS_EXEDIR=/usr/local/gaus/g16/bsd:/usr/local/gaus/g16
    MALLOC_CHECK_=0
    SIESTA_PP_PATH=.
    CRY2K9_EXEDIR=/home/vlasta/crystal/bin/Linux-ifort
    EXCITINGASE=/home/vlasta/md/run/excitingscripts/ase
    ASE_ABINIT_COMMAND=mpirun -np 2 abinit < PREFIX.files > PREFIX.log
    LC_ADDRESS=hr_HR.UTF-8
    LC_NUMERIC=hr_HR.UTF-8
    DACAPOEXE_PARALLEL=/usr/bin/dacapo_mpi.run
    FLEUR_INPGEN=/home/vlasta/bin/inpgen.x
    SSH_TTY=/dev/pts/0
    MAIL=/var/mail/vlasta
    PAPERSIZE=a4
    ELK_SPECIES_PATH=/usr/local/share/elk-dft/species/
    SHELL=/bin/bash
    TERM=xterm
    CRY2K9_ROOT=/home/vlasta/crystal
    CRY2K9_ARCH=Linux-ifort
    CRY2K9_BIN=bin
    G16BASIS=/usr/local/gaus/g16/basis
    FLEUR=/home/vlasta/bin/fleur.x
    LAMMPS_COMMAND=/usr/bin/lammps
    SHLVL=2
    LANGUAGE=en_US:en
    PYTHONPATH=/usr/local/gaus/gauopen:/usr/local/gaus/g16/bsd:/usr/local/gaus/g16
    LC_TELEPHONE=hr_HR.UTF-8
    LOGNAME=vlasta
    DBUS_SESSION_BUS_ADDRESS=unix:path=/run/user/1003/bus
    XDG_RUNTIME_DIR=/run/user/1003
    EXCITINGSHARE=/usr/share/exciting
    HOTBIT_DIR=/usr/share/doc/hotbit/examples
    PATH=/home/vlasta/crystal/tmp/tmp2648::/usr/local/share/XCrySDen/:/usr/local/sbin:/usr/lo-
cal/bin:/usr/sbin:/usr/bin:/sbin:/bin:/usr/games:/usr/local/games:./:/home/vlasta/crystal/bin/Linux-
ifort:/home/vlasta/crystal/utils09:/home/vlasta/crystal/crgra2006:/home/vlasta/md/run/exciting-
scripts:/home/vlasta/md/run/excitingscripts/ATAT@exciting:/usr/local/gaus/gauopen:/usr/lo-
cal/gaus/g16/bsd:/usr/local/gaus/g16:/home/tmpacc/QuantumWise/atk/vnl/bin:/home/tmpacc/QuantumWise/atk/atkpy-
thon/bin:/home/tmpacc/QuantumWise/atk/license:/usr/local/share/XCrySDen//scripts:/usr/local/share/XCryS-
Den//util
    LC_IDENTIFICATION=hr_HR.UTF-8
    PERLLIB=/usr/local/gaus/gauopen:/usr/local/gaus/g16/bsd:/usr/local/gaus/g16
    EXCITINGSCRIPTS=/home/vlasta/md/run/excitingscripts
    PGI_TERM=trace,abort
    XCRYSDEN_SCRATCH=/tmp
    XLIB_SKIP_ARGB_VISUALS=1
    ATATROOT=/home/vlasta/md/run/excitingscripts/atat
    EXCITINGBIN=/usr/bin
```

```
    LESSOPEN=| /usr/bin/lesspipe %s
    OMP_NUM_THREADS=2
    LC_TIME=hr_HR.UTF-8
    _=/usr/bin/mpirun.mpich

  Hydra internal environment:
  ---------------------------
    GFORTRAN_UNBUFFERED_PRECONNECTED=y


    Proxy information:
    *********************
      [1] proxy: lmfz640 (8 cores)
      Exec list: /home/vlasta/crystal/tmp/tmp2648/Pcrystal (8 processes);


==================================================================================================

[mpiexec@lmfz640] Timeout set to -1 (-1 means infinite)
[mpiexec@lmfz640] Got a control port string of lmfz640:44891

Proxy launch args: /usr/bin/hydra_pmi_proxy --control-port lmfz640:44891 --debug --rmk user --launcher ssh --
demux poll --pgid 0 --retries 10 --usize -2 --proxy-id

Arguments being passed to proxy 0:
--version 3.3a2 --iface-ip-env-name MPIR_CVAR_CH3_INTERFACE_HOSTNAME --hostname lmfz640 --global-core-map
0,8,8 --pmi-id-map 0,0 --global-process-count 8 --auto-cleanup 1 --pmi-kvsname kvs_2689_0 --pmi-process-map-
ping (vector,(0,1,8)) --ckpoint-num -1 --global-inherited-env 93 'EXCITINGVISUAL=/usr/share/exciting/xml/vis-
ualizationtemplates' 'LS_COL-
ORS=rs=0:di=01;34:ln=01;36:mh=00:pi=40;33:so=01;35:do=01;35:bd=40;33;01:cd=40;33;01:or=40;31;01:mi=00:su=37;4
1:sg=30;43:ca=30;41:tw=30;42:ow=34;42:st=37;44:ex=01;32:*.tar=01;31:*.tgz=01;31:*.arc=01;31:*.arj=01;31:*.taz
=01;31:*.lha=01;31:*.lz4=01;31:*.lzh=01;31:*.lzma=01;31:*.tlz=01;31:*.txz=01;31:*.tzo=01;31:*.t7z=01;31:*.zip
=01;31:*.z=01;31:*.Z=01;31:*.dz=01;31:*.gz=01;31:*.lrz=01;31:*.lz=01;31:*.lzo=01;31:*.xz=01;31:*.zst=01;31:*.
tzst=01;31:*.bz2=01;31:*.bz=01;31:*.tbz=01;31:*.tbz2=01;31:*.tz=01;31:*.deb=01;31:*.rpm=01;31:*.jar=01;31:*.w
ar=01;31:*.ear=01;31:*.sar=01;31:*.rar=01;31:*.alz=01;31:*.ace=01;31:*.zoo=01;31:*.cpio=01;31:*.7z=01;31:*.rz
=01;31:*.cab=01;31:*.wim=01;31:*.swm=01;31:*.dwm=01;31:*.esd=01;31:*.jpg=01;35:*.jpeg=01;35:*.mjpg=01;35:*.mj
peg=01;35:*.gif=01;35:*.bmp=01;35:*.pbm=01;35:*.pgm=01;35:*.ppm=01;35:*.tga=01;35:*.xbm=01;35:*.xpm=01;35:*.t
if=01;35:*.tiff=01;35:*.png=01;35:*.svg=01;35:*.svgz=01;35:*.mng=01;35:*.pcx=01;35:*.mov=01;35:*.mpg=01;35:*.
mpeg=01;35:*.m2v=01;35:*.mkv=01;35:*.webm=01;35:*.ogm=01;35:*.mp4=01;35:*.m4v=01;35:*.mp4v=01;35:*.vob=01;35:
*.qt=01;35:*.nuv=01;35:*.wmv=01;35:*.asf=01;35:*.rm=01;35:*.rmvb=01;35:*.flc=01;35:*.avi=01;35:*.fli=01;35:*.
flv=01;35:*.gl=01;35:*.dl=01;35:*.xcf=01;35:*.xwd=01;35:*.yuv=01;35:*.cgm=01;35:*.emf=01;35:*.ogv=01;35:*.ogx
=01;35:*.aac=00;36:*.au=00;36:*.flac=00;36:*.m4a=00;36:*.mid=00;36:*.midi=00;36:*.mka=00;36:*.mp3=00;36:*.mpc
=00;36:*.ogg=00;36:*.ra=00;36:*.wav=00;36:*.oga=00;36:*.opus=00;36:*.spx=00;36:*.xspf=00;36:' 'LD_LI-
BRARY_PATH=/usr/local/gaus/g16/bsd:/usr/local/gaus/g16:/usr/local/gaus/gv/lib'
'GPAW_SETUP_PATH=/usr/share/gpaw-setups' 'GAUSS_BSDDIR=/usr/local/gaus/g16/bsd' 'GRA6_EX-
EDIR=/home/vlasta/crystal/crgra2006/bin/Linux-pgf' 'CMR_REPOSITORY=/home/vlasta/cmr' 'LC_MEASURE-
MENT=hr_HR.UTF-8' 'SSH_CONNECTION=10.135.24.32 50621 10.135.24.38 22' 'LESSCLOSE=/usr/bin/lesspipe %s %s'
'LC_PAPER=hr_HR.UTF-8' 'LC_MONETARY=hr_HR.UTF-8' 'LANG=en_US.UTF-8' 'g16root=/usr/local/gaus'
'PSEUDO_DIR=/home/vlasta/md/potentials/UPF' 'CRY2K6_GRA=/home/vlasta/crystal/crgra2006' '_DSM_BARRIER=SHM'
'DACAPOPATH=/usr/share/dacapo-psp' 'OPENBLAS_NUM_THREADS=1' 'OLDPWD=/home/vlasta/crystal/tmp/c3s' 'XCRYS-
DEN_TOPDIR=/usr/local/share/XCrySDen/' 'TMP_DIR=/home/vlasta/crystal/tmp/tmp2648' 'EXCIT-
INGATAT=/home/vlasta/md/run/excitingscripts/ATAT@exciting' 'GV_DIR=/usr/local/gaus/gv' 'DACAPOEXE_SE-
RIAL=/usr/bin/dacapo_serial.run' 'CRY2K9_SCRDIR=/home/vlasta/crystal/tmp' 'SI-
ESTA_SCRIPT=/home/vlasta/bin/run_siesta.py' 'LC_NAME=hr_HR.UTF-8' 'GAUSS_LEXEDIR=/usr/local/gaus/g16/linda-
exe' 'XDG_SESSION_ID=10' 'USER=vlasta' 'EXCITINGRUNDIR=/home/vlasta/md/run' 'CRY2K9_TEST=/home/vlasta/crys-
tal/test_cases/inputs' 'VERSION=v2_0_1' 'HOTBIT_PARAMETERS=/usr/share/doc/hotbit/param/fixed_parameters'
'KSSOLVPATH=/home/vlasta/md/kssolv' 'NSLOTS=8' 'GAUSS_ARCHDIR=/usr/local/gaus/g16/arch'
'ABINIT_PP_PATH=/usr/local/share/abinit-pseudopotentials//GGA_PAW:/usr/local/share/abinit-pseudopoten-
tials//GGA_HGHK:/usr/local/share/abinit-pseudopotentials//GGA_FHI:/usr/local/share/abinit-pseudopoten-
tials//LDA_TM:/usr/local/share/abinit-pseudopotentials//LDA_PAW:/usr/local/share/abinit-pseudopoten-
tials//LDA_HGH:/usr/local/share/abinit-pseudopotentials//GGA_FHI:/usr/local/share/abinit-pseudopoten-
tials//LDA_FHI:/usr/share/doc/abinit-doc/tests/Psps_for_tests' 'PWD=/home/vlasta/crystal/tmp/tmp2648' 'EXCIT-
INGROOT=/usr' 'HOME=/home/vlasta' 'CRY2K9_UTILS=/home/vlasta/crystal/utils09' 'SSH_CLIENT=10.135.24.32 50621
22' 'DFTB_COMMAND=dftb+' 'TIMEFORMAT=   Elapsed time = %0lR' 'GMXCMD=mpirun -np 4 mdrun_mpi' 'PW_ROOT=/usr'
'DFTB_PREFIX=/home/vlasta/md/potentials/slako/pbc-0-3/' 'GAUSS_EXEDIR=/usr/local/gaus/g16/bsd:/usr/lo-
cal/gaus/g16' 'MALLOC_CHECK_=0' 'SIESTA_PP_PATH=.' 'CRY2K9_EXEDIR=/home/vlasta/crystal/bin/Linux-ifort' 'EX-
CITINGASE=/home/vlasta/md/run/excitingscripts/ase' 'ASE_ABINIT_COMMAND=mpirun -np 2 abinit < PREFIX.files >
PREFIX.log' 'LC_ADDRESS=hr_HR.UTF-8' 'LC_NUMERIC=hr_HR.UTF-8' 'DACAPOEXE_PARALLEL=/usr/bin/dacapo_mpi.run'
'FLEUR_INPGEN=/home/vlasta/bin/inpgen.x' 'SSH_TTY=/dev/pts/0' 'MAIL=/var/mail/vlasta' 'PAPERSIZE=a4'
'ELK_SPECIES_PATH=/usr/local/share/elk-dft/species/' 'SHELL=/bin/bash' 'TERM=xterm'
'CRY2K9_ROOT=/home/vlasta/crystal' 'CRY2K9_ARCH=Linux-ifort' 'CRY2K9_BIN=bin' 'G16BASIS=/usr/lo-
cal/gaus/g16/basis' 'FLEUR=/home/vlasta/bin/fleur.x' 'LAMMPS_COMMAND=/usr/bin/lammps' 'SHLVL=2' 'LAN-
GUAGE=en_US:en' 'PYTHONPATH=/usr/local/gaus/gauopen:/usr/local/gaus/g16/bsd:/usr/local/gaus/g16' 'LC_TELE-
PHONE=hr_HR.UTF-8' 'LOGNAME=vlasta' 'DBUS_SESSION_BUS_ADDRESS=unix:path=/run/user/1003/bus'
'XDG_RUNTIME_DIR=/run/user/1003' 'EXCITINGSHARE=/usr/share/exciting' 'HOTBIT_DIR=/usr/share/doc/hotbit/exam-
ples' 'PATH=/home/vlasta/crystal/tmp/tmp2648::/usr/local/share/XCrySDen/:/usr/local/sbin:/usr/lo-
cal/bin:/usr/sbin:/usr/bin:/sbin:/bin:/usr/games:/usr/local/games:./:/home/vlasta/crystal/bin/Linux-
ifort:/home/vlasta/crystal/utils09:/home/vlasta/crystal/crgra2006:/home/vlasta/md/run/exciting-
```

```
scripts:/home/vlasta/md/run/excitingscripts/ATAT@exciting:/usr/local/gaus/gauopen:/usr/lo-
cal/gaus/g16/bsd:/usr/local/gaus/g16:/home/tmpacc/QuantumWise/atk/vnl/bin:/home/tmpacc/QuantumWise/atk/atkpy-
thon/bin:/home/tmpacc/QuantumWise/atk/license:/usr/local/share/XCrySDen//scripts:/usr/local/share/XCryS-
Den//util' 'LC_IDENTIFICATION=hr_HR.UTF-8' 'PERLLIB=/usr/local/gaus/gauopen:/usr/local/gaus/g16/bsd:/usr/lo-
cal/gaus/g16' 'EXCITINGSCRIPTS=/home/vlasta/md/run/excitingscripts' 'PGI_TERM=trace,abort' 'XCRYS-
DEN_SCRATCH=/tmp' 'XLIB_SKIP_ARGB_VISUALS=1' 'ATATROOT=/home/vlasta/md/run/excitingscripts/atat' 'EXCIT-
INGBIN=/usr/bin' 'LESSOPEN=| /usr/bin/lesspipe %s' 'OMP_NUM_THREADS=2' 'LC_TIME=hr_HR.UTF-8' '_=/usr/bin/mpi-
run.mpich' --global-user-env 0 --global-system-env 1 'GFORTRAN_UNBUFFERED_PRECONNECTED=y' --proxy-core-count
8 --exec --exec-appnum 0 --exec-proc-count 8 --exec-local-env 0 --exec-wdir /home/vlasta/crystal/tmp/tmp2648
--exec-args 1 /home/vlasta/crystal/tmp/tmp2648/Pcrystal

[mpiexec@lmfz640] Launch arguments: /usr/bin/hydra_pmi_proxy --control-port lmfz640:44891 --debug --rmk user
--launcher ssh --demux poll --pgid 0 --retries 10 --usize -2 --proxy-id 0
[proxy:0:0@lmfz640] got pmi command (from 0): init
pmi_version=1 pmi_subversion=1
[proxy:0:0@lmfz640] PMI response: cmd=response_to_init pmi_version=1 pmi_subversion=1 rc=0
[proxy:0:0@lmfz640] got pmi command (from 0): get_maxes

[proxy:0:0@lmfz640] PMI response: cmd=maxes kvsname_max=256 keylen_max=64 vallen_max=1024
[proxy:0:0@lmfz640] got pmi command (from 6): init
pmi_version=1 pmi_subversion=1
[proxy:0:0@lmfz640] PMI response: cmd=response_to_init pmi_version=1 pmi_subversion=1 rc=0
[proxy:0:0@lmfz640] got pmi command (from 8): init
pmi_version=1 pmi_subversion=1
[proxy:0:0@lmfz640] PMI response: cmd=response_to_init pmi_version=1 pmi_subversion=1 rc=0
[proxy:0:0@lmfz640] got pmi command (from 13): init
pmi_version=1 pmi_subversion=1
[proxy:0:0@lmfz640] PMI response: cmd=response_to_init pmi_version=1 pmi_subversion=1 rc=0
[proxy:0:0@lmfz640] got pmi command (from 17): init
pmi_version=1 pmi_subversion=1
[proxy:0:0@lmfz640] PMI response: cmd=response_to_init pmi_version=1 pmi_subversion=1 rc=0
[proxy:0:0@lmfz640] got pmi command (from 21): init
pmi_version=1 pmi_subversion=1
[proxy:0:0@lmfz640] PMI response: cmd=response_to_init pmi_version=1 pmi_subversion=1 rc=0
[proxy:0:0@lmfz640] got pmi command (from 0): get_appnum

[proxy:0:0@lmfz640] PMI response: cmd=appnum appnum=0
[proxy:0:0@lmfz640] got pmi command (from 6): get_maxes

[proxy:0:0@lmfz640] PMI response: cmd=maxes kvsname_max=256 keylen_max=64 vallen_max=1024
[proxy:0:0@lmfz640] got pmi command (from 8): get_maxes

[proxy:0:0@lmfz640] PMI response: cmd=maxes kvsname_max=256 keylen_max=64 vallen_max=1024
[proxy:0:0@lmfz640] got pmi command (from 13): get_maxes

[proxy:0:0@lmfz640] PMI response: cmd=maxes kvsname_max=256 keylen_max=64 vallen_max=1024
[proxy:0:0@lmfz640] got pmi command (from 17): get_maxes

[proxy:0:0@lmfz640] PMI response: cmd=maxes kvsname_max=256 keylen_max=64 vallen_max=1024
[proxy:0:0@lmfz640] got pmi command (from 25): init
pmi_version=1 pmi_subversion=1
[proxy:0:0@lmfz640] PMI response: cmd=response_to_init pmi_version=1 pmi_subversion=1 rc=0
[proxy:0:0@lmfz640] got pmi command (from 29): init
pmi_version=1 pmi_subversion=1
[proxy:0:0@lmfz640] PMI response: cmd=response_to_init pmi_version=1 pmi_subversion=1 rc=0
[proxy:0:0@lmfz640] got pmi command (from 0): get_my_kvsname

[proxy:0:0@lmfz640] PMI response: cmd=my_kvsname kvsname=kvs_2689_0
[proxy:0:0@lmfz640] got pmi command (from 6): get_appnum

[proxy:0:0@lmfz640] PMI response: cmd=appnum appnum=0
[proxy:0:0@lmfz640] got pmi command (from 8): get_appnum

[proxy:0:0@lmfz640] PMI response: cmd=appnum appnum=0
[proxy:0:0@lmfz640] got pmi command (from 13): get_appnum

[proxy:0:0@lmfz640] PMI response: cmd=appnum appnum=0
[proxy:0:0@lmfz640] got pmi command (from 17): get_appnum

[proxy:0:0@lmfz640] PMI response: cmd=appnum appnum=0
[proxy:0:0@lmfz640] got pmi command (from 21): get_maxes

[proxy:0:0@lmfz640] PMI response: cmd=maxes kvsname_max=256 keylen_max=64 vallen_max=1024
[proxy:0:0@lmfz640] got pmi command (from 25): get_maxes

[proxy:0:0@lmfz640] PMI response: cmd=maxes kvsname_max=256 keylen_max=64 vallen_max=1024
[proxy:0:0@lmfz640] got pmi command (from 0): get_my_kvsname

[proxy:0:0@lmfz640] PMI response: cmd=my_kvsname kvsname=kvs_2689_0
```

```
[proxy:0:0@lmfz640] got pmi command (from 6): get_my_kvsname

[proxy:0:0@lmfz640] PMI response: cmd=my_kvsname kvsname=kvs_2689_0
[proxy:0:0@lmfz640] got pmi command (from 8): get_my_kvsname

[proxy:0:0@lmfz640] PMI response: cmd=my_kvsname kvsname=kvs_2689_0
[proxy:0:0@lmfz640] got pmi command (from 13): get_my_kvsname

[proxy:0:0@lmfz640] PMI response: cmd=my_kvsname kvsname=kvs_2689_0
[proxy:0:0@lmfz640] got pmi command (from 17): get_my_kvsname

[proxy:0:0@lmfz640] PMI response: cmd=my_kvsname kvsname=kvs_2689_0
[proxy:0:0@lmfz640] got pmi command (from 21): get_appnum

[proxy:0:0@lmfz640] PMI response: cmd=appnum appnum=0
[proxy:0:0@lmfz640] got pmi command (from 29): get_maxes

[proxy:0:0@lmfz640] PMI response: cmd=maxes kvsname_max=256 keylen_max=64 vallen_max=1024
[proxy:0:0@lmfz640] got pmi command (from 0): get
kvsname=kvs_2689_0 key=PMI_process_mapping
[proxy:0:0@lmfz640] PMI response: cmd=get_result rc=0 msg=success value=(vector,(0,1,8))
[proxy:0:0@lmfz640] got pmi command (from 6): get_my_kvsname

[proxy:0:0@lmfz640] PMI response: cmd=my_kvsname kvsname=kvs_2689_0
[proxy:0:0@lmfz640] got pmi command (from 8): get_my_kvsname

[proxy:0:0@lmfz640] PMI response: cmd=my_kvsname kvsname=kvs_2689_0
[proxy:0:0@lmfz640] got pmi command (from 13): get_my_kvsname

[proxy:0:0@lmfz640] PMI response: cmd=my_kvsname kvsname=kvs_2689_0
[proxy:0:0@lmfz640] got pmi command (from 17): get_my_kvsname

[proxy:0:0@lmfz640] PMI response: cmd=my_kvsname kvsname=kvs_2689_0
[proxy:0:0@lmfz640] got pmi command (from 21): get_my_kvsname

[proxy:0:0@lmfz640] PMI response: cmd=my_kvsname kvsname=kvs_2689_0
[proxy:0:0@lmfz640] got pmi command (from 25): get_appnum

[proxy:0:0@lmfz640] PMI response: cmd=appnum appnum=0
[proxy:0:0@lmfz640] got pmi command (from 0): put
kvsname=kvs_2689_0 key=sharedFilename[0] value=/dev/shm/mpich_shar_tmpMLEk6n
[proxy:0:0@lmfz640] cached command: sharedFilename[0]=/dev/shm/mpich_shar_tmpMLEk6n
[proxy:0:0@lmfz640] PMI response: cmd=put_result rc=0 msg=success
[proxy:0:0@lmfz640] got pmi command (from 6): get
kvsname=kvs_2689_0 key=PMI_process_mapping
[proxy:0:0@lmfz640] PMI response: cmd=get_result rc=0 msg=success value=(vector,(0,1,8))
[proxy:0:0@lmfz640] got pmi command (from 8): get
kvsname=kvs_2689_0 key=PMI_process_mapping
[proxy:0:0@lmfz640] PMI response: cmd=get_result rc=0 msg=success value=(vector,(0,1,8))
[proxy:0:0@lmfz640] got pmi command (from 13): get
kvsname=kvs_2689_0 key=PMI_process_mapping
[proxy:0:0@lmfz640] PMI response: cmd=get_result rc=0 msg=success value=(vector,(0,1,8))
[proxy:0:0@lmfz640] got pmi command (from 17): get
kvsname=kvs_2689_0 key=PMI_process_mapping
[proxy:0:0@lmfz640] PMI response: cmd=get_result rc=0 msg=success value=(vector,(0,1,8))
[proxy:0:0@lmfz640] got pmi command (from 21): get_my_kvsname

[proxy:0:0@lmfz640] PMI response: cmd=my_kvsname kvsname=kvs_2689_0
[proxy:0:0@lmfz640] got pmi command (from 29): get_appnum

[proxy:0:0@lmfz640] PMI response: cmd=appnum appnum=0
[proxy:0:0@lmfz640] got pmi command (from 0): barrier_in

[proxy:0:0@lmfz640] got pmi command (from 6): barrier_in

[proxy:0:0@lmfz640] got pmi command (from 8): barrier_in

[proxy:0:0@lmfz640] got pmi command (from 13): barrier_in

[proxy:0:0@lmfz640] got pmi command (from 17): barrier_in

[proxy:0:0@lmfz640] got pmi command (from 21): get
kvsname=kvs_2689_0 key=PMI_process_mapping
[proxy:0:0@lmfz640] PMI response: cmd=get_result rc=0 msg=success value=(vector,(0,1,8))
[proxy:0:0@lmfz640] got pmi command (from 25): get_my_kvsname

[proxy:0:0@lmfz640] PMI response: cmd=my_kvsname kvsname=kvs_2689_0
[proxy:0:0@lmfz640] got pmi command (from 21): barrier_in
```

```
[proxy:0:0@lmfz640] got pmi command (from 29): get_my_kvsname

[proxy:0:0@lmfz640] PMI response: cmd=my_kvsname kvsname=kvs_2689_0
[proxy:0:0@lmfz640] got pmi command (from 25): get_my_kvsname

[proxy:0:0@lmfz640] PMI response: cmd=my_kvsname kvsname=kvs_2689_0
[proxy:0:0@lmfz640] got pmi command (from 29): get_my_kvsname

[proxy:0:0@lmfz640] PMI response: cmd=my_kvsname kvsname=kvs_2689_0
[proxy:0:0@lmfz640] got pmi command (from 25): get
kvsname=kvs_2689_0 key=PMI_process_mapping
[proxy:0:0@lmfz640] PMI response: cmd=get_result rc=0 msg=success value=(vector,(0,1,8))
[proxy:0:0@lmfz640] got pmi command (from 29): get
kvsname=kvs_2689_0 key=PMI_process_mapping
[proxy:0:0@lmfz640] PMI response: cmd=get_result rc=0 msg=success value=(vector,(0,1,8))
[proxy:0:0@lmfz640] got pmi command (from 25): barrier_in

[proxy:0:0@lmfz640] got pmi command (from 29): barrier_in

[proxy:0:0@lmfz640] flushing 1 put command(s) out
[proxy:0:0@lmfz640] forwarding command (cmd=put sharedFilename[0]=/dev/shm/mpich_shar_tmpMLEk6n) upstream
[mpiexec@lmfz640] [pgid: 0] got PMI command: cmd=put sharedFilename[0]=/dev/shm/mpich_shar_tmpMLEk6n
[proxy:0:0@lmfz640] forwarding command (cmd=barrier_in) upstream
[mpiexec@lmfz640] [pgid: 0] got PMI command: cmd=barrier_in
[mpiexec@lmfz640] PMI response to fd 6 pid 29: cmd=keyval_cache sharedFilename[0]=/dev/shm/mpich_shar_tmpM-
LEk6n
[mpiexec@lmfz640] PMI response to fd 6 pid 29: cmd=barrier_out
[proxy:0:0@lmfz640] PMI response: cmd=barrier_out
[proxy:0:0@lmfz640] PMI response: cmd=barrier_out
[proxy:0:0@lmfz640] PMI response: cmd=barrier_out
[proxy:0:0@lmfz640] PMI response: cmd=barrier_out
[proxy:0:0@lmfz640] PMI response: cmd=barrier_out
[proxy:0:0@lmfz640] PMI response: cmd=barrier_out
[proxy:0:0@lmfz640] PMI response: cmd=barrier_out
[proxy:0:0@lmfz640] PMI response: cmd=barrier_out
[proxy:0:0@lmfz640] got pmi command (from 17): get
kvsname=kvs_2689_0 key=sharedFilename[0]
[proxy:0:0@lmfz640] PMI response: cmd=get_result rc=0 msg=success value=/dev/shm/mpich_shar_tmpMLEk6n
[proxy:0:0@lmfz640] got pmi command (from 25): get
kvsname=kvs_2689_0 key=sharedFilename[0]
[proxy:0:0@lmfz640] PMI response: cmd=get_result rc=0 msg=success value=/dev/shm/mpich_shar_tmpMLEk6n
[proxy:0:0@lmfz640] got pmi command (from 6): get
kvsname=kvs_2689_0 key=sharedFilename[0]
[proxy:0:0@lmfz640] PMI response: cmd=get_result rc=0 msg=success value=/dev/shm/mpich_shar_tmpMLEk6n
[proxy:0:0@lmfz640] got pmi command (from 8): get
kvsname=kvs_2689_0 key=sharedFilename[0]
[proxy:0:0@lmfz640] PMI response: cmd=get_result rc=0 msg=success value=/dev/shm/mpich_shar_tmpMLEk6n
[proxy:0:0@lmfz640] got pmi command (from 13): get
kvsname=kvs_2689_0 key=sharedFilename[0]
[proxy:0:0@lmfz640] PMI response: cmd=get_result rc=0 msg=success value=/dev/shm/mpich_shar_tmpMLEk6n
[proxy:0:0@lmfz640] got pmi command (from 21): get
kvsname=kvs_2689_0 key=sharedFilename[0]
[proxy:0:0@lmfz640] PMI response: cmd=get_result rc=0 msg=success value=/dev/shm/mpich_shar_tmpMLEk6n
[proxy:0:0@lmfz640] got pmi command (from 29): get
kvsname=kvs_2689_0 key=sharedFilename[0]
[proxy:0:0@lmfz640] PMI response: cmd=get_result rc=0 msg=success value=/dev/shm/mpich_shar_tmpMLEk6n
[proxy:0:0@lmfz640] got pmi command (from 0): put
kvsname=kvs_2689_0 key=P0-businesscard value=description#lmfz640$port#39501$ifname#10.135.24.38$
[proxy:0:0@lmfz640] cached command: P0-businesscard=description#lmfz640$port#39501$ifname#10.135.24.38$
[proxy:0:0@lmfz640] PMI response: cmd=put_result rc=0 msg=success
[proxy:0:0@lmfz640] got pmi command (from 6): put
kvsname=kvs_2689_0 key=P1-businesscard value=description#lmfz640$port#52411$ifname#10.135.24.38$
[proxy:0:0@lmfz640] cached command: P1-businesscard=description#lmfz640$port#52411$ifname#10.135.24.38$
[proxy:0:0@lmfz640] PMI response: cmd=put_result rc=0 msg=success
[proxy:0:0@lmfz640] got pmi command (from 8): put
kvsname=kvs_2689_0 key=P2-businesscard value=description#lmfz640$port#40759$ifname#10.135.24.38$
[proxy:0:0@lmfz640] cached command: P2-businesscard=description#lmfz640$port#40759$ifname#10.135.24.38$
[proxy:0:0@lmfz640] PMI response: cmd=put_result rc=0 msg=success
[proxy:0:0@lmfz640] got pmi command (from 13): put
kvsname=kvs_2689_0 key=P3-businesscard value=description#lmfz640$port#38407$ifname#10.135.24.38$
[proxy:0:0@lmfz640] cached command: P3-businesscard=description#lmfz640$port#38407$ifname#10.135.24.38$
[proxy:0:0@lmfz640] PMI response: cmd=put_result rc=0 msg=success
[proxy:0:0@lmfz640] got pmi command (from 17): put
kvsname=kvs_2689_0 key=P4-businesscard value=description#lmfz640$port#39411$ifname#10.135.24.38$
[proxy:0:0@lmfz640] cached command: P4-businesscard=description#lmfz640$port#39411$ifname#10.135.24.38$
[proxy:0:0@lmfz640] PMI response: cmd=put_result rc=0 msg=success
[proxy:0:0@lmfz640] got pmi command (from 21): put
kvsname=kvs_2689_0 key=P5-businesscard value=description#lmfz640$port#52769$ifname#10.135.24.38$
[proxy:0:0@lmfz640] cached command: P5-businesscard=description#lmfz640$port#52769$ifname#10.135.24.38$
```

```
[proxy:0:0@lmfz640] PMI response: cmd=put_result rc=0 msg=success
[proxy:0:0@lmfz640] got pmi command (from 25): put
kvsname=kvs_2689_0 key=P6-businesscard value=description#lmfz640$port#46695$ifname#10.135.24.38$
[proxy:0:0@lmfz640] cached command: P6-businesscard=description#lmfz640$port#46695$ifname#10.135.24.38$
[proxy:0:0@lmfz640] PMI response: cmd=put_result rc=0 msg=success
[proxy:0:0@lmfz640] got pmi command (from 29): put
kvsname=kvs_2689_0 key=P7-businesscard value=description#lmfz640$port#54707$ifname#10.135.24.38$
[proxy:0:0@lmfz640] cached command: P7-businesscard=description#lmfz640$port#54707$ifname#10.135.24.38$
[proxy:0:0@lmfz640] PMI response: cmd=put_result rc=0 msg=success
[proxy:0:0@lmfz640] got pmi command (from 0): barrier_in

[proxy:0:0@lmfz640] got pmi command (from 6): barrier_in

[proxy:0:0@lmfz640] got pmi command (from 8): barrier_in

[proxy:0:0@lmfz640] got pmi command (from 13): barrier_in

[proxy:0:0@lmfz640] got pmi command (from 17): barrier_in

[proxy:0:0@lmfz640] got pmi command (from 21): barrier_in

[proxy:0:0@lmfz640] got pmi command (from 25): barrier_in

[proxy:0:0@lmfz640] got pmi command (from 29): barrier_in

[proxy:0:0@lmfz640] flushing 8 put command(s) out
[proxy:0:0@lmfz640] forwarding command (cmd=put P0-businesscard=description#lmfz640$port#39501$if-
name#10.135.24.38$ P1-businesscard=description#lmfz640$port#52411$ifname#10.135.24.38$ P2-businesscard=de-
scription#lmfz640$port#40759$ifname#10.135.24.38$ P3-businesscard=description#lmfz640$port#38407$if-
name#10.135.24.38$ P4-businesscard=description#lmfz640$port#39411$ifname#10.135.24.38$ P5-businesscard=de-
scription#lmfz640$port#52769$ifname#10.135.24.38$ P6-businesscard=description#lmfz640$port#46695$if-
name#10.135.24.38$ P7-businesscard=description#lmfz640$port#54707$ifname#10.135.24.38$) upstream
[mpiexec@lmfz640] [pgid: 0] got PMI command: cmd=put P0-businesscard=description#lmfz640$port#39501$if-
name#10.135.24.38$ P1-businesscard=description#lmfz640$port#52411$ifname#10.135.24.38$ P2-businesscard=de-
scription#lmfz640$port#40759$ifname#10.135.24.38$ P3-businesscard=description#lmfz640$port#38407$if-
name#10.135.24.38$ P4-businesscard=description#lmfz640$port#39411$ifname#10.135.24.38$ P5-businesscard=de-
scription#lmfz640$port#52769$ifname#10.135.24.38$ P6-businesscard=description#lmfz640$port#46695$if-
name#10.135.24.38$ P7-businesscard=description#lmfz640$port#54707$ifname#10.135.24.38$
[proxy:0:0@lmfz640] forwarding command (cmd=barrier_in) upstream
[mpiexec@lmfz640] [pgid: 0] got PMI command: cmd=barrier_in
[mpiexec@lmfz640] PMI response to fd 6 pid 29: cmd=keyval_cache P0-businesscard=descrip-
tion#lmfz640$port#39501$ifname#10.135.24.38$ P1-businesscard=description#lmfz640$port#52411$if-
name#10.135.24.38$ P2-businesscard=description#lmfz640$port#40759$ifname#10.135.24.38$ P3-businesscard=de-
scription#lmfz640$port#38407$ifname#10.135.24.38$ P4-businesscard=description#lmfz640$port#39411$if-
name#10.135.24.38$ P5-businesscard=description#lmfz640$port#52769$ifname#10.135.24.38$ P6-businesscard=de-
scription#lmfz640$port#46695$ifname#10.135.24.38$ P7-businesscard=description#lmfz640$port#54707$if-
name#10.135.24.38$
[mpiexec@lmfz640] PMI response to fd 6 pid 29: cmd=barrier_out
[proxy:0:0@lmfz640] PMI response: cmd=barrier_out
[proxy:0:0@lmfz640] PMI response: cmd=barrier_out
[proxy:0:0@lmfz640] PMI response: cmd=barrier_out
[proxy:0:0@lmfz640] PMI response: cmd=barrier_out
[proxy:0:0@lmfz640] PMI response: cmd=barrier_out
[proxy:0:0@lmfz640] PMI response: cmd=barrier_out
[proxy:0:0@lmfz640] PMI response: cmd=barrier_out
[proxy:0:0@lmfz640] PMI response: cmd=barrier_out
 PROCESS            3  OF           8  WORKING
 PROCESS            6  OF           8  WORKING
 PROCESS            0  OF           8  WORKING
 PROCESS            4  OF           8  WORKING
 PROCESS            2  OF           8  WORKING
 PROCESS            5  OF           8  WORKING
 PROCESS            7  OF           8  WORKING
 PROCESS            1  OF           8  WORKING


 *******************************************************************************
 *                                                                             *
 *                                CRYSTAL09                                    *
 *                              public : 2.0                                   *
 *                      December 10th, 2012 - parallel executable              *
 *                                                                             *
 *                                                                             *
 *                                                                             *
 *                              MAIN AUTHORS                                   *
 *                                                                             *
 *    R. DOVESI(1,10), V.R. SAUNDERS(2), C. ROETTI(1,10), R. ORLANDO (1,3),    *
 *  C.M. ZICOVICH-WILSON(1,4), F. PASCALE(5), B. CIVALLERI(1,10), K. DOLL(6),  *
 *       N.M. HARRISON(2,7), I. J. BUSH(2), Ph. D'ARCO(8), M. LLUNELL(9)       *
```

```
*                                                                              *
* (1) THEORETICAL CHEMISTRY GROUP - UNIVERSITA' DI TORINO - TORINO (ITALY)    *
*      http://www.crystal.unito.it                                             *
* (2) COMPUTATIONAL SCIENCE & ENGINEERING DEPARTMENT - STFC DARESBURY LABORATO*Y (UK)
*     http://www.cse.stfc.ac.uk/cmg/CRYSTAL/                                   *
* (3) UNIVERSITA' DEL PIEMONTE ORIENTALE - ALESSANDRIA (ITALY)                 *
* (4) UNIVERSIDAD AUTONOMA DEL ESTADO DE MORELOS - CUERNAVACA (MEXICO)         *
* (5) UNIVERSITE' HENRI POINCARE' - NANCY (FRANCE)                             *
* (6) MPI FUER FESTKOERPERFORSCHUNG - STUTTGART (GERMANY)                      *
* (7) IMPERIAL COLLEGE - LONDON (UK)                                           *
* (8) UNIVERSITE' PIERRE ET MARIE CURIE - PARIS (FRANCE)                       *
* (9) UNIVERSIDAD DE BARCELONA - BARCELONA (SPAIN)                             *
*(10) NIS - NANOSTRUCTURED INTERFACES AND SURFACES - TORINO (ITALY)            *
*     http://www.crystal.unito.it                                              *
********************************************************************************
EEEEEEEEEE STARTING  DATE 11 10 2019 TIME 14:14:25.8
Ca3SiO5 Cm  81100 ICSD W G Mumme LDA i VWN PP

CRYSTAL CALCULATION
(INPUT ACCORDING TO THE INTERNATIONAL TABLES FOR X-RAY CRYSTALLOGRAPHY)
CRYSTAL FAMILY                       :  MONOCLINIC
CRYSTAL CLASS  (GROTH - 1921)        :  MONOCLINIC DOMATIC

SPACE GROUP (NONCENTROSYMMETRIC)     :  C M

LATTICE PARAMETERS  (ANGSTROMS AND DEGREES) - CONVENTIONAL CELL
       A           B           C        ALPHA        BETA       GAMMA
   12.23500     7.07300     9.29800    90.00000   116.31000    90.00000


NUMBER OF IRREDUCIBLE ATOMS IN THE CONVENTIONAL CELL:   27

INPUT COORDINATES

ATOM AT. N.              COORDINATES
  1  20     2.785434612087E-01  1.246419190595E-17  3.006880006403E-01
  2  20     1.531537006405E-02  2.585068888852E-01  3.338407517012E-01
  3  20     1.531537006405E-02 -2.585068888852E-01  3.338407517012E-01
  4  20    -2.413293694602E-01 -2.359161832691E-01 -2.038417046735E-02
  5  20    -2.413293694602E-01  2.359161832691E-01 -2.038417046735E-02
  6  20    -3.032353025404E-01 -1.112570498310E-17 -3.537485816856E-01
  7  20     2.760504695417E-02  5.000000000000E-01  2.002234093501E-02
  8  20    -1.864937213094E-02 -2.762003324293E-01 -3.065722468464E-01
  9  20    -1.864937213094E-02  2.762003324293E-01 -3.065722468464E-01
 10  14    -2.804944480822E-01 -5.000000000000E-01 -3.483602425146E-01
 11  14     2.866911555732E-01  5.000000000000E-01  3.584634624474E-01
 12  14     1.563117691256E-03  7.236497377012E-19  1.317167878642E-03
 13   8     1.799435653619E-01 -4.321182612850E-18  4.596840795229E-01
 14   8     3.146382415874E-01  3.135397453095E-01  2.746504196733E-01
 15   8     3.146382415874E-01 -3.135397453095E-01  2.746504196733E-01
 16   8     1.416369842747E-01  5.000000000000E-01  3.242115389560E-01
 17   8    -1.202960000615E-01  5.000000000000E-01  1.158423931343E-01
 18   8    -1.323967604115E-01  3.626286874279E-18 -4.442461060711E-01
 19   8    -1.316127431580E-01 -5.000000000000E-01 -2.417309932451E-01
 20   8    -3.379350013612E-01 -3.099994556439E-01 -3.067690551272E-01
 21   8    -3.379350013612E-01  3.099994556439E-01 -3.067690551272E-01
 22   8     4.801992063025E-01 -3.310750185887E-17  4.956578249650E-01
 23   8     1.247353671791E-01  5.000000000000E-01 -1.435912966766E-01
 24   8     2.507864841237E-03  1.841554408899E-19  1.768212209225E-01
 25   8    -1.429906011707E-01  2.018186860997E-18 -1.219030917026E-01
 26   8     7.001017431963E-02  1.873620964908E-01 -2.362955768687E-02
 27   8     7.001017431963E-02 -1.873620964908E-01 -2.362955768687E-02

********************************************************************************

<< INFORMATION >>: FROM NOW ON, ALL COORDINATES REFER TO THE PRIMITIVE CELL

********************************************************************************

LATTICE PARAMETERS  (ANGSTROMS AND DEGREES) - PRIMITIVE CELL
      A          B          C         ALPHA     BETA     GAMMA        VOLUME
   7.06616    7.06616    9.29800    67.43555 112.56445 119.93596    360.639613

INPUT LIST - ATOM N.   3 NOT IRREDUCIBLE

INPUT LIST - ATOM N.   5 NOT IRREDUCIBLE

INPUT LIST - ATOM N.   9 NOT IRREDUCIBLE
```

```
INPUT LIST - ATOM N.  15 NOT IRREDUCIBLE

INPUT LIST - ATOM N.  21 NOT IRREDUCIBLE

INPUT LIST - ATOM N.  27 NOT IRREDUCIBLE

COORDINATES OF THE EQUIVALENT ATOMS (FRACTIONAL UNITS)

N. ATOM EQUIV AT. N.          X                  Y                  Z

  1   1   1   20 CA    2.78543461209E-01 -2.78543461209E-01  3.00688000640E-01

  2   2   1   20 CA    2.73822258949E-01  2.43191518821E-01  3.33840751701E-01
  3   2   2   20 CA   -2.43191518821E-01 -2.73822258949E-01  3.33840751701E-01


  4   4   1   20 CA   -4.77245552729E-01  5.41318619110E-03 -2.03841704673E-02
  5   4   2   20 CA   -5.41318619110E-03  4.77245552729E-01 -2.03841704673E-02


  6   6   1   20 CA   -3.03235302540E-01  3.03235302540E-01 -3.53748581686E-01

  7   7   1   20 CA   -4.72394953046E-01  4.72394953046E-01  2.00223409350E-02

  8   8   1   20 CA   -2.94849704560E-01 -2.57550960298E-01 -3.06572246846E-01
  9   8   2   20 CA    2.57550960298E-01  2.94849704560E-01 -3.06572246846E-01


 10  10   1   14 SI    2.19505551918E-01 -2.19505551918E-01 -3.48360242515E-01

 11  11   1   14 SI   -2.13308844427E-01  2.13308844427E-01  3.58463462447E-01

 12  12   1   14 SI    1.56311769126E-03 -1.56311769126E-03  1.31716787864E-03

 13  13   1    8 O     1.79943565362E-01 -1.79943565362E-01  4.59684079523E-01

 14  14   1    8 O    -3.71822013103E-01 -1.09849627790E-03  2.74650419673E-01
 15  14   2    8 O     1.09849627790E-03  3.71822013103E-01  2.74650419673E-01


 16  16   1    8 O    -3.58363015725E-01  3.58363015725E-01  3.24211538956E-01

 17  17   1    8 O     3.79703999939E-01 -3.79703999939E-01  1.15842393134E-01

 18  18   1    8 O    -1.32396760412E-01  1.32396760412E-01 -4.44246106071E-01

 19  19   1    8 O     3.68387256842E-01 -3.68387256842E-01 -2.41730993245E-01

 20  20   1    8 O     3.52065542995E-01  2.79355457173E-02 -3.06769055127E-01
 21  20   2    8 O    -2.79355457173E-02 -3.52065542995E-01 -3.06769055127E-01


 22  22   1    8 O     4.80199206302E-01 -4.80199206303E-01  4.95657824965E-01

 23  23   1    8 O    -3.75264632821E-01  3.75264632821E-01 -1.43591296677E-01

 24  24   1    8 O     2.50786484124E-03 -2.50786484124E-03  1.76821220922E-01

 25  25   1    8 O    -1.42990601171E-01  1.42990601171E-01 -1.21903091703E-01

 26  26   1    8 O     2.57372270810E-01  1.17351922171E-01 -2.36295576869E-02
 27  26   2    8 O    -1.17351922171E-01 -2.57372270810E-01 -2.36295576869E-02

NUMBER OF SYMMETRY OPERATORS         :    2
*******************************************************************************
* GEOMETRY EDITING - INPUT COORDINATES ARE GIVEN IN ANGSTROM
*******************************************************************************

GEOMETRY NOW FULLY CONSISTENT WITH THE GROUP

FRQFRQFRQFRQFRQFRQFRQFRQFRQFRQFRQFRQFRQFRQFRQFRQFRQFRQFRQFRQFRQFRQFRQFRQFRQFRQ

                      FREQUENCY CALCULATION


GCALCO - MAX INDICES DIRECT LATTICE VECTOR    16    16    12
NO.OF VECTORS CREATED 6999 STARS 1811 RMAX   159.70567 BOHR
```

```
GEOMETRY FOR WAVE FUNCTION - DIMENSIONALITY OF THE SYSTEM    3
(NON PERIODIC DIRECTION: LATTICE PARAMETER FORMALLY SET TO 500)
*******************************************************************************
LATTICE PARAMETERS (ANGSTROMS AND DEGREES) - BOHR = 0.5291772083 ANGSTROM
PRIMITIVE CELL - CENTRING CODE 4/0 VOLUME=   360.639613 - DENSITY  3.147 g/cm^3
        A              B              C           ALPHA      BETA       GAMMA
    7.06616151     7.06616151     9.29800000    67.435546 112.564454 119.935962
*******************************************************************************
ATOMS IN THE ASYMMETRIC UNIT   21 - ATOMS IN THE UNIT CELL:   27
    ATOM                 X/A                 Y/B                 Z/C
*******************************************************************************
   1 T  20 CA    2.785434612087E-01 -2.785434612087E-01  3.006880006403E-01
   2 T  20 CA    2.738222589492E-01  2.431915188212E-01  3.338407517012E-01
   3 F  20 CA   -2.431915188211E-01 -2.738222589493E-01  3.338407517012E-01
   4 T  20 CA   -4.772455527293E-01  5.413186191100E-03 -2.038417046735E-02
   5 F  20 CA   -5.413186191100E-03  4.772455527293E-01 -2.038417046735E-02
   6 T  20 CA   -3.032353025404E-01  3.032353025404E-01 -3.537485816856E-01
   7 T  20 CA   -4.723949530458E-01  4.723949530458E-01  2.002234093501E-02
   8 T  20 CA   -2.948497045602E-01 -2.575509602984E-01 -3.065722468464E-01
   9 F  20 CA    2.575509602984E-01  2.948497045602E-01 -3.065722468464E-01
  10 T  14 SI    2.195055519178E-01 -2.195055519178E-01 -3.483602425146E-01
  11 T  14 SI   -2.133088444268E-01  2.133088444268E-01  3.584634624474E-01
  12 T  14 SI    1.563117691256E-03 -1.563117691256E-03  1.317167878642E-03
  13 T   8 O     1.799435653619E-01 -1.799435653619E-01  4.596840795229E-01
  14 T   8 O    -3.718220131031E-01 -1.098496277900E-03  2.746504196733E-01
  15 F   8 O     1.098496277900E-03  3.718220131031E-01  2.746504196733E-01
  16 T   8 O    -3.583630157253E-01  3.583630157253E-01  3.242115389560E-01
  17 T   8 O     3.797039999385E-01 -3.797039999385E-01  1.158423931343E-01
  18 T   8 O    -1.323967604115E-01  1.323967604115E-01 -4.442461060711E-01
  19 T   8 O     3.683872568420E-01 -3.683872568420E-01 -2.417309932451E-01
  20 T   8 O     3.520655429949E-01  2.793554571730E-02 -3.067690551272E-01
  21 F   8 O    -2.793554571730E-02 -3.520655429949E-01 -3.067690551272E-01
  22 T   8 O     4.801992063025E-01 -4.801992063025E-01  4.956578249650E-01
  23 T   8 O    -3.752646328209E-01  3.752646328209E-01 -1.435912966766E-01
  24 T   8 O     2.507864841237E-03 -2.507864841237E-03  1.768212209225E-01
  25 T   8 O    -1.429906011707E-01  1.429906011707E-01 -1.219030917026E-01
  26 T   8 O     2.573722708104E-01  1.173519221712E-01 -2.362955768687E-02
  27 F   8 O    -1.173519221712E-01 -2.573722708104E-01 -2.362955768687E-02

TRANSFORMATION MATRIX PRIMITIVE-CRYSTALLOGRAPHIC CELL
 1.0000  1.0000  0.0000 -1.0000  1.0000  0.0000  0.0000  0.0000  1.0000

*******************************************************************************
CRYSTALLOGRAPHIC CELL (VOLUME=        721.27922538)
        A              B              C           ALPHA      BETA       GAMMA
   12.23500000     7.07300000     9.29800000    90.000000 116.310000  90.000000

COORDINATES IN THE CRYSTALLOGRAPHIC CELL
    ATOM                 X/A                 Y/B                 Z/C
*******************************************************************************
   1 T  20 CA    2.785434612087E-01 -3.793449615605E-18  3.006880006403E-01
   2 T  20 CA    1.531537006405E-02  2.585068888852E-01  3.338407517012E-01
   3 F  20 CA    1.531537006405E-02 -2.585068888852E-01  3.338407517012E-01
   4 T  20 CA   -2.413293694602E-01 -2.359161832691E-01 -2.038417046735E-02
   5 F  20 CA   -2.413293694602E-01  2.359161832691E-01 -2.038417046735E-02
   6 T  20 CA   -3.032353025404E-01  7.075015762172E-18 -3.537485816856E-01
   7 T  20 CA    2.760504695417E-02  5.000000000000E-01  2.002234093501E-02
   8 T  20 CA   -1.864937213094E-02 -2.762003324293E-01 -3.065722468464E-01
   9 F  20 CA   -1.864937213094E-02  2.762003324293E-01 -3.065722468464E-01
  10 T  14 SI   -2.804944480822E-01 -5.000000000000E-01 -3.483602425146E-01
  11 T  14 SI    2.866911555732E-01 -5.000000000000E-01  3.584634624474E-01
  12 T  14 SI    1.563117691256E-03 -2.077396347165E-19  1.317167878642E-03
  13 T   8 O     1.799435653619E-01 -7.302045304812E-18  4.596840795229E-01
  14 T   8 O     3.146382415874E-01  3.135397453095E-01  2.746504196733E-01
  15 F   8 O     3.146382415874E-01 -3.135397453095E-01  2.746504196733E-01
  16 T   8 O     1.416369842747E-01  5.000000000000E-01  3.242115389560E-01
  17 T   8 O    -1.202960000615E-01  5.000000000000E-01  1.158423931343E-01
  18 T   8 O    -1.323967604115E-01  9.830355798328E-19 -4.442461060711E-01
  19 T   8 O    -1.316127431580E-01 -5.000000000000E-01 -2.417309932451E-01
  20 T   8 O     1.620649986388E-01  1.900005443561E-01 -3.067690551272E-01
  21 F   8 O     1.620649986388E-01 -1.900005443561E-01 -3.067690551272E-01
  22 T   8 O     4.801992063025E-01  2.631541868425E-18  4.956578249650E-01
  23 T   8 O    -3.752646328209E-01 -1.657743352210E-17 -1.435912966766E-01
  24 T   8 O     2.507864841237E-03 -6.822595532689E-18  1.768212209225E-01
  25 T   8 O    -1.429906011707E-01 -1.422163765940E-17 -1.219030917026E-01
  26 T   8 O     7.001017431963E-02  1.873620964908E-01 -2.362955768687E-02
  27 F   8 O     7.001017431963E-02 -1.873620964908E-01 -2.362955768687E-02

T = ATOM BELONGING TO THE ASYMMETRIC UNIT
```

```
****   2 SYMMOPS - TRANSLATORS IN FRACTIONAL UNITS
 V INV                  ROTATION MATRICES                        TRANSLATOR
 1  1  1.00  0.00  0.00  0.00  1.00  0.00  0.00  0.00  1.00    0.00  0.00  0.00
 2  2  0.00 -1.00 -0.00 -1.00  0.00  0.00  0.00  0.00  1.00    0.00  0.00  0.00

DIRECT LATTICE VECTORS CARTESIAN COMPONENTS (ANGSTROM)
         X                    Y                    Z
  0.611750000000E+01   0.353650000000E+01   0.000000000000E+00
 -0.611750000000E+01   0.353650000000E+01   0.000000000000E+00
 -0.412113069411E+01   0.569338296924E-15   0.833481168366E+01


CARTESIAN COORDINATES - PRIMITIVE CELL
*******************************************************************************
*      ATOM          X(ANGSTROM)         Y(ANGSTROM)         Z(ANGSTROM)
*******************************************************************************
  1    20 CA    2.168804699100E+00  4.061950824945E-16  2.506177860873E+00
  2    20 CA   -1.188417816046E+00  1.828419225085E+00  2.782499797761E+00
  3    20 CA   -1.188417816046E+00 -1.828419225085E+00  2.782499797761E+00
  4    20 CA   -2.868659004759E+00 -1.668635164262E+00 -1.698982221730E-01
  5    20 CA   -2.868659004759E+00  1.668635164262E+00 -1.698982221730E-01
  6    20 CA   -2.252239788600E+00 -4.364045033406E-16 -2.948427811711E+00
  7    20 CA    2.552330656891E-01 -3.536500000000E+00  1.668824411593E-01
  8    20 CA    1.035249228418E+00 -1.953564951272E+00 -2.555221944901E+00
  9    20 CA    1.035249228418E+00  1.953564951272E+00 -2.555221944901E+00
 10    14 SI   -1.996211484252E+00 -3.536500000000E+00 -2.903517019433E+00
 11    14 SI    2.030391510630E+00 -3.536500000000E+00  2.987725454972E+00
 12    14 SI    1.369652397855E-02  7.499141167885E-19  1.097834622425E-02
 13     8 O     3.071913524881E-01  3.792166951108E-16  3.831380236800E+00
 14     8 O     2.717728611156E+00  2.217666618574E+00  2.289159526815E+00
 15     8 O     2.717728611156E+00 -2.217666618574E+00  2.289159526815E+00
 16     8 O     3.968103780253E-01 -3.536500000000E+00  2.702242122868E+00
 17     8 O    -1.949223202777E+00 -3.536500000000E+00  9.655245317589E-01
 18     8 O     2.109218998330E-01 -3.704272655978E-16 -3.702707635302E+00
 19     8 O    -6.140768965584E-01 -3.536500000000E+00 -2.014782306802E+00
 20     8 O     3.247100627433E+00  1.343873850231E+00 -2.556862304860E+00
 21     8 O     3.247100627433E+00 -1.343873850231E+00 -2.556862304860E+00
 22     8 O     3.832566612873E+00  7.522007585316E-16  4.131214630616E+00
 23     8 O    -3.999604282423E+00 -5.517558009121E-16 -1.196806417212E+00
 24     8 O    -6.980196345809E-01  5.477228647047E-17  1.473771578064E+00
 25     8 O    -1.247111432401E+00 -3.044059869243E-16 -1.016039312997E+00
 26     8 O     9.539549782722E-01  1.325212108479E+00 -1.969479134882E-01
 27     8 O     9.539549782722E-01 -1.325212108479E+00 -1.969479134882E-01

*******************************************************************************
LOCAL ATOMIC FUNCTIONS BASIS SET
*******************************************************************************
   ATOM  X(AU)  Y(AU)  Z(AU)    NO. TYPE  EXPONENT  S COEF   P COEF   D/F/G COEF
*******************************************************************************
  1 CA  4.098  0.000  4.736
                                     1 S
                                            1.913E+05 2.204E-04 0.000E+00 0.000E+00
                                            2.697E+04 1.925E-03 0.000E+00 0.000E+00
                                            5.696E+03 1.109E-02 0.000E+00 0.000E+00
                                            1.489E+03 4.995E-02 0.000E+00 0.000E+00
                                            4.483E+02 1.701E-01 0.000E+00 0.000E+00
                                            1.546E+02 3.685E-01 0.000E+00 0.000E+00
                                            6.037E+01 4.034E-01 0.000E+00 0.000E+00
                                            2.509E+01 1.452E-01 0.000E+00 0.000E+00
                                2-   5 SP
                                            4.486E+02-5.750E-03 8.470E-03 0.000E+00
                                            1.057E+02-7.670E-02 6.027E-02 0.000E+00
                                            3.469E+01-1.122E-01 2.124E-01 0.000E+00
                                            1.350E+01 2.537E-01 3.771E-01 0.000E+00
                                            5.820E+00 6.880E-01 4.010E-01 0.000E+00
                                            1.819E+00 3.490E-01 1.980E-01 0.000E+00
                                6-   9 SP
                                            2.075E+01-2.000E-03-3.650E-02 0.000E+00
                                            8.400E+00-1.255E-01-6.850E-02 0.000E+00
                                            3.597E+00-6.960E-01 1.570E-01 0.000E+00
                                            1.408E+00 1.029E+00 1.482E+00 0.000E+00
                                            7.260E-01 9.440E-01 1.025E+00 0.000E+00
                               10-  13 SP
                                            4.530E-01 1.000E+00 1.000E+00 0.000E+00
                               14-  17 SP
                                            2.950E-01 1.000E+00 1.000E+00 0.000E+00
                               18-  22 D
                                            3.191E+00 0.000E+00 0.000E+00 1.600E-01
```

```
                                                  8.683E-01 0.000E+00 0.000E+00 3.130E-01
                                   23-  27 D
                                                  2.891E-01 0.000E+00 0.000E+00 4.060E-01
  2 CA -2.246  3.455  5.258
  3 CA -2.246 -3.455  5.258
  4 CA -5.421 -3.153 -0.321
  5 CA -5.421  3.153 -0.321
  6 CA -4.256 -0.000 -5.572
  7 CA  0.482 -6.683  0.315
  8 CA  1.956 -3.692 -4.829
  9 CA  1.956  3.692 -4.829
 10 SI -3.772 -6.683 -5.487
                                       244 S
                                                  8.765E+04 2.370E-04 0.000E+00 0.000E+00
                                                  1.285E+04 1.920E-03 0.000E+00 0.000E+00
                                                  2.786E+03 1.090E-02 0.000E+00 0.000E+00
                                                  7.280E+02 4.960E-02 0.000E+00 0.000E+00
                                                  2.195E+02 1.668E-01 0.000E+00 0.000E+00
                                                  7.590E+01 3.630E-01 0.000E+00 0.000E+00
                                                  2.946E+01 4.051E-01 0.000E+00 0.000E+00
                                                  1.199E+01 1.504E-01 0.000E+00 0.000E+00
                                  245- 248 SP
                                                  1.660E+02-8.840E-03 9.090E-03 0.000E+00
                                                  3.937E+01-8.590E-02 6.010E-02 0.000E+00
                                                  1.271E+01-7.120E-02 1.952E-01 0.000E+00
                                                  4.777E+00 4.147E-01 3.384E-01 0.000E+00
                                                  1.848E+00 6.168E-01 3.006E-01 0.000E+00
                                                  7.365E-01 1.154E-01 6.480E-02 0.000E+00
                                  249- 252 SP
                                                  4.175E+00-1.990E-02-8.700E-03 0.000E+00
                                                  1.447E+00-1.864E-01-4.380E-03 0.000E+00
                                                  5.023E-01 9.670E-02 2.207E-01 0.000E+00
                                  253- 256 SP
                                                  3.220E-01 1.000E+00 1.000E+00 0.000E+00
                                  257- 260 SP
                                                  1.300E-01 1.000E+00 1.000E+00 0.000E+00
                                  261- 265 D
                                                  6.000E-01 0.000E+00 0.000E+00 1.000E+00
 11 SI  3.837 -6.683  5.646
 12 SI  0.026  0.000  0.021
 13 O   0.581  0.000  7.240
                                       310 S
                                                  8.020E+03 1.080E-03 0.000E+00 0.000E+00
                                                  1.338E+03 8.040E-03 0.000E+00 0.000E+00
                                                  2.554E+02 5.324E-02 0.000E+00 0.000E+00
                                                  6.922E+01 1.681E-01 0.000E+00 0.000E+00
                                                  2.390E+01 3.581E-01 0.000E+00 0.000E+00
                                                  9.264E+00 3.855E-01 0.000E+00 0.000E+00
                                                  3.851E+00 1.468E-01 0.000E+00 0.000E+00
                                                  1.212E+00 7.280E-02 0.000E+00 0.000E+00
                                  311- 314 SP
                                                  4.943E+01-8.830E-03 9.580E-03 0.000E+00
                                                  1.047E+01-9.150E-02 6.960E-02 0.000E+00
                                                  3.235E+00-4.020E-02 2.065E-01 0.000E+00
                                                  1.217E+00 3.790E-01 3.470E-01 0.000E+00
                                  315- 318 SP
                                                  4.860E-01 1.000E+00 1.000E+00 0.000E+00
                                  319- 322 SP
                                                  1.925E-01 1.000E+00 1.000E+00 0.000E+00
                                  323- 327 D
                                                  2.000E+00 0.000E+00 0.000E+00 1.000E+00
                                  328- 332 D
                                                  5.000E-01 0.000E+00 0.000E+00 1.000E+00
 14 O   5.136  4.191  4.326
 15 O   5.136 -4.191  4.326
 16 O   0.750 -6.683  5.106
 17 O  -3.683 -6.683  1.825
 18 O   0.399 -0.000 -6.997
 19 O  -1.160 -6.683 -3.807
 20 O   6.136  2.540 -4.832
 21 O   6.136 -2.540 -4.832
 22 O   7.243  0.000  7.807
 23 O  -7.558 -0.000 -2.262
 24 O  -1.319  0.000  2.785
 25 O  -2.357 -0.000 -1.920
 26 O   1.803  2.504 -0.372
 27 O   1.803 -2.504 -0.372
INFORMATION **** READM2 **** FULL DIRECT SCF (MONO AND BIEL INT) SELECTED
INFORMATION **** TOLINTEG **** COULOMB AND EXCHANGE SERIES TOLERANCES MODIFIED
```

```
INFORMATION **** MAXCYCLE **** MAX NUMBER OF SCF CYCLES SET TO         800
INFORMATION **** LEVSHIFT **** LEVEL SHIFTER ACTIVE
INFORMATION **** TOLDEE **** SCF TOL ON TOTAL ENERGY SET TO          10
*******************************************************************************
N. OF ATOMS PER CELL       27  COULOMB OVERLAP TOL         (T1) 10** -6
NUMBER OF SHELLS          171  COULOMB PENETRATION TOL     (T2) 10** -6
NUMBER OF AO              654  EXCHANGE OVERLAP TOL        (T3) 10** 20
N. OF ELECTRONS PER CELL  342  EXCHANGE PSEUDO OVP (F(G))  (T4) 10** 20
CORE ELECTRONS PER CELL   222  EXCHANGE PSEUDO OVP (P(G))  (T5) 10** 20
N. OF SYMMETRY OPERATORS    2  POLE ORDER IN MONO ZONE             4
*******************************************************************************
TYPE OF CALCULATION :  RESTRICTED CLOSED SHELL
KOHN-SHAM HAMILTONIAN

(EXCHANGE)[CORRELATION] FUNCTIONAL:(DIRAC-SLATER LDA)[VOSKO-WILK-NUSAIR]

EIGENVALUE LEVEL SHIFTING OF  0.600 HARTREE
LOCKING - FERMI ENERGY ALTERED BY LEVEL SHIFTER

CAPPA:IS1  4;IS2  4;IS3  4; K PTS MONK NET   24; SYMMOPS: K SPACE  4;G SPACE  2

*******************************************************************************
MAX NUMBER OF SCF CYCLES     800  CONVERGENCE ON DELTAP        10**-20
WEIGHT OF F(I) IN F(I+1)      75% CONVERGENCE ON ENERGY        10**-10
EIGENVALUE LEVEL SHIFTING OF  0.600 HARTREE
LOCKING - FERMI ENERGY ALTERED BY LEVEL SHIFTER
SHRINK. FACT.(MONKH.)    4  4  4  NUMBER OF K POINTS IN THE IBZ     24
SHRINKING FACTOR(GILAT NET)    4  NUMBER OF K POINTS(GILAT NET)     24
*******************************************************************************
*** K POINTS COORDINATES (OBLIQUE COORDINATES IN UNITS OF IS =  4)
  1-R(  0  0  0)   2-C(  1  0  0)   3-R(  2  0  0)   4-C(  1  1  0)
  5-C(  2  1  0)   6-C(  3  1  0)   7-R(  2  2  0)   8-C(  0  0  1)
  9-C(  1  0  1)  10-C(  2  0  1)  11-C(  3  0  1)  12-C(  1  1  1)
 13-C(  2  1  1)  14-C(  3  1  1)  15-C(  1  2  1)  16-C(  2  2  1)
 17-C(  1  3  1)  18-R(  0  0  2)  19-C(  1  0  2)  20-R(  2  0  2)
 21-C(  1  1  2)  22-C(  2  1  2)  23-C(  3  1  2)  24-R(  2  2  2)

DIRECT LATTICE VECTORS COMPON. (A.U.)     RECIP. LATTICE VECTORS COMPON. (A.U.)
       X             Y            Z              X            Y            Z
  11.5603996    6.6830165    0.0000000      0.2717547    0.4700860    0.1343685
 -11.5603996    6.6830165    0.0000000     -0.2717547    0.4700860   -0.1343685
  -7.7878084    0.0000000   15.7505115      0.0000000    0.0000000    0.3989194

DISK SPACE FOR EIGENVECTORS (FTN 10)   17964072 REALS

SYMMETRY ADAPTION OF THE BLOCH FUNCTIONS ENABLED
TTTTTTTTTTTTTTTTTTTTTTTTTTTTTT gordsh1     TELAPSE        0.02 TCPU        0.01

DIMENSIONS  P(G)=  345586 F(G)=  173156 P(G),F(G) (IRR)  103599
MAX G-VECTOR INDEX FOR 1- AND 2-ELECTRON INTEGRALS  33

TTTTTTTTTTTTTTTTTTTTTTTTTTTTTT INPUT       TELAPSE        0.03 TCPU        0.02

NEIGHBORS OF THE NON-EQUIVALENT ATOMS

N = NUMBER OF NEIGHBORS AT DISTANCE R
   ATOM  N     R/ANG      R/AU   NEIGHBORS (ATOM LABELS AND CELL INDICES)
  1 CA   1     2.2851     4.3182  13 O    0 0 0
  1 CA   2     2.2949     4.3367  14 O    0 0 0  15 O    0 0 0
  1 CA   1     2.3257     4.3949  22 O    0 0 0
  1 CA   1     2.5242     4.7700  17 O    1 0 0
  1 CA   1     3.0471     5.7581  24 O    0 0 0
  1 CA   2     3.2464     6.1348  26 O    0 0 0  27 O    0 0 0

  2 CA   1     2.3014     4.3490  24 O    0 0 0
  2 CA   1     2.3223     4.3885  15 O    0 1 0
  2 CA   1     2.3317     4.4063  16 O    1 1 0
  2 CA   1     2.4370     4.6052  22 O    0 1 0
  2 CA   1     2.5846     4.8842  13 O    0 0 0
  2 CA   1     2.6073     4.9270  17 O    1 1 0

  4 CA   1     2.2623     4.2751  23 O    0 0 0
  4 CA   1     2.3583     4.4566  26 O   -1 0 0
  4 CA   1     2.3714     4.4813  17 O    0 0 0
  4 CA   1     2.4438     4.6181  20 O   -1 0 0
  4 CA   1     2.4758     4.6786  25 O    0 0 0
  4 CA   1     2.5400     4.7998  14 O   -1 0 0

  6 CA   1     2.1782     4.1161  25 O    0 0 0
```

```
  6 CA   2     2.3115     4.3681  20 O   -1 0 0  21 O    0 1 0
  6 CA   1     2.3859     4.5087  22 O   -1 1-1
  6 CA   1     2.4742     4.6755  23 O    0 0 0
  6 CA   1     2.5761     4.8681  18 O    0 0 0
  6 CA   1     2.7624     5.2201  16 O    0 1-1

  7 CA   1     2.3085     4.3624  23 O    0-1 0
  7 CA   1     2.3447     4.4308  17 O    0 0 0
  7 CA   2     2.3474     4.4360  26 O   -1-1 0  27 O    0 0 0
  7 CA   1     2.3485     4.4380  19 O    0 0 0
  7 CA   1     2.5393     4.7986  16 O    0 0 0
  7 CA   2     3.2441     6.1304   8 CA   0 0 0   9 CA  -1-1 0

  8 CA   1     2.2943     4.3357  21 O    0 0 0
  8 CA   1     2.3490     4.4391  19 O    0 0 0
  8 CA   1     2.3501     4.4411  23 O    0-1 0
  8 CA   1     2.4109     4.5560  18 O    0 0 0
  8 CA   1     2.4216     4.5762  22 O   -1 0-1
  8 CA   1     2.4419     4.6145  27 O    0 0 0

 10 SI   1     1.6291     3.0786  13 O   -1 0-1
 10 SI   2     1.6402     3.0996  20 O   -1 0 0  21 O   -1 0 0
 10 SI   1     1.6432     3.1052  19 O    0 0 0
 10 SI   2     3.4239     6.4701   4 CA   0 0 0   5 CA  -1-1 0
 10 SI   2     3.4310     6.4837   2 CA  -1 0-1   3 CA  -1 0-1
 10 SI   2     3.4375     6.4960   8 CA   0 0 0   9 CA  -1-1 0

 11 SI   2     1.6431     3.1050  14 O   -1-1 0  15 O    0 0 0
 11 SI   1     1.6539     3.1254  18 O    0-1 1
 11 SI   1     1.6583     3.1338  16 O    0 0 0
 11 SI   1     3.3137     6.2620   6 CA   0-1 1
 11 SI   1     3.3329     6.2983   7 CA   0 0 0
 11 SI   2     3.4333     6.4880   2 CA   0-1 0   3 CA   0-1 0

 12 SI   1     1.6262     3.0730  25 O    0 0 0
 12 SI   1     1.6267     3.0741  24 O    0 0 0
 12 SI   2     1.6381     3.0956  26 O    0 0 0  27 O    0 0 0
 12 SI   1     3.2970     6.2305   1 CA   0 0 0
 12 SI   2     3.3354     6.3030   4 CA   0 0 0   5 CA   0 0 0
 12 SI   2     3.3831     6.3931   8 CA   0 0 0   9 CA   0 0 0

 13 O    1     1.6291     3.0786  10 SI   1 0 1
 13 O    1     2.2851     4.3182   1 CA   0 0 0
 13 O    1     2.5630     4.8433  24 O    0 0 0
 13 O    2     2.5846     4.8842   2 CA   0 0 0   3 CA   0 0 0
 13 O    2     2.6439     4.9963  20 O    0 0 1  21 O    0 0 1
 13 O    1     2.7109     5.1229  19 O    1 0 1

 14 O    1     1.6431     3.1050  11 SI   1 1 0
 14 O    1     2.2949     4.3367   1 CA   0 0 0
 14 O    1     2.3223     4.3885   3 CA   1 0 0
 14 O    1     2.5400     4.7998   4 CA   1 0 0
 14 O    1     2.6377     4.9845  15 O    1 1 0
 14 O    1     2.7012     5.1046  16 O    1 1 0

 16 O    1     1.6583     3.1338  11 SI   0 0 0
 16 O    2     2.3317     4.4063   2 CA  -1-1 0   3 CA   0 0 0
 16 O    1     2.5393     4.7986   7 CA   0 0 0
 16 O    1     2.6462     5.0005  18 O    0-1 1
 16 O    2     2.7012     5.1046  14 O   -1-1 0  15 O    0 0 0
 16 O    1     2.7624     5.2201   6 CA   0-1 1

 17 O    1     2.3447     4.4308   7 CA   0 0 0
 17 O    2     2.3714     4.4813   4 CA   0 0 0   5 CA  -1-1 0
 17 O    1     2.5242     4.7700   1 CA  -1 0 0
 17 O    2     2.6073     4.9270   2 CA  -1-1 0   3 CA   0 0 0
 17 O    1     2.9189     5.5159  16 O    0 0 0
 17 O    2     2.9621     5.5976  14 O   -1 0 0  15 O   -1 0 0

 18 O    1     1.6539     3.1254  11 SI   0 1-1
 18 O    2     2.4109     4.5560   8 CA   0 0 0   9 CA   0 0 0
 18 O    1     2.5761     4.8681   6 CA   0 0 0
 18 O    1     2.6462     5.0005  16 O    0 1-1
 18 O    2     2.7367     5.1715  14 O   -1 0-1  15 O    0 1-1
 18 O    1     3.0568     5.7765  25 O    0 0 0

 19 O    1     1.6432     3.1052  10 SI   0 0 0
 19 O    1     2.3485     4.4380   7 CA   0 0 0
 19 O    2     2.3490     4.4391   8 CA   0 0 0   9 CA  -1-1 0
```

```
 19 O    2     2.6816     5.0674  20 O   -1 0 0  21 O   -1 0 0
 19 O    1     2.7109     5.1229  13 O   -1 0-1
 19 O    1     2.8518     5.3891  23 O    0-1 0

 20 O    1     1.6402     3.0996  10 SI   1 0 0
 20 O    1     2.2943     4.3357   9 CA   0 0 0
 20 O    1     2.3115     4.3681   6 CA   1 0 0
 20 O    1     2.4438     4.6181   4 CA   1 0 0
 20 O    1     2.6439     4.9963  13 O    0 0-1
 20 O    1     2.6816     5.0674  19 O    1 0 0

 22 O    1     2.3257     4.3949   1 CA   0 0 0
 22 O    1     2.3859     4.5087   6 CA   1-1 1
 22 O    2     2.4216     4.5762   8 CA   1 0 1   9 CA   0-1 1
 22 O    2     2.4370     4.6052   2 CA   0-1 0   3 CA   1 0 0
 22 O    1     3.0200     5.7069  23 O    1-1 1
 22 O    1     3.0387     5.7423  16 O    1 0 0

 23 O    2     2.2623     4.2751   4 CA   0 0 0   5 CA   0 0 0
 23 O    1     2.3085     4.3624   7 CA   0 1 0
 23 O    2     2.3501     4.4411   8 CA   0 1 0   9 CA  -1 0 0
 23 O    1     2.4742     4.6755   6 CA   0 0 0
 23 O    2     2.6915     5.0862  26 O   -1 0 0  27 O    0 1 0
 23 O    1     2.7584     5.2127  25 O    0 0 0

 24 O    1     1.6267     3.0741  12 SI   0 0 0
 24 O    2     2.3014     4.3490   2 CA   0 0 0   3 CA   0 0 0
 24 O    1     2.5496     4.8181  25 O    0 0 0
 24 O    1     2.5630     4.8433  13 O    0 0 0
 24 O    2     2.6975     5.0975  26 O    0 0 0  27 O    0 0 0
 24 O    1     3.0471     5.7581   1 CA   0 0 0

 25 O    1     1.6262     3.0730  12 SI   0 0 0
 25 O    1     2.1782     4.1161   6 CA   0 0 0
 25 O    2     2.4758     4.6786   4 CA   0 0 0   5 CA   0 0 0
 25 O    1     2.5496     4.8181  24 O    0 0 0
 25 O    2     2.6966     5.0959  26 O    0 0 0  27 O    0 0 0
 25 O    1     2.7584     5.2127  23 O    0 0 0

 26 O    1     1.6381     3.0956  12 SI   0 0 0
 26 O    1     2.3474     4.4360   7 CA   1 1 0
 26 O    1     2.3583     4.4566   4 CA   1 0 0
 26 O    1     2.4419     4.6145   9 CA   0 0 0
 26 O    1     2.6504     5.0086  27 O    0 0 0
 26 O    1     2.6915     5.0862  23 O    1 0 0

SYMMETRY ALLOWED INTERNAL DEGREE(S) OF FREEDOM:  46
TTTTTTTTTTTTTTTTTTTTTTTTTTTTTT SYMM        TELAPSE        0.10 TCPU        0.09
TTTTTTTTTTTTTTTTTTTTTTTTTTTTTT INT_SCREEN  TELAPSE        0.10 TCPU        0.09

*******************************************************************************
*                                                                             *
*                                                                             *
*         FFFFF  RRRR   EEEE   EEE   U   U  EEEE  N   N   CCC  Y   Y          *
*         F      R   R  E     E   E  U   U  E     NN  N  C      Y Y           *
*         FFF    RRRR   EEEE  E   E  U   U  EEEE  N N N  C       Y            *
*         F      R   R  E     E  EE  U   U  E     N  NN  C       Y            *
*         F      R   R  EEEE   EE E   UUU   EEEE  N   N   CCC    Y            *
*                                                                             *
*                                                                             *
* CALCULATION OF PHONON FREQUENCIES AT THE GAMMA POINT.                       *
*                                                                             *
* SYMMETRY IS EXPLOITED TO BUILD THE TOTAL HESSIAN MATRIX.                    *
* (F. PASCALE PHD THESIS TURIN-PARIS 2002)                                    *
*                                                                             *
*******************************************************************************


*                                                                             *
* REFERENCES TO BE QUOTED WHEN USING THIS MODULE:                             *
*                                                                             *
* F. Pascale, C.M. Zicovich-Wilson, F. Lopez, B. Civalleri                    *
* R. Orlando, R. Dovesi                                                       *
* The calculation of the vibration frequencies of crystalline                 *
* compounds and its implementation in the CRYSTAL code                        *
* J. Comput. Chem. 25 (2004) 888-897                                          *
*                                                                             *
* C.M. Zicovich-Wilson, F. Pascale, C. Roetti, V.R. Saunders,                 *
```

```
* R. Orlando, R. Dovesi                                                      *
* The calculation of the vibration frequencies of alpha-quartz:              *
* the effect of hamiltonian and basis set                                    *
* J. Comput. Chem. 25 (2004) 1873-1881                                       *
******************************************************************************



ATOMS ISOTOPIC MASS (AMU) FOR FREQUENCY CALCULATION

   1 CA     39.9626    2 CA     39.9626    3 CA     39.9626    4 CA     39.9626
   5 CA     39.9626    6 CA     39.9626    7 CA     39.9626    8 CA     39.9626
   9 CA     39.9626   10 SI     27.9769   11 SI     27.9769   12 SI     27.9769
  13 O      15.9949   14 O      15.9949   15 O      15.9949   16 O      15.9949
  17 O      15.9949   18 O      15.9949   19 O      15.9949   20 O      15.9949
  21 O      15.9949   22 O      15.9949   23 O      15.9949   24 O      15.9949
  25 O      15.9949   26 O      15.9949   27 O      15.9949

STEP SIZE                  0.0030 ANGSTROM

INFORMATION CONCERNING THE SCF+GRADIENT CALCULATIONS REQUIRED FOR
GENERATING FREQUENCIES. IN PRINCIPLE 3N+1 SCF + GRADIENT
CALCULATIONS  ARE REQUIRED;
FOR EACH OF THEM THE REMAINING POINT SYMMETRY IS INDICATED.
POINT SYMMETRY PERMITS TO GENERATE GRADIENTS FOR DISPLACEMENT B
STARTING FROM THE GRADIENT GENERATED BY DISPLACEMENT A.

  N   LABEL SYMBOL DISPLACEMENT     SYM.

  1     EQUILIBRIUM GEOMETRY            2

  2      1   CA           DX        GENERATED BY TRANSLATIONAL INVARIANCE
  3      1   CA           DY        GENERATED BY TRANSLATIONAL INVARIANCE
  4      1   CA           DZ        GENERATED BY TRANSLATIONAL INVARIANCE
  5      2   CA           DX            1
  6      2   CA           DY            1
  7      2   CA           DZ            1
  8      4   CA           DX            1
  9      4   CA           DY            1
 10      4   CA           DZ            1
 11      6   CA           DX            2
 12      6   CA           DY            1
 13      6   CA           DZ            2
 14      7   CA           DX            2
 15      7   CA           DY            1
 16      7   CA           DZ            2
 17      8   CA           DX            1
 18      8   CA           DY            1
 19      8   CA           DZ            1
 20     10   SI           DX            2
 21     10   SI           DY            1
 22     10   SI           DZ            2
 23     11   SI           DX            2
 24     11   SI           DY            1
 25     11   SI           DZ            2
 26     12   SI           DX            2
 27     12   SI           DY            1
 28     12   SI           DZ            2
 29     13   O            DX            2
 30     13   O            DY            1
 31     13   O            DZ            2
 32     14   O            DX            1
 33     14   O            DY            1
 34     14   O            DZ            1
 35     16   O            DX            2
 36     16   O            DY            1
 37     16   O            DZ            2
 38     17   O            DX            2
 39     17   O            DY            1
 40     17   O            DZ            2
 41     18   O            DX            2
 42     18   O            DY            1
 43     18   O            DZ            2
 44     19   O            DX            2
 45     19   O            DY            1
 46     19   O            DZ            2
 47     20   O            DX            1
 48     20   O            DY            1
 49     20   O            DZ            1
```

50	22	O	DX	2
51	22	O	DY	1
52	22	O	DZ	2
53	23	O	DX	2
54	23	O	DY	1
55	23	O	DZ	2
56	24	O	DX	2
57	24	O	DY	1
58	24	O	DZ	2
59	25	O	DX	2
60	25	O	DY	1
61	25	O	DZ	2
62	26	O	DX	1
63	26	O	DY	1
64	26	O	DZ	1

USE OF RESIDUAL SYMMETRY AFTER DISPLACEMENT

NUMERICAL GRADIENT COMPUTED WITH A SINGLE DISPLACEMENT (+dx) FOR EACH
CARTESIAN COORDINATE WITH RESPECT TO THE EQUILIBRIUM CONFIGURATION
dx= 0.003
NUMBER OF IRREDUCIBLE ATOMS 21
NUMBER OF SCF+GRADIENT CALCULATIONS 61

ATOM	SYMOP	ORDER
1	2	2
2	1	1
4	1	1
6	2	2
7	2	2
8	1	1
10	2	2
11	2	2
12	2	2
13	2	2
14	1	1
16	2	2
17	2	2
18	2	2
19	2	2
20	1	1
22	2	2
23	2	2
24	2	2
25	2	2
26	1	1

ATOM : IRREDUCIBLE ATOM
SYMOP : NUMBER OF SYMMETRY OPERATORS THAT DOESN'T MOVE THE IRREDUCIBLE ATOM
ORDER : MAXIMUM ORDER AMONG THE OPERATORS OF THE IRREDUCIBLE ATOM

****

GCALCO - MAX INDICES DIRECT LATTICE VECTOR 16 16 12
NO.OF VECTORS CREATED 6999 STARS 1811 RMAX 159.70567 BOHR

CAPPA:IS1 4;IS2 4;IS3 4; K PTS MONK NET 24; SYMMOPS: K SPACE 4;G SPACE 2

TTTTTTTTTTTTTTTTTTTTTTTTTTTTTT gordsh1 TELAPSE 0.11 TCPU 0.10

DIMENSIONS P(G)= 345586 F(G)= 173156 P(G),F(G) (IRR) 103599
MAX G-VECTOR INDEX FOR 1- AND 2-ELECTRON INTEGRALS 33

TTTTTTTTTTTTTTTTTTTTTTTTTTTTTT INPUT TELAPSE 0.12 TCPU 0.11

SYMMETRY ALLOWED INTERNAL DEGREE(S) OF FREEDOM: 46
TTTTTTTTTTTTTTTTTTTTTTTTTTTTTT SYMM TELAPSE 0.18 TCPU 0.17
TTTTTTTTTTTTTTTTTTTTTTTTTTTTTT INT_SCREEN TELAPSE 0.18 TCPU 0.17
INFORMATION **** EXCBUF **** EXCH. BIPO BUFFER: WORDS USED = 0

DFT PARAMETERS

ATOM			ELECTRONS	NET CHARGE	R(ANGSTROM)
1	20	CA	20.0000	0.0000	1.97000000
10	14	SI	14.0000	0.0000	1.17000000
13	8	O	8.0000	0.0000	0.74000000

SIZE OF GRID= 286574
TTTTTTTTTTTTTTTTTTTTTTTTTTTTTT MAKE_GRID2 TELAPSE 17.46 TCPU 17.45

```
BECKE WEIGHT FUNCTION
RADSAFE =     2.00
TOLERANCES - DENSITY:10**- 6; POTENTIAL:10**- 9; GRID WGT:10**-14

RADIAL INTEGRATION  - INTERVALS (POINTS,UPPER LIMIT):           1( 75,  4.0*R)

ANGULAR INTEGRATION - INTERVALS (ACCURACY LEVEL [N. POINTS] UPPER LIMIT):
 1(  4[  86]   0.2)  2(  8[ 194]   0.5)  3( 12[ 350]   0.9)  4( 16[ 974]   3.5)
 5( 12[ 350]9999.0)
TTTTTTTTTTTTTTTTTTTTTTTTTTTTTT INT_CALC    TELAPSE       17.51 TCPU       17.50

******************************************************************************
Ca3SiO5 Cm  81100 ICSD W G Mumme LDA i VWN PP
CRYSTAL - SCF - TYPE OF CALCULATION :  RESTRICTED CLOSED SHELL
******************************************************************************

CAPPA:IS1  4;IS2  4;IS3  4; K PTS MONK NET   24; SYMMOPS: K SPACE  4;G SPACE  2

TTTTTTTTTTTTTTTTTTTTTTTTTTTTTT SDIK        TELAPSE       17.74 TCPU       17.73

AAAAAAAAAAAAAAAAAAAAAAAAAAAAAAAAAAAAAAAAAAAAAAAAAAAAAAAAAAAAAAAAAAAAAAAAAAAAAA
        ATOMIC WAVEFUNCTION(S)


NUCLEAR CHARGE 20.0  SYMMETRY SPECIES            S    P
N. ELECTRONS   20.0  NUMBER OF PRIMITIVE GTOS   21   13
                     NUMBER OF CONTRACTED GTOS   5    4
                     NUMBER OF CLOSED SHELLS     4    2
                     OPEN SHELL OCCUPATION       0    0

 ZNUC SCFIT  TOTAL HF ENERGY   KINETIC ENERGY   VIRIAL THEOREM ACCURACY
 20.0   7   -6.759945013E+02  6.789840598E+02 -1.995597012E+00  3.2E-06

NUCLEAR CHARGE 14.0  SYMMETRY SPECIES            S    P
N. ELECTRONS   14.0  NUMBER OF PRIMITIVE GTOS   19   11
                     NUMBER OF CONTRACTED GTOS   5    4
                     NUMBER OF CLOSED SHELLS     3    1
                     OPEN SHELL OCCUPATION       0    2

 ZNUC SCFIT  TOTAL HF ENERGY   KINETIC ENERGY   VIRIAL THEOREM ACCURACY
 14.0  10   -2.888271346E+02  2.895266850E+02 -1.997583814E+00  2.8E-06

NUCLEAR CHARGE  8.0  SYMMETRY SPECIES            S    P
N. ELECTRONS    8.0  NUMBER OF PRIMITIVE GTOS   14    6
                     NUMBER OF CONTRACTED GTOS   4    3
                     NUMBER OF CLOSED SHELLS     2    0
                     OPEN SHELL OCCUPATION       0    4

 ZNUC SCFIT  TOTAL HF ENERGY   KINETIC ENERGY   VIRIAL THEOREM ACCURACY
  8.0  13   -7.480044816E+01  7.461868841E+01 -2.002435848E+00  2.7E-06

AAAAAAAAAAAAAAAAAAAAAAAAAAAAAAAAAAAAAAAAAAAAAAAAAAAAAAAAAAAAAAAAAAAAAAAAAAAAAA

TTTTTTTTTTTTTTTTTTTTTTTTTTTTTT MOQGAD      TELAPSE       18.25 TCPU       18.24
TTTTTTTTTTTTTTTTTTTTTTTTTTTTTT SHELLX      TELAPSE       22.65 TCPU       22.64
TTTTTTTTTTTTTTTTTTTTTTTTTTTTTT MONMO3      TELAPSE       23.46 TCPU       23.45
NUMERICALLY INTEGRATED DENSITY    342.0001584345
TTTTTTTTTTTTTTTTTTTTTTTTTTTTTT NUMDFT      TELAPSE       24.18 TCPU       24.17
CYC   0 ETOT(AU) -8.063283847514E+03 DETOT -8.06E+03 tst  0.00E+00 PX  1.00E+00
TTTTTTTTTTTTTTTTTTTTTTTTTTTTTT FDIK        TELAPSE       40.82 TCPU       40.81
INSULATING STATE - LEVEL SHIFTER    0.60 au
TOP OF VALENCE BANDS -    BAND    171; K    1; EIG -3.0283175E-01 AU
BOTTOM OF VIRTUAL BANDS - BAND    172; K    1; EIG -4.3152521E-02 AU
TTTTTTTTTTTTTTTTTTTTTTTTTTTTTT PDIG        TELAPSE       41.13 TCPU       41.12
TTTTTTTTTTTTTTTTTTTTTTTTTTTTTT MOQGAD      TELAPSE       41.15 TCPU       41.14
TTTTTTTTTTTTTTTTTTTTTTTTTTTTTT SHELLX      TELAPSE       46.56 TCPU       46.55
TTTTTTTTTTTTTTTTTTTTTTTTTTTTTT MONMO3      TELAPSE       47.37 TCPU       47.36
NUMERICALLY INTEGRATED DENSITY    342.0002379957
TTTTTTTTTTTTTTTTTTTTTTTTTTTTTT NUMDFT      TELAPSE       48.85 TCPU       48.84
CYC   1 ETOT(AU) -8.067609818095E+03 DETOT -4.33E+00 tst  0.00E+00 PX  1.00E+00
TTTTTTTTTTTTTTTTTTTTTTTTTTTTTT FDIK        TELAPSE       53.89 TCPU       53.88
INSULATING STATE - LEVEL SHIFTER    0.60 au
TOP OF VALENCE BANDS -    BAND    171; K    1; EIG -7.6875578E-01 AU
BOTTOM OF VIRTUAL BANDS - BAND    172; K    1; EIG  4.8307521E-03 AU
TTTTTTTTTTTTTTTTTTTTTTTTTTTTTT PDIG        TELAPSE       54.20 TCPU       54.19
TTTTTTTTTTTTTTTTTTTTTTTTTTTTTT MOQGAD      TELAPSE       54.22 TCPU       54.21
TTTTTTTTTTTTTTTTTTTTTTTTTTTTTT SHELLX      TELAPSE       59.63 TCPU       59.62
TTTTTTTTTTTTTTTTTTTTTTTTTTTTTT MONMO3      TELAPSE       60.45 TCPU       60.44
NUMERICALLY INTEGRATED DENSITY    342.0002456724
```

```
TTTTTTTTTTTTTTTTTTTTTTTTTTTTTT NUMDFT      TELAPSE       61.90 TCPU       61.89
CYC   2 ETOT(AU) -8.069243744910E+03 DETOT -1.63E+00 tst  2.70E-03 PX  8.07E-02
TTTTTTTTTTTTTTTTTTTTTTTTTTTTTT FDIK        TELAPSE       67.02 TCPU       67.01
INSULATING STATE - LEVEL SHIFTER    0.60 au
TOP OF VALENCE BANDS -    BAND    171; K    1; EIG -7.1009670E-01 AU
BOTTOM OF VIRTUAL BANDS - BAND    172; K    1; EIG  2.9096249E-02 AU
TTTTTTTTTTTTTTTTTTTTTTTTTTTTTT PDIG        TELAPSE       67.33 TCPU       67.32
TTTTTTTTTTTTTTTTTTTTTTTTTTTTTT MOQGAD      TELAPSE       67.35 TCPU       67.34
TTTTTTTTTTTTTTTTTTTTTTTTTTTTTT SHELLX      TELAPSE       72.76 TCPU       72.75
TTTTTTTTTTTTTTTTTTTTTTTTTTTTTT MONMO3      TELAPSE       73.58 TCPU       73.57
NUMERICALLY INTEGRATED DENSITY    342.0002448806
TTTTTTTTTTTTTTTTTTTTTTTTTTTTTT NUMDFT      TELAPSE       75.03 TCPU       75.02
CYC   3 ETOT(AU) -8.070080462805E+03 DETOT -8.37E-01 tst  2.16E-03 PX  7.68E-02
TTTTTTTTTTTTTTTTTTTTTTTTTTTTTT FDIK        TELAPSE       79.84 TCPU       79.83
INSULATING STATE - LEVEL SHIFTER    0.60 au
TOP OF VALENCE BANDS -    BAND    171; K    1; EIG -7.0072549E-01 AU
BOTTOM OF VIRTUAL BANDS - BAND    172; K    1; EIG  3.8513570E-02 AU
TTTTTTTTTTTTTTTTTTTTTTTTTTTTTT PDIG        TELAPSE       80.14 TCPU       80.13
TTTTTTTTTTTTTTTTTTTTTTTTTTTTTT MOQGAD      TELAPSE       80.17 TCPU       80.16
TTTTTTTTTTTTTTTTTTTTTTTTTTTTTT SHELLX      TELAPSE       85.58 TCPU       85.57
TTTTTTTTTTTTTTTTTTTTTTTTTTTTTT MONMO3      TELAPSE       86.39 TCPU       86.38
NUMERICALLY INTEGRATED DENSITY    342.0002379188
TTTTTTTTTTTTTTTTTTTTTTTTTTTTTT NUMDFT      TELAPSE       87.83 TCPU       87.82
CYC   4 ETOT(AU) -8.070267438742E+03 DETOT -1.87E-01 tst  1.44E-03 PX  4.83E-02
TTTTTTTTTTTTTTTTTTTTTTTTTTTTTT FDIK        TELAPSE       92.63 TCPU       92.62
INSULATING STATE - LEVEL SHIFTER    0.60 au
TOP OF VALENCE BANDS -    BAND    171; K    1; EIG -7.1190527E-01 AU
BOTTOM OF VIRTUAL BANDS - BAND    172; K    1; EIG  4.0032143E-02 AU
TTTTTTTTTTTTTTTTTTTTTTTTTTTTTT PDIG        TELAPSE       92.94 TCPU       92.92
TTTTTTTTTTTTTTTTTTTTTTTTTTTTTT MOQGAD      TELAPSE       92.96 TCPU       92.95
TTTTTTTTTTTTTTTTTTTTTTTTTTTTTT SHELLX      TELAPSE       98.37 TCPU       98.36
TTTTTTTTTTTTTTTTTTTTTTTTTTTTTT MONMO3      TELAPSE       99.17 TCPU       99.16
NUMERICALLY INTEGRATED DENSITY    342.0002304859
TTTTTTTTTTTTTTTTTTTTTTTTTTTTTT NUMDFT      TELAPSE      100.62 TCPU      100.61
CYC   5 ETOT(AU) -8.070286804014E+03 DETOT -1.94E-02 tst  5.70E-04 PX  3.28E-02
TTTTTTTTTTTTTTTTTTTTTTTTTTTTTT FDIK        TELAPSE      105.42 TCPU      105.41
INSULATING STATE - LEVEL SHIFTER    0.60 au
TOP OF VALENCE BANDS -    BAND    171; K    1; EIG -7.2493114E-01 AU
BOTTOM OF VIRTUAL BANDS - BAND    172; K    1; EIG  3.8472720E-02 AU
TTTTTTTTTTTTTTTTTTTTTTTTTTTTTT PDIG        TELAPSE      105.73 TCPU      105.72
TTTTTTTTTTTTTTTTTTTTTTTTTTTTTT MOQGAD      TELAPSE      105.75 TCPU      105.74
TTTTTTTTTTTTTTTTTTTTTTTTTTTTTT SHELLX      TELAPSE      111.16 TCPU      111.15
TTTTTTTTTTTTTTTTTTTTTTTTTTTTTT MONMO3      TELAPSE      111.97 TCPU      111.96
NUMERICALLY INTEGRATED DENSITY    342.0002251622
TTTTTTTTTTTTTTTTTTTTTTTTTTTTTT NUMDFT      TELAPSE      113.42 TCPU      113.41
CYC   6 ETOT(AU) -8.070299143102E+03 DETOT -1.23E-02 tst  1.08E-04 PX  1.84E-02
TTTTTTTTTTTTTTTTTTTTTTTTTTTTTT FDIK        TELAPSE      118.70 TCPU      118.69
INSULATING STATE - LEVEL SHIFTER    0.60 au
TOP OF VALENCE BANDS -    BAND    171; K    1; EIG -7.3373051E-01 AU
BOTTOM OF VIRTUAL BANDS - BAND    172; K    1; EIG  3.6457535E-02 AU
TTTTTTTTTTTTTTTTTTTTTTTTTTTTTT PDIG        TELAPSE      119.00 TCPU      118.99
TTTTTTTTTTTTTTTTTTTTTTTTTTTTTT MOQGAD      TELAPSE      119.03 TCPU      119.01
TTTTTTTTTTTTTTTTTTTTTTTTTTTTTT SHELLX      TELAPSE      124.44 TCPU      124.42
TTTTTTTTTTTTTTTTTTTTTTTTTTTTTT MONMO3      TELAPSE      125.24 TCPU      125.23
NUMERICALLY INTEGRATED DENSITY    342.0002222568
TTTTTTTTTTTTTTTTTTTTTTTTTTTTTT NUMDFT      TELAPSE      126.70 TCPU      126.68
CYC   7 ETOT(AU) -8.070310637342E+03 DETOT -1.15E-02 tst  2.26E-05 PX  1.01E-02
TTTTTTTTTTTTTTTTTTTTTTTTTTTTTT FDIK        TELAPSE      131.64 TCPU      131.63
INSULATING STATE - LEVEL SHIFTER    0.60 au
TOP OF VALENCE BANDS -    BAND    171; K    1; EIG -7.3739372E-01 AU
BOTTOM OF VIRTUAL BANDS - BAND    172; K    1; EIG  3.4991671E-02 AU
TTTTTTTTTTTTTTTTTTTTTTTTTTTTTT PDIG        TELAPSE      131.95 TCPU      131.93
TTTTTTTTTTTTTTTTTTTTTTTTTTTTTT MOQGAD      TELAPSE      131.97 TCPU      131.96
TTTTTTTTTTTTTTTTTTTTTTTTTTTTTT SHELLX      TELAPSE      137.38 TCPU      137.37
TTTTTTTTTTTTTTTTTTTTTTTTTTTTTT MONMO3      TELAPSE      138.17 TCPU      138.16
NUMERICALLY INTEGRATED DENSITY    342.0002210448
TTTTTTTTTTTTTTTTTTTTTTTTTTTTTT NUMDFT      TELAPSE      139.61 TCPU      139.60
CYC   8 ETOT(AU) -8.070315478104E+03 DETOT -4.84E-03 tst  1.62E-05 PX  6.75E-03
TTTTTTTTTTTTTTTTTTTTTTTTTTTTTT FDIK        TELAPSE      144.58 TCPU      144.57
INSULATING STATE - LEVEL SHIFTER    0.60 au
TOP OF VALENCE BANDS -    BAND    171; K    1; EIG -7.3762138E-01 AU
BOTTOM OF VIRTUAL BANDS - BAND    172; K    1; EIG  3.4188614E-02 AU
TTTTTTTTTTTTTTTTTTTTTTTTTTTTTT PDIG        TELAPSE      144.88 TCPU      144.87
TTTTTTTTTTTTTTTTTTTTTTTTTTTTTT MOQGAD      TELAPSE      144.91 TCPU      144.89
TTTTTTTTTTTTTTTTTTTTTTTTTTTTTT SHELLX      TELAPSE      150.31 TCPU      150.30
TTTTTTTTTTTTTTTTTTTTTTTTTTTTTT MONMO3      TELAPSE      151.12 TCPU      151.11
NUMERICALLY INTEGRATED DENSITY    342.0002207012
TTTTTTTTTTTTTTTTTTTTTTTTTTTTTT NUMDFT      TELAPSE      152.57 TCPU      152.56
CYC   9 ETOT(AU) -8.070316543527E+03 DETOT -1.07E-03 tst  4.20E-06 PX  3.12E-03
```

```
TTTTTTTTTTTTTTTTTTTTTTTTTTTTTT FDIK        TELAPSE      157.39 TCPU      157.37
INSULATING STATE - LEVEL SHIFTER    0.60 au
TOP OF VALENCE BANDS -    BAND    171; K    1; EIG -7.3651128E-01 AU
BOTTOM OF VIRTUAL BANDS - BAND    172; K    1; EIG  3.3843012E-02 AU
TTTTTTTTTTTTTTTTTTTTTTTTTTTTTT PDIG        TELAPSE      157.69 TCPU      157.68
TTTTTTTTTTTTTTTTTTTTTTTTTTTTTT MOQGAD      TELAPSE      157.71 TCPU      157.70
TTTTTTTTTTTTTTTTTTTTTTTTTTTTTT SHELLX      TELAPSE      163.12 TCPU      163.11
TTTTTTTTTTTTTTTTTTTTTTTTTTTTTT MONMO3      TELAPSE      163.92 TCPU      163.91
NUMERICALLY INTEGRATED DENSITY    342.0002206603
TTTTTTTTTTTTTTTTTTTTTTTTTTTTTT NUMDFT      TELAPSE      165.38 TCPU      165.37
CYC  10 ETOT(AU) -8.070316716856E+03 DETOT -1.73E-04 tst  1.90E-06 PX  1.24E-03
TTTTTTTTTTTTTTTTTTTTTTTTTTTTTT FDIK        TELAPSE      170.46 TCPU      170.44
INSULATING STATE - LEVEL SHIFTER    0.60 au
TOP OF VALENCE BANDS -    BAND    171; K    1; EIG -7.3541629E-01 AU
BOTTOM OF VIRTUAL BANDS - BAND    172; K    1; EIG  3.3723262E-02 AU
TTTTTTTTTTTTTTTTTTTTTTTTTTTTTT PDIG        TELAPSE      170.76 TCPU      170.75
TTTTTTTTTTTTTTTTTTTTTTTTTTTTTT MOQGAD      TELAPSE      170.78 TCPU      170.77
TTTTTTTTTTTTTTTTTTTTTTTTTTTTTT SHELLX      TELAPSE      176.19 TCPU      176.18
TTTTTTTTTTTTTTTTTTTTTTTTTTTTTT MONMO3      TELAPSE      177.00 TCPU      176.99
NUMERICALLY INTEGRATED DENSITY    342.0002206307
TTTTTTTTTTTTTTTTTTTTTTTTTTTTTT NUMDFT      TELAPSE      178.45 TCPU      178.43
CYC  11 ETOT(AU) -8.070316810887E+03 DETOT -9.40E-05 tst  2.32E-06 PX  1.45E-03
TTTTTTTTTTTTTTTTTTTTTTTTTTTTTT FDIK        TELAPSE      183.33 TCPU      183.32
INSULATING STATE - LEVEL SHIFTER    0.60 au
TOP OF VALENCE BANDS -    BAND    171; K    1; EIG -7.3482168E-01 AU
BOTTOM OF VIRTUAL BANDS - BAND    172; K    1; EIG  3.3677766E-02 AU
TTTTTTTTTTTTTTTTTTTTTTTTTTTTTT PDIG        TELAPSE      183.63 TCPU      183.62
TTTTTTTTTTTTTTTTTTTTTTTTTTTTTT MOQGAD      TELAPSE      183.66 TCPU      183.65
TTTTTTTTTTTTTTTTTTTTTTTTTTTTTT SHELLX      TELAPSE      189.07 TCPU      189.06
TTTTTTTTTTTTTTTTTTTTTTTTTTTTTT MONMO3      TELAPSE      189.88 TCPU      189.87
NUMERICALLY INTEGRATED DENSITY    342.0002205360
TTTTTTTTTTTTTTTTTTTTTTTTTTTTTT NUMDFT      TELAPSE      191.33 TCPU      191.32
CYC  12 ETOT(AU) -8.070316868770E+03 DETOT -5.79E-05 tst  1.90E-06 PX  1.79E-03
TTTTTTTTTTTTTTTTTTTTTTTTTTTTTT FDIK        TELAPSE      196.14 TCPU      196.13
INSULATING STATE - LEVEL SHIFTER    0.60 au
TOP OF VALENCE BANDS -    BAND    171; K    1; EIG -7.3468683E-01 AU
BOTTOM OF VIRTUAL BANDS - BAND    172; K    1; EIG  3.3636864E-02 AU
TTTTTTTTTTTTTTTTTTTTTTTTTTTTTT PDIG        TELAPSE      196.44 TCPU      196.43
TTTTTTTTTTTTTTTTTTTTTTTTTTTTTT MOQGAD      TELAPSE      196.47 TCPU      196.46
TTTTTTTTTTTTTTTTTTTTTTTTTTTTTT SHELLX      TELAPSE      201.88 TCPU      201.87
TTTTTTTTTTTTTTTTTTTTTTTTTTTTTT MONMO3      TELAPSE      202.70 TCPU      202.69
NUMERICALLY INTEGRATED DENSITY    342.0002203934
TTTTTTTTTTTTTTTTTTTTTTTTTTTTTT NUMDFT      TELAPSE      204.15 TCPU      204.14
CYC  13 ETOT(AU) -8.070316891118E+03 DETOT -2.23E-05 tst  1.14E-06 PX  1.56E-03
TTTTTTTTTTTTTTTTTTTTTTTTTTTTTT FDIK        TELAPSE      209.00 TCPU      208.99
INSULATING STATE - LEVEL SHIFTER    0.60 au
TOP OF VALENCE BANDS -    BAND    171; K    1; EIG -7.3479854E-01 AU
BOTTOM OF VIRTUAL BANDS - BAND    172; K    1; EIG  3.3583661E-02 AU
TTTTTTTTTTTTTTTTTTTTTTTTTTTTTT PDIG        TELAPSE      209.31 TCPU      209.29
TTTTTTTTTTTTTTTTTTTTTTTTTTTTTT MOQGAD      TELAPSE      209.33 TCPU      209.32
TTTTTTTTTTTTTTTTTTTTTTTTTTTTTT SHELLX      TELAPSE      214.74 TCPU      214.73
TTTTTTTTTTTTTTTTTTTTTTTTTTTTTT MONMO3      TELAPSE      215.55 TCPU      215.53
NUMERICALLY INTEGRATED DENSITY    342.0002202488
TTTTTTTTTTTTTTTTTTTTTTTTTTTTTT NUMDFT      TELAPSE      216.99 TCPU      216.98
CYC  14 ETOT(AU) -8.070316898820E+03 DETOT -7.70E-06 tst  5.44E-07 PX  1.13E-03
TTTTTTTTTTTTTTTTTTTTTTTTTTTTTT FDIK        TELAPSE      221.95 TCPU      221.93
INSULATING STATE - LEVEL SHIFTER    0.60 au
TOP OF VALENCE BANDS -    BAND    171; K    1; EIG -7.3497285E-01 AU
BOTTOM OF VIRTUAL BANDS - BAND    172; K    1; EIG  3.3523251E-02 AU
TTTTTTTTTTTTTTTTTTTTTTTTTTTTTT PDIG        TELAPSE      222.25 TCPU      222.24
TTTTTTTTTTTTTTTTTTTTTTTTTTTTTT MOQGAD      TELAPSE      222.28 TCPU      222.26
TTTTTTTTTTTTTTTTTTTTTTTTTTTTTT SHELLX      TELAPSE      227.69 TCPU      227.67
TTTTTTTTTTTTTTTTTTTTTTTTTTTTTT MONMO3      TELAPSE      228.51 TCPU      228.49
NUMERICALLY INTEGRATED DENSITY    342.0002201318
TTTTTTTTTTTTTTTTTTTTTTTTTTTTTT NUMDFT      TELAPSE      229.96 TCPU      229.95
CYC  15 ETOT(AU) -8.070316902628E+03 DETOT -3.81E-06 tst  2.28E-07 PX  7.17E-04
TTTTTTTTTTTTTTTTTTTTTTTTTTTTTT FDIK        TELAPSE      235.00 TCPU      234.98
INSULATING STATE - LEVEL SHIFTER    0.60 au
TOP OF VALENCE BANDS -    BAND    171; K    1; EIG -7.3511436E-01 AU
BOTTOM OF VIRTUAL BANDS - BAND    172; K    1; EIG  3.3464727E-02 AU
TTTTTTTTTTTTTTTTTTTTTTTTTTTTTT PDIG        TELAPSE      235.30 TCPU      235.29
TTTTTTTTTTTTTTTTTTTTTTTTTTTTTT MOQGAD      TELAPSE      235.33 TCPU      235.31
TTTTTTTTTTTTTTTTTTTTTTTTTTTTTT SHELLX      TELAPSE      240.74 TCPU      240.72
TTTTTTTTTTTTTTTTTTTTTTTTTTTTTT MONMO3      TELAPSE      241.55 TCPU      241.54
NUMERICALLY INTEGRATED DENSITY    342.0002200520
TTTTTTTTTTTTTTTTTTTTTTTTTTTTTT NUMDFT      TELAPSE      243.00 TCPU      242.98
CYC  16 ETOT(AU) -8.070316904595E+03 DETOT -1.97E-06 tst  9.41E-08 PX  4.28E-04
TTTTTTTTTTTTTTTTTTTTTTTTTTTTTT FDIK        TELAPSE      248.00 TCPU      247.99
INSULATING STATE - LEVEL SHIFTER    0.60 au
```

```
TOP OF VALENCE BANDS -    BAND    171; K    1; EIG -7.3519999E-01 AU
BOTTOM OF VIRTUAL BANDS - BAND    172; K    1; EIG  3.3413987E-02 AU
TTTTTTTTTTTTTTTTTTTTTTTTTTTTTT PDIG        TELAPSE      248.31 TCPU      248.29
TTTTTTTTTTTTTTTTTTTTTTTTTTTTTT MOQGAD      TELAPSE      248.33 TCPU      248.32
TTTTTTTTTTTTTTTTTTTTTTTTTTTTTT SHELLX      TELAPSE      253.74 TCPU      253.73
TTTTTTTTTTTTTTTTTTTTTTTTTTTTTT MONMO3      TELAPSE      254.55 TCPU      254.53
NUMERICALLY INTEGRATED DENSITY    342.0002200038
TTTTTTTTTTTTTTTTTTTTTTTTTTTTTT NUMDFT      TELAPSE      255.99 TCPU      255.98
CYC  17 ETOT(AU) -8.070316905342E+03 DETOT -7.48E-07 tst  4.99E-08 PX  2.59E-04
TTTTTTTTTTTTTTTTTTTTTTTTTTTTTT FDIK        TELAPSE      260.95 TCPU      260.93
INSULATING STATE - LEVEL SHIFTER    0.60 au
TOP OF VALENCE BANDS -    BAND    171; K    1; EIG -7.3524257E-01 AU
BOTTOM OF VIRTUAL BANDS - BAND    172; K    1; EIG  3.3372943E-02 AU
TTTTTTTTTTTTTTTTTTTTTTTTTTTTTT PDIG        TELAPSE      261.25 TCPU      261.24
TTTTTTTTTTTTTTTTTTTTTTTTTTTTTT MOQGAD      TELAPSE      261.28 TCPU      261.26
TTTTTTTTTTTTTTTTTTTTTTTTTTTTTT SHELLX      TELAPSE      266.69 TCPU      266.67
TTTTTTTTTTTTTTTTTTTTTTTTTTTTTT MONMO3      TELAPSE      267.51 TCPU      267.50
NUMERICALLY INTEGRATED DENSITY    342.0002199769
TTTTTTTTTTTTTTTTTTTTTTTTTTTTTT NUMDFT      TELAPSE      268.95 TCPU      268.94
CYC  18 ETOT(AU) -8.070316905529E+03 DETOT -1.87E-07 tst  2.87E-08 PX  1.72E-04
TTTTTTTTTTTTTTTTTTTTTTTTTTTTTT FDIK        TELAPSE      273.86 TCPU      273.85
INSULATING STATE - LEVEL SHIFTER    0.60 au
TOP OF VALENCE BANDS -    BAND    171; K    1; EIG -7.3526259E-01 AU
BOTTOM OF VIRTUAL BANDS - BAND    172; K    1; EIG  3.3340935E-02 AU
TTTTTTTTTTTTTTTTTTTTTTTTTTTTTT PDIG        TELAPSE      274.17 TCPU      274.15
TTTTTTTTTTTTTTTTTTTTTTTTTTTTTT MOQGAD      TELAPSE      274.19 TCPU      274.17
TTTTTTTTTTTTTTTTTTTTTTTTTTTTTT SHELLX      TELAPSE      279.60 TCPU      279.58
TTTTTTTTTTTTTTTTTTTTTTTTTTTTTT MONMO3      TELAPSE      280.41 TCPU      280.39
NUMERICALLY INTEGRATED DENSITY    342.0002199617
TTTTTTTTTTTTTTTTTTTTTTTTTTTTTT NUMDFT      TELAPSE      281.85 TCPU      281.83
CYC  19 ETOT(AU) -8.070316905560E+03 DETOT -3.04E-08 tst  1.74E-08 PX  1.27E-04
TTTTTTTTTTTTTTTTTTTTTTTTTTTTTT FDIK        TELAPSE      286.77 TCPU      286.75
INSULATING STATE - LEVEL SHIFTER    0.60 au
TOP OF VALENCE BANDS -    BAND    171; K    1; EIG -7.3527485E-01 AU
BOTTOM OF VIRTUAL BANDS - BAND    172; K    1; EIG  3.3316289E-02 AU
TTTTTTTTTTTTTTTTTTTTTTTTTTTTTT PDIG        TELAPSE      287.07 TCPU      287.05
TTTTTTTTTTTTTTTTTTTTTTTTTTTTTT MOQGAD      TELAPSE      287.10 TCPU      287.08
TTTTTTTTTTTTTTTTTTTTTTTTTTTTTT SHELLX      TELAPSE      292.50 TCPU      292.49
TTTTTTTTTTTTTTTTTTTTTTTTTTTTTT MONMO3      TELAPSE      293.31 TCPU      293.29
NUMERICALLY INTEGRATED DENSITY    342.0002199520
TTTTTTTTTTTTTTTTTTTTTTTTTTTTTT NUMDFT      TELAPSE      294.76 TCPU      294.74
CYC  20 ETOT(AU) -8.070316905560E+03 DETOT  1.09E-11 tst  1.06E-08 PX  1.01E-04
TTTTTTTTTTTTTTTTTTTTTTTTTTTTTT FDIK        TELAPSE      299.66 TCPU      299.65
INSULATING STATE - LEVEL SHIFTER    0.60 au
TOP OF VALENCE BANDS -    BAND    171; K    1; EIG -7.3528615E-01 AU
BOTTOM OF VIRTUAL BANDS - BAND    172; K    1; EIG  3.3297288E-02 AU
TTTTTTTTTTTTTTTTTTTTTTTTTTTTTT PDIG        TELAPSE      299.96 TCPU      299.95
TTTTTTTTTTTTTTTTTTTTTTTTTTTTTT MOQGAD      TELAPSE      299.99 TCPU      299.97
TTTTTTTTTTTTTTTTTTTTTTTTTTTTTT SHELLX      TELAPSE      305.40 TCPU      305.38
::: PSEUDO TOTAL   ENERGY                        -7.5790603503601E+03
::: VIRIAL COEFFICIENT                            1.0312847845847E+00
TTTTTTTTTTTTTTTTTTTTTTTTTTTTTT MONMO3      TELAPSE      306.21 TCPU      306.20
NUMERICALLY INTEGRATED DENSITY    342.0002199448
TTTTTTTTTTTTTTTTTTTTTTTTTTTTTT NUMDFT      TELAPSE      307.66 TCPU      307.64
CYC  21 ETOT(AU) -8.070316905547E+03 DETOT  1.25E-08 tst  6.42E-09 PX  1.01E-04

== SCF ENDED - CONVERGENCE ON ENERGY      E(AU) -8.0703169055473E+03 CYCLES  21


ENERGY EXPRESSION=HARTREE+FOCK EXCH*0.00000+(LDA    EXCH)*1.00000+VWN    CORR


TOTAL ENERGY(DFT)(AU)( 21) -8.0703169055473E+03 DE 1.2E-08 tester 6.4E-09
TTTTTTTTTTTTTTTTTTTTTTTTTTTTTT EDFT        TELAPSE      307.66 TCPU      307.65

*******************************************************************************
*                              FORCE CALCULATION                              *
*******************************************************************************

TTTTTTTTTTTTTTTTTTTTTTTTTTTTTT NUMDFG      TELAPSE      377.72 TCPU      377.70
INFORMATION **** EXCPOG **** EXCH. BIPO BUFFER LENGTH (WORDS) =          0
WARNING **** GENPOG **** COULOMB BIPO BUFFER TOO SMALL - TO AVOID I/O SET BIPOSIZE =     1066200
INFORMATION **** GENPOG **** BIPO BUFFER LENGTH (WORDS) =     1000000
TTTTTTTTTTTTTTTTTTTTTTTTTTTTTT SHELXG      TELAPSE      407.24 TCPU      407.22

CARTESIAN FORCES IN HARTREE/BOHR (ANALYTICAL)
  ATOM                     X                   Y                   Z
  1  20           6.757424268145E-06  6.877846226572E-15  4.689040031569E-05
  2  20          -1.155265695343E-04 -1.348895275006E-04  2.366446816671E-04
```

```
   3  20           -1.155265695538E-04  1.348895275157E-04  2.366446816728E-04
   4  20            1.902628409280E-04 -1.166766102667E-05  1.491934640701E-04
   5  20            1.902628409565E-04  1.166766102489E-05  1.491934640763E-04
   6  20            1.423772565534E-04 -4.964943983271E-16 -3.210263056479E-04
   7  20            1.060426438801E-04 -1.792991019995E-14  3.002514813488E-04
   8  20           -2.458029705865E-04  2.280905090668E-04  3.805548322804E-04
   9  20           -2.458029705927E-04 -2.280905090704E-04  3.805548322866E-04
  10  14           -1.533049770104E-04  7.871283816121E-15 -4.663603227417E-05
  11  14           -4.529288842370E-06 -2.665470509265E-14 -2.464482007112E-04
  12  14           -4.792563450264E-04  1.416497741050E-14 -4.180612757070E-04
  13   8            1.740129212993E-04  1.707527554440E-15 -2.046565451650E-04
  14   8            5.416301223848E-05  3.274278333132E-05  5.383060647857E-05
  15   8            5.416301225464E-05 -3.274278330423E-05  5.383060645636E-05
  16   8            9.562466737156E-05 -1.719728091466E-14  2.291747031602E-04
  17   8           -6.351628046752E-05 -1.032267892018E-14 -1.974589234970E-04
  18   8            3.433679657658E-04 -6.780164692410E-15  4.911468556479E-05
  19   8            7.720290633439E-05 -3.341978916126E-15 -8.377824640693E-05
  20   8           -3.486954638610E-05  1.975056911464E-04  8.275235439315E-05
  21   8           -3.486954638476E-05 -1.975056911528E-04  8.275235439537E-05
  22   8           -6.259004613329E-05  3.365324183762E-15 -1.798485564541E-05
  23   8           -3.154705907638E-05 -1.446816156453E-15 -3.355557600051E-04
  24   8           -1.093464623687E-04  8.337214584904E-15 -2.676994642217E-04
  25   8            7.399341277625E-05  4.262280490855E-15 -1.974570036349E-04
  26   8            9.412886286775E-05 -1.047497637008E-05 -4.731026794524E-05
  27   8            9.412886286508E-05  1.047497637807E-05 -4.731026795124E-05

RESULTANT FORCE    -1.603828181374E-12  8.301668272086E-16 -6.465938895417E-13

SYMMETRY ALLOWED FORCES (ANALYTICAL) (DIRECTION, FORCE)

    1  6.8861487E-06    2  4.7783631E-05    3 -1.6966794E-04    4 -1.9076260E-04
    5  3.5098619E-04    6  2.6723239E-04    7 -1.6500564E-05    8  2.5238820E-04
    9  1.5299624E-04   10 -2.9049773E-04   11  1.2323192E-04   12  3.3194370E-04
   13 -3.3626061E-04   14  3.2256869E-04   15  6.2676154E-04   16 -1.6272047E-04
   17  4.1128388E-05   18 -1.9236650E-05   19 -1.6233843E-04   20 -5.1077497E-04
   21 -3.5006367E-04   22  1.3015652E-04   23 -1.5333893E-04   24  2.2721764E-05
   25  4.6305288E-05   26  1.5239552E-04   27  6.1430984E-05   28  3.0471050E-04
   29 -9.9614163E-05   30 -1.1366349E-04   31  3.1876605E-04   32  1.3425169E-04
   33  7.4080604E-05   34  9.1437085E-06   35 -5.4702178E-05   36  2.7931523E-04
   37  2.8778070E-04   38 -7.9293402E-05   39  1.4704680E-04   40 -6.2407072E-05
   41 -1.7496025E-04   42 -1.7040220E-04   43 -1.4751901E-04   44 -1.6440526E-05
   45 -1.2259430E-04   46 -1.4813854E-05
TTTTTTTTTTTTTTTTTTTTTTTTTTTTT TOTGRA      TELAPSE      409.68 TCPU      409.67

HHHHHHHHHHHHHHHHHHHHHHHHHHHHHHHHHHHHHHHHHHHHHHHHHHHHHHHHHHHHHHHHHHHHHHHHHHHHHHHH
                  FORCE CONSTANT MATRIX - NUMERICAL ESTIMATE
HHHHHHHHHHHHHHHHHHHHHHHHHHHHHHHHHHHHHHHHHHHHHHHHHHHHHHHHHHHHHHHHHHHHHHHHHHHHHHHH
MAX ABS(DGRAD): MAXIMUM ABSOLUTE GRADIENT DIFFERENCE WITH RESPECT TO
                THE CENTRAL POINT
DE:             ENERGY DIFFERENCE WITH RESPECT TO THE CENTRAL POINT
                (DE IS EXPECTED TO BE POSITIVE FOR ALL DISPLACEMENTS)

  ATOM      MAX ABS(DGRAD)       TOTAL ENERGY (AU)  N.CYC      DE       SYM
   CENTRAL POINT             -8.070316905547E+03    21     0.0000E+00    2
   1 CA                      GENERATED BY TRANSLATIONAL INVARIANCE
   2 CA DX   4.9533E-04      -8.070316903826E+03    11     1.7211E-06    1
   2 CA DY   4.8677E-04      -8.070316902969E+03    11     2.5784E-06    1
   2 CA DZ   3.4926E-04      -8.070316905851E+03    14    -3.0421E-07    1
   4 CA DX   4.6142E-04      -8.070316905578E+03    15    -3.0303E-08    1
   4 CA DY   4.7327E-04      -8.070316904679E+03    17     8.6839E-07    1
   4 CA DZ   4.4511E-04      -8.070316905024E+03    15     5.2309E-07    1
   6 CA DX   4.8127E-04      -8.070316905908E+03    16    -3.6098E-07    2
   6 CA DY   5.8378E-04      -8.070316903682E+03    16     1.8650E-06    1
   6 CA DZ   6.6626E-04      -8.070316900498E+03    14     5.0489E-06    2
   7 CA DX   5.1357E-04      -8.070316902474E+03    16     3.0729E-06    2
   7 CA DY   5.3704E-04      -8.070316903829E+03    11     1.7183E-06    1
   7 CA DZ   5.3390E-04      -8.070316905597E+03    16    -4.9796E-08    2
   8 CA DX   5.6616E-04      -8.070316902056E+03    17     3.4911E-06    1
   8 CA DY   5.3335E-04      -8.070316906367E+03     6    -8.1980E-07    1
   8 CA DZ   4.3511E-04      -8.070316905490E+03    17     5.7005E-08    1
  10 SI DX   2.5775E-03      -8.070316896960E+03    20     8.5874E-06    2
  10 SI DY   2.6603E-03      -8.070316897814E+03    12     7.7335E-06    1
  10 SI DZ   2.7626E-03      -8.070316898623E+03    18     6.9244E-06    2
  11 SI DX   2.4798E-03      -8.070316899057E+03    17     6.4898E-06    2
  11 SI DY   2.5260E-03      -8.070316898174E+03    12     7.3738E-06    1
  11 SI DZ   2.6027E-03      -8.070316895084E+03    19     1.0463E-05    2
  12 SI DX   2.8919E-03      -8.070316894022E+03    15     1.1525E-05    2
  12 SI DY   2.6233E-03      -8.070316897905E+03    12     7.6428E-06    1
  12 SI DZ   2.7190E-03      -8.070316896109E+03    17     9.4386E-06    2
```

```
  13 O  DX   4.5826E-04      -8.070316905138E+03    18     4.0944E-07    2
  13 O  DY   2.7064E-04      -8.070316904601E+03     8     9.4600E-07    1
  13 O  DZ   2.0966E-03      -8.070316896971E+03    14     8.5765E-06    2
  14 O  DX   7.0178E-04      -8.070316903390E+03    18     2.1576E-06    1
  14 O  DY   1.4914E-03      -8.070316901079E+03    19     4.4686E-06    1
  14 O  DZ   5.5767E-04      -8.070316904664E+03    17     8.8284E-07    1
  16 O  DX   1.8290E-03      -8.070316901094E+03    17     4.4530E-06    2
  16 O  DY   4.2920E-04      -8.070316904127E+03    15     1.4207E-06    1
  16 O  DZ   3.6763E-04      -8.070316904500E+03    14     1.0474E-06    2
  17 O  DX   4.2432E-04      -8.070316904336E+03    16     1.2108E-06    2
  17 O  DY   3.6624E-04      -8.070316904307E+03    15     1.2405E-06    1
  17 O  DZ   3.7288E-04      -8.070316901925E+03    18     3.6224E-06    2
  18 O  DX   3.6265E-04      -8.070316905854E+03    10    -3.0721E-07    2
  18 O  DY   4.0592E-04      -8.070316904257E+03    11     1.2899E-06    1
  18 O  DZ   1.7205E-03      -8.070316902536E+03    17     3.0113E-06    2
  19 O  DX   1.5389E-03      -8.070316901584E+03    18     3.9629E-06    2
  19 O  DY   4.1461E-04      -8.070316904170E+03    11     1.3773E-06    1
  19 O  DZ   8.6372E-04      -8.070316901965E+03    14     3.5821E-06    2
  20 O  DX   9.3138E-04      -8.070316902351E+03    16     3.1965E-06    1
  20 O  DY   1.5388E-03      -8.070316901563E+03    15     3.9843E-06    1
  20 O  DZ   4.0989E-04      -8.070316904664E+03    18     8.8351E-07    1
  22 O  DX   4.4343E-04      -8.070316902802E+03    16     2.7449E-06    2
  22 O  DY   3.5988E-04      -8.070316904331E+03    13     1.2160E-06    1
  22 O  DZ   5.2961E-04      -8.070316903430E+03    14     2.1175E-06    2
  23 O  DX   6.3495E-04      -8.070316903770E+03    19     1.7770E-06    2
  23 O  DY   7.0988E-04      -8.070316903335E+03    11     2.2126E-06    1
  23 O  DZ   5.0273E-04      -8.070316903343E+03    16     2.2039E-06    2
  24 O  DX   6.8302E-04      -8.070316902746E+03    16     2.8016E-06    2
  24 O  DY   4.1232E-04      -8.070316904178E+03    11     1.3691E-06    1
  24 O  DZ   1.9200E-03      -8.070316899229E+03    17     6.3187E-06    2
  25 O  DX   1.4325E-03      -8.070316901234E+03    16     4.3136E-06    2
  25 O  DY   2.4577E-04      -8.070316904658E+03     7     8.8975E-07    1
  25 O  DZ   1.3495E-03      -8.070316900945E+03    18     4.6022E-06    2
  26 O  DX   9.2340E-04      -8.070316904370E+03    16     1.1774E-06    1
  26 O  DY   1.4855E-03      -8.070316901645E+03    20     3.9023E-06    1
  26 O  DZ   3.5501E-04      -8.070316903771E+03    16     1.7764E-06    1

GCALCO - MAX INDICES DIRECT LATTICE VECTOR    16    16    12
NO.OF VECTORS CREATED 6999 STARS 1811 RMAX   159.70567 BOHR

CAPPA:IS1  4;IS2  4;IS3  4; K PTS MONK NET   24; SYMMOPS: K SPACE  4;G SPACE  2

TTTTTTTTTTTTTTTTTTTTTTTTTTTTTT gordsh1     TELAPSE    25568.18 TCPU    25565.29

DIMENSIONS  P(G)=  345586 F(G)=  173156 P(G),F(G) (IRR)  103599
MAX G-VECTOR INDEX FOR 1- AND 2-ELECTRON INTEGRALS  33

TTTTTTTTTTTTTTTTTTTTTTTTTTTTTT INPUT       TELAPSE    25568.18 TCPU    25565.29

SYMMETRY ALLOWED INTERNAL DEGREE(S) OF FREEDOM:  46
TTTTTTTTTTTTTTTTTTTTTTTTTTTTTT SYMM        TELAPSE    25568.21 TCPU    25565.32
TTTTTTTTTTTTTTTTTTTTTTTTTTTTTT INT_SCREEN  TELAPSE    25568.22 TCPU    25565.33

+++ SYMMETRY ADAPTION OF VIBRATIONAL MODES +++

SYMMETRY INFORMATION:
K-LITTLE GROUP: CLASS TABLE, CHARACTER TABLE.
IRREP-(DIMENSION, NO. IRREDUCIBLE SETS)
[WARNINGS: (1) ONLY ACTIVE IRREPS ARE GENERATED AND LISTED.
           (2) ONLY RELEVANT CLASSES ARE CONSIDERED IN THE CHARACTER TABLE
           (3) SYMBOLS MAY NOT FULLY COINCIDE WITH THOSE FROM TEXT BOOKS.
IN CLASSES WRITTEN AS CX.Y OR SX.Y, X.Y IS A DECIMAL NUMBER THAT
APPROACHES THE OPERATION FRACTIONAL ORDER.]

(P, D, RP, RD, STAND FOR PAIRING, DOUBLING, REAL PAIRING AND REAL DOUBLING
OF THE IRREPS (SEE MANUAL))

K[   1] (  0  0  0)

CLASS | GROUP OPERATORS (SEE SYMMOPS KEYWORD)
-----------------------------------------------------------------------
SGH   |  2;

IRREP/CLA   E    SGH
--------------------
   A'  |  1.00  1.00
   B"  |  1.00 -1.00

A' -(1,  48); B" -(1,  33);
```

HHHHHHHHHHHHHHHHHHHHHHHHHHHHHHHHHHHHHHHHHHHHHHHHHHHHHHHHHHHHHHHHHHHHHHHHHHHHHHHH

EIGENVALUES (EIGV) OF THE MASS WEIGHTED HESSIAN MATRIX AND HARMONIC FREQUENCIES. IRREP LABELS REFER TO SYMMETRY REPRESENTATION ANALYSIS; A AND I INDICATE WHETHER THE MODE IS ACTIVE OR INACTIVE, RESPECTIVELY, FOR IR AND RAMAN; IR INTENSITIES IN BRACKETS.

CONVERSION FACTORS FOR FREQUENCIES:

1 CM**(-1) = 0.2194746E+06 HARTREE

1 THZ = 0.2997925E-01 CM**(-1)

HHHHHHHHHHHHHHHHHHHHHHHHHHHHHHHHHHHHHHHHHHHHHHHHHHHHHHHHHHHHHHHHHHHHHHHHHHHHHHHH

MODES	EIGV (HARTREE**2)	FREQUENCIES (CM**-1)	(THZ)	IRREP	IR	INTENS (KM/MOL)	RAMAN
1- 1	-0.1386E-06	-81.7207	-2.4499	(B")	A	(0.00)	A
2- 2	-0.5307E-07	-50.5605	-1.5158	(A')	A	(0.00)	A
3- 3	-0.1895E-10	-0.9555	-0.0286	(B")	A	(0.00)	A
4- 4	0.4323E-08	14.4311	0.4326	(A')	A	(0.00)	A
5- 5	0.5373E-07	50.8737	1.5252	(A')	A	(0.00)	A
6- 6	0.5384E-07	50.9280	1.5268	(B")	A	(0.00)	A
7- 7	0.1378E-06	81.4698	2.4424	(B")	A	(0.00)	A
8- 8	0.2018E-06	98.5856	2.9555	(A')	A	(0.00)	A
9- 9	0.2844E-06	117.0374	3.5087	(A')	A	(0.00)	A
10- 10	0.3186E-06	123.8884	3.7141	(B")	A	(0.00)	A
11- 11	0.3430E-06	128.5314	3.8533	(B")	A	(0.00)	A
12- 12	0.3935E-06	137.6781	4.1275	(A')	A	(0.00)	A
13- 13	0.4223E-06	142.6170	4.2756	(B")	A	(0.00)	A
14- 14	0.4965E-06	154.6408	4.6360	(A')	A	(0.00)	A
15- 15	0.5117E-06	156.9981	4.7067	(A')	A	(0.00)	A
16- 16	0.5289E-06	159.6205	4.7853	(B")	A	(0.00)	A
17- 17	0.5896E-06	168.5311	5.0524	(B")	A	(0.00)	A
18- 18	0.6254E-06	173.5707	5.2035	(A')	A	(0.00)	A
19- 19	0.6515E-06	177.1471	5.3107	(B")	A	(0.00)	A
20- 20	0.6735E-06	180.1129	5.3996	(A')	A	(0.00)	A
21- 21	0.7100E-06	184.9289	5.5440	(B")	A	(0.00)	A
22- 22	0.8123E-06	197.8092	5.9302	(A')	A	(0.00)	A
23- 23	0.8126E-06	197.8459	5.9313	(B")	A	(0.00)	A
24- 24	0.8261E-06	199.4834	5.9804	(B")	A	(0.00)	A
25- 25	0.9225E-06	210.7967	6.3195	(A')	A	(0.00)	A
26- 26	0.9508E-06	214.0121	6.4159	(A')	A	(0.00)	A
27- 27	0.9807E-06	217.3500	6.5160	(B")	A	(0.00)	A
28- 28	0.1013E-05	220.8424	6.6207	(A')	A	(0.00)	A
29- 29	0.1065E-05	226.5278	6.7911	(A')	A	(0.00)	A
30- 30	0.1249E-05	245.2820	7.3534	(B")	A	(0.00)	A
31- 31	0.1256E-05	245.9659	7.3739	(A')	A	(0.00)	A
32- 32	0.1352E-05	255.1534	7.6493	(B")	A	(0.00)	A
33- 33	0.1383E-05	258.0844	7.7372	(A')	A	(0.00)	A
34- 34	0.1407E-05	260.3622	7.8055	(A')	A	(0.00)	A
35- 35	0.1504E-05	269.1398	8.0686	(A')	A	(0.00)	A
36- 36	0.1511E-05	269.8237	8.0891	(A')	A	(0.00)	A
37- 37	0.1693E-05	285.5356	8.5601	(B")	A	(0.00)	A
38- 38	0.1763E-05	291.4489	8.7374	(A')	A	(0.00)	A
39- 39	0.1802E-05	294.6063	8.8321	(B")	A	(0.00)	A
40- 40	0.1810E-05	295.2330	8.8509	(B")	A	(0.00)	A
41- 41	0.1890E-05	301.7101	9.0450	(A')	A	(0.00)	A
42- 42	0.2032E-05	312.8421	9.3788	(B")	A	(0.00)	A
43- 43	0.2068E-05	315.5866	9.4610	(B")	A	(0.00)	A
44- 44	0.2093E-05	317.5496	9.5199	(B")	A	(0.00)	A
45- 45	0.2102E-05	318.1901	9.5391	(A')	A	(0.00)	A
46- 46	0.2214E-05	326.5861	9.7908	(A')	A	(0.00)	A
47- 47	0.2289E-05	332.0423	9.9544	(A')	A	(0.00)	A
48- 48	0.2405E-05	340.3355	10.2030	(B")	A	(0.00)	A
49- 49	0.2489E-05	346.2879	10.3815	(B")	A	(0.00)	A
50- 50	0.2519E-05	348.3371	10.4429	(A')	A	(0.00)	A
51- 51	0.2630E-05	355.9257	10.6704	(A')	A	(0.00)	A
52- 52	0.2837E-05	369.6787	11.0827	(A')	A	(0.00)	A
53- 53	0.3014E-05	381.0401	11.4233	(B")	A	(0.00)	A
54- 54	0.3140E-05	388.9334	11.6599	(A')	A	(0.00)	A
55- 55	0.3231E-05	394.5082	11.8271	(A')	A	(0.00)	A
56- 56	0.3540E-05	412.9269	12.3792	(B")	A	(0.00)	A
57- 57	0.3968E-05	437.1764	13.1062	(A')	A	(0.00)	A
58- 58	0.4178E-05	448.6084	13.4489	(A')	A	(0.00)	A
59- 59	0.4279E-05	454.0122	13.6109	(A')	A	(0.00)	A
60- 60	0.4342E-05	457.3477	13.7109	(B")	A	(0.00)	A
61- 61	0.5074E-05	494.3583	14.8205	(A')	A	(0.00)	A
62- 62	0.5132E-05	497.2171	14.9062	(B")	A	(0.00)	A
63- 63	0.5280E-05	504.3231	15.1192	(A')	A	(0.00)	A

64-	64	0.5368E-05	508.4763	15.2437	(A')	A (0.00)	A
65-	65	0.5779E-05	527.6282	15.8179	(B")	A (0.00)	A
66-	66	0.5827E-05	529.8052	15.8832	(A')	A (0.00)	A
67-	67	0.5869E-05	531.6882	15.9396	(A')	A (0.00)	A
68-	68	0.5921E-05	534.0607	16.0107	(B")	A (0.00)	A
69-	69	0.6180E-05	545.5958	16.3566	(A')	A (0.00)	A
70-	70	0.1399E-04	821.0347	24.6140	(A')	A (0.00)	A
71-	71	0.1521E-04	855.8369	25.6573	(A')	A (0.00)	A
72-	72	0.1552E-04	864.7241	25.9238	(A')	A (0.00)	A
73-	73	0.1580E-04	872.2873	26.1505	(A')	A (0.00)	A
74-	74	0.1652E-04	892.1374	26.7456	(B")	A (0.00)	A
75-	75	0.1707E-04	906.8861	27.1878	(A')	A (0.00)	A
76-	76	0.1718E-04	909.6901	27.2718	(A')	A (0.00)	A
77-	77	0.1739E-04	915.1337	27.4350	(B")	A (0.00)	A
78-	78	0.1792E-04	929.0265	27.8515	(A')	A (0.00)	A
79-	79	0.1797E-04	930.2760	27.8890	(B")	A (0.00)	A
80-	80	0.1980E-04	976.6831	29.2802	(A')	A (0.00)	A
81-	81	0.2023E-04	987.0618	29.5914	(A')	A (0.00)	A

NORMAL MODES NORMALIZED TO CLASSICAL AMPLITUDES

FREQ(CM**-1)		-81.72	-50.56	-0.96	14.43	50.87	50.93
AT. 1 CA	X	0.0000	0.0407	0.0000	-0.0850	0.0351	0.0000
	Y	0.0082	0.0000	0.4313	0.0000	0.0000	0.0205
	Z	0.0000	0.0482	0.0000	0.0567	0.0253	0.0000
AT. 2 CA	X	-0.0015	0.0528	-0.0010	-0.0781	-0.0010	-0.0212
	Y	0.0057	-0.0041	0.4313	0.0013	-0.0093	-0.0161
	Z	0.0408	0.0389	-0.0015	0.0847	0.0147	0.0305
AT. 3 CA	X	0.0015	0.0528	0.0010	-0.0781	-0.0010	0.0212
	Y	0.0057	0.0041	0.4313	-0.0013	0.0093	-0.0161
	Z	-0.0408	0.0389	0.0015	0.0847	0.0147	-0.0305
AT. 4 CA	X	-0.0079	0.0193	0.0008	-0.0753	0.0557	0.0571
	Y	-0.0293	-0.0075	0.4273	-0.0003	0.0196	0.0392
	Z	-0.0391	0.0296	-0.0012	0.0782	0.0303	0.0024
AT. 5 CA	X	0.0079	0.0193	-0.0008	-0.0753	0.0557	-0.0571
	Y	-0.0293	0.0075	0.4273	0.0003	-0.0196	0.0392
	Z	0.0391	0.0296	0.0012	0.0782	0.0303	-0.0024
AT. 6 CA	X	0.0000	0.0415	0.0000	-0.0750	0.0202	0.0000
	Y	0.0311	0.0000	0.4287	0.0000	0.0000	0.0527
	Z	0.0000	0.0550	0.0000	0.0828	-0.0255	0.0000
AT. 7 CA	X	0.0000	0.0257	0.0000	-0.0745	0.0340	0.0000
	Y	-0.0209	0.0000	0.4283	0.0000	0.0000	-0.0628
	Z	0.0000	0.0226	0.0000	0.0828	0.0392	0.0000
AT. 8 CA	X	0.0030	0.0355	0.0003	-0.0766	0.0185	-0.0327
	Y	0.0223	-0.0040	0.4304	-0.0022	0.0120	-0.0217
	Z	0.0262	0.0250	0.0007	0.0777	0.0447	-0.0138
AT. 9 CA	X	-0.0030	0.0355	-0.0003	-0.0766	0.0185	0.0327
	Y	0.0223	0.0040	0.4304	0.0022	-0.0120	-0.0217
	Z	-0.0262	0.0250	-0.0007	0.0777	0.0447	0.0138
AT. 10 SI	X	0.0000	0.0363	0.0000	-0.0739	0.0121	0.0000
	Y	-0.0095	0.0000	0.4282	0.0000	0.0000	-0.0171
	Z	0.0000	0.0471	0.0000	0.0826	-0.0047	0.0000
AT. 11 SI	X	0.0000	0.0368	0.0000	-0.0769	0.0145	0.0000
	Y	0.0020	0.0000	0.4304	0.0000	0.0000	0.0013
	Z	0.0000	0.0329	0.0000	0.0807	0.0257	0.0000
AT. 12 SI	X	0.0000	0.0456	0.0000	-0.0734	0.0109	0.0000
	Y	-0.0089	0.0000	0.4277	0.0000	0.0000	0.0114
	Z	0.0000	0.0292	0.0000	0.0819	0.0176	0.0000
AT. 13 O	X	0.0000	0.0491	0.0000	-0.0671	-0.0328	0.0000
	Y	-0.1113	0.0000	0.4302	0.0000	0.0000	-0.0818
	Z	0.0000	0.0487	0.0000	0.0837	-0.0265	0.0000
AT. 14 O	X	-0.0086	0.0550	0.0015	-0.0751	-0.0026	0.0267
	Y	0.0146	0.0016	0.4311	0.0009	-0.0043	0.0099
	Z	-0.0355	0.0412	-0.0028	0.0800	0.0182	0.0139
AT. 15 O	X	0.0086	0.0550	-0.0015	-0.0751	-0.0026	-0.0267
	Y	0.0146	-0.0016	0.4311	-0.0009	0.0043	0.0099
	Z	0.0355	0.0412	0.0028	0.0800	0.0182	-0.0139
AT. 16 O	X	0.0000	0.0413	0.0000	-0.0762	0.0044	0.0000
	Y	-0.0033	0.0000	0.4309	0.0000	0.0000	-0.0405
	Z	0.0000	0.0044	0.0000	0.0806	0.0604	0.0000
AT. 17 O	X	0.0000	0.0288	0.0000	-0.0769	0.0337	0.0000
	Y	0.0050	0.0000	0.4271	0.0000	0.0000	0.0106
	Z	0.0000	0.0294	0.0000	0.0793	0.0219	0.0000
AT. 18 O	X	0.0000	0.0096	0.0000	-0.0775	0.0506	0.0000
	Y	-0.0109	0.0000	0.4285	0.0000	0.0000	0.0126
	Z	0.0000	0.0432	0.0000	0.0824	0.0192	0.0000
AT. 19 O	X	0.0000	0.0373	0.0000	-0.0772	0.0225	0.0000
	Y	0.0311	0.0000	0.4299	0.0000	0.0000	-0.0572

```
            Z     0.0000    0.0452    0.0000    0.0876   -0.0116    0.0000
AT.  20 O   X    -0.0001    0.0354   -0.0019   -0.0758    0.0193    0.0700
            Y     0.0184    0.0030    0.4282    0.0002    0.0012    0.0405
            Z    -0.0948    0.0382    0.0010    0.0793    0.0127   -0.0359
AT.  21 O   X     0.0001    0.0354    0.0019   -0.0758    0.0193   -0.0700
            Y     0.0184   -0.0030    0.4282   -0.0002   -0.0012    0.0405
            Z     0.0948    0.0382   -0.0010    0.0793    0.0127    0.0359
AT.  22 O   X     0.0000    0.0486    0.0000   -0.0769   -0.0024    0.0000
            Y     0.0519    0.0000    0.4305    0.0000    0.0000   -0.0014
            Z     0.0000    0.0371    0.0000    0.0786    0.0240    0.0000
AT.  23 O   X     0.0000    0.0301    0.0000   -0.0756    0.0315    0.0000
            Y    -0.0053    0.0000    0.4291    0.0000    0.0000   -0.0103
            Z     0.0000    0.0295    0.0000    0.0798    0.0391    0.0000
AT.  24 O   X     0.0000    0.1396    0.0000   -0.0610   -0.1836    0.0000
            Y     0.0771    0.0000    0.4295    0.0000    0.0000    0.0145
            Z     0.0000    0.0754    0.0000    0.0885   -0.0667    0.0000
AT.  25 O   X     0.0000   -0.0147    0.0000   -0.0796    0.1054    0.0000
            Y    -0.0801    0.0000    0.4288    0.0000    0.0000    0.1542
            Z     0.0000    0.0968    0.0000    0.0892   -0.0972    0.0000
AT.  26 O   X    -0.0059    0.0310    0.0022   -0.0732    0.0314    0.1116
            Y    -0.0164    0.0053    0.4274   -0.0005    0.0002   -0.0626
            Z    -0.0724   -0.0114   -0.0013    0.0800    0.0979    0.0319
AT.  27 O   X     0.0059    0.0310   -0.0022   -0.0732    0.0314   -0.1116
            Y    -0.0164   -0.0053    0.4274    0.0005   -0.0002   -0.0626
            Z     0.0724   -0.0114    0.0013    0.0800    0.0979   -0.0319

FREQ(CM**-1)      81.47     98.59    117.04    123.89    128.53    137.68

AT.   1 CA X     0.0000   -0.0803   -0.0416    0.0000    0.0000    0.0136
            Y    -0.0384    0.0000    0.0000    0.0538    0.0204    0.0000
            Z     0.0000   -0.0293    0.0258    0.0000    0.0000   -0.0221
AT.   2 CA X     0.0249   -0.0461   -0.0080   -0.0564    0.0165   -0.0116
            Y    -0.0834    0.0181    0.0179   -0.0149   -0.0167   -0.0221
            Z     0.0517   -0.0077   -0.0339    0.0063    0.0487    0.0411
AT.   3 CA X    -0.0249   -0.0461   -0.0080    0.0564   -0.0165   -0.0116
            Y    -0.0834   -0.0181   -0.0179   -0.0149   -0.0167    0.0221
            Z    -0.0517   -0.0077   -0.0339   -0.0063   -0.0487    0.0411
AT.   4 CA X    -0.0147    0.0291   -0.0209    0.0070    0.0053   -0.0168
            Y     0.0281   -0.0114    0.0122   -0.0134   -0.0115    0.0062
            Z     0.0060   -0.0211    0.0252   -0.0087   -0.0285   -0.0029
AT.   5 CA X     0.0147    0.0291   -0.0209   -0.0070   -0.0053   -0.0168
            Y     0.0281    0.0114   -0.0122   -0.0134   -0.0115   -0.0062
            Z    -0.0060   -0.0211    0.0252    0.0087    0.0285   -0.0029
AT.   6 CA X     0.0000    0.0138    0.0348    0.0000    0.0000    0.0572
            Y     0.0233    0.0000    0.0000   -0.0398    0.0024    0.0000
            Z     0.0000   -0.0105   -0.0011    0.0000    0.0000   -0.0334
AT.   7 CA X     0.0000    0.0544   -0.0274    0.0000    0.0000   -0.0315
            Y     0.0353    0.0000    0.0000    0.0080   -0.0359    0.0000
            Z     0.0000    0.0334   -0.0459    0.0000    0.0000    0.0039
AT.   8 CA X     0.0074    0.0091    0.0442    0.0318    0.0138    0.0253
            Y     0.0295   -0.0079    0.0041    0.0146   -0.0099   -0.0181
            Z    -0.0078    0.0093   -0.0064   -0.0100    0.0002   -0.0033
AT.   9 CA X    -0.0074    0.0091    0.0442   -0.0318   -0.0138    0.0253
            Y     0.0295    0.0079   -0.0041    0.0146   -0.0099    0.0181
            Z     0.0078    0.0093   -0.0064    0.0100   -0.0002   -0.0033
AT.  10 SI X     0.0000    0.0089    0.0113    0.0000    0.0000    0.0198
            Y     0.0159    0.0000    0.0000   -0.0049    0.0256    0.0000
            Z     0.0000    0.0237    0.0164    0.0000    0.0000   -0.0191
AT.  11 SI X     0.0000   -0.0055   -0.0034    0.0000    0.0000   -0.0048
            Y    -0.0183    0.0000    0.0000   -0.0001    0.0087    0.0000
            Z     0.0000    0.0014    0.0036    0.0000    0.0000    0.0113
AT.  12 SI X     0.0000    0.0249   -0.0126    0.0000    0.0000   -0.0228
            Y     0.0126    0.0000    0.0000    0.0079   -0.0099    0.0000
            Z     0.0000   -0.0040   -0.0010    0.0000    0.0000   -0.0003
AT.  13 O   X     0.0000   -0.0003   -0.0675    0.0000    0.0000    0.0309
            Y    -0.0125    0.0000    0.0000   -0.0108    0.1380    0.0000
            Z     0.0000    0.0254   -0.0015    0.0000    0.0000   -0.0150
AT.  14 O   X    -0.0075   -0.0310   -0.0108    0.0637   -0.0036   -0.0191
            Y    -0.0355    0.0022   -0.0068    0.0269    0.0228    0.0042
            Z     0.0328   -0.0335    0.0093    0.0137   -0.0313   -0.0092
AT.  15 O   X     0.0075   -0.0310   -0.0108   -0.0637    0.0036   -0.0191
            Y    -0.0355   -0.0022    0.0068    0.0269    0.0228   -0.0042
            Z    -0.0328   -0.0335    0.0093   -0.0137    0.0313   -0.0092
AT.  16 O   X     0.0000   -0.0169   -0.0004    0.0000    0.0000   -0.0180
            Y    -0.0389    0.0000    0.0000   -0.0724    0.0115    0.0000
            Z     0.0000    0.0642    0.0034    0.0000    0.0000    0.0780
AT.  17 O   X     0.0000    0.0323   -0.0042    0.0000    0.0000   -0.0182
            Y     0.0376    0.0000    0.0000   -0.0029    0.0147    0.0000
            Z     0.0000    0.0204   -0.0015    0.0000    0.0000   -0.0066
```

AT. 18 O	X	0.0000	0.0518	0.0104	0.0000	0.0000	0.0514
	Y	0.0309	0.0000	0.0000	0.0168	-0.0133	0.0000
	Z	0.0000	-0.0057	0.0035	0.0000	0.0000	0.0060
AT. 19 O	X	0.0000	0.0028	0.0551	0.0000	0.0000	0.0050
	Y	0.0419	0.0000	0.0000	0.0478	-0.0051	0.0000
	Z	0.0000	0.0382	-0.0556	0.0000	0.0000	-0.0021
AT. 20 O	X	-0.0086	0.0116	0.0339	-0.0391	-0.0176	0.0187
	Y	0.0193	0.0036	0.0015	-0.0275	0.0006	0.0068
	Z	-0.0289	0.0219	0.0637	-0.0061	0.0520	-0.0408
AT. 21 O	X	0.0086	0.0116	0.0339	0.0391	0.0176	0.0187
	Y	0.0193	-0.0036	-0.0015	-0.0275	0.0006	-0.0068
	Z	0.0289	0.0219	0.0637	0.0061	-0.0520	-0.0408
AT. 22 O	X	0.0000	-0.0280	0.0181	0.0000	0.0000	0.0008
	Y	-0.0153	0.0000	0.0000	0.0005	0.0051	0.0000
	Z	0.0000	-0.0016	-0.0063	0.0000	0.0000	0.0001
AT. 23 O	X	0.0000	0.0226	-0.0031	0.0000	0.0000	-0.0111
	Y	0.0198	0.0000	0.0000	-0.0077	0.0063	0.0000
	Z	0.0000	0.0012	0.0063	0.0000	0.0000	-0.0001
AT. 24 O	X	0.0000	0.0667	-0.0155	0.0000	0.0000	-0.0081
	Y	-0.0501	0.0000	0.0000	-0.0012	0.0175	0.0000
	Z	0.0000	0.0187	-0.0021	0.0000	0.0000	0.0076
AT. 25 O	X	0.0000	0.0131	-0.0170	0.0000	0.0000	-0.0366
	Y	0.0139	0.0000	0.0000	0.0194	0.0089	0.0000
	Z	0.0000	0.0088	0.0113	0.0000	0.0000	0.0160
AT. 26 O	X	-0.0273	0.0263	-0.0144	0.0031	0.0179	-0.0232
	Y	0.0362	-0.0023	0.0020	0.0089	-0.0258	-0.0002
	Z	0.0221	-0.0096	0.0051	0.0189	-0.0204	-0.0126
AT. 27 O	X	0.0273	0.0263	-0.0144	-0.0031	-0.0179	-0.0232
	Y	0.0362	0.0023	-0.0020	0.0089	-0.0258	0.0002
	Z	-0.0221	-0.0096	0.0051	-0.0189	0.0204	-0.0126

FREQ(CM**-1)		142.62	154.64	157.00	159.62	168.53	173.57
AT. 1 CA	X	0.0000	0.0287	0.0237	0.0000	0.0000	0.0095
	Y	-0.0074	0.0000	0.0000	-0.0075	0.0150	0.0000
	Z	0.0000	0.0504	0.0432	0.0000	0.0000	-0.0163
AT. 2 CA	X	0.0138	0.0082	0.0037	0.0286	0.0131	0.0046
	Y	-0.0060	-0.0104	-0.0003	0.0108	-0.0211	0.0319
	Z	-0.0316	-0.0104	-0.0330	0.0018	-0.0242	-0.0294
AT. 3 CA	X	-0.0138	0.0082	0.0037	-0.0286	-0.0131	0.0046
	Y	-0.0060	0.0104	0.0003	0.0108	-0.0211	-0.0319
	Z	0.0316	-0.0104	-0.0330	-0.0018	0.0242	-0.0294
AT. 4 CA	X	0.0194	-0.0153	-0.0018	0.0014	-0.0010	-0.0208
	Y	-0.0454	-0.0034	-0.0122	-0.0204	0.0277	-0.0044
	Z	-0.0282	0.0077	-0.0360	-0.0240	0.0223	-0.0010
AT. 5 CA	X	-0.0194	-0.0153	-0.0018	-0.0014	0.0010	-0.0208
	Y	-0.0454	0.0034	0.0122	-0.0204	0.0277	0.0044
	Z	0.0282	0.0077	-0.0360	0.0240	-0.0223	-0.0010
AT. 6 CA	X	0.0000	-0.0323	0.0117	0.0000	0.0000	0.0266
	Y	0.0224	0.0000	0.0000	-0.0095	-0.0172	0.0000
	Z	0.0000	-0.0058	-0.0364	0.0000	0.0000	-0.0070
AT. 7 CA	X	0.0000	-0.0009	0.0226	0.0000	0.0000	0.0037
	Y	-0.0197	0.0000	0.0000	0.0295	-0.0228	0.0000
	Z	0.0000	0.0048	0.0099	0.0000	0.0000	0.0220
AT. 8 CA	X	-0.0089	-0.0074	-0.0058	0.0345	0.0112	-0.0029
	Y	0.0480	0.0092	-0.0095	-0.0237	0.0140	-0.0105
	Z	0.0173	0.0205	-0.0048	-0.0157	0.0440	-0.0008
AT. 9 CA	X	0.0089	-0.0074	-0.0058	-0.0345	-0.0112	-0.0029
	Y	0.0480	-0.0092	0.0095	-0.0237	0.0140	0.0105
	Z	-0.0173	0.0205	-0.0048	0.0157	-0.0440	-0.0008
AT. 10 SI	X	0.0000	-0.0113	-0.0097	0.0000	0.0000	0.0105
	Y	0.0191	0.0000	0.0000	-0.0090	0.0094	0.0000
	Z	0.0000	0.0112	0.0025	0.0000	0.0000	0.0123
AT. 11 SI	X	0.0000	0.0246	-0.0048	0.0000	0.0000	0.0276
	Y	0.0035	0.0000	0.0000	0.0007	-0.0099	0.0000
	Z	0.0000	-0.0252	0.0544	0.0000	0.0000	-0.0172
AT. 12 SI	X	0.0000	-0.0102	-0.0147	0.0000	0.0000	-0.0281
	Y	-0.0118	0.0000	0.0000	0.0269	-0.0069	0.0000
	Z	0.0000	-0.0204	-0.0164	0.0000	0.0000	0.0332
AT. 13 O	X	0.0000	-0.0186	-0.0071	0.0000	0.0000	0.0751
	Y	0.0226	0.0000	0.0000	-0.0381	-0.0037	0.0000
	Z	0.0000	0.0071	0.0031	0.0000	0.0000	0.0259
AT. 14 O	X	-0.0017	-0.0044	-0.0077	-0.0238	-0.0016	0.0161
	Y	-0.0116	0.0031	-0.0008	0.0020	0.0216	-0.0021
	Z	0.0295	-0.0645	0.0572	-0.0248	-0.0653	-0.0212
AT. 15 O	X	0.0017	-0.0044	-0.0077	0.0238	0.0016	0.0161
	Y	-0.0116	-0.0031	0.0008	0.0020	0.0216	0.0021
	Z	-0.0295	-0.0645	0.0572	0.0248	0.0653	-0.0212
AT. 16 O	X	0.0000	0.0091	-0.0054	0.0000	0.0000	0.0262

	Y	0.0048	0.0000	0.0000	0.0378	-0.0118	0.0000
	Z	0.0000	0.0523	0.0572	0.0000	0.0000	-0.0182
AT. 17 O	X	0.0000	0.0183	0.0119	0.0000	0.0000	0.0057
	Y	-0.0351	0.0000	0.0000	0.0019	-0.0175	0.0000
	Z	0.0000	0.0209	0.0158	0.0000	0.0000	0.0277
AT. 18 O	X	0.0000	0.0995	-0.0238	0.0000	0.0000	0.0196
	Y	0.0434	0.0000	0.0000	-0.0371	-0.0595	0.0000
	Z	0.0000	-0.0375	0.0561	0.0000	0.0000	-0.0145
AT. 19 O	X	0.0000	-0.0062	-0.0137	0.0000	0.0000	-0.0118
	Y	0.0314	0.0000	0.0000	0.0287	0.0194	0.0000
	Z	0.0000	0.0020	0.0108	0.0000	0.0000	0.0510
AT. 20 O	X	0.0020	-0.0057	-0.0074	-0.0172	-0.0173	-0.0011
	Y	0.0182	0.0007	0.0001	-0.0095	0.0012	-0.0008
	Z	0.0027	0.0162	0.0064	-0.0281	-0.0133	-0.0006
AT. 21 O	X	-0.0020	-0.0057	-0.0074	0.0172	0.0173	-0.0011
	Y	0.0182	-0.0007	-0.0001	-0.0095	0.0012	0.0008
	Z	-0.0027	0.0162	0.0064	0.0281	0.0133	-0.0006
AT. 22 O	X	0.0000	-0.0099	0.0028	0.0000	0.0000	0.0146
	Y	-0.0013	0.0000	0.0000	0.0079	0.0336	0.0000
	Z	0.0000	0.0042	0.0101	0.0000	0.0000	-0.0008
AT. 23 O	X	0.0000	-0.0071	-0.0079	0.0000	0.0000	-0.0122
	Y	-0.0129	0.0000	0.0000	-0.0142	-0.0027	0.0000
	Z	0.0000	0.0040	0.0069	0.0000	0.0000	0.0018
AT. 24 O	X	0.0000	0.0166	-0.0049	0.0000	0.0000	-0.0469
	Y	-0.0492	0.0000	0.0000	-0.0065	-0.0323	0.0000
	Z	0.0000	-0.0089	-0.0113	0.0000	0.0000	0.0243
AT. 25 O	X	0.0000	-0.0151	-0.0300	0.0000	0.0000	-0.0150
	Y	0.0260	0.0000	0.0000	0.0987	0.0253	0.0000
	Z	0.0000	-0.0132	0.0012	0.0000	0.0000	0.0159
AT. 26 O	X	0.0154	-0.0181	-0.0144	0.0156	0.0072	-0.0204
	Y	-0.0176	0.0016	0.0001	0.0194	-0.0123	-0.0023
	Z	0.0329	-0.0342	-0.0186	0.0353	0.0042	0.0444
AT. 27 O	X	-0.0154	-0.0181	-0.0144	-0.0156	-0.0072	-0.0204
	Y	-0.0176	-0.0016	-0.0001	0.0194	-0.0123	0.0023
	Z	-0.0329	-0.0342	-0.0186	-0.0353	-0.0042	0.0444

FREQ(CM**-1)		177.15	180.11	184.93	197.81	197.85	199.48
AT. 1 CA	X	0.0000	-0.0194	0.0000	0.0158	0.0000	0.0000
	Y	0.0559	0.0000	0.0040	0.0000	0.0039	-0.0111
	Z	0.0000	-0.0352	0.0000	0.0119	0.0000	0.0000
AT. 2 CA	X	0.0168	0.0235	0.0169	-0.0109	0.0151	-0.0147
	Y	-0.0220	0.0064	-0.0011	0.0016	0.0073	0.0109
	Z	-0.0016	0.0039	-0.0064	0.0024	0.0176	0.0171
AT. 3 CA	X	-0.0168	0.0235	-0.0169	-0.0109	-0.0151	0.0147
	Y	-0.0220	-0.0064	-0.0011	-0.0016	0.0073	0.0109
	Z	0.0016	0.0039	0.0064	0.0024	-0.0176	-0.0171
AT. 4 CA	X	-0.0134	-0.0049	0.0278	-0.0014	-0.0231	-0.0421
	Y	-0.0176	0.0025	0.0167	-0.0262	0.0039	-0.0287
	Z	-0.0019	0.0066	0.0145	0.0031	0.0100	0.0442
AT. 5 CA	X	0.0134	-0.0049	-0.0278	-0.0014	0.0231	0.0421
	Y	-0.0176	-0.0025	0.0167	0.0262	0.0039	-0.0287
	Z	0.0019	0.0066	-0.0145	0.0031	-0.0100	-0.0442
AT. 6 CA	X	0.0000	-0.0369	0.0000	-0.0303	0.0000	0.0000
	Y	0.0135	0.0000	-0.0155	0.0000	-0.0438	0.0129
	Z	0.0000	0.0204	0.0000	0.0404	0.0000	0.0000
AT. 7 CA	X	0.0000	0.0013	0.0000	0.0496	0.0000	0.0000
	Y	0.0186	0.0000	-0.0191	0.0000	-0.0197	-0.0082
	Z	0.0000	-0.0274	0.0000	0.0017	0.0000	0.0000
AT. 8 CA	X	-0.0249	-0.0094	0.0145	0.0148	0.0032	-0.0030
	Y	-0.0149	0.0000	0.0039	0.0159	0.0167	0.0121
	Z	-0.0038	-0.0358	-0.0453	-0.0119	0.0272	-0.0209
AT. 9 CA	X	0.0249	-0.0094	-0.0145	0.0148	-0.0032	0.0030
	Y	-0.0149	-0.0000	0.0039	-0.0159	0.0167	0.0121
	Z	0.0038	-0.0358	0.0453	-0.0119	-0.0272	0.0209
AT. 10 SI	X	0.0000	-0.0085	0.0000	0.0374	0.0000	0.0000
	Y	-0.0203	0.0000	0.0073	0.0000	-0.0343	0.0204
	Z	0.0000	-0.0013	0.0000	-0.0223	0.0000	0.0000
AT. 11 SI	X	0.0000	0.0307	0.0000	-0.0240	0.0000	0.0000
	Y	0.0428	0.0000	0.0310	0.0000	0.0367	-0.0029
	Z	0.0000	0.0352	0.0000	0.0005	0.0000	0.0000
AT. 12 SI	X	0.0000	0.0006	0.0000	-0.0275	0.0000	0.0000
	Y	-0.0140	0.0000	-0.0389	0.0000	0.0004	-0.0164
	Z	0.0000	0.0070	0.0000	0.0156	0.0000	0.0000
AT. 13 O	X	0.0000	-0.0018	0.0000	0.0256	0.0000	0.0000
	Y	-0.0160	0.0000	-0.0082	0.0000	-0.0261	0.0144
	Z	0.0000	-0.0013	0.0000	-0.0208	0.0000	0.0000
AT. 14 O	X	0.0094	0.0135	-0.0203	-0.0261	-0.0171	-0.0028
	Y	0.0549	-0.0024	0.0143	0.0003	0.0074	0.0025

	Z	-0.0171	0.0211	0.0079	-0.0055	0.0386	-0.0185
AT. 15 O	X	-0.0094	0.0135	0.0203	-0.0261	0.0171	0.0028
	Y	0.0549	0.0024	0.0143	-0.0003	0.0074	0.0025
	Z	0.0171	0.0211	-0.0079	-0.0055	-0.0386	0.0185
AT. 16 O	X	0.0000	0.0203	0.0000	-0.0208	0.0000	0.0000
	Y	0.0078	0.0000	0.0324	0.0000	0.0334	0.0031
	Z	0.0000	0.0857	0.0000	0.0035	0.0000	0.0000
AT. 17 O	X	0.0000	0.0210	0.0000	0.0101	0.0000	0.0000
	Y	0.0129	0.0000	0.0021	0.0000	0.0287	-0.0220
	Z	0.0000	-0.0051	0.0000	-0.0063	0.0000	0.0000
AT. 18 O	X	0.0000	0.0698	0.0000	-0.0052	0.0000	0.0000
	Y	0.0160	0.0000	0.0308	0.0000	0.0549	-0.0099
	Z	0.0000	0.0292	0.0000	-0.0014	0.0000	0.0000
AT. 19 O	X	0.0000	-0.0036	0.0000	0.0372	0.0000	0.0000
	Y	-0.0462	0.0000	0.0412	0.0000	-0.0164	0.0118
	Z	0.0000	-0.0074	0.0000	-0.0252	0.0000	0.0000
AT. 20 O	X	0.0242	-0.0070	-0.0271	0.0215	-0.0079	0.0002
	Y	-0.0010	-0.0005	-0.0054	-0.0096	-0.0383	0.0209
	Z	-0.0066	0.0022	-0.0272	-0.0164	0.0060	-0.0103
AT. 21 O	X	-0.0242	-0.0070	0.0271	0.0215	0.0079	-0.0002
	Y	-0.0010	0.0005	-0.0054	0.0096	-0.0383	0.0209
	Z	0.0066	0.0022	0.0272	-0.0164	-0.0060	0.0103
AT. 22 O	X	0.0000	-0.0162	0.0000	-0.0019	0.0000	0.0000
	Y	0.0155	0.0000	0.0283	0.0000	-0.0259	0.0269
	Z	0.0000	-0.0065	0.0000	-0.0010	0.0000	0.0000
AT. 23 O	X	0.0000	-0.0001	0.0000	0.0119	0.0000	0.0000
	Y	-0.0119	0.0000	-0.0132	0.0000	-0.0003	-0.0144
	Z	0.0000	-0.0108	0.0000	-0.0071	0.0000	0.0000
AT. 24 O	X	0.0000	-0.0064	0.0000	-0.0384	0.0000	0.0000
	Y	-0.0420	0.0000	-0.0232	0.0000	0.0231	-0.0061
	Z	0.0000	0.0045	0.0000	0.0092	0.0000	0.0000
AT. 25 O	X	0.0000	0.0049	0.0000	-0.0340	0.0000	0.0000
	Y	-0.0282	0.0000	-0.0546	0.0000	0.0263	0.0343
	Z	0.0000	0.0016	0.0000	0.0282	0.0000	0.0000
AT. 26 O	X	-0.0253	0.0052	0.0016	-0.0181	0.0239	0.0139
	Y	0.0111	0.0008	-0.0370	-0.0070	-0.0157	-0.0215
	Z	0.0394	0.0244	-0.0026	-0.0017	0.0106	0.0173
AT. 27 O	X	0.0253	0.0052	-0.0016	-0.0181	-0.0239	-0.0139
	Y	0.0111	-0.0008	-0.0370	0.0070	-0.0157	-0.0215
	Z	-0.0394	0.0244	0.0026	-0.0017	-0.0106	-0.0173

FREQ(CM**-1)		210.80	214.01	217.35	220.84	226.53	245.28
AT. 1 CA	X	-0.0085	-0.0369	0.0000	-0.0135	0.0020	0.0000
	Y	0.0000	0.0000	-0.0098	0.0000	0.0000	0.0001
	Z	-0.0493	0.0228	0.0000	-0.0078	0.0194	0.0000
AT. 2 CA	X	0.0150	0.0078	0.0068	-0.0031	-0.0151	0.0139
	Y	-0.0125	0.0192	-0.0054	0.0275	0.0158	-0.0264
	Z	0.0072	0.0148	0.0101	-0.0061	0.0087	-0.0328
AT. 3 CA	X	0.0150	0.0078	-0.0068	-0.0031	-0.0151	-0.0139
	Y	0.0125	-0.0192	-0.0054	-0.0275	-0.0158	-0.0264
	Z	0.0072	0.0148	-0.0101	-0.0061	0.0087	0.0328
AT. 4 CA	X	0.0027	0.0028	0.0280	0.0186	-0.0002	-0.0227
	Y	-0.0037	-0.0232	-0.0138	0.0247	-0.0105	-0.0041
	Z	-0.0215	0.0013	0.0117	0.0231	-0.0386	-0.0058
AT. 5 CA	X	0.0027	0.0028	-0.0280	0.0186	-0.0002	0.0227
	Y	0.0037	0.0232	-0.0138	-0.0247	0.0105	-0.0041
	Z	-0.0215	0.0013	-0.0117	0.0231	-0.0386	0.0058
AT. 6 CA	X	-0.0274	0.0246	0.0000	-0.0290	-0.0039	0.0000
	Y	0.0000	0.0000	-0.0301	0.0000	0.0000	-0.0099
	Z	-0.0160	0.0039	0.0000	-0.0204	-0.0007	0.0000
AT. 7 CA	X	0.0021	0.0175	0.0000	-0.0099	-0.0358	0.0000
	Y	0.0000	0.0000	0.0118	0.0000	0.0000	0.0208
	Z	0.0332	0.0083	0.0000	-0.0157	-0.0337	0.0000
AT. 8 CA	X	0.0038	-0.0017	-0.0331	0.0048	-0.0064	0.0023
	Y	0.0059	-0.0113	0.0345	0.0141	0.0309	-0.0015
	Z	0.0234	0.0105	-0.0203	0.0233	0.0225	-0.0166
AT. 9 CA	X	0.0038	-0.0017	0.0331	0.0048	-0.0064	-0.0023
	Y	-0.0059	0.0113	0.0345	-0.0141	-0.0309	-0.0015
	Z	0.0234	0.0105	0.0203	0.0233	0.0225	0.0166
AT. 10 SI	X	0.0159	-0.0292	0.0000	0.0150	0.0134	0.0000
	Y	0.0000	0.0000	-0.0291	0.0000	0.0000	0.0027
	Z	0.0146	-0.0356	0.0000	-0.0175	-0.0108	0.0000
AT. 11 SI	X	0.0194	0.0343	0.0000	0.0068	0.0097	0.0000
	Y	0.0000	0.0000	-0.0081	0.0000	0.0000	0.0064
	Z	0.0035	-0.0049	0.0000	0.0187	-0.0003	0.0000
AT. 12 SI	X	-0.0318	-0.0162	0.0000	-0.0136	0.0083	0.0000
	Y	0.0000	0.0000	0.0299	0.0000	0.0000	0.0047
	Z	-0.0103	0.0060	0.0000	-0.0211	0.0312	0.0000

AT. 13 O	X	-0.0299	-0.0553	0.0000	0.0484	0.0185	0.0000
	Y	0.0000	0.0000	-0.0045	0.0000	0.0000	-0.0002
	Z	0.0014	-0.0373	0.0000	-0.0088	-0.0017	0.0000
AT. 14 O	X	0.0171	0.0228	-0.0057	0.0066	0.0100	0.0114
	Y	-0.0031	-0.0088	0.0054	0.0014	-0.0032	0.0075
	Z	0.0078	0.0020	-0.0356	0.0122	0.0080	0.0070
AT. 15 O	X	0.0171	0.0228	0.0057	0.0066	0.0100	-0.0114
	Y	0.0031	0.0088	0.0054	-0.0014	0.0032	0.0075
	Z	0.0078	0.0020	0.0356	0.0122	0.0080	-0.0070
AT. 16 O	X	0.0197	0.0350	0.0000	0.0097	0.0053	0.0000
	Y	0.0000	0.0000	0.0010	0.0000	0.0000	-0.0252
	Z	-0.0212	-0.0218	0.0000	0.0031	0.0161	0.0000
AT. 17 O	X	-0.0127	0.0144	0.0000	0.0088	-0.0039	0.0000
	Y	0.0000	0.0000	0.0001	0.0000	0.0000	0.0093
	Z	-0.0344	-0.0102	0.0000	0.0226	-0.0296	0.0000
AT. 18 O	X	0.0023	0.0132	0.0000	-0.0104	0.0197	0.0000
	Y	0.0000	0.0000	-0.0328	0.0000	0.0000	0.0206
	Z	0.0056	-0.0017	0.0000	0.0199	0.0031	0.0000
AT. 19 O	X	0.0321	-0.0228	0.0000	-0.0046	0.0112	0.0000
	Y	0.0000	0.0000	-0.0088	0.0000	0.0000	-0.0048
	Z	-0.0176	-0.0432	0.0000	0.0157	-0.0086	0.0000
AT. 20 O	X	0.0173	-0.0168	0.0024	0.0059	0.0081	0.0112
	Y	-0.0046	0.0063	-0.0294	0.0004	-0.0048	0.0078
	Z	0.0411	-0.0359	0.0188	-0.0346	-0.0028	0.0087
AT. 21 O	X	0.0173	-0.0168	-0.0024	0.0059	0.0081	-0.0112
	Y	0.0046	-0.0063	-0.0294	-0.0004	0.0048	0.0078
	Z	0.0411	-0.0359	-0.0188	-0.0346	-0.0028	-0.0087
AT. 22 O	X	0.0233	-0.0011	0.0000	-0.0005	0.0088	0.0000
	Y	0.0000	0.0000	0.0180	0.0000	0.0000	0.0259
	Z	-0.0142	0.0012	0.0000	0.0005	0.0042	0.0000
AT. 23 O	X	-0.0144	-0.0017	0.0000	0.0025	0.0006	0.0000
	Y	0.0000	0.0000	0.0006	0.0000	0.0000	0.0162
	Z	0.0044	0.0111	0.0000	0.0198	-0.0166	0.0000
AT. 24 O	X	-0.0110	-0.0083	0.0000	0.0043	0.0105	0.0000
	Y	0.0000	0.0000	0.0303	0.0000	0.0000	0.0540
	Z	-0.0024	0.0039	0.0000	-0.0100	0.0268	0.0000
AT. 25 O	X	-0.0366	-0.0199	0.0000	-0.0355	0.0248	0.0000
	Y	0.0000	0.0000	0.0069	0.0000	0.0000	0.0270
	Z	-0.0008	0.0108	0.0000	0.0037	0.0038	0.0000
AT. 26 O	X	-0.0274	-0.0113	0.0048	-0.0111	0.0081	0.0308
	Y	-0.0017	-0.0033	0.0214	-0.0030	0.0007	-0.0227
	Z	-0.0046	-0.0047	-0.0090	-0.0365	0.0205	-0.0525
AT. 27 O	X	-0.0274	-0.0113	-0.0048	-0.0111	0.0081	-0.0308
	Y	0.0017	0.0033	0.0214	0.0030	-0.0007	-0.0227
	Z	-0.0046	-0.0047	0.0090	-0.0365	0.0205	0.0525

FREQ(CM**-1)		245.97	255.15	258.08	260.36	269.14	269.82
AT. 1 CA	X	0.0195	0.0000	-0.0220	0.0079	0.0090	0.0375
	Y	0.0000	-0.0001	0.0000	0.0000	0.0000	0.0000
	Z	-0.0065	0.0000	0.0128	0.0137	0.0053	-0.0484
AT. 2 CA	X	0.0029	-0.0061	-0.0002	-0.0155	-0.0053	-0.0083
	Y	0.0046	0.0094	0.0009	0.0157	0.0198	0.0150
	Z	0.0123	-0.0018	0.0277	0.0112	0.0158	-0.0004
AT. 3 CA	X	0.0029	0.0061	-0.0002	-0.0155	-0.0053	-0.0083
	Y	-0.0046	0.0094	-0.0009	-0.0157	-0.0198	-0.0150
	Z	0.0123	0.0018	0.0277	0.0112	0.0158	-0.0004
AT. 4 CA	X	0.0361	-0.0293	-0.0110	0.0203	-0.0207	-0.0132
	Y	0.0122	0.0277	-0.0244	0.0124	0.0321	-0.0187
	Z	-0.0026	-0.0388	0.0047	-0.0215	0.0013	0.0010
AT. 5 CA	X	0.0361	0.0293	-0.0110	0.0203	-0.0207	-0.0132
	Y	-0.0122	0.0277	0.0244	-0.0124	-0.0321	0.0187
	Z	-0.0026	0.0388	0.0047	-0.0215	0.0013	0.0010
AT. 6 CA	X	0.0190	0.0000	0.0053	-0.0062	0.0200	0.0181
	Y	0.0000	0.0001	0.0000	0.0000	0.0000	0.0000
	Z	-0.0073	0.0000	-0.0220	0.0392	-0.0107	0.0184
AT. 7 CA	X	0.0219	0.0000	0.0175	-0.0328	0.0294	0.0059
	Y	0.0000	-0.0061	0.0000	0.0000	0.0000	0.0000
	Z	-0.0478	0.0000	-0.0283	0.0245	0.0270	-0.0201
AT. 8 CA	X	-0.0124	-0.0060	-0.0012	0.0062	-0.0266	-0.0038
	Y	-0.0009	0.0235	0.0100	-0.0112	0.0226	0.0046
	Z	-0.0015	-0.0229	-0.0112	-0.0234	-0.0128	0.0138
AT. 9 CA	X	-0.0124	0.0060	-0.0012	0.0062	-0.0266	-0.0038
	Y	0.0009	0.0235	-0.0100	0.0112	-0.0226	-0.0046
	Z	-0.0015	0.0229	-0.0112	-0.0234	-0.0128	0.0138
AT. 10 SI	X	-0.0247	0.0000	0.0099	0.0026	0.0064	-0.0106
	Y	0.0000	-0.0190	0.0000	0.0000	0.0000	0.0000
	Z	0.0172	0.0000	0.0331	0.0038	-0.0000	-0.0159
AT. 11 SI	X	-0.0084	0.0000	0.0072	0.0125	0.0038	-0.0018

	Y	0.0000	-0.0160	0.0000	0.0000	0.0000	0.0000
	Z	-0.0154	0.0000	-0.0070	-0.0036	0.0013	0.0064
AT. 12 SI	X	-0.0198	0.0000	-0.0141	-0.0008	0.0048	0.0048
	Y	0.0000	-0.0246	0.0000	0.0000	0.0000	0.0000
	Z	0.0201	0.0000	-0.0259	-0.0105	0.0027	-0.0104
AT. 13 O	X	-0.0161	0.0000	0.0312	-0.0075	-0.0103	-0.0242
	Y	0.0000	-0.0109	0.0000	0.0000	0.0000	0.0000
	Z	0.0180	0.0000	0.0343	0.0017	-0.0053	-0.0182
AT. 14 O	X	-0.0080	-0.0042	0.0103	0.0080	-0.0005	-0.0023
	Y	0.0004	-0.0068	-0.0083	-0.0071	0.0009	0.0019
	Z	-0.0149	-0.0184	0.0164	0.0082	0.0015	0.0022
AT. 15 O	X	-0.0080	0.0042	0.0103	0.0080	-0.0005	-0.0023
	Y	-0.0004	-0.0068	0.0083	0.0071	-0.0009	-0.0019
	Z	-0.0149	0.0184	0.0164	0.0082	0.0015	0.0022
AT. 16 O	X	-0.0096	0.0000	0.0101	0.0144	0.0068	0.0001
	Y	0.0000	-0.0056	0.0000	0.0000	0.0000	0.0000
	Z	0.0092	0.0000	-0.0223	-0.0194	0.0061	0.0065
AT. 17 O	X	0.0079	0.0000	-0.0084	-0.0109	0.0136	-0.0078
	Y	0.0000	-0.0136	0.0000	0.0000	0.0000	0.0000
	Z	-0.0056	0.0000	0.0120	0.0422	-0.0001	0.0568
AT. 18 O	X	-0.0081	0.0000	0.0018	0.0260	-0.0068	0.0027
	Y	0.0000	-0.0260	0.0000	0.0000	0.0000	0.0000
	Z	-0.0110	0.0000	-0.0075	-0.0056	0.0014	0.0068
AT. 19 O	X	-0.0162	0.0000	0.0023	-0.0012	0.0295	-0.0076
	Y	0.0000	-0.0231	0.0000	0.0000	0.0000	0.0000
	Z	0.0039	0.0000	0.0382	0.0079	-0.0355	-0.0114
AT. 20 O	X	-0.0137	0.0020	0.0012	0.0079	0.0079	-0.0029
	Y	0.0027	-0.0170	-0.0028	0.0008	0.0023	-0.0024
	Z	0.0273	0.0097	0.0113	0.0129	0.0064	0.0195
AT. 21 O	X	-0.0137	-0.0020	0.0012	0.0079	0.0079	-0.0029
	Y	-0.0027	-0.0170	0.0028	-0.0008	-0.0023	0.0024
	Z	0.0273	-0.0097	0.0113	0.0129	0.0064	0.0195
AT. 22 O	X	0.0027	0.0000	0.0057	0.0059	0.0195	0.0025
	Y	0.0000	-0.0025	0.0000	0.0000	0.0000	0.0000
	Z	-0.0030	0.0000	0.0067	0.0145	-0.0229	0.0255
AT. 23 O	X	-0.0046	0.0000	0.0110	0.0034	-0.0089	0.0030
	Y	0.0000	0.0086	0.0000	0.0000	0.0000	0.0000
	Z	-0.0197	0.0000	-0.0029	-0.0026	-0.0159	0.0437
AT. 24 O	X	-0.0219	0.0000	-0.0266	-0.0325	0.0114	0.0073
	Y	0.0000	-0.0272	0.0000	0.0000	0.0000	0.0000
	Z	0.0193	0.0000	-0.0249	-0.0210	0.0042	-0.0136
AT. 25 O	X	-0.0387	0.0000	0.0155	-0.0047	0.0090	0.0125
	Y	0.0000	0.0023	0.0000	0.0000	0.0000	0.0000
	Z	0.0348	0.0000	-0.0524	-0.0064	-0.0055	-0.0124
AT. 26 O	X	-0.0258	-0.0002	-0.0033	-0.0134	0.0036	0.0117
	Y	-0.0002	-0.0193	-0.0051	0.0062	-0.0004	-0.0061
	Z	-0.0029	0.0188	-0.0201	-0.0274	-0.0140	-0.0202
AT. 27 O	X	-0.0258	0.0002	-0.0033	-0.0134	0.0036	0.0117
	Y	0.0002	-0.0193	0.0051	-0.0062	0.0004	0.0061
	Z	-0.0029	-0.0188	-0.0201	-0.0274	-0.0140	-0.0202

FREQ(CM**-1)		285.54	291.45	294.61	295.23	301.71	312.84
AT. 1 CA	X	0.0000	-0.0099	0.0000	0.0000	-0.0191	0.0000
	Y	0.0092	0.0000	-0.0137	0.0091	0.0000	-0.0208
	Z	0.0000	0.0007	0.0000	0.0000	-0.0002	0.0000
AT. 2 CA	X	0.0082	0.0069	0.0174	-0.0311	0.0417	-0.0071
	Y	0.0099	-0.0124	0.0082	-0.0208	0.0162	-0.0055
	Z	0.0062	0.0027	-0.0054	-0.0077	0.0039	0.0044
AT. 3 CA	X	-0.0082	0.0069	-0.0174	0.0311	0.0417	0.0071
	Y	0.0099	0.0124	0.0082	-0.0208	-0.0162	-0.0055
	Z	-0.0062	0.0027	0.0054	0.0077	0.0039	-0.0044
AT. 4 CA	X	-0.0005	0.0032	-0.0109	-0.0065	0.0008	0.0024
	Y	0.0023	0.0021	0.0047	0.0013	-0.0027	0.0059
	Z	-0.0030	-0.0072	-0.0045	-0.0082	-0.0113	0.0015
AT. 5 CA	X	0.0005	0.0032	0.0109	0.0065	0.0008	-0.0024
	Y	0.0023	-0.0021	0.0047	0.0013	0.0027	0.0059
	Z	0.0030	-0.0072	0.0045	0.0082	-0.0113	-0.0015
AT. 6 CA	X	0.0000	-0.0072	0.0000	0.0000	0.0160	0.0000
	Y	-0.0129	0.0000	0.0052	-0.0036	0.0000	0.0396
	Z	0.0000	-0.0310	0.0000	0.0000	0.0195	0.0000
AT. 7 CA	X	0.0000	-0.0049	0.0000	0.0000	-0.0195	0.0000
	Y	0.0469	0.0000	-0.0469	-0.0123	0.0000	0.0258
	Z	0.0000	0.0028	0.0000	0.0000	0.0088	0.0000
AT. 8 CA	X	-0.0310	0.0011	-0.0238	-0.0242	-0.0044	0.0009
	Y	-0.0088	0.0186	-0.0142	-0.0105	0.0053	-0.0066
	Z	0.0106	-0.0170	-0.0058	-0.0008	0.0056	0.0093
AT. 9 CA	X	0.0310	0.0011	0.0238	0.0242	-0.0044	-0.0009
	Y	-0.0088	-0.0186	-0.0142	-0.0105	-0.0053	-0.0066

	Z	-0.0106	-0.0170	0.0058	0.0008	0.0056	-0.0093
AT. 10 SI	X	0.0000	0.0105	0.0000	0.0000	0.0088	0.0000
	Y	0.0074	0.0000	-0.0013	0.0028	0.0000	-0.0176
	Z	0.0000	-0.0071	0.0000	0.0000	0.0035	0.0000
AT. 11 SI	X	0.0000	0.0024	0.0000	0.0000	-0.0192	0.0000
	Y	-0.0133	0.0000	-0.0015	0.0140	0.0000	0.0083
	Z	0.0000	-0.0178	0.0000	0.0000	-0.0086	0.0000
AT. 12 SI	X	0.0000	-0.0063	0.0000	0.0000	-0.0015	0.0000
	Y	-0.0154	0.0000	0.0118	0.0063	0.0000	0.0053
	Z	0.0000	0.0139	0.0000	0.0000	-0.0080	0.0000
AT. 13 O	X	0.0000	-0.0232	0.0000	0.0000	0.0040	0.0000
	Y	0.0282	0.0000	0.0115	0.0046	0.0000	-0.0031
	Z	0.0000	-0.0115	0.0000	0.0000	0.0013	0.0000
AT. 14 O	X	0.0133	0.0132	0.0255	-0.0399	-0.0362	0.0018
	Y	-0.0089	0.0014	0.0076	-0.0053	-0.0039	0.0099
	Z	0.0092	-0.0048	0.0061	-0.0032	-0.0184	-0.0050
AT. 15 O	X	-0.0133	0.0132	-0.0255	0.0399	-0.0362	-0.0018
	Y	-0.0089	-0.0014	0.0076	-0.0053	0.0039	0.0099
	Z	-0.0092	-0.0048	-0.0061	0.0032	-0.0184	0.0050
AT. 16 O	X	0.0000	0.0009	0.0000	0.0000	-0.0276	0.0000
	Y	-0.0139	0.0000	-0.0471	0.0699	0.0000	0.0119
	Z	0.0000	-0.0065	0.0000	0.0000	0.0333	0.0000
AT. 17 O	X	0.0000	0.0145	0.0000	0.0000	0.0282	0.0000
	Y	0.0203	0.0000	0.0007	-0.0045	0.0000	-0.0683
	Z	0.0000	0.0804	0.0000	0.0000	-0.0033	0.0000
AT. 18 O	X	0.0000	-0.0217	0.0000	0.0000	-0.0374	0.0000
	Y	-0.0251	0.0000	0.0199	0.0015	0.0000	0.0350
	Z	0.0000	-0.0125	0.0000	0.0000	-0.0047	0.0000
AT. 19 O	X	0.0000	0.0082	0.0000	0.0000	0.0033	0.0000
	Y	0.0456	0.0000	0.0405	0.0471	0.0000	-0.0059
	Z	0.0000	-0.0078	0.0000	0.0000	0.0089	0.0000
AT. 20 O	X	-0.0236	0.0119	-0.0295	-0.0138	0.0072	-0.0305
	Y	-0.0028	0.0015	-0.0157	-0.0039	-0.0008	-0.0404
	Z	-0.0254	-0.0043	-0.0129	-0.0078	-0.0012	0.0165
AT. 21 O	X	0.0236	0.0119	0.0295	0.0138	0.0072	0.0305
	Y	-0.0028	-0.0015	-0.0157	-0.0039	0.0008	-0.0404
	Z	0.0254	-0.0043	0.0129	0.0078	-0.0012	-0.0165
AT. 22 O	X	0.0000	-0.0459	0.0000	0.0000	-0.0119	0.0000
	Y	-0.0200	0.0000	0.0272	-0.0177	0.0000	0.0469
	Z	0.0000	0.0605	0.0000	0.0000	0.0072	0.0000
AT. 23 O	X	0.0000	0.0010	0.0000	0.0000	0.0032	0.0000
	Y	-0.0058	0.0000	-0.0030	0.0024	0.0000	-0.0016
	Z	0.0000	0.0261	0.0000	0.0000	0.0122	0.0000
AT. 24 O	X	0.0000	0.0145	0.0000	0.0000	-0.0155	0.0000
	Y	-0.0066	0.0000	0.0218	-0.0063	0.0000	0.0025
	Z	0.0000	0.0220	0.0000	0.0000	-0.0128	0.0000
AT. 25 O	X	0.0000	-0.0124	0.0000	0.0000	0.0114	0.0000
	Y	-0.0148	0.0000	0.0178	0.0300	0.0000	-0.0100
	Z	0.0000	0.0143	0.0000	0.0000	-0.0162	0.0000
AT. 26 O	X	0.0307	-0.0039	-0.0186	-0.0069	-0.0053	0.0196
	Y	-0.0362	-0.0011	0.0282	0.0086	0.0003	-0.0102
	Z	0.0088	0.0197	0.0100	-0.0187	-0.0109	0.0038
AT. 27 O	X	-0.0307	-0.0039	0.0186	0.0069	-0.0053	-0.0196
	Y	-0.0362	0.0011	0.0282	0.0086	-0.0003	-0.0102
	Z	-0.0088	0.0197	-0.0100	0.0187	-0.0109	-0.0038

FREQ(CM**-1)		315.59	317.55	318.19	326.59	332.04	340.34
AT. 1 CA	X	0.0000	0.0000	-0.0073	0.0105	0.0077	0.0000
	Y	0.0252	-0.0370	0.0000	0.0000	0.0000	-0.0396
	Z	0.0000	0.0000	0.0040	0.0046	-0.0013	0.0000
AT. 2 CA	X	-0.0056	-0.0072	-0.0062	-0.0007	0.0010	-0.0162
	Y	-0.0003	0.0017	-0.0113	0.0156	0.0070	0.0012
	Z	-0.0008	0.0072	-0.0073	0.0140	0.0077	-0.0082
AT. 3 CA	X	0.0056	0.0072	-0.0062	-0.0007	0.0010	0.0162
	Y	-0.0003	0.0017	0.0113	-0.0156	-0.0070	0.0012
	Z	0.0008	-0.0072	-0.0073	0.0140	0.0077	0.0082
AT. 4 CA	X	0.0010	0.0004	0.0009	-0.0008	-0.0206	0.0046
	Y	-0.0002	0.0071	0.0034	-0.0119	0.0183	-0.0064
	Z	-0.0019	-0.0120	0.0013	0.0040	-0.0131	-0.0025
AT. 5 CA	X	-0.0010	-0.0004	0.0009	-0.0008	-0.0206	-0.0046
	Y	-0.0002	0.0071	-0.0034	0.0119	-0.0183	-0.0064
	Z	0.0019	0.0120	0.0013	0.0040	-0.0131	0.0025
AT. 6 CA	X	0.0000	0.0000	0.0131	-0.0280	-0.0088	0.0000
	Y	-0.0145	-0.0483	0.0000	0.0000	0.0000	0.0085
	Z	0.0000	0.0000	0.0143	-0.0279	0.0089	0.0000
AT. 7 CA	X	0.0000	0.0000	0.0018	-0.0137	0.0192	0.0000
	Y	0.0006	0.0094	0.0000	0.0000	0.0000	0.0036
	Z	0.0000	0.0000	-0.0164	-0.0035	-0.0118	0.0000

```
AT.   8 CA X    -0.0005   -0.0043   -0.0343   -0.0038    0.0208    0.0012
           Y    -0.0030   -0.0049   -0.0226   -0.0339   -0.0090   -0.0008
           Z     0.0037    0.0042    0.0074    0.0026    0.0132    0.0042
AT.   9 CA X     0.0005    0.0043   -0.0343   -0.0038    0.0208   -0.0012
           Y    -0.0030   -0.0049    0.0226    0.0339    0.0090   -0.0008
           Z    -0.0037   -0.0042    0.0074    0.0026    0.0132   -0.0042
AT.  10 SI X     0.0000    0.0000    0.0241    0.0052    0.0021    0.0000
           Y    -0.0079    0.0058    0.0000    0.0000    0.0000   -0.0060
           Z     0.0000    0.0000   -0.0036   -0.0084    0.0061    0.0000
AT.  11 SI X     0.0000    0.0000    0.0072   -0.0077    0.0071    0.0000
           Y    -0.0239    0.0133    0.0000    0.0000    0.0000    0.0002
           Z     0.0000    0.0000    0.0009   -0.0006    0.0073    0.0000
AT.  12 SI X     0.0000    0.0000   -0.0011    0.0012   -0.0018    0.0000
           Y     0.0040    0.0048    0.0000    0.0000    0.0000   -0.0123
           Z     0.0000    0.0000   -0.0057    0.0029   -0.0009    0.0000
AT.  13 O  X     0.0000    0.0000    0.0247    0.0073   -0.0272    0.0000
           Y     0.0330    0.0277    0.0000    0.0000    0.0000    0.0087
           Z     0.0000    0.0000   -0.0043   -0.0045   -0.0013    0.0000
AT.  14 O  X    -0.0303    0.0120    0.0155   -0.0077    0.0113   -0.0010
           Y    -0.0418    0.0236    0.0022   -0.0006    0.0083    0.0199
           Z     0.0164   -0.0128    0.0019   -0.0056   -0.0082   -0.0245
AT.  15 O  X     0.0303   -0.0120    0.0155   -0.0077    0.0113    0.0010
           Y    -0.0418    0.0236   -0.0022    0.0006   -0.0083    0.0199
           Z    -0.0164    0.0128    0.0019   -0.0056   -0.0082    0.0245
AT.  16 O  X     0.0000    0.0000    0.0064   -0.0060    0.0066    0.0000
           Y    -0.0073    0.0051    0.0000    0.0000    0.0000   -0.0059
           Z     0.0000    0.0000   -0.0070   -0.0009   -0.0090    0.0000
AT.  17 O  X     0.0000    0.0000    0.0120    0.0483    0.0145    0.0000
           Y     0.0178   -0.0505    0.0000    0.0000    0.0000    0.1039
           Z     0.0000    0.0000   -0.0252    0.0340   -0.0063    0.0000
AT.  18 O  X     0.0000    0.0000    0.0047   -0.0196   -0.0203    0.0000
           Y     0.0142    0.0409    0.0000    0.0000    0.0000    0.0259
           Z     0.0000    0.0000    0.0018   -0.0012    0.0096    0.0000
AT.  19 O  X     0.0000    0.0000    0.0325    0.0221   -0.0151    0.0000
           Y    -0.0383   -0.0086    0.0000    0.0000    0.0000   -0.0166
           Z     0.0000    0.0000   -0.0164   -0.0321    0.0302    0.0000
AT.  20 O  X    -0.0008    0.0262    0.0374    0.0101    0.0035   -0.0098
           Y     0.0006    0.0311    0.0075   -0.0042    0.0048   -0.0107
           Z    -0.0188   -0.0242    0.0057    0.0167   -0.0039   -0.0053
AT.  21 O  X     0.0008   -0.0262    0.0374    0.0101    0.0035    0.0098
           Y     0.0006    0.0311   -0.0075    0.0042   -0.0048   -0.0107
           Z     0.0188    0.0242    0.0057    0.0167   -0.0039    0.0053
AT.  22 O  X     0.0000    0.0000   -0.0514    0.0206   -0.0656    0.0000
           Y     0.1009    0.0184    0.0000    0.0000    0.0000    0.0137
           Z     0.0000    0.0000    0.0302   -0.0183    0.0194    0.0000
AT.  23 O  X     0.0000    0.0000    0.0043    0.0034    0.0028    0.0000
           Y    -0.0103   -0.0082    0.0000    0.0000    0.0000    0.0160
           Z     0.0000    0.0000    0.0231   -0.0351   -0.0562    0.0000
AT.  24 O  X     0.0000    0.0000   -0.0079    0.0107   -0.0144    0.0000
           Y     0.0002   -0.0147    0.0000    0.0000    0.0000   -0.0212
           Z     0.0000    0.0000   -0.0073    0.0084   -0.0087    0.0000
AT.  25 O  X     0.0000    0.0000    0.0002    0.0044   -0.0023    0.0000
           Y     0.0005   -0.0010    0.0000    0.0000    0.0000    0.0084
           Z     0.0000    0.0000   -0.0017   -0.0062   -0.0013    0.0000
AT.  26 O  X    -0.0001   -0.0010   -0.0078    0.0108    0.0128   -0.0026
           Y     0.0050    0.0050    0.0023   -0.0004   -0.0065   -0.0108
           Z     0.0113    0.0037   -0.0075    0.0102   -0.0020    0.0043
AT.  27 O  X     0.0001    0.0010   -0.0078    0.0108    0.0128    0.0026
           Y     0.0050    0.0050   -0.0023    0.0004    0.0065   -0.0108
           Z    -0.0113   -0.0037   -0.0075    0.0102   -0.0020   -0.0043

FREQ(CM**-1)    346.29    348.34    355.93    369.68    381.04    388.93

AT.   1 CA X     0.0000    0.0028   -0.0136    0.0051    0.0000    0.0109
           Y     0.0121    0.0000    0.0000    0.0000   -0.0021    0.0000
           Z     0.0000   -0.0069   -0.0026   -0.0010    0.0000    0.0047
AT.   2 CA X     0.0051   -0.0117   -0.0005   -0.0083    0.0020   -0.0059
           Y     0.0142   -0.0137   -0.0142    0.0028    0.0020    0.0125
           Z     0.0104   -0.0040    0.0007    0.0027   -0.0011    0.0051
AT.   3 CA X    -0.0051   -0.0117   -0.0005   -0.0083   -0.0020   -0.0059
           Y     0.0142    0.0137    0.0142   -0.0028    0.0020   -0.0125
           Z    -0.0104   -0.0040    0.0007    0.0027    0.0011    0.0051
AT.   4 CA X     0.0035    0.0024   -0.0115   -0.0022    0.0013    0.0019
           Y    -0.0014   -0.0116    0.0171    0.0056    0.0001   -0.0031
           Z     0.0020    0.0076   -0.0127   -0.0027    0.0034   -0.0000
AT.   5 CA X    -0.0035    0.0024   -0.0115   -0.0022   -0.0013    0.0019
           Y    -0.0014    0.0116   -0.0171   -0.0056    0.0001    0.0031
           Z    -0.0020    0.0076   -0.0127   -0.0027   -0.0034   -0.0000
AT.   6 CA X     0.0000    0.0176   -0.0040   -0.0011    0.0000   -0.0112
```

	Y	-0.0034	0.0000	0.0000	0.0000	0.0102	0.0000
	Z	0.0000	0.0080	0.0075	0.0043	0.0000	-0.0086
AT. 7 CA	X	0.0000	-0.0246	0.0077	0.0016	0.0000	-0.0019
	Y	0.0096	0.0000	0.0000	0.0000	-0.0004	0.0000
	Z	0.0000	0.0095	-0.0110	0.0025	0.0000	0.0034
AT. 8 CA	X	-0.0041	-0.0024	0.0028	0.0129	0.0016	0.0077
	Y	-0.0038	0.0172	-0.0039	-0.0030	0.0078	-0.0036
	Z	0.0024	-0.0020	0.0059	-0.0031	-0.0026	-0.0104
AT. 9 CA	X	0.0041	-0.0024	0.0028	0.0129	-0.0016	0.0077
	Y	-0.0038	-0.0172	0.0039	0.0030	0.0078	0.0036
	Z	-0.0024	-0.0020	0.0059	-0.0031	0.0026	-0.0104
AT. 10 SI	X	0.0000	-0.0019	-0.0104	-0.0075	0.0000	-0.0014
	Y	-0.0076	0.0000	0.0000	0.0000	-0.0152	0.0000
	Z	0.0000	0.0040	0.0006	0.0032	0.0000	0.0119
AT. 11 SI	X	0.0000	0.0051	-0.0094	0.0030	0.0000	0.0048
	Y	0.0008	0.0000	0.0000	0.0000	0.0085	0.0000
	Z	0.0000	0.0013	-0.0036	0.0002	0.0000	-0.0127
AT. 12 SI	X	0.0000	-0.0050	-0.0008	-0.0095	0.0000	0.0036
	Y	-0.0139	0.0000	0.0000	0.0000	0.0016	0.0000
	Z	0.0000	-0.0042	0.0012	-0.0029	0.0000	-0.0022
AT. 13 O	X	0.0000	-0.0012	0.0203	0.0235	0.0000	-0.0010
	Y	-0.0221	0.0000	0.0000	0.0000	0.0403	0.0000
	Z	0.0000	0.0019	0.0058	0.0109	0.0000	0.0103
AT. 14 O	X	-0.0151	0.0189	-0.0169	0.0211	0.0254	0.0144
	Y	-0.0046	0.0051	-0.0144	0.0105	0.0075	0.0019
	Z	-0.0068	0.0014	0.0114	-0.0105	0.0251	-0.0084
AT. 15 O	X	0.0151	0.0189	-0.0169	0.0211	-0.0254	0.0144
	Y	-0.0046	-0.0051	0.0144	-0.0105	0.0075	-0.0019
	Z	0.0068	0.0014	0.0114	-0.0105	-0.0251	-0.0084
AT. 16 O	X	0.0000	0.0113	0.0016	-0.0005	0.0000	-0.0047
	Y	-0.0444	0.0000	0.0000	0.0000	0.0221	0.0000
	Z	0.0000	0.0042	-0.0280	0.0187	0.0000	0.0233
AT. 17 O	X	0.0000	0.0874	0.0661	0.0258	0.0000	0.0056
	Y	-0.0070	0.0000	0.0000	0.0000	-0.0091	0.0000
	Z	0.0000	0.0025	0.0032	-0.0251	0.0000	-0.0489
AT. 18 O	X	0.0000	-0.0175	0.0279	-0.0294	0.0000	-0.0228
	Y	0.0333	0.0000	0.0000	0.0000	-0.0346	0.0000
	Z	0.0000	0.0072	-0.0069	0.0022	0.0000	-0.0106
AT. 19 O	X	0.0000	-0.0071	-0.0036	0.0137	0.0000	0.0033
	Y	0.0286	0.0000	0.0000	0.0000	-0.0501	0.0000
	Z	0.0000	0.0100	-0.0112	-0.0303	0.0000	-0.0027
AT. 20 O	X	0.0114	-0.0054	-0.0165	-0.0379	-0.0143	-0.0007
	Y	-0.0046	-0.0031	-0.0045	-0.0198	-0.0143	0.0066
	Z	0.0230	0.0062	-0.0047	0.0061	-0.0419	0.0023
AT. 21 O	X	-0.0114	-0.0054	-0.0165	-0.0379	0.0143	-0.0007
	Y	-0.0046	0.0031	0.0045	0.0198	-0.0143	-0.0066
	Z	-0.0230	0.0062	-0.0047	0.0061	0.0419	0.0023
AT. 22 O	X	0.0000	-0.0236	0.0555	-0.0135	0.0000	-0.0087
	Y	0.0130	0.0000	0.0000	0.0000	-0.0107	0.0000
	Z	0.0000	-0.0489	0.0440	0.0143	0.0000	0.0139
AT. 23 O	X	0.0000	0.0065	0.0009	0.0446	0.0000	-0.0619
	Y	0.0001	0.0000	0.0000	0.0000	-0.0085	0.0000
	Z	0.0000	-0.0200	0.0240	0.0333	0.0000	0.0611
AT. 24 O	X	0.0000	-0.0183	-0.0159	0.0214	0.0000	-0.0118
	Y	-0.0697	0.0000	0.0000	0.0000	-0.0354	0.0000
	Z	0.0000	-0.0117	-0.0047	0.0116	0.0000	-0.0043
AT. 25 O	X	0.0000	-0.0194	-0.0103	0.0211	0.0000	-0.0149
	Y	0.0444	0.0000	0.0000	0.0000	0.0318	0.0000
	Z	0.0000	0.0154	0.0116	-0.0325	0.0000	0.0083
AT. 26 O	X	-0.0206	0.0104	0.0155	-0.0358	-0.0113	0.0318
	Y	-0.0064	-0.0119	-0.0136	0.0202	0.0042	-0.0234
	Z	-0.0470	-0.0103	-0.0020	0.0040	-0.0377	-0.0031
AT. 27 O	X	0.0206	0.0104	0.0155	-0.0358	0.0113	0.0318
	Y	-0.0064	0.0119	0.0136	-0.0202	0.0042	0.0234
	Z	0.0470	-0.0103	-0.0020	0.0040	0.0377	-0.0031

FREQ(CM**-1)		394.51	412.93	437.18	448.61	454.01	457.35
AT. 1 CA	X	-0.0209	0.0000	-0.0001	0.0033	-0.0003	0.0000
	Y	0.0000	0.0134	0.0000	0.0000	0.0000	-0.0008
	Z	0.0061	0.0000	0.0007	-0.0045	-0.0009	0.0000
AT. 2 CA	X	-0.0010	0.0014	-0.0058	-0.0053	-0.0010	0.0009
	Y	-0.0127	0.0029	-0.0036	0.0059	0.0002	0.0012
	Z	-0.0004	-0.0046	0.0002	-0.0026	-0.0021	0.0032
AT. 3 CA	X	-0.0010	-0.0014	-0.0058	-0.0053	-0.0010	-0.0009
	Y	0.0127	0.0029	0.0036	-0.0059	-0.0002	0.0012
	Z	-0.0004	0.0046	0.0002	-0.0026	-0.0021	-0.0032
AT. 4 CA	X	0.0001	-0.0005	-0.0031	0.0029	-0.0051	0.0002
	Y	0.0030	-0.0023	0.0034	-0.0064	-0.0001	-0.0069

	Z	-0.0059	0.0036	-0.0026	0.0110	-0.0004	0.0004
AT. 5 CA	X	0.0001	0.0005	-0.0031	0.0029	-0.0051	-0.0002
	Y	-0.0030	-0.0023	-0.0034	0.0064	0.0001	-0.0069
	Z	-0.0059	-0.0036	-0.0026	0.0110	-0.0004	-0.0004
AT. 6 CA	X	0.0036	0.0000	-0.0079	0.0047	-0.0073	0.0000
	Y	0.0000	0.0044	0.0000	0.0000	0.0000	-0.0088
	Z	0.0156	0.0000	0.0093	-0.0009	-0.0088	0.0000
AT. 7 CA	X	0.0076	0.0000	0.0052	-0.0059	-0.0069	0.0000
	Y	0.0000	-0.0018	0.0000	0.0000	0.0000	-0.0073
	Z	-0.0084	0.0000	-0.0107	0.0096	0.0015	0.0000
AT. 8 CA	X	-0.0012	0.0017	-0.0006	-0.0052	-0.0063	-0.0022
	Y	-0.0018	0.0006	-0.0033	0.0054	-0.0035	-0.0045
	Z	0.0056	-0.0048	-0.0021	-0.0038	-0.0023	-0.0002
AT. 9 CA	X	-0.0012	-0.0017	-0.0006	-0.0052	-0.0063	0.0022
	Y	0.0018	0.0006	0.0033	-0.0054	0.0035	-0.0045
	Z	0.0056	0.0048	-0.0021	-0.0038	-0.0023	0.0002
AT. 10 SI	X	0.0017	0.0000	0.0052	0.0026	-0.0020	0.0000
	Y	0.0000	-0.0010	0.0000	0.0000	0.0000	0.0024
	Z	-0.0071	0.0000	0.0013	-0.0066	0.0120	0.0000
AT. 11 SI	X	0.0102	0.0000	-0.0064	-0.0025	0.0102	0.0000
	Y	0.0000	-0.0030	0.0000	0.0000	0.0000	0.0031
	Z	0.0034	0.0000	-0.0020	-0.0037	-0.0025	0.0000
AT. 12 SI	X	0.0042	0.0000	-0.0109	-0.0016	-0.0011	0.0000
	Y	0.0000	0.0173	0.0000	0.0000	0.0000	0.0012
	Z	-0.0066	0.0000	0.0067	-0.0235	0.0133	0.0000
AT. 13 O	X	0.0244	0.0000	-0.0384	-0.0097	-0.0219	0.0000
	Y	0.0000	0.0232	0.0000	0.0000	0.0000	-0.0172
	Z	-0.0019	0.0000	-0.0082	-0.0162	0.0046	0.0000
AT. 14 O	X	0.0260	-0.0294	0.0147	0.0094	0.0040	0.0080
	Y	0.0219	-0.0092	0.0086	0.0004	0.0018	0.0014
	Z	-0.0284	-0.0310	-0.0067	-0.0007	-0.0120	0.0125
AT. 15 O	X	0.0260	0.0294	0.0147	0.0094	0.0040	-0.0080
	Y	-0.0219	-0.0092	-0.0086	-0.0004	-0.0018	0.0014
	Z	-0.0284	0.0310	-0.0067	-0.0007	-0.0120	-0.0125
AT. 16 O	X	0.0067	0.0000	-0.0005	-0.0057	0.0044	0.0000
	Y	0.0000	-0.0412	0.0000	0.0000	0.0000	0.0110
	Z	0.0369	0.0000	0.0117	0.0181	0.0195	0.0000
AT. 17 O	X	-0.0291	0.0000	0.0288	0.0099	-0.0232	0.0000
	Y	0.0000	-0.0187	0.0000	0.0000	0.0000	-0.0088
	Z	0.0418	0.0000	0.0023	-0.0339	-0.0090	0.0000
AT. 18 O	X	-0.0407	0.0000	-0.0186	-0.0078	-0.0189	0.0000
	Y	0.0000	0.0477	0.0000	0.0000	0.0000	-0.0209
	Z	0.0137	0.0000	0.0032	-0.0093	0.0026	0.0000
AT. 19 O	X	0.0084	0.0000	-0.0208	-0.0121	0.0113	0.0000
	Y	0.0000	-0.0288	0.0000	0.0000	0.0000	-0.0019
	Z	-0.0191	0.0000	0.0436	0.0168	0.0090	0.0000
AT. 20 O	X	-0.0086	-0.0148	0.0323	0.0060	0.0034	-0.0078
	Y	-0.0123	-0.0047	0.0239	-0.0052	0.0041	0.0068
	Z	0.0042	-0.0304	-0.0159	0.0053	-0.0093	0.0030
AT. 21 O	X	-0.0086	0.0148	0.0323	0.0060	0.0034	0.0078
	Y	0.0123	-0.0047	-0.0239	0.0052	-0.0041	0.0068
	Z	0.0042	0.0304	-0.0159	0.0053	-0.0093	-0.0030
AT. 22 O	X	0.0394	0.0000	0.0471	0.0337	0.0234	0.0000
	Y	0.0000	-0.0566	0.0000	0.0000	0.0000	0.0222
	Z	-0.0031	0.0000	-0.0205	0.0778	0.0055	0.0000
AT. 23 O	X	-0.0333	0.0000	-0.0084	0.0032	0.0900	0.0000
	Y	0.0000	0.0321	0.0000	0.0000	0.0000	0.1103
	Z	-0.0199	0.0000	0.0270	-0.0501	0.0124	0.0000
AT. 24 O	X	-0.0181	0.0000	0.0136	0.0127	-0.0295	0.0000
	Y	0.0000	-0.0021	0.0000	0.0000	0.0000	-0.0289
	Z	-0.0195	0.0000	0.0123	-0.0154	0.0026	0.0000
AT. 25 O	X	-0.0050	0.0000	0.0212	-0.0160	-0.0075	0.0000
	Y	0.0000	-0.0106	0.0000	0.0000	0.0000	-0.0104
	Z	0.0043	0.0000	-0.0250	0.0006	0.0281	0.0000
AT. 26 O	X	0.0095	0.0162	-0.0219	-0.0031	0.0207	0.0142
	Y	-0.0036	0.0068	0.0086	0.0078	-0.0139	0.0048
	Z	0.0016	-0.0004	0.0057	0.0121	-0.0153	0.0016
AT. 27 O	X	0.0095	-0.0162	-0.0219	-0.0031	0.0207	-0.0142
	Y	0.0036	0.0068	-0.0086	-0.0078	0.0139	0.0048
	Z	0.0016	0.0004	0.0057	0.0121	-0.0153	-0.0016

FREQ(CM**-1)		494.36	497.22	504.32	508.48	527.63	529.81
AT. 1 CA	X	0.0019	0.0000	0.0027	-0.0074	0.0000	0.0014
	Y	0.0000	-0.0106	0.0000	0.0000	-0.0045	0.0000
	Z	-0.0040	0.0000	0.0023	-0.0016	0.0000	0.0025
AT. 2 CA	X	-0.0015	-0.0051	0.0020	0.0044	-0.0027	-0.0043
	Y	-0.0022	0.0012	0.0056	-0.0016	-0.0016	-0.0047
	Z	-0.0022	0.0001	0.0021	-0.0031	0.0002	0.0042

AT. 3 CA	X	-0.0015	0.0051	0.0020	0.0044	0.0027	-0.0043
	Y	0.0022	0.0012	-0.0056	0.0016	-0.0016	0.0047
	Z	-0.0022	-0.0001	0.0021	-0.0031	-0.0002	0.0042
AT. 4 CA	X	0.0049	-0.0025	0.0017	-0.0008	0.0083	-0.0029
	Y	-0.0064	0.0002	-0.0007	-0.0003	-0.0004	0.0012
	Z	0.0042	-0.0046	-0.0052	0.0028	0.0003	-0.0012
AT. 5 CA	X	0.0049	0.0025	0.0017	-0.0008	-0.0083	-0.0029
	Y	0.0064	0.0002	0.0007	0.0003	-0.0004	-0.0012
	Z	0.0042	0.0046	-0.0052	0.0028	-0.0003	-0.0012
AT. 6 CA	X	0.0009	0.0000	0.0028	-0.0031	0.0000	-0.0044
	Y	0.0000	-0.0026	0.0000	0.0000	-0.0010	0.0000
	Z	-0.0051	0.0000	0.0065	-0.0036	0.0000	0.0006
AT. 7 CA	X	-0.0073	0.0000	-0.0026	0.0014	0.0000	0.0030
	Y	0.0000	-0.0010	0.0000	0.0000	0.0102	0.0000
	Z	0.0017	0.0000	0.0014	0.0101	0.0000	-0.0001
AT. 8 CA	X	0.0004	-0.0031	-0.0004	0.0053	-0.0018	0.0045
	Y	0.0036	0.0005	0.0048	0.0015	0.0009	0.0046
	Z	-0.0056	-0.0002	0.0001	0.0004	0.0034	-0.0020
AT. 9 CA	X	0.0004	0.0031	-0.0004	0.0053	0.0018	0.0045
	Y	-0.0036	0.0005	-0.0048	-0.0015	0.0009	-0.0046
	Z	-0.0056	0.0002	0.0001	0.0004	-0.0034	-0.0020
AT. 10 SI	X	-0.0047	0.0000	-0.0249	-0.0259	0.0000	-0.0240
	Y	0.0000	0.0231	0.0000	0.0000	0.0019	0.0000
	Z	0.0152	0.0000	0.0131	-0.0159	0.0000	-0.0169
AT. 11 SI	X	0.0125	0.0000	-0.0202	-0.0183	0.0000	0.0289
	Y	0.0000	0.0359	0.0000	0.0000	0.0184	0.0000
	Z	0.0369	0.0000	0.0105	-0.0036	0.0000	-0.0045
AT. 12 SI	X	-0.0005	0.0000	-0.0182	-0.0080	0.0000	0.0065
	Y	0.0000	0.0218	0.0000	0.0000	-0.0388	0.0000
	Z	0.0184	0.0000	-0.0262	0.0253	0.0000	-0.0118
AT. 13 O	X	-0.0036	0.0000	0.0111	0.0300	0.0000	0.0326
	Y	0.0000	-0.0131	0.0000	0.0000	0.0051	0.0000
	Z	0.0129	0.0000	0.0237	-0.0064	0.0000	-0.0023
AT. 14 O	X	-0.0213	-0.0318	0.0090	0.0229	-0.0192	-0.0249
	Y	0.0125	0.0152	0.0259	0.0119	0.0040	-0.0245
	Z	-0.0310	0.0194	-0.0087	0.0121	0.0074	-0.0076
AT. 15 O	X	-0.0213	0.0318	0.0090	0.0229	0.0192	-0.0249
	Y	-0.0125	0.0152	-0.0259	-0.0119	0.0040	0.0245
	Z	-0.0310	-0.0194	-0.0087	0.0121	-0.0074	-0.0076
AT. 16 O	X	0.0236	0.0000	-0.0153	-0.0236	0.0000	0.0285
	Y	0.0000	-0.0466	0.0000	0.0000	-0.0278	0.0000
	Z	-0.0286	0.0000	-0.0175	0.0088	0.0000	0.0065
AT. 17 O	X	0.0080	0.0000	-0.0041	-0.0028	0.0000	-0.0023
	Y	0.0000	0.0033	0.0000	0.0000	-0.0189	0.0000
	Z	-0.0087	0.0000	0.0059	-0.0044	0.0000	-0.0061
AT. 18 O	X	-0.0029	0.0000	0.0188	0.0075	0.0000	-0.0310
	Y	0.0000	-0.0408	0.0000	0.0000	-0.0155	0.0000
	Z	0.0401	0.0000	0.0131	-0.0161	0.0000	0.0090
AT. 19 O	X	0.0076	0.0000	0.0047	-0.0280	0.0000	-0.0314
	Y	0.0000	-0.0194	0.0000	0.0000	-0.0128	0.0000
	Z	-0.0130	0.0000	-0.0279	-0.0164	0.0000	-0.0092
AT. 20 O	X	0.0013	-0.0176	0.0123	0.0164	-0.0068	0.0223
	Y	0.0088	0.0073	0.0293	0.0165	0.0016	0.0218
	Z	-0.0091	0.0088	-0.0102	0.0202	-0.0052	0.0198
AT. 21 O	X	0.0013	0.0176	0.0123	0.0164	0.0068	0.0223
	Y	-0.0088	0.0073	-0.0293	-0.0165	0.0016	-0.0218
	Z	-0.0091	-0.0088	-0.0102	0.0202	0.0052	0.0198
AT. 22 O	X	0.0052	0.0000	-0.0019	-0.0023	0.0000	-0.0046
	Y	0.0000	0.0070	0.0000	0.0000	-0.0038	0.0000
	Z	0.0324	0.0000	-0.0041	0.0117	0.0000	-0.0044
AT. 23 O	X	-0.0212	0.0000	0.0203	0.0023	0.0000	0.0127
	Y	0.0000	-0.0259	0.0000	0.0000	0.0272	0.0000
	Z	-0.0115	0.0000	0.0025	-0.0157	0.0000	0.0083
AT. 24 O	X	-0.0079	0.0000	0.0159	-0.0150	0.0000	0.0043
	Y	0.0000	-0.0199	0.0000	0.0000	0.0501	0.0000
	Z	0.0191	0.0000	-0.0133	0.0239	0.0000	-0.0076
AT. 25 O	X	0.0249	0.0000	-0.0279	0.0270	0.0000	-0.0059
	Y	0.0000	-0.0155	0.0000	0.0000	0.0354	0.0000
	Z	-0.0112	0.0000	-0.0105	-0.0090	0.0000	0.0063
AT. 26 O	X	-0.0056	0.0168	0.0098	0.0003	-0.0357	-0.0020
	Y	-0.0045	0.0055	-0.0100	-0.0121	-0.0118	0.0096
	Z	-0.0180	-0.0027	0.0281	-0.0295	0.0125	0.0108
AT. 27 O	X	-0.0056	-0.0168	0.0098	0.0003	0.0357	-0.0020
	Y	0.0045	0.0055	0.0100	0.0121	-0.0118	-0.0096
	Z	-0.0180	0.0027	0.0281	-0.0295	-0.0125	0.0108

FREQ(CM**-1)		531.69	534.06	545.60	821.03	855.84	864.72
AT. 1 CA	X	-0.0018	0.0000	0.0053	0.0004	-0.0015	-0.0003

	Y	0.0000	0.0048	0.0000	0.0000	0.0000	0.0000
	Z	0.0097	0.0000	-0.0038	-0.0001	0.0012	-0.0005
AT. 2 CA	X	0.0016	0.0032	-0.0032	-0.0002	0.0003	0.0006
	Y	-0.0015	-0.0002	-0.0044	0.0000	-0.0005	-0.0007
	Z	0.0027	0.0028	-0.0067	-0.0005	0.0006	-0.0012
AT. 3 CA	X	0.0016	-0.0032	-0.0032	-0.0002	0.0003	0.0006
	Y	0.0015	-0.0002	0.0044	-0.0000	0.0005	0.0007
	Z	0.0027	-0.0028	-0.0067	-0.0005	0.0006	-0.0012
AT. 4 CA	X	0.0032	0.0019	0.0052	-0.0004	0.0003	0.0002
	Y	0.0061	-0.0002	0.0021	0.0003	-0.0008	-0.0002
	Z	-0.0002	-0.0022	0.0026	0.0007	-0.0005	0.0001
AT. 5 CA	X	0.0032	-0.0019	0.0052	-0.0004	0.0003	0.0002
	Y	-0.0061	-0.0002	-0.0021	-0.0003	0.0008	0.0002
	Z	-0.0002	0.0022	0.0026	0.0007	-0.0005	0.0001
AT. 6 CA	X	0.0051	0.0000	0.0028	0.0002	-0.0005	0.0008
	Y	0.0000	-0.0099	0.0000	0.0000	0.0000	0.0000
	Z	0.0020	0.0000	0.0130	-0.0006	-0.0003	0.0015
AT. 7 CA	X	-0.0024	0.0000	-0.0018	0.0007	-0.0009	-0.0002
	Y	0.0000	0.0022	0.0000	0.0000	0.0000	0.0000
	Z	-0.0010	0.0000	0.0012	0.0010	-0.0007	0.0007
AT. 8 CA	X	-0.0028	-0.0081	0.0005	-0.0004	0.0007	-0.0002
	Y	-0.0016	0.0001	-0.0022	0.0004	0.0001	0.0000
	Z	-0.0056	-0.0008	0.0041	-0.0007	-0.0003	0.0006
AT. 9 CA	X	-0.0028	0.0081	0.0005	-0.0004	0.0007	-0.0002
	Y	0.0016	0.0001	0.0022	-0.0004	-0.0001	-0.0000
	Z	-0.0056	0.0008	0.0041	-0.0007	-0.0003	0.0006
AT. 10 SI	X	0.0128	0.0000	-0.0034	-0.0003	-0.0060	-0.0010
	Y	0.0000	0.0418	0.0000	0.0000	0.0000	0.0000
	Z	-0.0259	0.0000	0.0138	0.0005	-0.0059	-0.0002
AT. 11 SI	X	0.0018	0.0000	0.0151	0.0009	-0.0025	-0.0194
	Y	0.0000	-0.0151	0.0000	0.0000	0.0000	0.0000
	Z	0.0108	0.0000	-0.0136	-0.0131	-0.0041	-0.0143
AT. 12 SI	X	-0.0303	0.0000	-0.0307	-0.0019	0.0013	-0.0118
	Y	0.0000	-0.0049	0.0000	0.0000	0.0000	0.0000
	Z	-0.0036	0.0000	0.0005	-0.0014	-0.0004	-0.0046
AT. 13 O	X	0.0014	0.0000	-0.0042	-0.0003	0.0098	-0.0006
	Y	0.0000	-0.0390	0.0000	0.0000	0.0000	0.0000
	Z	-0.0283	0.0000	0.0178	-0.0019	-0.0386	-0.0012
AT. 14 O	X	-0.0082	0.0149	-0.0071	0.0167	-0.0010	-0.0006
	Y	0.0089	-0.0060	-0.0279	-0.0345	0.0010	0.0014
	Z	-0.0118	-0.0083	0.0152	-0.0135	0.0002	0.0010
AT. 15 O	X	-0.0082	-0.0149	-0.0071	0.0167	-0.0010	-0.0006
	Y	-0.0089	-0.0060	0.0279	0.0345	-0.0010	-0.0014
	Z	-0.0118	0.0083	0.0152	-0.0135	0.0002	0.0010
AT. 16 O	X	0.0054	0.0000	0.0093	-0.0454	0.0048	0.0333
	Y	0.0000	0.0230	0.0000	0.0000	0.0000	0.0000
	Z	-0.0142	0.0000	0.0247	-0.0071	0.0001	0.0064
AT. 17 O	X	-0.0080	0.0000	-0.0073	-0.0005	-0.0001	-0.0022
	Y	0.0000	-0.0003	0.0000	0.0000	0.0000	0.0000
	Z	-0.0092	0.0000	0.0114	0.0011	-0.0002	-0.0014
AT. 18 O	X	0.0058	0.0000	-0.0179	0.0081	0.0004	0.0030
	Y	0.0000	0.0246	0.0000	0.0000	0.0000	0.0000
	Z	0.0144	0.0000	-0.0174	0.0575	0.0052	0.0155
AT. 19 O	X	-0.0187	0.0000	0.0196	-0.0005	0.0472	0.0018
	Y	0.0000	-0.0479	0.0000	0.0000	0.0000	0.0000
	Z	0.0250	0.0000	-0.0159	-0.0006	0.0286	0.0001
AT. 20 O	X	-0.0031	-0.0398	-0.0024	-0.0005	-0.0237	-0.0007
	Y	-0.0241	0.0124	0.0080	-0.0011	0.0357	-0.0008
	Z	0.0243	0.0108	-0.0184	-0.0002	0.0094	-0.0003
AT. 21 O	X	-0.0031	0.0398	-0.0024	-0.0005	-0.0237	-0.0007
	Y	0.0241	0.0124	-0.0080	0.0011	-0.0357	0.0008
	Z	0.0243	-0.0108	-0.0184	-0.0002	0.0094	-0.0003
AT. 22 O	X	-0.0003	0.0000	-0.0004	0.0002	-0.0002	-0.0019
	Y	0.0000	-0.0033	0.0000	0.0000	0.0000	0.0000
	Z	-0.0070	0.0000	0.0053	-0.0004	0.0014	0.0014
AT. 23 O	X	0.0127	0.0000	-0.0060	-0.0012	-0.0017	-0.0028
	Y	0.0000	-0.0114	0.0000	0.0000	0.0000	0.0000
	Z	0.0122	0.0000	-0.0246	-0.0019	-0.0028	0.0003
AT. 24 O	X	0.0199	0.0000	0.0207	-0.0016	0.0012	-0.0139
	Y	0.0000	0.0011	0.0000	0.0000	0.0000	0.0000
	Z	0.0200	0.0000	0.0222	0.0038	-0.0010	0.0349
AT. 25 O	X	-0.0072	0.0000	0.0076	0.0013	0.0019	-0.0169
	Y	0.0000	0.0142	0.0000	0.0000	0.0000	0.0000
	Z	-0.0270	0.0000	-0.0335	-0.0012	0.0013	-0.0183
AT. 26 O	X	0.0150	-0.0082	0.0060	0.0018	-0.0032	0.0254
	Y	-0.0330	-0.0018	-0.0280	0.0023	-0.0036	0.0357
	Z	0.0077	-0.0047	-0.0008	-0.0017	-0.0002	-0.0064
AT. 27 O	X	0.0150	0.0082	0.0060	0.0018	-0.0032	0.0254
	Y	0.0330	-0.0018	0.0280	-0.0023	0.0036	-0.0357

	Z	0.0077	0.0047	-0.0008	-0.0017	-0.0002	-0.0064
FREQ(CM**-1)		872.29	892.14	906.89	909.69	915.13	929.03
AT. 1 CA	X	0.0005	0.0000	0.0003	0.0002	0.0000	0.0003
	Y	0.0000	-0.0008	0.0000	0.0000	0.0006	0.0000
	Z	-0.0000	0.0000	-0.0006	-0.0003	0.0000	-0.0002
AT. 2 CA	X	0.0005	-0.0007	-0.0007	-0.0002	0.0003	0.0005
	Y	0.0015	-0.0001	0.0001	-0.0001	0.0000	-0.0002
	Z	0.0004	-0.0001	-0.0006	0.0005	-0.0006	0.0002
AT. 3 CA	X	0.0005	0.0007	-0.0007	-0.0002	-0.0003	0.0005
	Y	-0.0015	-0.0001	-0.0001	0.0001	0.0000	0.0002
	Z	0.0004	0.0001	-0.0006	0.0005	0.0006	0.0002
AT. 4 CA	X	-0.0000	-0.0001	0.0001	-0.0004	-0.0005	-0.0004
	Y	-0.0001	0.0001	-0.0001	0.0001	-0.0001	0.0002
	Z	0.0002	-0.0008	0.0009	-0.0002	0.0008	0.0000
AT. 5 CA	X	-0.0000	0.0001	0.0001	-0.0004	0.0005	-0.0004
	Y	0.0001	0.0001	0.0001	-0.0001	-0.0001	-0.0002
	Z	0.0002	0.0008	0.0009	-0.0002	-0.0008	0.0000
AT. 6 CA	X	-0.0015	0.0000	-0.0014	-0.0002	0.0000	0.0002
	Y	0.0000	0.0001	0.0000	0.0000	0.0006	0.0000
	Z	-0.0025	0.0000	0.0004	0.0001	0.0000	-0.0005
AT. 7 CA	X	-0.0000	0.0000	0.0002	0.0004	0.0000	0.0003
	Y	0.0000	-0.0000	0.0000	0.0000	-0.0008	0.0000
	Z	0.0017	0.0000	0.0001	0.0004	0.0000	0.0001
AT. 8 CA	X	0.0005	0.0001	0.0004	0.0009	0.0002	-0.0002
	Y	-0.0010	-0.0002	-0.0009	0.0005	-0.0001	0.0004
	Z	0.0001	0.0002	0.0005	-0.0003	-0.0007	0.0001
AT. 9 CA	X	0.0005	-0.0001	0.0004	0.0009	-0.0002	-0.0002
	Y	0.0010	-0.0002	0.0009	-0.0005	-0.0001	-0.0004
	Z	0.0001	-0.0002	0.0005	-0.0003	0.0007	0.0001
AT. 10 SI	X	-0.0011	0.0000	0.0030	-0.0433	0.0000	0.0021
	Y	0.0000	-0.0012	0.0000	0.0000	-0.0300	0.0000
	Z	0.0024	0.0000	0.0038	-0.0047	0.0000	-0.0324
AT. 11 SI	X	-0.0337	0.0000	0.0233	0.0035	0.0000	-0.0012
	Y	0.0000	0.0439	0.0000	0.0000	-0.0100	0.0000
	Z	-0.0188	0.0000	-0.0369	-0.0025	0.0000	0.0033
AT. 12 SI	X	0.0073	0.0000	-0.0015	0.0044	0.0000	0.0098
	Y	0.0000	0.0123	0.0000	0.0000	0.0312	0.0000
	Z	-0.0005	0.0000	-0.0074	0.0034	0.0000	-0.0283
AT. 13 O	X	0.0004	0.0000	-0.0004	0.0020	0.0000	-0.0121
	Y	0.0000	0.0001	0.0000	0.0000	0.0008	0.0000
	Z	0.0019	0.0000	-0.0016	-0.0019	0.0000	0.0448
AT. 14 O	X	0.0039	0.0193	-0.0159	-0.0018	-0.0035	0.0026
	Y	-0.0057	-0.0387	0.0334	0.0029	0.0086	-0.0009
	Z	-0.0012	-0.0177	0.0171	0.0016	0.0043	-0.0014
AT. 15 O	X	0.0039	-0.0193	-0.0159	-0.0018	0.0035	0.0026
	Y	0.0057	-0.0387	-0.0334	-0.0029	0.0086	0.0009
	Z	-0.0012	0.0177	0.0171	0.0016	-0.0043	-0.0014
AT. 16 O	X	0.0497	0.0000	-0.0120	-0.0027	0.0000	-0.0011
	Y	0.0000	-0.0009	0.0000	0.0000	0.0006	0.0000
	Z	0.0099	0.0000	-0.0000	-0.0014	0.0000	-0.0006
AT. 17 O	X	-0.0036	0.0000	0.0033	0.0016	0.0000	0.0009
	Y	0.0000	0.0047	0.0000	0.0000	-0.0010	0.0000
	Z	-0.0018	0.0000	-0.0008	-0.0010	0.0000	-0.0006
AT. 18 O	X	0.0053	0.0000	0.0032	-0.0003	0.0000	0.0003
	Y	0.0000	-0.0027	0.0000	0.0000	0.0007	0.0000
	Z	0.0276	0.0000	0.0312	0.0021	0.0000	-0.0031
AT. 19 O	X	-0.0045	0.0000	-0.0055	0.0388	0.0000	0.0130
	Y	0.0000	0.0006	0.0000	0.0000	0.0011	0.0000
	Z	-0.0036	0.0000	-0.0046	0.0247	0.0000	0.0096
AT. 20 O	X	0.0033	-0.0003	-0.0003	0.0201	-0.0174	-0.0038
	Y	-0.0059	0.0011	-0.0005	-0.0290	0.0275	0.0050
	Z	-0.0012	0.0007	-0.0012	-0.0071	0.0065	0.0018
AT. 21 O	X	0.0033	0.0003	-0.0003	0.0201	0.0174	-0.0038
	Y	0.0059	0.0011	0.0005	0.0290	0.0275	-0.0050
	Z	-0.0012	-0.0007	-0.0012	-0.0071	-0.0065	0.0018
AT. 22 O	X	-0.0035	0.0000	0.0019	-0.0023	0.0000	0.0011
	Y	0.0000	0.0033	0.0000	0.0000	-0.0025	0.0000
	Z	0.0017	0.0000	0.0025	-0.0005	0.0000	0.0003
AT. 23 O	X	0.0011	0.0000	-0.0013	-0.0049	0.0000	-0.0011
	Y	0.0000	0.0012	0.0000	0.0000	0.0017	0.0000
	Z	-0.0018	0.0000	-0.0026	0.0014	0.0000	-0.0013
AT. 24 O	X	0.0057	0.0000	-0.0018	0.0008	0.0000	-0.0182
	Y	0.0000	-0.0018	0.0000	0.0000	-0.0008	0.0000
	Z	-0.0163	0.0000	0.0062	-0.0004	0.0000	0.0355
AT. 25 O	X	0.0153	0.0000	0.0119	-0.0056	0.0000	0.0128
	Y	0.0000	-0.0006	0.0000	0.0000	-0.0013	0.0000
	Z	0.0128	0.0000	0.0075	-0.0057	0.0000	0.0122

```
 AT.  26 O  X    -0.0154   -0.0072   -0.0028   -0.0023   -0.0192   -0.0063
            Y    -0.0230   -0.0112   -0.0031   -0.0002   -0.0281   -0.0081
            Z     0.0025    0.0027   -0.0009   -0.0001    0.0036    0.0017
 AT.  27 O  X    -0.0154    0.0072   -0.0028   -0.0023    0.0192   -0.0063
            Y     0.0230   -0.0112    0.0031    0.0002   -0.0281    0.0081
            Z     0.0025   -0.0027   -0.0009   -0.0001   -0.0036    0.0017

 FREQ(CM**-1)    930.28    976.68    987.06

 AT.   1 CA X     0.0000    0.0005   -0.0005
            Y     0.0006    0.0000    0.0000
            Z     0.0000    0.0009   -0.0001
 AT.   2 CA X     0.0004   -0.0001    0.0005
            Y    -0.0000    0.0003   -0.0007
            Z     0.0004    0.0007   -0.0001
 AT.   3 CA X    -0.0004   -0.0001    0.0005
            Y    -0.0000   -0.0003    0.0007
            Z    -0.0004    0.0007   -0.0001
 AT.   4 CA X    -0.0004    0.0009    0.0004
            Y    -0.0003    0.0008    0.0001
            Z    -0.0003   -0.0007    0.0015
 AT.   5 CA X     0.0004    0.0009    0.0004
            Y    -0.0003   -0.0008   -0.0001
            Z     0.0003   -0.0007    0.0015
 AT.   6 CA X     0.0000   -0.0004   -0.0000
            Y    -0.0009    0.0000    0.0000
            Z     0.0000   -0.0018   -0.0005
 AT.   7 CA X     0.0000   -0.0002   -0.0003
            Y    -0.0009    0.0000    0.0000
[proxy:0:0@lmfz640] got pmi command (from 13): finalize

            Z     0.0000   -0.0002    0.0011
 AT.   8 CA X    -0.0002    0.0000    0.0001
            Y    -0.0003    0.0001   -0.0002
            Z    -0.0005   -0.0006   -0.0009
[proxy:0:0@lmfz640] PMI response: cmd=finalize_ack
 AT.   9 CA X     0.0002    0.0000    0.0001
            Y    -0.0003   -0.0001    0.0002
            Z     0.0005   -0.0006   -0.0009
 AT.  10 SI X     0.0000   -0.0050    0.0043
            Y     0.0319    0.0000    0.0000
            Z     0.0000    0.0013   -0.0277
 AT.  11 SI X     0.0000   -0.0011    0.0010
            Y    -0.0077    0.0000    0.0000
            Z     0.0000    0.0052   -0.0073
 AT.  12 SI X     0.0000   -0.0375   -0.0118
            Y     0.0287    0.0000    0.0000
            Z     0.0000   -0.0120    0.0278
 AT.  13 O  X     0.0000    0.0016   -0.0073
            Y    -0.0013    0.0000    0.0000
            Z     0.0000   -0.0031    0.0415
 AT.  14 O  X    -0.0024    0.0005   -0.0025
            Y     0.0066   -0.0027    0.0036
            Z     0.0040   -0.0020    0.0016
 AT.  15 O  X     0.0024    0.0005   -0.0025
            Y     0.0066    0.0027   -0.0036
            Z    -0.0040   -0.0020    0.0016
 AT.  16 O  X     0.0000    0.0007    0.0018
            Y     0.0011    0.0000    0.0000
            Z     0.0000   -0.0016    0.0006
 AT.  17 O  X     0.0000    0.0000    0.0005
            Y    -0.0016    0.0000    0.0000
[proxy:0:0@lmfz640] got pmi command (from 21): finalize

[proxy:0:0@lmfz640] PMI response: cmd=finalize_ack
            Z     0.0000    0.0000   -0.0031
 AT.  18 O  X     0.0000    0.0004    0.0010
            Y    -0.0002    0.0000    0.0000
            Z     0.0000   -0.0057    0.0094
 AT.  19 O  X     0.0000    0.0047    0.0093
            Y     0.0004    0.0000    0.0000
            Z     0.0000    0.0032    0.0078
 AT.  20 O  X     0.0191    0.0011   -0.0045
            Y    -0.0289   -0.0042    0.0066
            Z    -0.0080   -0.0006    0.0038
 AT.  21 O  X    -0.0191    0.0011   -0.0045
            Y    -0.0289    0.0042   -0.0066
            Z     0.0080   -0.0006    0.0038
 AT.  22 O  X     0.0000   -0.0020    0.0007
```

```
           Y     0.0024    0.0000    0.0000
           Z     0.0000   -0.0011    0.0042
 AT.  23 O  X     0.0000   -0.0057   -0.0003
           Y     0.0096    0.0000    0.0000
           Z     0.0000    0.0024   -0.0007
 AT.  24 O  X     0.0000    0.0060    0.0155
           Y    -0.0012    0.0000    0.0000
           Z     0.0000   -0.0135   -0.0408
 AT.  25 O  X     0.0000    0.0459   -0.0080
           Y     0.0004    0.0000    0.0000
           Z     0.0000    0.0431   -0.0093
 AT.  26 O  X    -0.0181    0.0095    0.0062
           Y    -0.0255    0.0127    0.0083
           Z     0.0042   -0.0007   -0.0038
 AT.  27 O  X     0.0181    0.0095    0.0062
           Y    -0.0255   -0.0127   -0.0083
           Z    -0.0042   -0.0007   -0.0038

[proxy:0:0@lmfz640] got pmi command (from 0): finalize

[proxy:0:0@lmfz640] PMI response: cmd=finalize_ack
 ESTIMATED NUMBER OF IMAGINARY FREQS    1


 ******************************************************************************

 VIBRATIONAL TEMPERATURES (K) [MODE NUMBER;IRREP]

 TO MODES
    20.8 [   4;A' ]    73.2 [   5;A' ]    73.3 [   6;B" ]   117.2 [   7;B" ]
   141.8 [   8;A' ]   168.4 [   9;A' ]   178.2 [  10;B" ]   184.9 [  11;B" ]
   198.1 [  12;A' ]   205.2 [  13;B" ]   222.5 [  14;A' ]   225.9 [  15;A' ]
   229.7 [  16;B" ]   242.5 [  17;B" ]   249.7 [  18;A' ]   254.9 [  19;B" ]
   259.1 [  20;A' ]   266.1 [  21;B" ]   284.6 [  22;A' ]   284.7 [  23;B" ]
   287.0 [  24;B" ]   303.3 [  25;A' ]   307.9 [  26;A' ]   312.7 [  27;B" ]
   317.7 [  28;A' ]   325.9 [  29;A' ]   352.9 [  30;B" ]   353.9 [  31;A' ]
   367.1 [  32;B" ]   371.3 [  33;A' ]   374.6 [  34;A' ]   387.2 [  35;A' ]
   388.2 [  36;A' ]   410.8 [  37;B" ]   419.3 [  38;A' ]   423.9 [  39;B" ]
   424.8 [  40;B" ]   434.1 [  41;A' ]   450.1 [  42;B" ]   454.1 [  43;B" ]
   456.9 [  44;B" ]   457.8 [  45;A' ]   469.9 [  46;A' ]   477.7 [  47;A' ]
   489.7 [  48;B" ]   498.2 [  49;B" ]   501.2 [  50;A' ]   512.1 [  51;A' ]
   531.9 [  52;A' ]   548.2 [  53;B" ]   559.6 [  54;A' ]   567.6 [  55;A' ]
   594.1 [  56;B" ]   629.0 [  57;A' ]   645.4 [  58;A' ]   653.2 [  59;A' ]
   658.0 [  60;B" ]   711.3 [  61;A' ]   715.4 [  62;B" ]   725.6 [  63;A' ]
[proxy:0:0@lmfz640] got pmi command (from 6): finalize

[proxy:0:0@lmfz640] PMI response: cmd=finalize_ack
   731.6 [  64;A' ]   759.1 [  65;B" ]   762.3 [  66;A' ]   765.0 [  67;A' ]
   768.4 [  68;B" ]   785.0 [  69;A' ]  1181.3 [  70;A' ]  1231.4 [  71;A' ]
  1244.1 [  72;A' ]  1255.0 [  73;A' ]  1283.6 [  74;B" ]  1304.8 [  75;A' ]
  1308.8 [  76;A' ]  1316.7 [  77;B" ]  1336.7 [  78;A' ]  1338.5 [  79;B" ]
  1405.2 [  80;A' ]  1420.2 [  81;A' ]

 ******************************************************************************

 HARMONIC VIBRATIONAL CONTRIBUTIONS TO THERMODYNAMIC FUNCTIONS AT GIVEN
 TEMPERATURE AND PRESSURE:

 (EL = ELECTRONIC ENERGY
  E0 = ZERO-POINT ENERGY
  ET = THERMAL CONTRIBUTION TO THE VIBRATIONAL ENERGY
  PV = PRESSURE * VOLUME
  TS = TEMPERATURE * ENTROPY)

                         AU/CELL             EV/CELL                 KJ/MOL
 EL            :   -8070.316905547273 -219604.487476348411   -21188614.04949711
 E0            :       0.068004327334       1.850491823936         178.54533625


 ******************************************************************************

 THERMODYNAMIC FUNCTIONS WITH VIBRATIONAL CONTRIBUTIONS

 AT (T =  298.15 K, P =   0.10132500E+00 MPA):

                         AU/CELL             EV/CELL                 KJ/MOL
[proxy:0:0@lmfz640] got pmi command (from 17): finalize

[proxy:0:0@lmfz640] PMI response: cmd=finalize_ack
```

```
 ET            :       0.031216939561       0.849456110970          81.96006327
 PV            :       0.000008381641       0.000228076055           0.02200600
 TS            :       0.057876292596       1.574893987598         151.95418480
 ET+PV-TS      :      -0.026650971394      -0.725209800573         -69.97211553
 EL+E0+ET+PV-TS:   -8070.275552191333 -219603.362194325076   -21188505.47627639

 OTHER THERMODINAMIC FUNCTIONS:

                     mHARTREE/(CELL*K)     mEV/(CELL*K)              J/(MOL*K)
 ENTROPY       :       0.194118036545       5.282220317284         509.65683313
 HEAT CAPACITY :       0.184274756063       5.014371038166         483.81330386

 *******************************************************************************
 TTTTTTTTTTTTTTTTTTTTTTTTTTTTTT END         TELAPSE    25568.56 TCPU    25565.67
 EEEEEEEEEE TERMINATION  DATE 11 10 2019 TIME 21:20:34.3
NODE    0 CPU TIME =     25565.674
NODE    1 CPU TIME =     25565.263
NODE    2 CPU TIME =     25565.408
NODE    3 CPU TIME =     25565.409
NODE    4 CPU TIME =     25562.682
NODE    5 CPU TIME =     25565.759
NODE    6 CPU TIME =     25563.302
NODE    7 CPU TIME =     25565.073
    TOTAL CPU TIME =    204518.570
[proxy:0:0@lmfz640] got pmi command (from 25): finalize

[proxy:0:0@lmfz640] PMI response: cmd=finalize_ack
[proxy:0:0@lmfz640] got pmi command (from 29): finalize

[proxy:0:0@lmfz640] PMI response: cmd=finalize_ack
[proxy:0:0@lmfz640] got pmi command (from 8): finalize

[proxy:0:0@lmfz640] PMI response: cmd=finalize_ack
-----------------------------------------------
total 462532
-rw-rw-r-- 1 vlasta vlasta        0 lis  11 14:14 dffit3.dat
-rw-rw-r-- 1 vlasta vlasta     5978 lis  11 21:19 ERROR
-rw-rw-r-- 1 vlasta vlasta     5978 lis  11 21:19 ERROR.pe1
-rw-rw-r-- 1 vlasta vlasta     5978 lis  11 21:19 ERROR.pe2
-rw-rw-r-- 1 vlasta vlasta     5978 lis  11 21:19 ERROR.pe3
-rw-rw-r-- 1 vlasta vlasta     5978 lis  11 21:19 ERROR.pe4
-rw-rw-r-- 1 vlasta vlasta     5978 lis  11 21:19 ERROR.pe5
-rw-rw-r-- 1 vlasta vlasta     5978 lis  11 21:19 ERROR.pe6
-rw-rw-r-- 1 vlasta vlasta     5978 lis  11 21:19 ERROR.pe7
-rw-rw-r-- 1 vlasta vlasta    27160 lis  11 21:20 fort.10.pe0
-rw-rw-r-- 1 vlasta vlasta 27373856 lis  11 21:16 fort.10.pe1
-rw-rw-r-- 1 vlasta vlasta 27373856 lis  11 21:16 fort.10.pe2
-rw-rw-r-- 1 vlasta vlasta 23952128 lis  11 21:16 fort.10.pe3
-rw-rw-r-- 1 vlasta vlasta 30795592 lis  11 21:16 fort.10.pe4
-rw-rw-r-- 1 vlasta vlasta 27373864 lis  11 21:16 fort.10.pe5
-rw-rw-r-- 1 vlasta vlasta 27373864 lis  11 21:16 fort.10.pe6
-rw-rw-r-- 1 vlasta vlasta 30795592 lis  11 21:16 fort.10.pe7
-rw-rw-r-- 1 vlasta vlasta  1394664 lis  11 21:17 fort.11.pe0
-rw-rw-r-- 1 vlasta vlasta  1394664 lis  11 21:17 fort.11.pe1
-rw-rw-r-- 1 vlasta vlasta  1394664 lis  11 21:17 fort.11.pe2
-rw-rw-r-- 1 vlasta vlasta  1394664 lis  11 21:17 fort.11.pe3
-rw-rw-r-- 1 vlasta vlasta  1394664 lis  11 21:17 fort.11.pe4
-rw-rw-r-- 1 vlasta vlasta  1394664 lis  11 21:17 fort.11.pe5
-rw-rw-r-- 1 vlasta vlasta  1394664 lis  11 21:17 fort.11.pe6
-rw-rw-r-- 1 vlasta vlasta  1394664 lis  11 21:17 fort.11.pe7
-rw-rw-r-- 1 vlasta vlasta    50480 lis  11 21:11 fort.12
-rw-rw-r-- 1 vlasta vlasta  2764696 lis  11 14:21 fort.13
-rw-rw-r-- 1 vlasta vlasta        0 lis  11 14:14 fort.17.pe1
-rw-rw-r-- 1 vlasta vlasta        0 lis  11 14:14 fort.17.pe2
-rw-rw-r-- 1 vlasta vlasta        0 lis  11 14:14 fort.17.pe3
-rw-rw-r-- 1 vlasta vlasta        0 lis  11 14:14 fort.17.pe4
-rw-rw-r-- 1 vlasta vlasta        0 lis  11 14:14 fort.17.pe5
-rw-rw-r-- 1 vlasta vlasta        0 lis  11 14:14 fort.17.pe6
-rw-rw-r-- 1 vlasta vlasta        0 lis  11 14:14 fort.17.pe7
-rw-rw-r-- 1 vlasta vlasta        0 lis  11 14:14 fort.18.pe0
-rw-rw-r-- 1 vlasta vlasta        0 lis  11 14:14 fort.18.pe1
-rw-rw-r-- 1 vlasta vlasta        0 lis  11 14:14 fort.18.pe2
-rw-rw-r-- 1 vlasta vlasta        0 lis  11 14:14 fort.18.pe3
-rw-rw-r-- 1 vlasta vlasta        0 lis  11 14:14 fort.18.pe4
-rw-rw-r-- 1 vlasta vlasta        0 lis  11 14:14 fort.18.pe5
-rw-rw-r-- 1 vlasta vlasta        0 lis  11 14:14 fort.18.pe6
-rw-rw-r-- 1 vlasta vlasta        0 lis  11 14:14 fort.18.pe7
-rw-rw-r-- 1 vlasta vlasta     1776 lis  11 21:20 fort.19.pe0
-rw-rw-r-- 1 vlasta vlasta   157248 lis  11 21:20 fort.19.pe1
```

```
-rw-rw-r-- 1 vlasta vlasta   157248 lis  11 21:20 fort.19.pe2
-rw-rw-r-- 1 vlasta vlasta   157248 lis  11 21:20 fort.19.pe3
-rw-rw-r-- 1 vlasta vlasta   157248 lis  11 21:20 fort.19.pe4
-rw-rw-r-- 1 vlasta vlasta   157248 lis  11 21:20 fort.19.pe5
-rw-rw-r-- 1 vlasta vlasta   157248 lis  11 21:20 fort.19.pe6
-rw-rw-r-- 1 vlasta vlasta   157248 lis  11 21:20 fort.19.pe7
-rw-rw-r-- 1 vlasta vlasta        0 lis  11 14:14 fort.1.pe0
-rw-rw-r-- 1 vlasta vlasta        0 lis  11 14:14 fort.1.pe1
-rw-rw-r-- 1 vlasta vlasta        0 lis  11 14:14 fort.1.pe2
-rw-rw-r-- 1 vlasta vlasta        0 lis  11 14:14 fort.1.pe3
-rw-rw-r-- 1 vlasta vlasta        0 lis  11 14:14 fort.1.pe4
-rw-rw-r-- 1 vlasta vlasta        0 lis  11 14:14 fort.1.pe5
-rw-rw-r-- 1 vlasta vlasta        0 lis  11 14:14 fort.1.pe6
-rw-rw-r-- 1 vlasta vlasta        0 lis  11 14:14 fort.1.pe7
-rw-rw-r-- 1 vlasta vlasta  2174324 lis  11 14:21 fort.20
-rw-rw-r-- 1 vlasta vlasta        0 lis  11 14:14 fort.28
-rw-rw-r-- 1 vlasta vlasta        0 lis  11 14:14 fort.29
-rw-rw-r-- 1 vlasta vlasta        0 lis  11 14:14 fort.38.pe0
-rw-rw-r-- 1 vlasta vlasta        0 lis  11 14:14 fort.38.pe1
-rw-rw-r-- 1 vlasta vlasta        0 lis  11 14:14 fort.38.pe2
-rw-rw-r-- 1 vlasta vlasta        0 lis  11 14:14 fort.38.pe3
-rw-rw-r-- 1 vlasta vlasta        0 lis  11 14:14 fort.38.pe4
-rw-rw-r-- 1 vlasta vlasta        0 lis  11 14:14 fort.38.pe5
-rw-rw-r-- 1 vlasta vlasta        0 lis  11 14:14 fort.38.pe6
-rw-rw-r-- 1 vlasta vlasta        0 lis  11 14:14 fort.38.pe7
-rw-rw-r-- 1 vlasta vlasta        0 lis  11 14:14 fort.3.pe0
-rw-rw-r-- 1 vlasta vlasta        0 lis  11 14:14 fort.3.pe1
-rw-rw-r-- 1 vlasta vlasta        0 lis  11 14:14 fort.3.pe2
-rw-rw-r-- 1 vlasta vlasta        0 lis  11 14:14 fort.3.pe3
-rw-rw-r-- 1 vlasta vlasta        0 lis  11 14:14 fort.3.pe4
-rw-rw-r-- 1 vlasta vlasta        0 lis  11 14:14 fort.3.pe5
-rw-rw-r-- 1 vlasta vlasta        0 lis  11 14:14 fort.3.pe6
-rw-rw-r-- 1 vlasta vlasta        0 lis  11 14:14 fort.3.pe7
-rw-rw-r-- 1 vlasta vlasta   534896 lis  11 21:19 fort.40.pe0
-rw-rw-r-- 1 vlasta vlasta   534896 lis  11 21:19 fort.40.pe1
-rw-rw-r-- 1 vlasta vlasta   534896 lis  11 21:19 fort.40.pe2
-rw-rw-r-- 1 vlasta vlasta   534896 lis  11 21:19 fort.40.pe3
-rw-rw-r-- 1 vlasta vlasta   534896 lis  11 21:19 fort.40.pe4
-rw-rw-r-- 1 vlasta vlasta   534896 lis  11 21:19 fort.40.pe5
-rw-rw-r-- 1 vlasta vlasta   534896 lis  11 21:19 fort.40.pe6
-rw-rw-r-- 1 vlasta vlasta   534896 lis  11 21:19 fort.40.pe7
-rw-rw-r-- 1 vlasta vlasta 23952128 lis  11 21:17 fort.8.pe0
-rw-rw-r-- 1 vlasta vlasta 27373856 lis  11 21:17 fort.8.pe1
-rw-rw-r-- 1 vlasta vlasta 27373856 lis  11 21:17 fort.8.pe2
-rw-rw-r-- 1 vlasta vlasta 23952128 lis  11 21:17 fort.8.pe3
-rw-rw-r-- 1 vlasta vlasta 30795592 lis  11 21:17 fort.8.pe4
-rw-rw-r-- 1 vlasta vlasta 27373864 lis  11 21:17 fort.8.pe5
-rw-rw-r-- 1 vlasta vlasta 27373864 lis  11 21:17 fort.8.pe6
-rw-rw-r-- 1 vlasta vlasta 30795592 lis  11 21:17 fort.8.pe7
-rw-rw-r-- 1 vlasta vlasta  2174324 lis  11 14:21 fort.9
-rw-rw-r-- 1 vlasta vlasta  1434280 lis  11 21:16 fort.95.pe0
-rw-rw-r-- 1 vlasta vlasta  1434280 lis  11 21:16 fort.95.pe1
-rw-rw-r-- 1 vlasta vlasta  1434280 lis  11 21:16 fort.95.pe2
-rw-rw-r-- 1 vlasta vlasta  1434280 lis  11 21:16 fort.95.pe3
-rw-rw-r-- 1 vlasta vlasta  1434280 lis  11 21:16 fort.95.pe4
-rw-rw-r-- 1 vlasta vlasta  1434280 lis  11 21:16 fort.95.pe5
-rw-rw-r-- 1 vlasta vlasta  1434280 lis  11 21:16 fort.95.pe6
-rw-rw-r-- 1 vlasta vlasta  1434280 lis  11 21:16 fort.95.pe7
-rw-rw-r-- 1 vlasta vlasta 11512815 lis  11 21:20 FREQINFO.DAT
-rw-rw-r-- 1 vlasta vlasta   139422 lis  11 21:20 HESSFREQ.DAT
-rw-rw-r-- 1 vlasta vlasta     3564 lis  11 14:14 INPUT
-rw-rw-r-- 1 vlasta vlasta       64 lis  11 14:14 machines
-rwxr-xr-x 1 vlasta vlasta 11332453 lis  11 14:14 Pcrystal
-rw-rw-r-- 1 vlasta vlasta  1198932 lis  11 21:20 SCFOUT.LOG
-----------------------------------------------
=:= Removing scratch directories on:  ...
=:= finished pet, 11.10.2019. 21:20:34 CEST
=:= Elapsed time: 7h:06m:09s
```

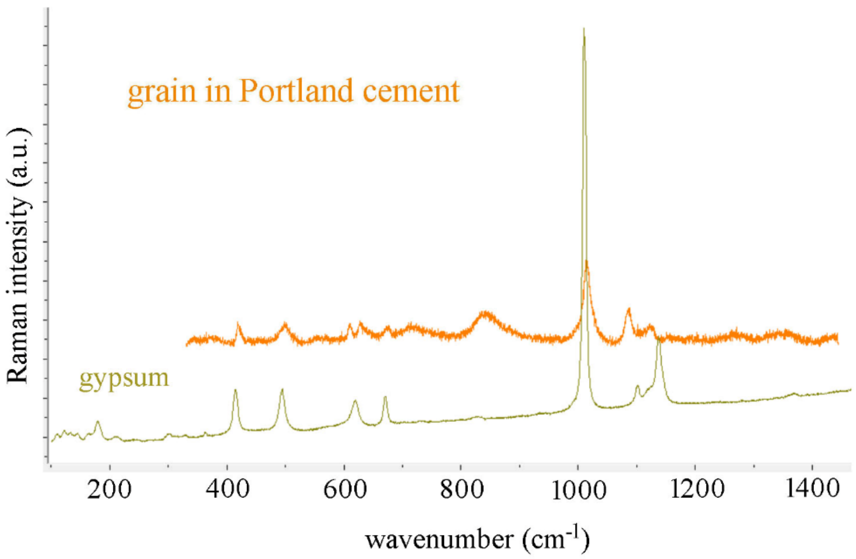

Figure S1. Comparison of Raman spectra of gypsum and Portland cement.

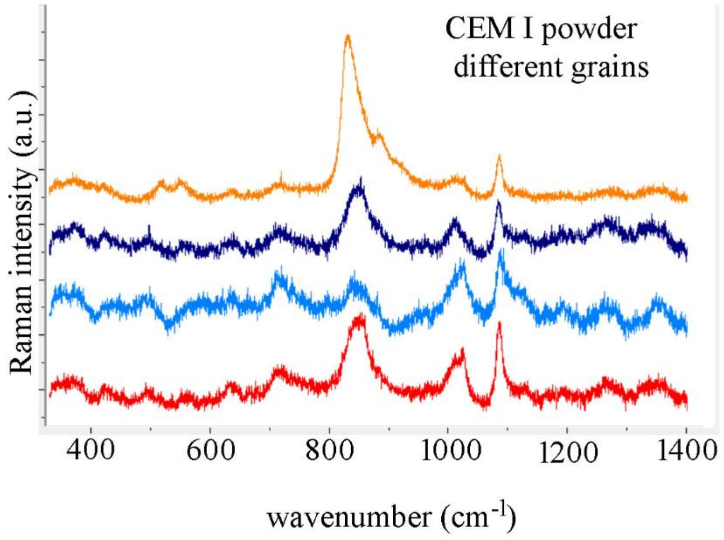

Figure S2. Raman spectra of CEM I powder, collected at four different sites.

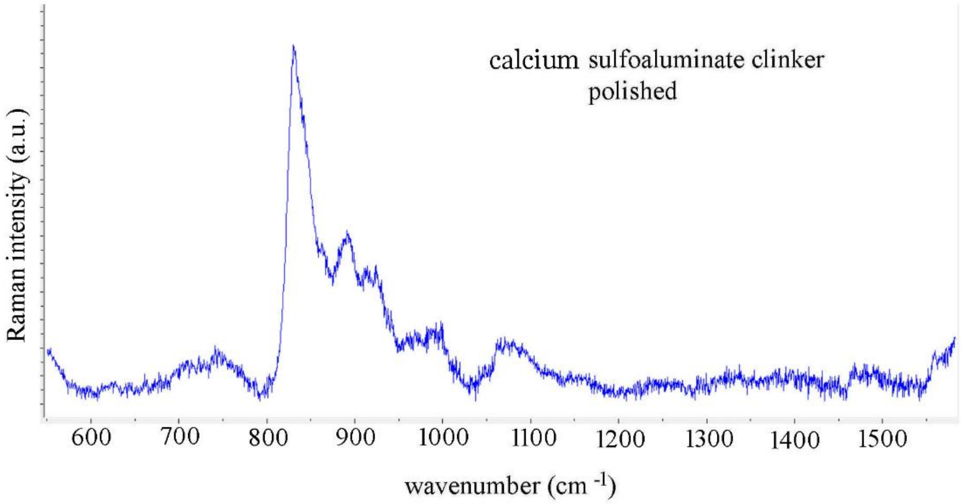

Figure S3. Raman spectrum of polished sample of calcium sulfoaluminate clinker.
